# The haplotype-resolved T2T genome for *Bauhinia × blakeana* sheds light on the genetic basis of flower heterosis

**DOI:** 10.1093/gigascience/giaf044

**Published:** 2025-04-25

**Authors:** Weixue Mu, Joshua Casey Darian, Wing-Kin Sung, Xing Guo, Tuo Yang, Mandy Wai Man Tang, Ziqiang Chen, Steve Kwan Hok Tong, Irene Wing Shan Chik, Robert L Davidson, Scott C Edmunds, Tong Wei, Stephen Kwok-Wing Tsui

**Affiliations:** School of Biomedical Sciences, The Chinese University of Hong Kong, Shatin, N.T., Hong Kong SAR, China; Hong Kong Bioinformatics Centre, The Chinese University of Hong Kong, Shatin, N.T., Hong Kong SAR, China; School of Computing, National University of Singapore, Singapore 117417; Department of Chemical Pathology, The Chinese University of Hong Kong, Shatin, N.T., Hong Kong SAR, China; JC STEM Laboratory of Computational Genomics, Li Ka Shing Institute of Health Sciences, The Chinese University of Hong Kong, Shatin, N.T., Hong Kong SAR, China; Hong Kong Genome Institute, Hong Kong Science Park, Shatin, N.T., Hong Kong SAR, China; BGI Research, East Lake High-Tech Development Zone, Wuhan 430074, China; Key Laboratory of Southern Subtropical Plant Diversity, Fairy Lake Botanical Garden, Shenzhen & Chinese Academy of Sciences, Shenzhen 518004, China; School of Biomedical Sciences, The Chinese University of Hong Kong, Shatin, N.T., Hong Kong SAR, China; National Key Laboratory for Germplasm Innovation & Utilization of Horticultural Crops, College of Horticulture & Forestry Sciences, Huazhong Agricultural University, Wuhan 430070, China; BGI Genomics, Tai Po, N.T., Hong Kong SAR, China; International DNA Research Centre, Kowloon, Hong Kong SAR, China; BGI Genomics, Tai Po, N.T., Hong Kong SAR, China; School of Physics, Engineering & Computer Science, University of Hertfordshire, Hatfield AL10 9AB, United Kingdom; GigaScience Press, BGI Hong Kong Tech Co. Ltd., Sheung Wan, Hong Kong SAR, China; BGI Research, East Lake High-Tech Development Zone, Wuhan 430074, China; School of Biomedical Sciences, The Chinese University of Hong Kong, Shatin, N.T., Hong Kong SAR, China; Hong Kong Bioinformatics Centre, The Chinese University of Hong Kong, Shatin, N.T., Hong Kong SAR, China

**Keywords:** *Bauhinia × blakeana*, trio-binning, genome evolution, transcriptome profiling, flower heterosis

## Abstract

**Background:**

The Hong Kong orchid tree *Bauhinia × blakeana* Dunn has long been proposed to be a sterile interspecific hybrid exhibiting flower heterosis when compared to its likely parental species, *Bauhinia purpurea* L. and *Bauhinia variegata* L. Here, we report comparative genomic and transcriptomic analyses of the 3 *Bauhinia* species.

**Findings:**

We generated chromosome-level assemblies for the parental species and applied a trio-binning approach to construct a haplotype-resolved telomere-to-telomere (T2T) genome for *B. blakeana*. Comparative chloroplast genome analysis confirmed *B. purpurea* as the maternal parent. Transcriptome profiling of flower tissues highlighted a closer resemblance of *B. blakeana* to its maternal parent. Differential gene expression analyses revealed distinct expression patterns among the 3 species, particularly in biosynthetic and metabolic processes. To investigate the genetic basis of flower heterosis observed in *B. blakeana*, we focused on gene expression patterns within pigment biosynthesis-related pathways. High-parent dominance and overdominance expression patterns were observed, particularly in genes associated with carotenoid biosynthesis. Additionally, allele-specific expression analysis revealed a balanced contribution of maternal and paternal alleles in shaping the gene expression patterns in *B. blakeana*.

**Conclusions:**

Our study offers valuable insights into the genome architecture of hybrid *B. blakeana*, establishing a comprehensive genomic and transcriptomic resource for future functional genetics research within the *Bauhinia* genus. It also serves as a model for exploring the characteristics of hybrid species using T2T haplotype-resolved genomes, providing a novel approach to understanding genetic interactions and evolutionary mechanisms in complex genomes with high heterozygosity.

## Background

*Bauhinia × blakeana* Dunn, commonly known as the Hong Kong orchid tree, is a popular ornamental tree species admired for its striking purplish orchid-like flowers and extended blooming period. Its initial discovery traced back to a chance discovery by French horticulturalist Jean-Marie Delavay on Hong Kong Island in the 1880s, where it was later determined to be completely sterile and grown solely by vegetative propagation [1]. In 1908, due to its distinctive characteristics, it was proposed as a new species [2]. With the species name honoring the former governor of Hong Kong Sir Henry Blake, it has subsequently been made the emblem of the Hong Kong Special Administrative Region. However, the taxonomic status and precise origin of *Bauhinia blakeana* remain uncertain and curious because of its sterility. Due to this complete sterility, artificial propagation methods are conventionally required for *B. blakeana*, often involving cutting or grafting onto rootstocks of other *Bauhinia* species. Considering its limited natural occurrence and dependence on artificial cultivation, *B. blakeana* is usually regarded as a horticultural cultivar rather than a naturally existing species.

The sterility of *B. blakeana* has prompted the hypothesis that it may be an interspecific hybrid between *Bauhinia purpurea* L. and *Bauhinia variegata* L., a proposition first proposed by de Wit [3] based on the shared morphological similarities among the 3 species. The potential for hybridization between *B. purpurea* and *B. variegata* is further supported by their coexistence across much of their distribution ranges, partially overlapping flowering periods, xenogamous nature, and shared common pollinators [1]. Moreover, the consistent diploid chromosome number of 2n = 28 across all 3 species indicates that the genesis of *B. blakeana* likely involved the integration of one set of chromosomes from each parent, mirroring that of both parents and ruling out the idea of *B. blakeana* as a sterile polyploid [4]. It is worth noting that the genus *Bauhinia*, a member of Cercidoideae, 1 of the 6 subfamilies of Leguminosae, stands as the largest genus within the subfamily and appears to have arisen from an allotetraploid merger, exhibiting double the chromosome count observed in the earlier-diverging genus *Cercis* [5]. In some instances, wide hybridization is succeeded by the following genome doubling, a phenomenon that can potentially restore fertility to the initial potentially sterile wide hybrid. However, the 2n genome of *B. blakeana* is likely the reason causing irregular chromosome segregation and abnormal spindle formation during microsporogenesis, ultimately resulting in its complete sterility [6]. Previous research, encompassing morphological, karyotypic, and molecular analyses, including the utilization of ISSR markers and sequencing of key genetic regions (rbcL, atpB-rbcL intergenic spacer, ITS1), has provided evidence supporting this rare interspecific hybridization event [1, 4, 6, 7]. While these discoveries provide valuable insights, there remains a lack of definitive confirmation, especially at the genomic level. Despite the significant horticultural, cultural, and historical value of *B. blakeana*, our understanding of its biology remains limited primarily due to the absence of its genomic information.

Recent advancements in genome sequencing technologies, along with innovative bioinformatic approaches, have revolutionized our capacity to generate high-quality genomes for various plant species, including those with high levels of heterozygosity [8–10]. The availability of these high-quality genomes serves as a foundation for understanding the origin and evolutionary history of plants, as well as unraveling the genetic mechanisms governing essential traits. Additionally, novel methodologies such as high-throughput/resolution chromosome conformation capture (Hi-C) and assembly algorithms like trio-binning have emerged as powerful tools, enabling the construction of haplotype-resolved genomes [11–13]. The historical and cultural interest of Hong Kong *Bauhinia* led to a community-crowdfunded genome project to try to answer some of the questions on the species’ origin [14], but it only raised enough money to sequence the transcriptomes of the 3 key species [15]. Telomere-to-telomere (T2T)–level assembly completeness and haplotype-level resolution offer significant advantages in identifying genetic variations, particularly in the study of hybrid heterosis. It allows precise tracking and analysis of genetic variations across parental lines and their hybrid offspring, thereby facilitating a comprehensive understanding of the underlying genetic mechanisms.

Heterosis, also known as hybrid vigor, refers to the phenomenon in which hybrid offspring display enhanced or superior traits compared to their parents. When comparing the flower phenotype of *B. blakeana* to its putative parental species, *B. purpurea* and *B. variegata*, distinct characteristics such as more vibrant flower color, larger flower size, and an extended flowering period are observed, suggesting the presence of heterosis. Heterosis has been extensively studied and utilized in crop breeding [16–18]. However, the genetic basis of this phenomenon remains incompletely understood. Classical hypotheses, including dominance complementation, overdominance, and epistasis, have been proposed to explain the genetic mechanisms underlying heterosis [17–19]. Transcriptome profiling is commonly employed to investigate heterosis at the transcriptional level, as gene expression plays a pivotal role in linking DNA sequence variation to resulting phenotypic diversity. Several modes of gene expression differences between parents and hybrids have been suggested as contributing factors to heterosis, including additivity/nonadditivity, high-/low-parent dominance, and over-/underdominance [20]. Gene expression is a complex process regulated by a combination of genetic and epigenetic variations, involving the interplay of various genomic elements, including *cis*-acting elements, *trans*-acting factors, their intricate interactions, and other epigenomic factors [21, 22]. Moreover, allele-specific expression (ASE) introduces an additional layer of complexity to the genetic underpinnings of heterosis by elucidating the differential mRNA abundance (gene expression imbalance) between alleles in diploid (or higher-ploidy) organisms [23, 24].

In this study, we presented chromosome-level genome assemblies for the 3 *Bauhinia* species and employed a trio-binning strategy to reconstruct the high-quality haplotypes of the hybrid *B. blakeana* with gapless T2T completeness. The adoption of T2T genomes has significantly advanced genomics research by providing a detailed depiction of each chromosome from end to end, known as “telomere to telomere.” It enhances our ability to characterize genomic structure and variations, particularly in regions rich in repetitive sequences, providing insights into mechanisms and genomic evolution while elucidating the genetic underpinnings of specific traits. Leveraging our haplotype-resolved T2T genome, through an integrated approach encompassing comparative genomics, transcriptomics, and ASE analyses, we have gained valuable insights into the evolutionary dynamics of *Bauhinia* species and shed light on the genetic basis underlying the intriguing biology of *B. blakeana*. Our haplotype-resolved T2T genome serves as a valuable reference for studying genomes with high heterozygosity, particularly in analyzing the traits of hybrid genomes. It also provides a clear roadmap for future studies, facilitating key discoveries of biosynthetic genes essential for synthetic biology applications.

## Results

### Sequencing and assembly of the 3 *Bauhinia* genomes

We employed a multiplatform sequencing strategy, combining single-tube long fragment read (stLFR), BGI-SEQ short read (whole-genome sequencing, WGS), Oxford Nanopore Technologies (ONT) long read, and Hi-C sequencing methods to obtain high-quality genome assemblies for the 3 *Bauhinia* species (Supplementary Table S1). We first conducted *k*-mer analyses [25] for all 3 species to survey their overall genome characteristics. The genome size of *B. purpurea, B. variegata*, and *B. blakeana* was estimated to be ∼303.68 Mb, ∼314.49 Mb, and ∼290.97 Mb, with a heterozygosity ratio of 0.60%, 0.24%, and 4.64%, respectively (Supplementary Fig. S1).

**Figure 1:**
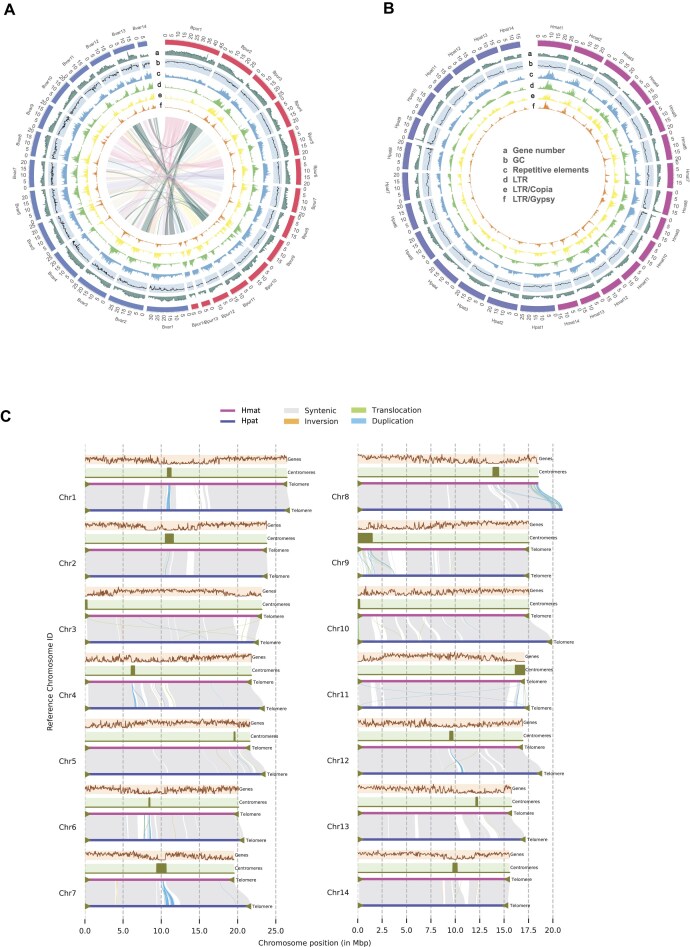
Genome assemblies of the *Bauhinia* species. (A) Circos plot of *B. purpurea* (Bpur1–Bpur14) and *B. variegata* (Bvar1–Bvar14) genome assemblies. Outer tracks depict pseudochromosomes with annotation tracks (from outer to inner): (a) gene density, (b) GC content, (c) repeat element density, (d) LTR retrotransposon density, (e) LTR/Copia subclass density, and (f) LTR/Gypsy subclass density. Synteny blocks between species are visualized by internal links. (B) Haplotype-resolved Circos plot of *B. blakeana*. Maternal (Hmat1–Hmat14) and paternal (Hpat1–Hpat14) haplotypes are annotated with gene density and repetitive element distribution. (C) Structural variations (SVs) between *B. blakeana* haplotypes. SVs (inversions, translocations, and duplications) were identified using SyRI, with the maternal haplotype (Hmat) as the reference. Additional annotations include gene density, centromere regions, and telomere positions.

We performed assembly of the stLFR reads using the Supernova assembler [26] to generate draft genome assemblies for the parental species *B. purpurea* and *B. variegata*. This process yielded 2 assemblies with genome sizes of approximately 285.15 Mb and 311.01 Mb, respectively, closely matching their estimated genome sizes (Table 1). Subsequently, we utilized Hi-C data to anchor the 2 initial assemblies onto 14 pseudochromosomes, achieving high anchor rates of 99.98% for both parental species. The resulting assemblies exhibited scaffold N50 values of 21.60 Mb for *B. purpurea* and 24.40 Mb for *B. variegata* (Table 1; Fig. 1A; Supplementary Fig. S2). The completeness of the assemblies was assessed using 1614 conserved embryophyte proteins from the BUSCO [27]. The analysis revealed a high level of completeness, with 97.8% for *B. purpurea* and 98.4% for *B. variegata*, respectively. To evaluate the quality of the genome assemblies, we calculated the mapping rate and sequencing coverage using WGS data. The mapping rate was 95.72% for *B. purpurea* and 99.29% for *B. variegata*, with coverage rates of 94.88% and 94.17%, respectively (Supplementary Table S2). These high values provide strong evidence of consistency between the assemblies and the WGS short reads, confirming the high accuracy of our assemblies.

**Table 1:** Statistics for genome assembly and annotation of 3 *Bauhinia* species

Species	*Bauhinia blakeana* Hmat	*Bauhinia blakeana* Hpat	*Bauhinia purpurea*	*Bauhinia variegata*
**Assembly feature**				
Estimated genome size	290,967,258	290,967,258	303,677,508	314,486,060
Assembled genome size	275,484,977	290,698,387	285,147,376	311,011,643
GC content	34.05%	34.22%	33.88%	34.04%
N50 of contigs (bp)	19,540,838	20,987,561	161,057	109,234
N50 of scaffold (bp)	19,540,838	20,987,561	1,475,774	2,613,106
Complete BUSCOs	C:99.0% [S:81.5%, D:17.5%], F:0.6%, M:0.4%	C:99.2% [S:78.6%, D:20.6%], F:0.7%, M:0.1%	C:97.8% [S:77.6%, D:20.2%], F:1.4%, M:0.8%	C:98.4% [S:77.0%, D:21.4%], F:1.2%, M:0.4%
**Hi-C**				
Anchor size	/	/	285,099,865	310,940,945
Anchor rate	/	/	99.98%	99.98%
Number of pseudochromosomes	14	14	14	14
N50 of scaffold (bp)	19,540,838	19,540,838	21,596,737	24,404,849
**Characteristics of protein-coding genes**				
Total number of protein-coding genes	37,804	37,956	38,735	40,111
Mean gene size (bp)	2,615.06	2,619.36	2,602.43	2,595.09
Mean CDS length (bp)	1,120.11	1,179.70	1,192.31	1,187.11
Mean exon number per gene	5.36	5.16	5.13	5.08
Mean exon length (bp)	208.89	228.60	232.52	233.74
Mean intron length (bp)	342.71	346.02	341.63	345.20
Complete BUSCOs	C:94.2% [S:78.4%, D:15.8%], F:4.0%, M:1.8%	C:96.4% [S:79.2%, D:17.2%], F:2.4%, M:1.2%	C:97.4% [S:78.4%, D:19.0%], F:1.4%, M:1.2%	C:97.8% [S:77.0%, D:20.8%], F:1.4%, M:0.8%
**Functional annotation by searching public databases**				
% of proteins with hits in NCBI nr database	97.25%	98.16%	97.80%	95.80%
% of proteins with hits in Swiss-Prot database	75.86%	78.54%	80.60%	79.54%
% of proteins with hits in KEGG database	70.12%	72.58%	74.49%	50.44%
% of proteins with hits in KOG database	70.77%	73.24%	75.01%	74.67%
% of proteins with hits in TrEMBL database	92.98%	94.23%	96.62%	96.30%
% of proteins with hits in Interpro database	93.68%	94.95%	96.83%	96.89%
% of proteins with functional annotation (combined)	99.98%	99.97%	99.98%	99.96%

To overcome the challenges posed by the high heterozygosity of the *B. blakeana* genome, we further generated ∼17.66 Gb ONT long reads for assembly. Employing a trio-binning approach, we categorized all sequencing reads into 3 groups: paternal reads, maternal reads, and ambiguous reads. Subsequently, we applied hypo-assembler [28] in haploid mode to assemble each haplotype, with paternal and ambiguous reads, and with maternal and ambiguous reads, respectively. The resulting 2 sets of high-quality, gap-free haplotypes, hereafter referred to as Hmat and Hpat, represent the maternal and paternal haplotypes of the allodiploid *B. blakeana* genome. Hmat exhibits a genome size of ∼275.48 Mb, with a contig N50 value of 19.54 Mb, while Hpat has a size of ∼290.70 Mb, with a contig N50 value of 20.99 Mb (Fig. 1B; Table 1). We used Merqury [29] to evaluate the phasing quality of the 2 *B. blakeana* haplotypes by comparing *k*-mers from parental read sets to the *k*-mers in each of the haplotype-resolved assemblies. We estimated quality value (QV) scores of 40.46 for Hmat, 45.64 for Hpat, and 42.39 for the combined set of sequences (Supplementary Table S3A). We counted the number of expected haplotype-specific *k*-mers (hap-mers) present in the corresponding haplotype assemblies and found that the maternal and paternal haplotypes recovered 92.21% and 95.82% of the expected hap-mers, respectively (Supplementary Table S3B). The maternal haplotype Hmat contains 1.74% paternal hap-mers, while the paternal haplotype Hpat contains 1.03% maternal hap-mers. These discrepancies likely arose from switch errors or base-pair errors. The *k*-mers completeness for Hmat, Hpat, and the combined diploid assembly were estimated to be 58.57%, 60.28%, and 93.67%, respectively. The result indicates that approximately 40% of the *k*-mers were haplotype-specific, highlighting the high heterozygosity in *B. blakeana* (Supplementary Table S3C). The haplotype evaluation results align with a haplotype-resolved genome assembly, indicating a satisfactory resolution of both haplotypes within the *B. blakeana* genome assembly. We further evaluated the completeness of Hmat and Hpat using BUSCO, resulting in a high completeness score of 99.0% for Hmat and 99.2% for Hpat.

### Genome annotation reveals repeat and gene landscapes

Utilizing our 4 high-quality assemblies, we conducted annotations of repetitive elements and protein-coding genes to examine the repeat and gene landscape of the 3 *Bauhinia* species. Our analysis revealed varying percentages of repetitive elements in each assembly. Specifically, we found that Hpat contains 32.21% repetitive elements, followed by *B. purpurea* with 27.92%, *B. variegata* with 27.38%, and Hmat with 25.32% (Supplementary Table S4). Among these repetitive elements, LTR retrotransposons were the most prevalent in all 4 assemblies (Fig. 1A, B).

We identified 37,804, 37,956, 38,735, and 40,111 protein-coding genes in Hmat, Hpat, *B. purpurea*, and *B. variegata*, respectively. Notably, a high percentage of these genes, 99.98%, 99.97%, 99.98%, and 99.96%, could be functionally annotated against at least 1 of the 6 databases searched—namely, Nr, SwissProt [30], KEGG [31], KOG [32], TrEMBL [30], and InterPro [33] (Table 1). Moreover, we found that the gene number, gene length, coding sequence (CDS) length, exon number, exon length, and intron length showed comparable characteristics across the 4 assemblies. The predicted gene sets for Hmat, Hpat, *B. purpurea*, and *B. variegata* were evaluated using BUSCO, yielding respective scores of 94.2%, 96.4%, 97.4%, and 97.8%. These results indicate a high level of functional completeness in the annotated proteomes, accurately representing the corresponding genomes.

We further predicted noncoding RNAs (ncRNAs), including microRNA (miRNA), transfer RNA (tRNA), ribosomal RNA (rRNA), and small nuclear RNA (snRNA) (Supplementary Table S5), as well as transcription factors (TFs), transcription regulators (TRs), and protein kinases (PTKs) in these assemblies (Supplementary Table S6). This comprehensive analysis provides valuable insights into the repetitive elements, protein-coding genes, and regulatory elements present in the genomes of these 3 *Bauhinia* species.

### Structural variations in *B. blakeana* haplotype chromosomes

Structural variation (SV) encompasses a diverse range of genomic alterations, including inversions, translocations, and duplications, which can significantly impact the organization and structure of the genome. In our study, we investigated SVs in the haplotype chromosomes of *B. blakeana*, which might be associated with its high genome heterozygosity and observed sterility. By utilizing repeat and gene annotations from previous analyses in conjunction with the quarTeT prediction software [34], we identified putative centromeres for each of the 14 pseudochromosomes in both Hmat and Hpat. The centromeres exhibited variable lengths, ranging from 101.60 Kb to 1.48 Mb in Hmat and from 143.29 Kb to 2.79 Mb in Hpat (Fig. 1C; Supplementary Table S7). Additionally, we searched for the presence of the telomere repeat motif “TTTAGGG” along each of the haplotype assembly chromosomes. This allowed us to identify 27 potential telomeric regions in Hmat, with motif repeat numbers ranging from 12 to 848, as well as 27 potential telomeric regions in Hpat, with motif repeats ranging from 31 to 1,117 (Fig. 1C; Supplementary Table S8). Notably, except for chromosome 8 in both the Hmat and Hpat assemblies, each chromosome displayed telomeres at both ends, indicating complete reconstruction to a gapless and T2T level. Using the SyRI tool [35], we detected a total of 424 SVs between Hmat and Hpat, including 12 inversions (totaling 180.38 Kb), 30 translocations (totaling 655.61 Kb), and 382 duplications (totaling 3.81 Mb) (Fig. 1C; Supplementary Table S9). The relatively low number of observed SVs could be attributed to factors such as high synteny between the 2 parental species of *B. blakeana* or limitations in SV detection methods.

### Comparative genomics reveals evolutionary dynamics in *Bauhinia*

**Figure 2:**
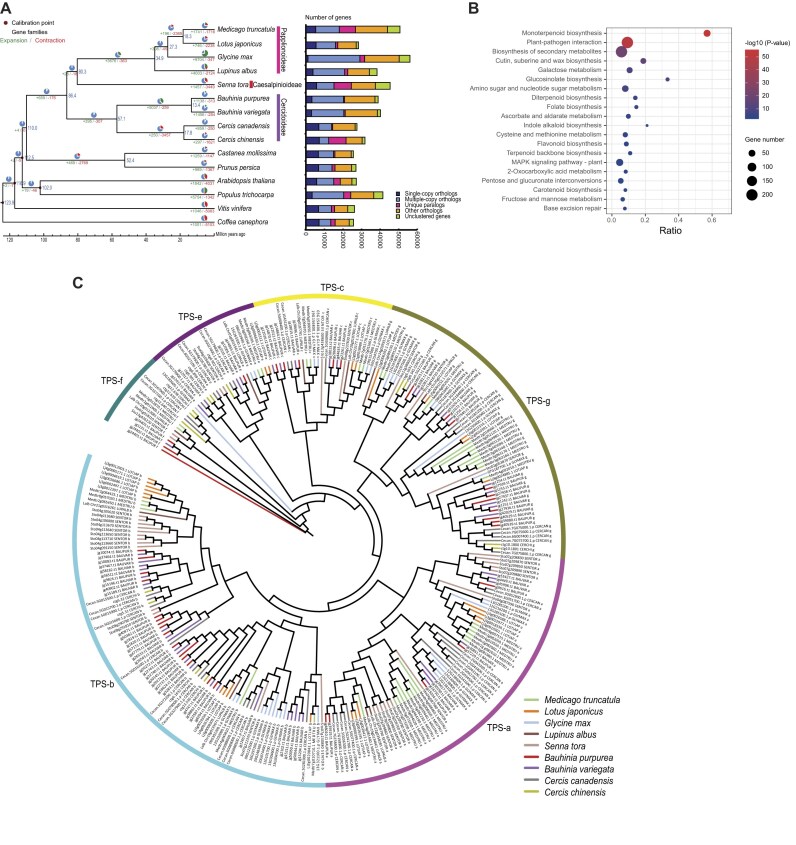
Comparative genomic analysis and terpene synthase (TPS) gene family expansion in *Bauhinia*. (A) Phylogenetic tree with divergence times of *Bauhinia* and related angiosperms. Divergence times (blue labels, million years ago) and gene family expansions (green) or contractions (red) at key evolutionary nodes are plotted. (B) KEGG enrichment of expanded gene families in *Bauhinia*. The top 20 enriched pathways (adjusted *P* < 0.05) are plotted. Terpenoid backbone biosynthesis (ko00900) and related metabolic pathways are annotated. (C) Phylogenetic classification of TPSs in *Bauhinia*. Phylogenetic tree of TPS proteins showing 6 major subfamilies (TPS-a, b, c, e, f, g).

To investigate the relationships and evolutionary history of *Bauhinia* species, we performed comparative genomic analyses involving the *Bauhinia* genomes (*B. purpurea* and *B. variegata*) and 13 other selected representative plant species. The selected species included 9 Fabaceae species from different subfamilies (*Senna tora, Lupinus albus, Glycine max, Lotus japonicus, Medicago truncatula, B. purpurea, B. variegata, Cercis canadensis, Cercis chinensis*) and 6 other eudicot species (*Vitis vinifera, Castanea mollissima, Prunus persica, Populus trichocarpa, Arabidopsis thaliana, Coffea canephora*) (Supplementary Table S10). To minimize potential impacts on the results, the sterile hybrid *B. blakeana* was excluded from the evolutionary analyses. Through gene clustering analysis, we identified 17,904 gene families in *B. purpurea* and 18,095 gene families in *B. variegata*. Across all 15 species, we identified 213 single-copy gene families shared among them. Subsequently, a maximum likelihood phylogenetic tree was constructed by combining all the genes within these single-copy gene families (Fig. 2A; Supplementary Table S11). The topology of the generated phylogenetic tree was consistent with previous research findings [5]. Molecular dating analysis estimated the divergence of the *Bauhinia* genus from the common ancestor with Cercis to have occurred approximately 57.1 million years ago (Mya), followed by the divergence of *B. purpurea* and *B. variegata* around 13.4 Mya.

We used the birth-and-death model to identify expanded and contracted gene families within the selected plant species by comparing them to gene families in their most recent common ancestor (MRCA). *B. purpurea* exhibited 1,138 gene family expansions and 313 gene family contractions, while *B. variegata* had 1,456 expanded and 284 contracted gene families. Examining the entire *Bauhinia* genus, we identified 5,037 expanded gene families and 259 contracted gene families compared to their MRCA (Fig. 2A). These 5,037 expanded gene families showed significant enrichment in the KEGG biosynthesis pathways related to bioactive compounds, including monoterpenoid, diterpenoid, flavonoid, terpenoid backbone, and carotenoid (Fig. 2B; Supplementary Table S12A). Notably, similar enrichment patterns were also observed in the expanded gene families of *B. purpurea* and *B. variegata*. Specifically, in *B. purpurea*, expanded gene families were enriched in KEGG terms such as “plant–pathogen interaction,” “isoflavonoid biosynthesis,” “flavone and flavonol biosynthesis,” and “monoterpenoid biosynthesis.” On the other hand, in *B. variegata*, expanded gene families were enriched in terms such as “phenylpropanoid biosynthesis,” “flavonoid biosynthesis,” and “sesquiterpenoid and triterpenoid biosynthesis” (Supplementary Table S12B, C).

### Evolution and expansion of terpene synthase genes in *Bauhinia*

Terpenes and terpenoids encompass a large and diverse group of natural compounds with multiple functions in plants. Terpene synthases (TPSs) are key enzymes responsible for the biosynthesis of terpenoids. These TPS proteins play crucial roles in plant growth, development, and enhancing resistance to abiotic and biotic stress [36]. To deepen our understanding of terpenoid biosynthesis in *Bauhinia* species, we identified candidate TPSs in the *Bauhinia* species and other selected plants from the Fabaceae family that were used in our previous comparative genomic analyses. We identified 39 TPS genes in *B. purpurea*, fewer than the 47 TPS genes found in *B. variegata* (Supplementary Table S13A). Within the Fabaceae family, *B. variegata* exhibits the highest TPS gene count, followed by *C. canadensis* (46), *M. truncatula* (41), and *B. purpurea* (39). Subsequently, a phylogenetic tree containing a total of 288 TPS genes across all Fabaceae species was constructed. These TPS genes were categorized into 6 clades, denoted as TPS-a, b, c, e, f, and g, according to the established subfamily classification of TPS genes (Fig. 2C) [37]. The TPS-a, b, and g collectively comprise the majority of these identified TPS genes. Within the *Bauhinia* species, TPS-b genes emerge as the most prevalent among the TPS genes, with 21 identified in *B. variegata* and 15 in *B. purpurea*, surpassing the TPS-b gene counts observed in all other species within the Fabaceae family. Following TPS-b, TPS-g genes exhibit the second-highest representation in the *Bauhinia* species, with 10 in *B. purpurea* and 9 in *B. variegata*, while TPS-a genes follow with 5 in *B. purpurea* and 7 in *B. variegata*. Notably, TPS-a, TPS-b, and TPS-g constitute clades specific to angiosperms, with TPS-a primarily containing sesquiterpene and diterpene synthases, while TPS-b and TPS-g clades typically encode monoterpene synthases.

To investigate the origin of the increased TPS gene count in the *Bauhinia* species in comparison to other species within the Fabaceae family, we analyzed the duplication events of TPS genes. The results showed that transpositional duplication was the primary driver contributing to the expansion of TPS-b in *B. purpurea* (7, 46.67%), while proximal repeats (7, 36.84%) and tandem duplication (6, 31.58%) were the major contributors to the expansion of TPS-b genes in *B. variegata* (Supplementary Table S13B). These expanded TPS-b genes are likely to contribute to the biosynthesis of monoterpenes, consequently enhancing the antimicrobial activity within these *Bauhinia* species.

### Genomic and phylogenetic insights into the maternal parentage and hybrid origin of *B. blakeana*

**Figure 3:**
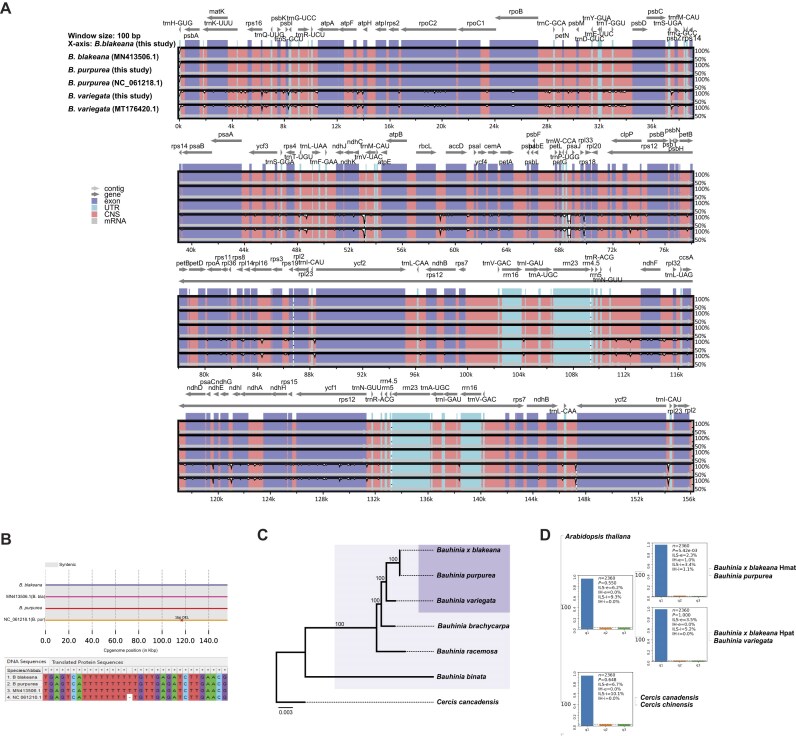
Genomic evidence for the maternal parentage and hybrid origin of *B. blakeana*. (A) Comparative analysis of 6 *Bauhinia* chloroplast (cp) genomes using mVISTA, with the assembled *B. blakeana* cp genome from this study as the reference. The y-axis represents percent identity (50%–100%). Gray arrows indicate transcriptional orientation. (B) Identification of a 1-bp deletion at position 116,948 in the published *B. purpurea* cp genome (NC061218). (C) Phylogenetic tree of the *Bauhinia* genus based on available cp genomes, with *C. canadensis* as the outgroup. (D) Phylogenetic tree of *B. blakeana* haplotypes (Hmat, Hpat) and parental species (*B. purpurea, B. variegata*), inferred from 2,360 single-copy nuclear genes using the ASTRAL method. ILS/IH indices are calculated and shown.

The parthenogenetic inheritance and low substitution rate of the chloroplast (cp) genome make it a valuable tool for phylogenetic analysis and determining hybrid parentage. Using our short-read sequencing data, we successfully assembled and annotated the cp genomes of 3 *Bauhinia* species. The complete sequences obtained were 156,100 bp for both *B. blakeana* and *B. purpurea*, and 155,415 bp for *B. variegata*. To ensure the accuracy of our assemblies, we performed comparative analyses using ClustalW alignment [38] and mVISTA software [39] to compare our assembled sequences with the previously published cp genomes of *B. blakeana* (MN413506), *B. purpurea* (NC061218), and *B. variegata* (MT176420) (Fig. 3A). Our assembled *B. blakeana* and *B. variegata* cp genomes matched published references, whereas the published *B. purpurea* genome contained a 1-bp deletion (Fig. 3B). Importantly, our assembled versions of the 3 *Bauhinia* cp genomes demonstrated identical sequences for both *B. blakeana* and *B. purpurea*, providing strong evidence supporting *B. purpurea* as the maternal parent of *B. blakeana*.

We used a maximum likelihood (ML) model to construct a phylogenetic tree to further explore the genetic relationships among the sequenced *Bauhinia* species. The tree included 3 additional *Bauhinia* species available in the NCBI database, with *C. canadensis* serving as the outgroup. The resulting phylogenetic structure was consistent with previous research, confirming a close genetic relationship among *B. blakeana, B. purpurea*, and *B. variegata* (Fig. 3C) [40].

To investigate the hybrid origin of *B. blakeana* more comprehensively, we expanded our study by incorporating a broader genomic perspective. Initially, a phylogenetic tree was constructed, integrating the 2 *B. blakeana* haplotypes alongside *B. purpurea, B. variegata, C. canadensis, C. chinensis*, and *A. thaliana* as an outgroup, which was inferred from 2,360 single-copy orthologous gene trees using the ASTRAL method. Subsequently, we utilized Phytop to assess the heterogeneity within the species tree by quantifying incomplete lineage sorting (ILS) and introgression/hybridization (IH) [41]. ILS and IH are crucial concepts in evolutionary biology that play a significant role in understanding genetic relationships and divergence patterns among species. Our results showed that *B. purpurea* and the maternal haplotype of *B. blakeana* (Hmat) formed a strongly supported monophyletic group, with ILS-e = 2.3%, ILS-i = 3.4%, IH-e = 1.0%, and IH-i = 1.1% (Fig. 3D). Additionally, *B. variegata* and the paternal haplotype of *B. blakeana* (Hpat) formed another monophyletic clade, with ILS-e = 3.5%, ILS-i = 5.2%, and no detectable introgression signals (IH-e = 0.0%, IH-i = 0.0%). Notably, the ILS/IH indices for both clades were relatively low, suggesting a limited phylogenetic conflict between the parental species and their respective *B. blakeana* haplotypes. The absence of significant IH signals at the *Bauhinia* genus node further supports the hypothesis that *B. blakeana* more likely originated from a rare single, recent hybridization event rather than recurrent gene flow. The low ILS/IH indices, combined with the clear phylogenetic clustering of *B. blakeana* haplotypes with their parental species, provide genomic evidence of its hybrid origin and highlight the utility of phased haplotypes in resolving complex evolutionary histories.

### Transcriptomic profiling reveals flower tissue gene expression patterns

**Figure 4:**
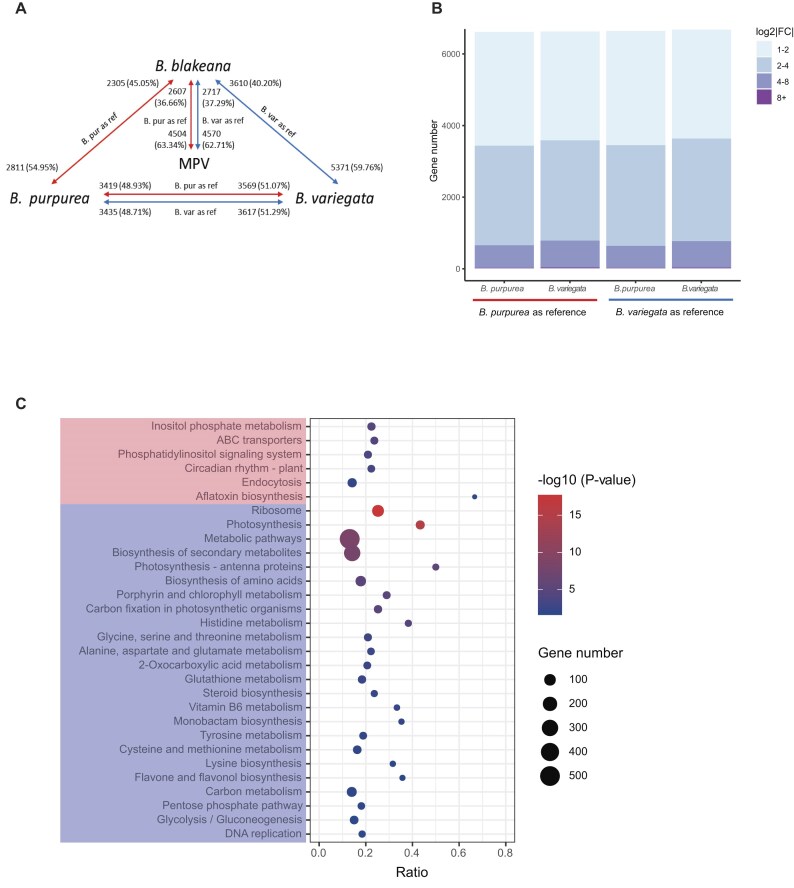
Comparative transcriptomics analyses. (A) Global DEG landscape across *Bauhinia* species comparisons. Pairwise comparisons include parental species (*B. purpurea* vs. *B. variegat*a), hybrids (*B. blakeana* vs. each parent), and mid-parent value (MPV) deviation (*B. blakeana* vs. MPV). Arrows represent the comparisons, with the numbers and proportions of upregulated DEGs indicated at the arrow ends. Reference genome impacts are color-coded (*B. purpurea*: red; *B. variegata*: blue). (B) Reference genome bias in DEG detection between parental species. Box plots compare log2|FC| distributions (binned: 1–2, 2–4, 4–8, >8) using alternate references. Both references yield comparable total DEGs. (C) Functional divergence of species-specific upregulated genes. KEGG enrichment of *B. purpurea*–upregulated genes (pink) and *B. variegata*–upregulated genes (blue).

To understand the gene expression dynamics among the 3 *Bauhinia* species, we conducted a comprehensive analysis of differential gene expressions in flower tissues. Various differential gene expression (DGE) analyses were performed, including comparisons between the parental species, comparisons between *B. blakeana* and each of the parental species, as well as comparisons between *B. blakeana* and the mid-parent value (MPV) (Fig. 4A). To ensure the reliability of our analysis, we collected 3 biological replicates for each *Bauhinia* species and performed RNA sequencing, generating a substantial amount of sequencing data for each sample (Supplementary Table S1). Initially, the reads of each sample were aligned to the *B. purpurea* genome to generate a read count matrix, which was then used for principal component analysis (PCA). Upon analyzing the PCA results, we observed that 1 sample, VAR3, exhibited an abnormal location in the PCA plot (Supplementary Fig. S3). To maintain the integrity of the analysis and ensure that this outlier did not influence our results, we excluded the VAR3 sample from subsequent analyses.

To assess preexisting differential gene expressions, we first identified differentially expressed genes (DEGs) between *B. purpurea* and *B. variegata*. To avoid false-negative results where the expression level is zero due to the inability to map reads to the reference genome caused by significant genetic differences between the parental genomes, we only selected genes that expressed in both species (with raw counts ≥10) for further analyses. Regardless of whether we used *B. purpurea* or *B. variegata* as the reference, a similar number of DEGs was observed (Fig. 4B). Using the *B. purpurea* genome as the reference, we identified a total of 6,988 DEGs, with 3,419 (48.93%) genes upregulated in *B. purpurea* and 3,569 (51.07%) genes upregulated in *B. variegata* (log_2_|FC (fold change) | > 2; *P* < 0.01). Similarly, when selecting *B. variegata* as the reference, we identified 7,052 DEGs, with 3,435 (48.71%) DEGs exhibiting higher expression levels in *B. purpurea* and 3,617 (51.29%) DEGs showing higher expression levels in *B. variegata* (Fig. 4A, B).

To assess the functional implications of these DEGs, we performed a KEGG enrichment analysis, and the results were highly consistent regardless of the reference species used (Supplementary Table S14). Specifically, enriched KEGG terms obtained using both reference genomes included “photosynthesis,” “ribosome,” and “carbon fixation in photosynthetic organisms,” indicating differences in energy production and metabolism between the 2 parental species. The term “circadian rhythm” was also enriched, suggesting possible differences in the regulation of growth and flowering timing between them. Additionally, separate KEGG enrichment analyses were conducted on the upregulated DEGs in *B. purpurea* and *B. variegata*, respectively. The overlapping results of enriched KEGG terms for upregulated DEGs in *B. purpurea* included “inositol phosphate metabolism,” “ABC transporters,” “phosphatidylinositol signaling system,” and “circadian rhythm—plant.” In contrast, the upregulated DEGs in *B. variegata* revealed enrichment in “ribosome,” “photosynthesis,” and various metabolic pathways (Fig. 4C).

### Transcriptome divergence between *B. blakeana* and its parental species

**Figure 5:**
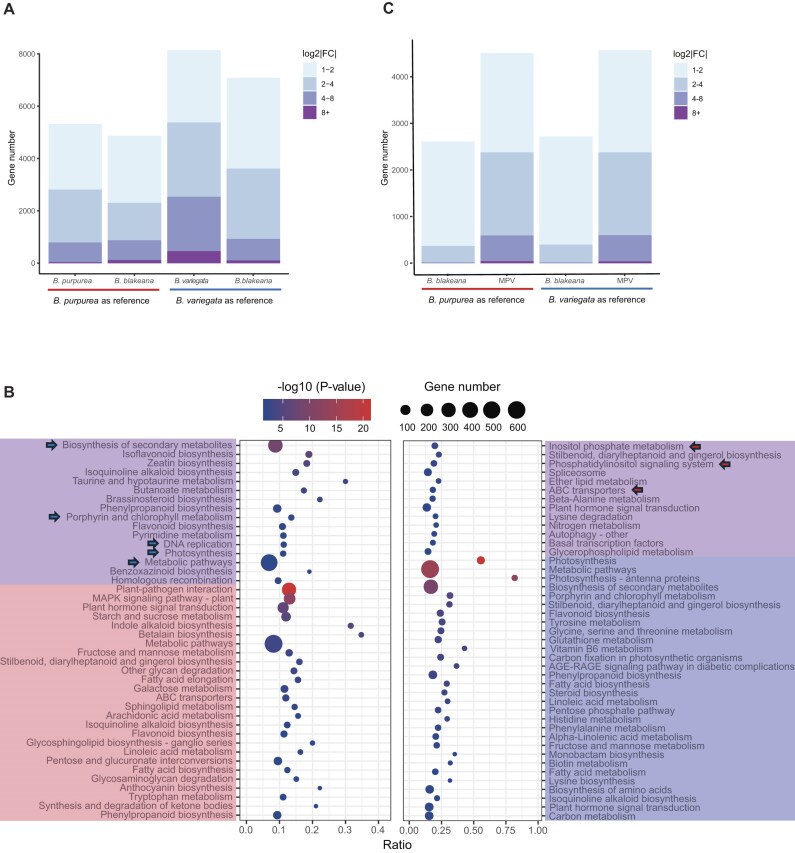
Transcriptomic divergence between *B. blakeana* and parental species. (A) Distribution of DEGs between *B. blakeana* and parental species. Box plot illustrating the number and distribution of DEGs (DE1+, log2|FC| > 1) between *B. blakeana* and *B. purpurea* (with *B. purpurea* as the reference) and between *B. blakeana* and *B. variegata* (with *B. variegata* as the reference), binned by fold-change intervals (1–2, 2–4, 4–8, >8). (B) KEGG pathway enrichment of upregulated DEGs in hybrid and parental comparisons. Enriched terms for *B. blakeana*–upregulated genes (purple), *B. purpurea*–upregulated genes (pink), and *B. variegata*–upregulated genes (blue). Arrows mark shared pathways from parental comparisons (red: *B. purpurea*; blue: *B. variegata*). (C) Distribution of DEGs between *B. blakeana* and MPV. Box plot illustrating the number and distribution of DEGs (DE1+, log2|FC| > 1) between *B. blakeana* and the MPV using *B. purpurea* (red) or *B. variegata* (blue) as references.

Considering the notable phenotypic distinctions between the parental species and *B. blakeana*, our subsequent goal was to evaluate the transcriptome divergence between them, aiming to reveal any possible association with the observed flower heterosis in *B. blakeana*. Using *B. purpurea* as the reference, we identified a total of 5,116 DEGs (log_2_|FC| > 2, *P* < 0.01) between *B. blakeana* and *B. purpurea*. Among these DEGs, 2,305 (45.05%) were upregulated in *B. blakeana*, while 2,811 (54.95%) were upregulated in *B. purpurea* (Fig. 5A). These DEGs demonstrated significant enrichment in KEGG terms such as “plant–pathogen interaction,” “plant hormone signal transduction,” and various signaling and metabolic pathways (Supplementary Table S15A). In the comparison between *B. blakeana* and *B. variegata*, we identified a larger set of DEGs, totaling 8,981 genes, using *B. variegata* as the reference. Among these DEGs, 3,610 (40.20%) exhibited upregulation in *B. blakeana*, while 5,371 (59.76%) genes exhibited upregulation in *B. variegata* (Fig. 5A). These DEGs were significantly enriched in KEGG terms, including “photosynthesis,” and “plant hormone signal transduction,” as well as various biosynthesis and metabolic pathways (Supplementary Table S15D). Notably, the number of DEGs between *B. blakeana* and *B. variegata* was approximately twice as large as the number of DEGs between *B. blakeana* and *B. purpurea*, suggesting a stronger resemblance in the gene expression profile of *B. blakeana* to its maternal parent, *B. purpurea* (Fig. 5A). Additionally, we conducted KEGG enrichment analysis on the DEGs with upregulated expression levels in each species during the comparisons separately. Interestingly, several KEGG pathways enriched in upregulated DEGs in *B. blakeana* (vs *B. purpurea*) overlapped with those enriched in upregulated DEGs in *B. variegata* (vs. *B. purpurea*). A similar pattern was observed when comparing *B. blakeana* vs. *B. variegata*, where upregulated DEGs in *B. blakeana* overlapped with pathways enriched in *B. purpurea* (vs. *B. variegata*) (Fig. 5B; Supplementary Table S14B, E; Supplementary Table S15C, F). This functional convergence suggests that *B. blakeana* retains expression patterns resembling the higher-expressing parent in these pathways.

To further investigate the gene expression patterns in *B. blakeana*, an additivity analysis was conducted to determine whether they followed an additive model, where the gene expression levels were not significantly different from the average level of parental gene expression, known as the MPV [20]. *B. purpurea* and *B. variegata* were used as references, comparing *B. blakeana* to the MPV, resulting in the identification of 7,111 and 7,287 DEGs, respectively (log_2_|FC| > 1; *P* < 0.01) (Fig. 5C). These MPV-hybrid DEGs were defined as genes with nonadditive expression patterns, attributed to allelic interactions that alter regulatory networks and consequently result in gene activity patterns distinct from the average parental values [42]. KEGG enrichment analysis of these MPV-hybrid DEGs revealed their involvement in energy conversion, utilization, and metabolic transformations. Specifically, pathways such as “photosynthesis,” “carbon fixation in photosynthetic organisms,” “carbon metabolism,” and various metabolic and biosynthetic pathways were significantly enriched (Supplementary Table S16A, D). Among these MPV-hybrid DEGs, 2,607 and 2,717 exhibited upregulation, while 4,504 and 4,570 showed downregulation in *B. blakeana* when using　*B. purpurea* and *B. variegata* as references, respectively. The upregulated DEGs in *B. blakeana* were enriched in pathways critical for cellular maintenance, biosynthesis, and stress responses, including “autophagy,” “ribosome biogenesis in eukaryotes” and “arginine and proline metabolism.” In contrast, downregulated DEGs were predominantly associated with primary metabolism and energy production, including “photosynthesis,” “biosynthesis of secondary metabolites” and “carbon fixation in photosynthetic organisms” (Supplementary Table S16B, C, E, F). Importantly, a substantial proportion of genes (67.52%: 14,780 out of a total of 21,891 expressed genes when using *B. purpurea* as a reference; 67.09%: 14,853 out of a total of 22,140 expressed genes when using *B. variegata* as a reference) in *B. blakeana* exhibited expression levels that followed an additive model, which can be explained by the combination of gene expression from its parental species. This suggests that while certain genes in *B. blakeana* exhibit nonadditive expression patterns, indicating hybrid-specific regulation, a considerable number of genes maintain an additive expression profile, reflecting the balanced contribution of both parental genomes to the gene expression in the hybrid.

### Allele-specific expression patterns in *B. blakeana*

**Figure 6:**
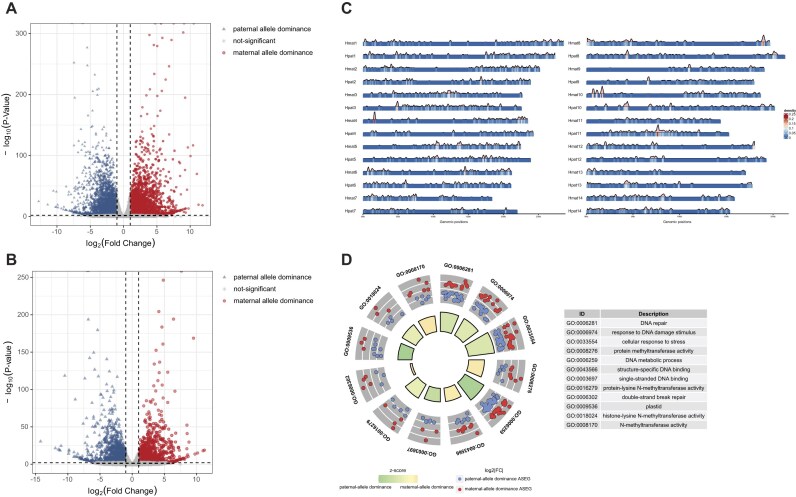
Allele-specific expression (ASE) landscape in *B. blakeana*. (A) Volcano plot of ASEGs identified by diagnostic SNP-based method using the HyLiTe pipeline. Dots represent ASEGs, with red/blue indicating maternal/paternal allele dominance. (B) Volcano plot of ASEGs identified by the haplotype-resolved method. Dots represent ASEGs with red/blue indicating maternal/paternal allele dominance. (C) Genomic distribution of ASEGs. Maternal-dominant ASEGs mapped to maternal haplotype (Hmat1–Hmat14) and paternal-dominant ASEGs to paternal haplotype (Hpat1–Hpat14). (D) Functional enrichment of ASEGs with parental allele bias. Top 12 Gene Ontology (GO) enrichment results of ASEGs in *B. blakeana*, with paternal allele dominance ASEGs (blue dots) and maternal allele dominance ASEGs (red dots) within each GO category plotted.

Expanding upon the discovery of nonadditive expression patterns observed in the genes of *B. blakeana*, our study aimed to delve deeper into the underlying molecular mechanisms by identifying genes that exhibit ASE, in which case the gene expression level differed between the 2 alleles. To accomplish this, we employed the HyLiTE (Hybrid Lineage Transcriptome Explorer) pipeline [41], which utilizes diagnostic sinmgle nucleotide polymorphisms (SNPs) to assign RNA sequencing (RNA-seq) reads to maternal or paternal alleles. Of the *B. blakeana* RNA-seq reads, 33.86% and 32.65% were unambiguously assigned to maternal and paternal alleles, respectively, with no significant bias in allelic assignment (Supplementary Table S17A). Using DESeq2 on allelic read counts, we identified 6,934 allele-specific expressed genes (ASEGs) (*P* < 0.01, |log2FC| > 1), with nearly equal proportions of maternal (3,492, 50.36%) and paternal (3,442, 49.64%) allele dominance (Fig. 6A). KEGG enrichment analysis was performed on these ASEGs, revealing significant enrichment in KEGG pathways associated with energy generation, transformation, and utilization. Enriched pathways included “photosynthesis,” “ribosome,” “carbon metabolism,” and “oxidative phosphorylation” (Supplementary Table S17B). Besides the pathways related to energy metabolism, our analysis also identified significant enrichment of KEGG pathways associated with flower color formation, including “anthocyanin biosynthesis” and “porphyrin and chlorophyll metabolism.” These pathways are known to play important roles in the synthesis and regulation of pigments responsible for flower coloration. Notably, the enrichment analysis of maternal allele dominance ASEGs revealed an enrichment of the “carotenoid biosynthesis” pathway (Supplementary Table S17C). Conversely, ASEGs showing paternal allele dominance were enriched in pathways such as “anthocyanin biosynthesis,” “flavone and flavonol biosynthesis,” and “porphyrin and chlorophyll metabolism” (Supplementary Table S17D).

However, as a portion of the *B. blakeana* RNA-seq reads (33.49%) could not be assigned using the aforementioned method, we employed a genome-wide approach to identify ASEGs. This involved identifying syntenic gene blocks between the 2 *B. blakeana* haplotypes, Hmat and Hpat, and identifying a total of 10,421 gene pairs within these blocks that exhibited a one-to-one relationship within the same orthogroups, referred to as allele pairs. By mapping the *B. blakeana* RNA-seq reads to the metagenome constructed from the combined gene sets of Hmat and Hpat, we quantified allelic read counts and employed DESeq2 to identify ASEGs on a genome-wide scale, minimizing potential errors associated with a reference-dominated approach. This approach led to the identification of 2,614 ASEGs, with 1,254 showing maternal allele dominance and 1,360 showing paternal allele dominance (log_2_|FC |> 1; *P* < 0.01) (Fig. 6B). The number of ASEGs with maternal allele dominance and paternal allele dominance was approximately equal, suggesting a balanced influence of both parental alleles and a well-maintained equilibrium in *B. blakeana*. Notably, we observed an interlaced genomic distribution of these ASEGs, with maternal and paternal dominance genes interspersed throughout the genome (Fig. 6C). Furthermore, Gene Ontology (GO) and KEGG enrichment analyses of these ASEGs provided valuable insights into their functional implications. We observed significant enrichment in several biological processes associated with maintaining genomic stability, responding to DNA damage, and ensuring proper cellular function under stress conditions. Enriched GO categories included “DNA repair,” “response to DNA damage stimulus,” “cellular response to stress,” and “double-strand break repair” (Fig. 6D; Supplementary Table S18A, B). KEGG pathway analysis further revealed enrichment in pathways such as “non-homologous end-joining,” “circadian rhythm—plant,” and “glycosylphosphatidylinositol (GPI)–anchor biosynthesis” (Supplementary Table S18C, D).

### Pigment biosynthesis in *B. blakeana*

**Figure 7:**
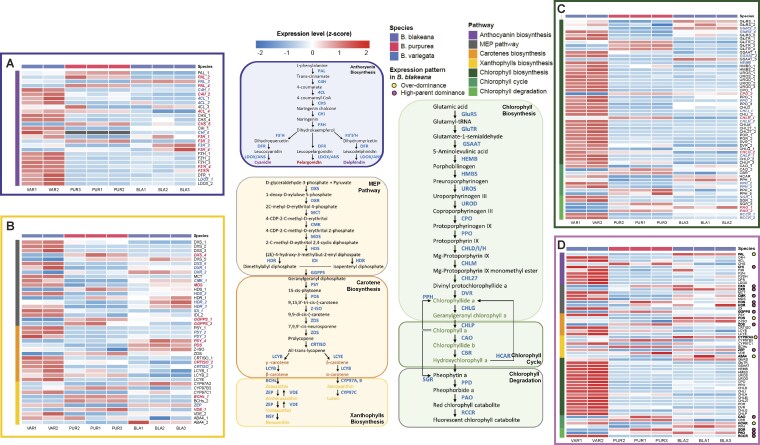
Pigment biosynthesis dynamics and allelic regulation in *B. blakeana*. The carotenoid enzymatic genes are divided into 3 groups: “MEP Pathway,” “Carotene Biosynthesis,” and “Xanthophylls Biosynthesis.” The chlorophyll enzymatic genes are divided into 3 groups: “Chlorophyll Biosynthesis,” “Chlorophyll Cycle,” and “Chlorophyll Degradation.” (A) Anthocyanin pathway expression profiles across *Bauhinia* species. Heatmap of gene expression (FPKM) for anthocyanin biosynthesis genes. ASEGs are labeled with gene IDs (maternal allele dominance: red; paternal: blue). (B) Carotenoid pathway expression profiles across *Bauhinia* species. Expression profiles of *MEP Pathway, Carotene Biosynthesis*, and *Xanthophylls Biosynthesis* modules. ASEG annotations as in (A). (C) Chlorophyll pathway expression profiles across *Bauhinia* species. Expression patterns in *Biosynthesis, Cycle*, and *Degradation* modules. ASEG labels follow (A). (D) Parental dominance and hybrid expression patterns in pigment pathways. Summed FPKM (per gene copy) heatmap. Colored dots after gene IDs indicate *B. blakeana* expression patterns: yellow (overdominance), pink (high-parent dominance).

*B. blakeana* exhibits flower heterosis, characterized by significant improvements in various traits compared to its parental species [1]. To investigate the underlying mechanisms associated with flower color formation in *Bauhinia* species, we constructed pigment metabolic pathways, specifically focusing on anthocyanins, carotenoids, and chlorophylls (Fig. 7). Initially, we identified orthologous gene groups associated with these pigment metabolic pathways in *B. purpurea, B. variegata*, and the 2 haplotypes of *B. blakeana* (Supplementary Tables S19–S21). This information also allowed us to investigate the impact of reference genome choice on gene expression analysis. Using ortholog information, we performed pairwise comparisons of expression values (FPKM: fragments per kilobase of transcript per million mapped reads) for orthologous genes within the same sample. Specifically, we compared the FPKM values obtained using 2 different reference genomes: *B. purpurea* and *B. variegata*. Our findings were consistent with our previous results, as we observed no significant differences in gene expression among all groups when directly comparing the data between groups using Welch’s *t* -test (Supplementary Table S22; Supplementary Fig. S4). However, when we performed the paired *t* -test, which considers the paired nature of the data within each group, we found no significant differences in gene expression in 6 of the total 8 samples, regardless of the reference genome chosen (Supplementary Table S23A; Supplementary Fig. S5A). To examine whether the expression value of the maternal and paternal alleles adds up to the expression value in *B. blakeana* when using *B. purpurea* or *B. variegata* as references, we obtained allelic expression counts of *B. blakeana* using the *B. blakeana* haplotype metagenome as a reference (Supplementary Table S22). Our analysis revealed no significant difference among 3 key expression values of the genes involved in pigment biosynthesis in *B. blakeana*: the sum of allelic expression level when using the haplotype metagenome as a reference, the expression level observed in *B. blakeana* when using *B. purpurea* as a reference, and the expression level observed in *B. blakeana* when using *B. variegata* as a reference (Supplementary Table S23B; Supplementary Fig. S5B). This finding indicates that the combined expression value of the maternal and paternal alleles accurately represents the overall expression level in *B. blakeana*, regardless of whether *B. purpurea* or *B. variegata* is used as the reference genome. This consistency in expression levels strengthens the reliability of our analysis and demonstrates that our gene expression assessment effectively captures the contributions of both parental alleles.

We then investigated the copy number differences of metabolism genes within these pathways across 4 *Bauhinia* assemblies. In general, most genes displayed conserved copy numbers across all 4 assemblies. However, we observed an interesting exception concerning the CHI (chalcone isomerase; EC:5.5.1.6) gene, which plays a crucial role in the anthocyanin biosynthesis pathway. Specifically, the *B. purpurea* and *B. blakeana* maternal haplotypes exhibited 3 copies of the CHI gene, while the *B. variegata* and *B. blakeana* paternal haplotype contained 4 copies of CHI (Fig. 7A; Supplementary Table S19). The presence of 1 less CHI gene in *B. purpurea* was further supported by the absence of RNA-seq expression counts for its orthologous gene in *B. purpurea* samples when using *B. variegata* as a reference (Supplementary Table S22).

Subsequently, we conducted a comparative transcriptome analysis on these pigment biosynthesis pathways in the 3 *Bauhinia* species. To facilitate this analysis, we created a new expression matrix by calculating the average FPKM from the FPKM expression data obtained in previous DEG analyses, using *B. purpurea* and *B. variegata* as references (Supplementary Table S24). With this matrix, we examined the expression-level dynamics of genes involved in pigment biosynthesis pathways in the parental species and the hybrid *B. blakeana* (Fig. 7A–C). Interestingly, we observed that the paternal species, *B. variegata*, exhibited an overall higher expression level of these genes. Next, we summed up the average FPKM values of individual gene copies belonging to the same gene, resulting in a new expression matrix. This matrix allows us to consolidate expression information and provide a representation measure of gene expression for further analysis (Supplementary Table S25). This approach allowed us to capture the overall expression level of each functional gene within the context of pigment biosynthesis pathways. We identified several genes (22.41%, 13 of 58) that exhibited clear high-parent dominance, where the expression level differed between the 2 parents but resembled the higher expressing parent in *B. blakeana*—namely, CHI, DXS, DXR, CMK, HDS, HDR, GGPPS, ZDS, ZEP, CAO, SGR, PAO, and RCCR (Fig. 7D; Supplementary Table S25). Additionally, we observed 5 genes, including PAL, PDS, CYP97A3, ABA4, and HCAR, showing overdominance expression patterns in *B. blakeana*. In these cases, *B. blakeana* exhibited higher expression levels compared to both parental species. Notably, these dominance complementation and overdominance expression patterns were particularly evident in genes involved in carotenoid biosynthesis pathways (47.83%, 11 of 23). These expression patterns of dominance complementation and overdominance likely play a role in the elevated expression levels of carotenoid biosynthesis–related genes, thus contributing to the flower color heterosis in *B. blakeana*.

### The roles of gene copy expression preference and ASEGs in flower color heterosis

Expanding upon our previous examination of gene copy expression differences among the 3 *Bauhinia* species and overall ASE patterns in *B. blakeana*, our subsequent investigation aimed to delve deeper into the role of gene copy expression preference and ASEGs in flower color heterosis. First, we examined the expression levels of individual gene copies to identify any distinct preferences in gene copy utilization within each specific gene. Our findings revealed variations in the expression levels of specific gene copies among the 3 *Bauhinia* species, indicating the presence of species-specific expression patterns. For instance, the DXR gene (1-deoxy-D-xylulose-5-phosphate reductoisomerase; EC:1.1.1.267), a key enzyme in the MEP pathway [43, 44], exhibited overdominance expression in *B. blakeana*. Between a total of 2 copies of the DXR gene, DXR_2 was consistently favored and exhibited higher expression levels in all 3 *Bauhinia* species (Supplementary Table S24). On the other hand, the PAL gene (phenylalanine ammonia-lyase; EC:4.3.1.24), a crucial enzyme involved in plant metabolism responsible for the first step in the biosynthesis of various natural products containing the phenylpropane skeleton [45], also exhibited overdominance expression in *B. blakeana*. However, this overdominance pattern was not consistently observed across all 4 PAL gene copies. Among the examined gene copies, PAL_3 consistently demonstrated the highest expression levels in both *B. blakeana* and *B. variegata*, while *B. purpurea* specifically exhibited the highest expression level in PAL_4. The observed overdominance in the PAL gene of *B. blakeana* was attributed to the elevated expression level of PAL_3 (Supplementary Table S24). Furthermore, variations in expression preferences were also observed in other genes within the pigment biosynthesis pathways, highlighting the presence of species-specific expression patterns that are likely to contribute to the character specialization observed within each *Bauhinia* species.

Motivated by the observed variations in gene copy utilization preferences and expression-level differences among the *Bauhinia* species, we further investigated the ASE patterns of each of these genes to determine whether the alleles in *B. blakeana* inherited the expression patterns corresponding to those of the parental species. To investigate ASE within pigment biosynthesis pathways, we applied a replicate-specific thresholding approach (ASE ratios >0.7 or <0.3 in ≥2 replicates) to identify ASEGs with consistent allelic bias. Specifically, we calculated the ASE ratio by dividing maternal allele expression by the sum of maternal and paternal allele expressions. Genes were classified as ASEGs if they exhibited significant ASE ratios (>0.7 maternal allele dominance or <0.3 paternal allele dominance) in at least 2 of 3 *B. blakeana* replicates. This method, while less stringent than the genome-wide DESeq2 analysis, allowed us to capture a broader profile of ASEGs with reproducible allelic imbalances. It allowed targeted exploration of allele-specific regulation in pathways critical to flower color heterosis in *B. blakeana*. Within the anthocyanin biosynthesis pathway, we identified 10 genes with maternal allele dominance and 7 genes with paternal allele dominance out of the total 27 genes analyzed (Fig. 7A–C; Supplementary Table S26). In the carotenoid biosynthesis pathway, we found 9 genes with maternal allele dominance and 12 genes with paternal allele dominance out of the total 43 genes analyzed. Similarly, within the chlorophyll biosynthesis pathway, we detected 5 genes with maternal allele dominance and 13 genes with paternal allele dominance out of the total 58 genes analyzed. The proportions of ASEGs were 62.96% in the anthocyanin pathway, 48.84% in the carotenoid pathway, and 31.03% in the chlorophyll pathway. Although the majority (85.71%, 48 of 56) of the identified ASEGs in *B. blakeana* exhibited parental allelic dominance biased toward the parent with a higher expression level, there are instances when the allelic dominance did not strictly correspond to the expression patterns of the parental species. This observation suggests that additional factors beyond the expression levels or *cis*-regulation of the parental species also play a role in regulating gene expression and establishing allelic dominance in *B. blakeana*. These factors, such as *trans*-regulatory elements, epigenetic modifications, or genetic interactions, may also contribute to shaping the observed expression patterns in *B. blakeana*.

## Discussion

In this study, we successfully generated chromosome-level genome assemblies for 2 parental species, *B. purpurea* and *B. variegata*, as well as haplotype-resolved gapless genome assemblies for the hybrid *B. blakeana*. The utilization of the trio-binning assembly strategy, taking advantage of the high heterozygosity in the *B. blakeana* genome, enabled us to overcome the challenges posed by heterozygosity and obtain high-quality genome assemblies for further analyses. The haplotype-resolved genome assemblies served as a solid foundation for our extensive downstream investigations, offering prospects for delving into the complex characteristics of the heterogeneous *B. blakeana* genome and uncovering deeper insights into its biology. Furthermore, by obtaining the cp genomes of all 3 *Bauhinia* species, we were able to confirm *B. purpurea* as the maternal parent of *B. blakeana* through comparative cp genome analyses, as well as confirming a close phylogenetic relationship between *B. blakeana* and *B. variegata*. Nuclear phylogenomics further corroborated the hybrid origin as the strong monophyly of *B. blakeana* haplotypes with their respective parents (Hmat with *B. purpurea*; Hpat with *B. variegata*).

Utilizing the high-quality genome assemblies, our subsequent comparative genomic analysis uncovered several gene families associated with terpenoid and flavonoid biosynthesis that have undergone expansions during the evolutionary diversification of the *Bauhinia* genus. Additionally, notable expansions were also observed within the TPS gene family of *Bauhinia* species when compared to other members in the Fabaceae family. Terpenes, commonly released by plants in response to insect herbivory, are primarily derived from the 5-carbon precursor, isopentenyl diphosphate (IPP). These compounds are synthesized through 2 distinct pathways within the plant cell: the mevalonate (MVA) pathway in the cytosol and the 2C-methyl erythritol 4-phosphate (MEP) pathway in plastids. The specific terpene synthase enzymes play a crucial role in determining the structure of the terpenes produced [46]. The expansion of TPS genes in *Bauhinia* species has the potential to confer heightened resistance to pathogens, establishing a more robust defense mechanism against a diverse range of microbial invaders. Terpenoids and flavonoids are major classes of secondary metabolites that exhibit a variety of pharmacological bioactivity, including antimicrobial, anti-inflammatory, antidiabetic, and anticancer effects. The genus *Bauhinia* has a long history of usage in herbal medicine for treating conditions such as malaria, diarrhea, diabetes, and various other health conditions. Specifically, *B. purpurea* and *B. variegata* have been extensively used in traditional medicine and investigated for their medicinal properties [47–50]. The expansion of TPS family genes, particularly TPS-b genes, likely contributes to the abundant terpenoid content, thereby underpinning the observed medicinal properties of these species. We also observed enrichment of the KEGG term “cutin, suberine, and wax biosynthesis” within the expanded gene families of the *Bauhinia* genus, potentially explaining the unique leaf characteristics of *B. blakeana*, characterized by cells and epicuticular wax arranged in a regular pattern, leading to its limited dust-catching capacity [51].

*B. blakeana* exhibits flower heterosis characterized by its large, showy, and vibrant magenta-colored flowers resembling orchids. Despite its sterile nature, *B. blakeana* has gained popularity as an ornamental species worldwide, mainly due to its unique floral display and extended flowering period. Therefore, our study aimed to investigate the transcriptome profiles of flower tissues and the genetic mechanisms contributing to the observed phenotypic variation among the 3 *Bauhinia* species, with a specific focus on studying the flower color heterosis in *B. blakeana*. Specifically, *B. purpurea* displayed much paler coloration compared to *B. variegata*. Through further comparing the transcriptome profiles between *B. blakeana* and its parental species, we found that even though *B. blakeana* exhibits flower color that is more similar to its paternal parent [1], the general gene expression profile of *B. blakeana* aligns more closely with its maternal parent, as evidenced by the lower number of DEGs between *B. blakeana* and *B. purpurea* (5,116 genes) compared to *B. blakeana* and *B. variegata* (8,981 genes) (Fig. 5A). We observed that the number of genes exhibiting upregulated expression in *B. blakeana* (2,607 and 2,717 genes using *B. purpurea* and *B. variegata* as references, respectively) is comparatively lower than the number of genes showing downregulated expression (4,504 and 4,570 genes) when compared to the MPV (Fig. 5C). This observation suggests a potential trade-off, wherein *B. blakeana* may have sacrificed certain functional attributes in favor of achieving heterosis-related traits such as flower color and a prolonged flowering period [52, 53].

Two main classical hypotheses aim to explain the mechanisms underlying heterosis: dominance and overdominance hypotheses [54, 55]. The dominance hypothesis focuses on the significance of dominant alleles, while the overdominance hypothesis emphasizes the advantages of heterozygosity. These 2 hypotheses are not mutually exclusive, as both mechanisms may contribute to heterosis. To investigate the genetic mechanisms underlying flower color heterosis in *B. blakeana*, we conducted analyses of gene expression patterns involved in pigment biosynthesis pathways. We found that 31.03% (18 of 58) of these genes exhibited dominance complementation or overdominance expression patterns (Fig. 7D). Notably, within the subset of genes related to carotenoid biosynthesis pathways, approximately half (47.83%, 11 of 23) displayed such nonadditive expression modes. Genes associated with carotenoid biosynthesis showed significant expression differences between the parental species, with enrichment of the carotenoid biosynthesis pathway (KEGG pathway ko00906) among DEGs with large expression disparities (log2|FC| > 4; *P* < 0.01) between *B. purpurea* and *B. variegata*. The pronounced variations in expression levels observed within the carotenoid biosynthesis pathways between the parental species may reflect underlying divergence in *cis*/*trans*-regulatory elements or epigenetic differences. Recent studies have shown that when parental species exhibit significant expression divergence for a particular gene, hybrids often exhibit an expression-level dominance-UP pattern, where promoter activity and transcriptional output in the hybrid are biased toward the parent with higher expression levels [56, 57]. This regulatory asymmetry likely arises from differential binding affinities of TFs and epigenetic modifications inherited from the parents, ultimately driving higher-than-average gene expression in the hybrid. Such mechanisms may explain the observed enrichment of nonadditive expression patterns in carotenoid biosynthesis genes in *B. blakeana*. For example, DXS (1-deoxy-D-xylulose 5-phosphate synthase; EC:2.2.1.7) and DXR (1-deoxy-D-xylulose 5-phosphate reductoisomerase; EC:1.1.1.267), which catalyze the first 2 committed steps of the MEP pathway, exhibited high-parent dominance in *B. blakeana*. Subsequent enzymes (CMK, HDS, HDR; EC:2.7.1.148, EC:1.17.7.1, EC:1.17.1.2) also showed dominance complementation, suggesting coordinated upregulation of the MEP pathway, potentially enhancing the flux of isoprenoid precursors into carotenoid biosynthesis, thus contributing to the flower color heterosis in *B. blakeana* [58, 59].

ASE is another mechanism that has been suggested to contribute to heterosis [60–62]. We employed 2 distinct approaches to conduct genome-wide analyses of ASE in flower tissues of *B. blakeana*. Although there were variations in the total number of identified ASEGs between the 2 methods, we observed a balance in both the number and level of ASEGs biased toward each parental allele within each method. Despite the limitations of both approaches used, they yielded valuable insights into the ASE landscape within the *B. blakeana* genome, highlighting the equitable participation of maternal and paternal alleles in shaping the observed ASE patterns. Through our in-depth analysis of pigment biosynthesis-related genes in *B. blakeana*, we discovered that the ASE patterns demonstrate a preference for the parental allele linked to higher expression levels in the comparison between the parental species (Fig. 7A–C). However, it is important to note that the ASE patterns do not consistently correlate with dominance complementation or overdominance expression patterns. In our analysis, genes exhibiting dominance complementation or overdominance expression patterns may display diverse ASE behaviors across their different copies. For example, within the same gene, there can be variations among gene copies: all copies of a gene may exhibit ASE bias toward the same parental allele (e.g., DXR and GGPPS), and some copies of a gene may show ASE bias toward different parental alleles (e.g., PAL and DXS), and in some cases, all copies of a gene may not display ASE at all (e.g., ZDS and ABA4). The variability observed indicates that dominance patterns and ASE are influenced by complex layers of regulation, highlighting the intricate interplay between allelic-specific expression and genetic regulatory mechanisms. Further investigation into the specific regulatory factors influencing ASE and dominance patterns, such as TFs, *cis*-regulatory elements, and epigenetic modifications, will provide a deeper understanding of how these mechanisms interact to shape gene expression and phenotypic outcomes. Resolving these complexities will elucidate the molecular mechanisms underlying heterosis and uncover how hybrid organisms acquire superior traits via synergistic interactions between parental genetic and epigenetic regulatory networks.

Overall, our study provides comprehensive genomic and transcriptomic insights into the biology of *B. blakeana*. Through the utilization of our *de novo* assembled haplotype-resolved and gapless T2T genome, we have advanced our understanding of the genomic structure and genetic mechanisms underlying the captivating flower color trait in this popular ornamental hybrid tree species, serving as a case study for investigating traits in hybrid species. Furthermore, the resources generated in this study lay the foundation for future genetic studies, breeding programs, and conservation initiatives in *Bauhinia* species.

## Methods

### Plant sampling, library preparation, and sequencing

Fresh leaves of 3 *Bauhinia* species—namely, *Bauhinia × blakeana* Dunn (NCBI: txid180222), *B. purpurea* L. (NCBI: txid3806), and *B. variegata L*. (NCBI: txid167791)—were collected from Shenzhen, Guangdong Province, China. To perform whole-genome sequencing on all 3 species, high-molecular-weight (HMW) genomic DNA was extracted using a modified cetyltrimethylammonium bromide (CTAB) method [63]. The extracted DNA from each species was used to prepare stLFR libraries [64] and WGS short-read libraries, following the respective protocols. Hi-C libraries were constructed for each species using the MboI enzyme and following the standard Hi-C library preparation protocol [65]. These libraries were subsequently sequenced on the BGISEQ500 platform to generate pair-end reads with an insert size of ∼250 bp [66]. In addition, we prepared an extra ONT library for the hybrid species *B. blakeana* using the LSK108 kit (SQK-LSK108, Oxford), which was then sequenced on the Nanopore MinION sequencer [67].

To perform transcriptome sequencing, we collected 3 fully blossomed flower tissues from each individual of the 3 sequenced *Bauhinia* species. Total RNA was isolated using the TIANGEN Kit with DNase I and processed using the NEBNextUltra RNA Library Prep Kit (New England Biolabs) to create a pair-end library with a 250-bp insert size for each sample. The libraries were then barcoded and pooled together as input for downstream sequencing on the BGI-DIPSEQ platform.

### Genome size estimation

Previous studies have shown that the 3 *Bauhinia* species share the same chromosome number (2n = 28) [4]. To estimate the genome size of each species, we performed *k*-mer analysis. First, the raw WGS short reads were filtered according to the sequencing quality with Trimmomatic (RRID:SCR_011848) (v0.40) with the “ILLUMINACLIP:adapter.fa:2:30:10 HEADCROP:5 LEADING:3 TRAILING:3 SLIDINGWINDOW:5:15 MINLEN:95” parameter [68]. Next, *k*-mer frequencies were counted by Jellyfish (RRID:SCR_005491) (v2.2.6) with a *k*-value of 21 using the clean WGS reads [69]. Based on the 21-mer frequency distribution analysis with GenomeScope (RRID:SCR_017014) [70], we estimated the genome size of *B. purpurea, B. variegata*, and *B. blakeana* to be ∼303.68 Mb, ∼314.49 Mb, and ∼290.97 Mb, respectively. Notably, the estimated genome size of *B. variegata* was close to the previously published genome size of 326.4 Mb [5].

### Genome assembly and quality control

To generate draft assemblies for the parental species *B. purpurea* and *B. variegata*, we performed *de novo* assembly using the Supernova assembler (RRID:SCR_016756) (v2.1.1) with the “–max reads 2140000000” parameter for each species using the stLFR reads [26]. Next, we used the clean WGS short-read data of each species to fill gaps in the draft assemblies using the GapCloser (RRID:SCR_015026) with default parameters. To further improve the assembly contiguity, we utilized Hi-C data from each parental species. We aligned the Hi-C data to the draft assemblies using BWA (RRID:SCR_010910)–MEM [71] and then integrated the assemblies from contig level into pseudochromosome level using ALLHiC (RRID:SCR_022750) [13]. Specifically, we used the bam files resulting from the alignment to assign contigs into a predefined number of groups (14 groups in our research), and unplaced contigs were assigned into partitioned clusters. Finally, we reordered and oriented each group to optimize the result and generate the fasta format sequences and agp location files. We evaluated the genome scaffolding by plotting the chromatin contact matrix.

To assemble the hybrid offspring *B. blakeana*, we employed a trio-binning strategy to generate 2 fully phased haplotype assemblies. First, we identified solid *k*-mers, which are *k*-mers that are unique in the 3 *Bauhinia* genomes. This yielded 3 sets of solid *k*-mers for the hybrid, paternal, and maternal sequencing data. Subsequently, we defined paternal and maternal hap-mers. Paternal hap-mers are the intersection between the paternal and hybrid solid *k*-mers, while maternal hap-mers are the intersection between the maternal and hybrid solid *k*-mers. This definition is similar to the concept of hap-mers used in Merqury but adjusted to accommodate our solid *k*-mers. We proceeded to assemble the 2 haplotypes of *B. blakeana* separately. First, we categorized all reads into 3 groups: paternal reads, maternal reads, and ambiguous reads. Reads that exclusively contained paternal hap-mers as their solid *k*-mers were classified as paternal reads, and the same principle applied to maternal reads. Reads containing both types of hap-mers or neither were labeled as ambiguous. We then ran the hypo-assembler in haploid mode for each haplotype, once with paternal and ambiguous reads and once with maternal and ambiguous reads. This approach resulted in 2 distinct yet more accurate assemblies. Following this, haplotype-specific Hi-C reads were aligned to their respective draft assemblies for scaffolding based on contact frequency. Subsequently, we manually clustered the remaining long reads from the previous steps and assembled them. This newly assembled set of contigs was used for gap-filling purposes. After completing the aforementioned steps, the majority of the genome was resolved. However, not all the telomeres are fully assembled. To address this, we identified reads displaying a high abundance of telomere signals that were not utilized in the initial assembly. Subsequently, we manually clustered these reads based on their SNPs in comparison with the existing contig terminals and then assigned the clusters to their respective terminal positions.

The genome completeness was evaluated by BUSCO (RRID:SCR_015008) using the embryophyta_odb10 database [27]. The genome continuity was evaluated by calculating contig N50 length. The accuracy of the genome was evaluated by mapping the WGS sequencing data to the genome with BWA-MEM and calculating mapping rate and coverages with SAMTOOLS (RRID:SCR_002105) [72]. To further assess the 2 haplotype genomes of *B. blakeana*, we used Merqury (RRID:SCR_022964) [29] to evaluate the haplotype-specific accuracy, completeness, and phase block continuity based on the trio information.

### Identification of repetitive elements

To identify repetitive elements in our assembled genomes, we employed a combination of homology-based and *de novo* prediction methods following the Repeat Library Construction–Advanced pipeline [73]. First, we employed RepeatMasker (RRID:SCR_012954) [74] and RepeatProteinMasker to identify transposable elements (TEs) based on similarity-based comparisons to search for known repeat sequences with Repbase (RRID:SCR_021169) [75]. In addition, we used LTR_Finder (RRID:SCR_015247) [76] to search for LTR retrotransposons *de novo*. The resulting repetitive sequence libraries were then integrated using RepeatModeler (RRID:SCR_015027) [77] to create a complete and nonredundant custom library, which served as input for RepeatMasker to identify and classify TEs in the genome assemblies. Furthermore, we searched for tandem repeats across the genomes using Tandem Repeats Finder (RRID:SCR_022193) [78]. All identified repeats were used to soft mask the genome assemblies with RepeatMasker before gene structure prediction.

### Protein-coding gene prediction and functional annotation

We utilized a combination of *ab initio*, homology-based, and RNA-seq–based approaches with the BRAKER2 (RRID:SCR_018964) pipeline [79] to predict the protein-coding gene set in our assembled genomes. To begin, we obtained and assembled the publicly available leaf transcriptome data for each species from the crowdfunded *Bauhinia* Genome project [15]. The leaf data were then aligned to the corresponding genomes using HISAT2 (RRID:SCR_015530) (v2.1.0) [80] with “–max-intronlen 500000 –sensitive –dta –dta-cufflinks –phred33 –no-discordant –no-mixed” parameters, and the resulting BAM files were sorted using SAMTOOLS. We used the BAM files, along with the OrthoDB (RRID:SCR_011980) v10.1 protein database [81] (the published *B. variegata* proteins were added), as input for BRAKER2 with “–softmasking –etpmode.” We further filtered the predicted gene sets to remove any translated proteins less than 30 amino acids in length or with in-frame stop codons. Finally, we evaluated the completeness of the gene sets using BUSCO with the embryophyta_odb10 database.

We used 2 methods to infer the functions of our predicted genes. First, we performed a BLASTP (RRID:SCR_001010) homolog search against public protein databases such as UniProtKB/Swiss-Prot (RRID:SCR_021164), TrEMBL, NCBI nonredundant (NR), and KEGG (RRID:SCR_012773). Second, we utilized InterProScan (RRID:SCR_005829) to search for conserved amino acid sequences, motifs, and domains by comparing the sequences against domain databases including Pfam (RRID:SCR_004726), PANTHER (RRID:SCR_004869), PRINTS (RRID:SCR_003412), PROSITE (RRID:SCR_003457), ProDom (RRID:SCR_006969), and SMART (RRID:SCR_005026).

### Identification of structural variations, centromeres, and telomeres

The Nucmer alignment tool from the MUMmer (RRID:SCR_018171) [82] was used for conducting whole-genome alignments. Nucmer was executed with the -maxmatch option to retrieve all alignments between the *B. blakeana* allelic chromosomes, with parameters -c 500, -b 500, and -l 100. Subsequently, the delta-filter and show-coords subprograms were employed to filter the alignments and convert them into tab-delimited files. Lastly, SyRI (RRID:SCR_023008) [35] was used to detect inversions, translocations, and duplications.

CentroMiner from the quarTeT prediction software (RRID:SCR_025258) [34] was employed for centromere identification. To enhance its performance, the repeat and gene annotations obtained from previous analyses were added as input. The resulting predictions underwent a manual selection process to ensure accuracy and reliability before finalization. TeloExplorer from quarTeT was used for telomere identification by searching for the characteristic motif (TTTAGGG).

### Identification of noncoding RNAs

In addition to protein-coding genes, we also identified ncRNAs within our assembled genomes. We used tRNAscan-SE (RRID:SCR_008637) [83] to identify tRNA genes and BLASTN (RRID:SCR_001598) to search for rRNA genes by comparing the rRNA sequences of *A. thaliana* and *Oryza sativa* against each of the 3 *Bauhinia* assemblies. We predicted miRNAs and snRNAs by searching the sequences against the Rfam (RRID:SCR_007891) database using Infernal (RRID:SCR_011809) [84].

### Identification of transcription factors

We identified and classified TFs, TRs, and PTKs among our predicted gene models into different families using the online tool iTAK pipeline with default parameters [85].

### Phylogenetic analysis and divergence time estimation

Single-copy genes from 15 selected plants were identified using OrthoFinder (RRID:SCR_017118) [86] and subsequently used to construct the phylogenetic tree, following these steps: (i) For each single-copy gene orthogroup data set, we performed multiple amino acid sequence alignments using MAFFT (RRID:SCR_011811) (v.7.310) [87], followed by gap position removal using Gblocks (RRID:SCR_015945) (v.0.91b) (positions where 50% or more of the sequences have a gap were removed) [88]. (ii) We used the ML software IQ-TREE (RRID:SCR_017254) (v.1.6.1) [89] to reconstruct the phylogenetic tree for each single-copy gene family. (iii) The gene trees of each data set were then analyzed using ASTRAL (RRID:SCR_024520) (v.5.5.9) [90] to infer the species tree with quartet scores and posterior probabilities. (iv) The sequences generated from step i were also concatenated as a single supermatrix and a concatenation tree was generated using RAxML (RRID:SCR_006086) [91].

We used the MCMCTree program in the PAML package (RRID:SCR_014932) (v4.5) [92] to estimate the divergence time of each tree node, based on the estimated divergence times of the following nodes from the TimeTree (RRID:SCR_021162): *C. canephora*–*V. vinifera* (111.4–123.9 Mya), *A. thaliana–V. vinifera* (111.24–117.56 Mya), *A. thaliana–M. truncatula* (102–112.5 Mya), and *A. thaliana–P. trichocarpa* (107–109 Mya). To perform this analysis, we used the sequential PHYLIP format nucleotide sequences and rooted phylogenetic tree derived from the result of the gene family analysis as inputs for MCMCTree.

We used CAFE (v2.1) [93] to infer the expansion and contraction of gene families based on the phylogenetic analysis and divergence time. The input tree for CAFE was the species tree constructed by ASTRAL. For each gene family that was significantly expanded or contracted (*P* < 0.05), we inferred functional information based on the functional annotation results. KEGG and GO enrichment analyses of genes were conducted using an enrichment pipeline (parameter setting: *p* Adjust Method: fdr; TestMethod: FisherChiSquare) [94, 95].

### Assembly of chloroplast genome and phylogenetic analysis of hybrid origin

The chloroplast genomes (cp) of the 3 *Bauhinia* species were assembled using the clean WGS short-read data in GetOrganelle (RRID:SCR_022963) [96] and further annotated using CpGAVAS2 [97]. We obtained additional available *Bauhinia* cp genomes from the NCBI database, including *B. binate* (NC_037764.1), *B. brachycarpa* (NC_037762.1), and *B. racemosa* (ON456405.1). *C. cancadensis* (KF856619.1) from the *Cercis* genus was also obtained to serve as an outgroup. To construct the phylogenetic tree, a total of 77 protein-coding genes were aligned and trimmed following the same pipeline used for the nuclear tree, and the phylogenetic tree was built by RAxML with “-f a -#1000 -m PROTGAMMAJTT” parameters. In addition, we obtained previously published chloroplast genomes of *B. blakeana* (MN413506.1), *B. purpurea* (NC_061218.1), and *B. variegata* (MT176420) from the NCBI database for comparison with our assembled genomes using mVISTA [39, 98].

Hybrid origin analysis based on whole-genome data followed the phylogenetic workflow described previously, with modifications to taxon sampling and data processing. Single-copy orthologs were identified from *B. purpurea, B. variegata, B. blakeana* haplotypes (Hmat/Hpat), *C. canadensis, C. chinensis*, and *A. thaliana* using OrthoFinder (RRID:SCR_017118) [86] with default parameters. From the resulting 2,360 single-copy genes, gene trees were constructed using IQ-TREE (RRID:SCR_017254) [89]. Subsequently, species tree inference was performed via ASTRAL (RRID:SCR_024520) [90] with the “-t 2” parameter. Phytop [41] was employed to quantify ILS and IH signals based on the ASTRAL species tree.

### RNA-seq data analysis and ASE gene identification

RNA-seq sequencing data were trimmed using Trimmomatic to remove low-quality bases and adapter sequences. Clean reads of all 3 species were mapped to the selected reference genome using Bowtie 2 (RRID:SCR_016368), and the counts and FPKM values were calculated by the eXpress program [99], which was incorporated in the Trinity (RRID:SCR_013048) package. DEGs were identified based on the counts using DESeq2 (RRID:SCR_015687) [100].

We employed 2 distinct methods for genome-wide identification of ASEGs in *B. blakeana*:

1. Diagnostic SNP-Based Method: Clean RNA-seq reads were processed by HyLiTE to produce tables of parental and allelic expression data in a single step. First, RNA-seq reads from *B. blakeana* and its parents were aligned to the *B. purpurea* reference genome using Bowtie2 with default parameters. Next, alignments were proceeded to SAMtools to generate the .pileup file. HyLiTE was used to detect diagnostic SNPs (positions with fixed differences between *B. purpurea* and *B. variegata*) and assign *B. blakeana* reads to parental alleles. The HyLiTE output provided maternal and paternal allele counts per gene for each *B. blakeana* sample. These counts were manually transformed into a DESeq2-compatible matrix, where each gene’s expression was represented as a 2-column matrix (maternal counts vs. paternal counts). DESeq2 was then applied to test for significant allelic imbalance.
2. Haplotype-Resolved Method: Syntenic gene blocks between *B. blakeana* maternal haplotype and paternal haplotype were identified using BLASTP and MCScanX (RRID:SCR_022067) [101] with annotations and protein sequences. Genes from the same orthogroup of the 2 haplotypes were identified using OrthoFinder. Gene pairs belonging to the same orthogroup and located in large syntenic blocks were identified as alleles. The assemblies and annotations of both haplotypes were then combined to construct a metagenome. Clean RNA-seq reads of *B. blakeana* were mapped to the metagenome using Bowtie2 by retaining the best alignment. FPKM and counts were calculated using the eXpress program. To screen for ASEGs, we employed DESeq2 using the allelic read count data.

For ASEG identification in pigment biosynthesis pathways, we applied a replicate-specific thresholding approach. This approach relaxed statistical stringency compared to the DESeq2-based methods described above to capture ASEGs with reproducible allelic biases, even if they did not meet genome-wide significance thresholds. For each gene in *B. blakeana*, we calculated the ASE ratio as


\begin{eqnarray*}
{\mathrm{ASE\,\,{Ratio}}} = \frac{{{\mathrm{Maternal\,\,{Allele}\,\,{Expression}\,\,}}\left( {{\mathrm{FPKM}}} \right)}}{{{\mathrm{Maternal}} + {\mathrm{Paternal\,\,{Allele}\,\,{Expression}\,\,}}\left( {{\mathrm{FPKM}}} \right)}}
\end{eqnarray*}

The allele expression values were derived from RNA-seq read counts mapped to the haplotype-resolved metagenome, same as the haplotype-resolved method described above. ASEG was defined as a gene with an ASE ratio >0.7 (maternal dominance) or <0.3 (paternal dominance) in ≥2 of 3 replicates.

### Identification of flower pigmentation genes

To elucidate the mechanisms underlying flower pigmentation, we focused on the metabolism and accumulation of flavonols, anthocyanins, carotenoids, and chlorophylls. Initially, we constructed the metabolic pathways associated with these compounds. For reference, we downloaded gene sequences encoding enzymes involved in these pathways from UniProt (RRID:SCR_002380). These reference sequences served as a basis for identifying corresponding genes in our assemblies. Our candidate gene selection process involved the following criteria: (i) Candidate gene sequences were identified through BLASTP searches using a cutoff *E*-value of 1e−05, comparing them to the query gene sequences we obtained. (ii) Functional annotations of the candidate genes were manually inspected to ensure similarity to the query genes. (iii) Following the initial identification, the candidate genes underwent further verification by constructing phylogenetic trees. The maximum likelihood trees were constructed using IQTREE after aligning the sequences with MAFFT.

## Editors’ Note

GigaScience Press was one of the founders of the Bauhinia Genome in 2015, a community genomics project engaging the community in the sequencing of Hong Kong’s floral emblem to promote genomics literacy and education. As an open science project also teaching reproducible science practices, we committed to make all the data, protocols, and supporting materials open and transparent, and we hope the publication of this fully complete genome and its supporting data fulfills that pledge. We thank the Bauhinia Genome community for crowdfunding the original *Bauhinia* transcriptomic data used here and their ongoing support and interest. For more, see bauhiniagenome.hk.

## Abbreviations

ASE: allele-specific expression; ASEG: allele-specific expressed genes; bp: base pair; BUSCO: Benchmarking Universal Single-Copy Orthologs; CHI: chalcone isomerase; FPKM: fragments per kilobase of transcript per million mapped reads; Hi-C: high-throughput/resolution chromosome conformation capture; KEGG: Kyoto Encyclopedia of Genes and Genomes; MPV: mid-parent value; NCBI: National Center for Biotechnology Information; ONT: Oxford Nanopore Technologies; stLFR: single-tube long fragment read; SV: structural variation; T2T: telomere-to-telomere; TPS: terpene synthases; WGS: whole-genome sequencing.

## Supplementary Material

giaf044_Supplemental_Files

giaf044_GIGA-D-24-00537_Original_Submission

giaf044_GIGA-D-24-00537_Revision_1

giaf044_Response_to_Reviewer_Comments_Original_Submission

giaf044_Reviewer_1_Report_Original_Submission Steven Cannon -- 12/23/2024

giaf044_Reviewer_2_Report_Original_Submission Yongpeng Ma -- 12/29/2024

giaf044_Reviewer_2_Report_Revision_1 Yongpeng Ma -- 2/27/2025

## Data Availability

The raw genomic sequencing data for all 3 analyzed species have been deposited in the NCBI Sequence Read Archive (SRA) under BioProject accession number PRJNA1219298. The genome assemblies, annotations, and flower tissue transcriptomic data are available at the China National GeneBank (CNGB) Sequence Archive (CNSA) under accession numbers CNP0001583 and CNP0006215. Additionally, previously published leaf transcriptome data can be accessed via NCBI BioProject PRJEB21302 and GigaDB [15]. All additional supporting data are available in the *GigaScience* repository, GigaDB [102], with individual datasets for parental species *B. purpurea* [103], *B. variegata* [104], and the hybrid species *B. blakeana* [105].

## Additional Files

**Supplementary Fig. S1**. Genome survey analysis of *Bauhinia* species. (A–C) The *k*-mer spectra (*k* = 21) for *B. purpurea, B. variegata*, and *B. blakeana* generated by GenomeScope 2.0. Peaks correspond to heterozygous (left) and homozygous (right) *k*-mer distributions. The x-axis refers to the *k*-mer coverage, and the y-axis refers to the frequency of the *k*-mer for a given coverage.

**Supplementary Fig. S2**. Hi-C scaffolding of *Bauhinia* pseudochromosomes. (A) *B. purpurea* chromatin contact matrix. (B) *B. variegata* matrix. Colors indicate contact frequency.

**Supplementary Fig. S3**. Principal component analysis (PCA) plot of RNA-seq data. PCA of transcriptomes (PC1 vs. PC2) for *B. purpurea, B. variegata*, and *B. blakeana* (*n* = 3 replicates). Outlier sample VAR3 (gray) was excluded from downstream analysis.

**Supplementary Fig. S4**. Reference genome bias in ortholog expression quantification. The figure consists of 8 violin box plots, each representing a different sample. The violin plot shows the distribution of gene expression values, with the width indicating the density of data points at different expression levels. Welch’s *t* test was employed to compare the gene expression data between groups.

**Supplementary Fig. S5**. Expression concordance between allelic and reference-based quantification. (A) Bar plot illustrating the mean difference (paired *t* test) in gene expression values for each of the *Bauhinia* samples, with consideration given to distinct reference genomes used (*B. purpurea* or *B. variegata*). (B) Bar plot illustrating the mean difference in expression values within *B. blakeana* samples when utilizing varying reference genomes. We compared the allelic expression using the *B. blakeana* haplotype metagenome with the overall expression values when using either *B. purpurea* or *B. variegata* as a reference.

**Supplementary Table S1**. Sequencing methods and their depth of coverage for *Bauhinia* species.

**Supplementary Table S2**. Assessment of genome consistency.

**Supplementary Table S3**. Phasing quality estimation of the *B. blakeana* haplotypes.

**Supplementary Table S4**. Statistics of repetitive sequences identified in the *Bauhinia* genomes.

**Supplementary Table S5**. Summary of noncoding RNA annotated in the *Bauhinia* genomes.

**Supplementary Table S6**. Statistics of transcription factors (TFs), transcription regulators (TRs), and protein kinases (PTKs) in *Bauhinia* genomes.

**Supplementary Table S7**. Summary of centromeres in *B. blakeana* haplotype assembly chromosomes.

**Supplementary Table S8**. Summary of telomeres in *B. blakeana* haplotype assembly chromosomes.

**Supplementary Table S9**. Summary of structural variations between Hmat and Hpat.

**Supplementary Table S10**. Genomic data used for comparative genomic analysis.

**Supplementary Table S11**. Summary of gene ortholog analysis conducted on 15 genomes.

**Supplementary Table S12**. KEGG enrichment of expanded gene families of selected evolutionary nodes.

**Supplementary Table S13**. TPS gene identification.

**Supplementary Table S14**. KEGG enrichment of DEGs (|log2 FC|>2, *P* < 0.01) between *B. purpurea* and *B. variegata*.

**Supplementary Table S15**. KEGG enrichment of DEGs (|log2 FC|>2, *P* < 0.01) between *B. blakeana* and its parental species.

**Supplementary Table S16**. KEGG enrichment of DEGs (|log2 FC|>1, *P* < 0.01) between *B. blakeana* and MPV.

**Supplementary Table S17**. Results of allele-specific expression analysis using the HyLiTE pipeline.

**Supplementary Table S18**. Functional enrichment of ASEGs identified using a genome-wide approach.

**Supplementary Table S19**. The number and gene ID of anthocyanin biosynthesis genes in the 4 *Bauhinia* assemblies.

**Supplementary Table S20**. The number and gene ID of carotenoid biosynthesis genes in the 4 *Bauhinia* assemblies.

**Supplementary Table S21**. The number and gene ID of chlorophyll biosynthesis genes in the 4 *Bauhinia* assemblies.

**Supplementary Table S22**. FPKM data for paired *t* test analysis of gene expression using different references.

**Supplementary Table S23**. Summary of paired *t* test analysis for gene expression using difference references.

**Supplementary Table S24**. Average FPKM expression matrix for comparative transcriptome analysis of pigment biosynthesis pathways in 3 *Bauhinia* species.

**Supplementary Table S25**. Summed expression matrix of genes involved in pigment biosynthesis pathways in 3 *Bauhinia* species.

**Supplementary Table S26**. ASE ratio results of genes involved in pigment biosynthesis pathways in *B. blakeana*.

## References

[1] Lau CP, Ramsden L, Saunders RM. Hybrid origin of “*Bauhinia blakeana*” (Leguminosae: caesalpinioideae), inferred using morphological, reproductive, and molecular data. Am J Bot. 2005;92:525–33. 10.3732/ajb.92.3.525. 21652431

[2] Dunn S . New Chinese plants. J Bot. 1908;46:324–26.

[3] de Wit HC . A revision of Malaysian Bauhinieae. Reinwardtia. 1956;3:381–41.

[4] Sharma AK, Raju DT. Structure and behaviour of chromosomes in *Bauhinia* and allied genera. Cytologia (Tokyo). 1968;33:411–26. 10.1508/cytologia.33.411.

[5] Zhong Y, Chen Y, Zheng D, et al. Chromosomal-level genome assembly of the orchid tree *bauhinia variegata* (Leguminosae; Cercidoideae) supports the allotetraploid origin hypothesis of Bauhinia. DNA Res. 2022;29(2):dsac012. 10.1093/dnares/dsac012. 35438173 PMC9052405

[6] Mak CY, Cheung KS, Yip PY, et al. Molecular evidence for the hybrid origin of *Bauhinia blakeana* (Caesalpinioideae). JIPB. 2008;50:111–18. 10.1111/j.1744-7909.2007.00591.x. 18666958

[7] Yuping L, Yingxiong Q, Chan YSG. Dentification of three species in Bauhinia and hybrid origin of *Bauhinia blakeana* using ISSR markers. Acta Horticult Sinica. 2006;33:433.

[8] Satam H, Joshi K, Mangrolia U, et al. Next-generation sequencing technology: current trends and advancements. Biology (Basel). 2023;12(7):997. 10.3390/biology12070997. 37508427 PMC10376292

[9] Michael TP, VanBuren R. Building near-complete plant genomes. Curr Opin Plant Biol. 2020;54:26–33. 10.1016/j.pbi.2019.12.009. 31981929

[10] Kress WJ, Soltis DE, Kersey PJ, et al. Green plant genomes: what we know in an era of rapidly expanding opportunities. Proc Natl Acad Sci U S A. 2022;119(4):e2115640118. 10.1073/pnas.2115640118. 35042803 PMC8795535

[11] Garg S, Fungtammasan A, Carroll A, et al. Chromosome-scale, haplotype-resolved assembly of human genomes. Nat Biotechnol. 2021;39:309–12. 10.1038/s41587-020-0711-0. 33288905 PMC7954703

[12] Koren S, Rhie A, Walenz BP, et al. De novo assembly of haplotype-resolved genomes with trio binning. Nat Biotechnol. 2018;36:1174–82. 10.1038/nbt.4277. PMC6476705 30346939

[13] Zhang X, Zhang S, Zhao Q, et al. Assembly of allele-aware, chromosomal-scale autopolyploid genomes based on hi-C data. Nat Plants. 2019;5:833–45. 10.1038/s41477-019-0487-8. 31383970

[14] Lazarus S . Unique project to sequence the genome of the Hong Kong bauhinia tree. SCMP. 2015. https://www.scmp.com/magazines/post-magazine/article/1875917/unique-project-sequence-genome-hong-kong-bauhinia-tree.Accessed 14 April 2025.

[15] Tang WM, Kwok JSL, Genome Community Bauhinia, et al. Transcriptome assemblies of three *Bauhinia* species. GigaScience Database. 2018. 10.5524/100345.

[16] Huang X, Yang S, Gong J, et al. Genomic architecture of heterosis for yield traits in rice. Nature. 2016;537:629–33. 10.1038/nature19760. 27602511

[17] Baranwal VK, Mikkilineni V, Zehr UB, et al. Heterosis: emerging ideas about hybrid vigour. J Exp Bot. 2012;63:6309–14. 10.1093/jxb/ers291. 23095992

[18] Chen ZJ . Genomic and epigenetic insights into the molecular bases of heterosis. Nat Rev Genet. 2013;14:471–82. 10.1038/nrg3503. 23752794

[19] Hochholdinger F, Baldauf JA. Heterosis in plants. Curr Biol. 2018;28:R1089–92. 10.1016/j.cub.2018.06.041. 30253145

[20] Swanson-Wagner RA, Jia Y, DeCook R, et al. All possible modes of gene action are observed in a global comparison of gene expression in a maize F1 hybrid and its inbred parents. Proc Natl Acad Sci U S A. 2006;103:6805–10. 10.1073/pnas.0510430103. 16641103 PMC1447595

[21] Ma X, Xing F, Jia Q, et al. Parental variation in CHG methylation is associated with allelic-specific expression in elite hybrid rice. Plant Physiol. 2021;186:1025–41. 10.1093/plphys/kiab088. 33620495 PMC8195538

[22] Li D, Lu X, Zhu Y, et al. The multi-omics basis of potato heterosis. JIPB. 2022;64:671–87. 10.1111/jipb.13211. 34963038

[23] Springer NM, Stupar RM. Allele-specific expression patterns reveal biases and embryo-specific parent-of-origin effects in hybrid maize. Plant Cell. 2007;19:2391–402. 10.1105/tpc.107.052258. 17693532 PMC2002603

[24] Shao L, Xing F, Xu CH, et al. Patterns of genome-wide allele-specific expression in hybrid rice and the implications on the genetic basis of heterosis. Proc Natl Acad Sci U S A. 2019;116:5653–58. 10.1073/pnas.1820513116. 30833384 PMC6431163

[25] Chikhi R, Medvedev P. Informed and automated k-mer size selection for genome assembly. Bioinformatics. 2014;30:31–37. 10.1093/bioinformatics/btt310. 23732276

[26] Weisenfeld NI, Kumar V, Shah P, et al. Direct determination of diploid genome sequences. Genome Res. 2017;27:757–67. 10.1101/gr.214874.116. 28381613 PMC5411770

[27] Simao FA, Waterhouse RM, Ioannidis P, et al. BUSCO: assessing genome assembly and annotation completeness with single-copy orthologs. Bioinformatics. 2015;31:3210–12. 10.1093/bioinformatics/btv351. 26059717

[28] Creating diploid assemblies from Nanopore and Illumina reads with hypo-assembler. Nat Methods. 2024;21:560–61. 10.1038/s41592-023-02142-0. 38459387

[29] Rhie A, Walenz BP, Koren S, et al. Merqury: reference-free quality, completeness, and phasing assessment for genome assemblies. Genome Biol. 2020;21:1–27. 10.1186/s13059-020-02134-9. PMC7488777 32928274

[30] Boeckmann B, Bairoch A, Apweiler R, et al. The SWISS-PROT protein knowledgebase and its supplement TrEMBL in 2003. Nucleic Acids Res. 2003;31:365–70. 10.1093/nar/gkg095. 12520024 PMC165542

[31] Kanehisa M, Araki M, Goto S, et al. KEGG for linking genomes to life and the environment. Nucleic Acids Res. 2008;36:D480–84. 10.1093/nar/gkm882. 18077471 PMC2238879

[32] Tatusov RL, Fedorova ND, Jackson JD, et al. The COG database: an updated version includes eukaryotes. BMC Bioinf. 2003;4:41. 10.1186/1471-2105-4-41. PMC222959 12969510

[33] Hunter S, Apweiler R, Attwood TK, et al. InterPro: the integrative protein signature database. Nucleic Acids Res. 2009;37:D211–15. 10.1093/nar/gkn785. 18940856 PMC2686546

[34] Lin Y, Ye C, Li X, et al. quarTeT: a telomere-to-telomere toolkit for gap-free genome assembly and centromeric repeat identification. Hortic Res. 2023;10:uhad127. 10.1093/hr/uhad127. 37560017 PMC10407605

[35] Goel M, Sun H, Jiao WB, et al. SyRI: finding genomic rearrangements and local sequence differences from whole-genome assemblies. Genome Biol. 2019;20:277. 10.1186/s13059-019-1911-0. 31842948 PMC6913012

[36] Chen F, Tholl D, Bohlmann J, et al. The family of terpene synthases in plants: a mid-size family of genes for specialized metabolism that is highly diversified throughout the kingdom. Plant J. 2011;66:212–29. 10.1111/j.1365-313X.2011.04520.x. 21443633

[37] Aubourg S, Lecharny A, Bohlmann J. Genomic analysis of the terpenoid synthase (AtTPS) gene family of Arabidopsis thaliana. Mol Gen Genomics. 2002;267:730–45. 10.1007/s00438-002-0709-y. 12207221

[38] Thompson JD, Higgins DG, Gibson TJ. CLUSTAL W: improving the sensitivity of progressive multiple sequence alignment through sequence weighting, position-specific gap penalties and weight matrix choice. Nucl Acids Res. 1994;22:4673–80. 10.1093/nar/22.22.4673. 7984417 PMC308517

[39] Frazer KA, Pachter L, Poliakov A, et al. VISTA: computational tools for comparative genomics. Nucleic Acids Res. 2004;32:W273–79. 10.1093/nar/gkh458. 15215394 PMC441596

[40] Xiao Y, Qu YY, Hao CH, et al. The complete chloroplast genome of *Bauhinia racemosa* Lam. (Fabaceae): a versatile tropical medicinal plant. Mitochondrial DNA Part B. 2022;7:1528–30. 10.1080/23802359.2022.2110010. 36034532 PMC9415444

[41] Shang H-Y, Jia K-H, Li N-W, et al. Phytop: a tool for visualizing and recognizing signals of incomplete lineage sorting and hybridization using species trees output from ASTRAL. Horticult Res. 2024;12(3):uhae330. 10.1093/hr/uhae330. PMC11879507 40046323

[42] Stupar RM, Springer NM. Cis-transcriptional variation in maize inbred lines B73 and Mo17 leads to additive expression patterns in the F1 hybrid. Genetics. 2006;173:2199–210. 10.1534/genetics.106.060699. 16702414 PMC1569691

[43] Carretero-Paulet L, Ahumada I, Cunillera N, et al. Expression and molecular analysis of the Arabidopsis DXR gene encoding 1-deoxy-D-xylulose 5-phosphate reductoisomerase, the first committed enzyme of the 2-C-methyl-D-erythritol 4-phosphate pathway. Plant Physiol. 2002;129:1581–91. 10.1104/pp.003798. 12177470 PMC166745

[44] Carretero-Paulet L, Cairo A, Botella-Pavía P, et al. Enhanced flux through the methylerythritol 4-phosphate pathway in Arabidopsis plants overexpressing deoxyxylulose 5-phosphate reductoisomerase. Plant Mol Biol. 2006;62:683–95. 10.1007/s11103-006-9051-9. 16941216

[45] Cochrane FC, Davin LB, Lewis NG. The Arabidopsis phenylalanine ammonia lyase gene family: kinetic characterization of the four PAL isoforms. Phytochemistry. 2004;65:1557–64. 10.1016/j.phytochem.2004.05.006. 15276452

[46] Lichtenthaler HK . The 1-deoxy-D-xylulose-5-phosphate pathway of isoprenoid biosynthesis in plants. Annu Rev Plant Physiol Plant Mol Biol. 1999;50:47–65. 10.1146/annurev.arplant.50.1.47. 15012203

[47] Gudavalli D, Pandey K, Ede VG, et al. Phytochemistry and pharmacological activities of five species of Bauhinia genus: a review. Fitoterapia. 2024;174:105830. 10.1016/j.fitote.2024.105830. 38286316

[48] da Fonseca STD, Teixeira TR, Ferreira JMS, et al. Flavonoid-rich fractions of *bauhinia holophylla* leaves inhibit *Candida albicans* biofilm formation and hyphae growth. Plants (Basel). 2022;11(14):1796. 10.3390/plants11141796. 35890430 PMC9322443

[49] Chinnappan S, Kandasamy S, Arumugam S, et al. Biomimetic synthesis of silver nanoparticles using flower extract of *Bauhinia purpurea* and its antibacterial activity against clinical pathogens. Environ Sci Pollut Res. 2018;25:963–69. 10.1007/s11356-017-0841-1. 29218578

[50] Mishra A, Sharma AK, Kumar S, et al. *Bauhinia variegata* leaf extracts exhibit considerable antibacterial, antioxidant, and anticancer activities. Biomed Res Int. 2013;2013:1. 10.1155/2013/915436. PMC3777169 24093108

[51] Liu L, Guan D, Peart MR. The morphological structure of leaves and the dust-retaining capability of afforested plants in urban Guangzhou, South China. Environ Sci Pollut Res. 2012;19:3440–49. 10.1007/s11356-012-0876-2. 22614051

[52] Seymour DK, Chae E, Grimm DG, et al. Genetic architecture of nonadditive inheritance in Arabidopsis thaliana hybrids. Proc Natl Acad Sci U S A. 2016;113:E7317–26. 10.1073/pnas.1615268113. 27803326 PMC5135357

[53] Birchler JA, Yao H, Chudalayandi S, et al. Heterosis. Plant Cell. 2010;22:2105–12. 10.1105/tpc.110.076133. 20622146 PMC2929104

[54] Xiao J, Li J, Yuan L, et al. Dominance is the major genetic basis of heterosis in rice as revealed by QTL analysis using molecular markers. Genetics. 1995;140:745–54. 10.1093/genetics/140.2.745. 7498751 PMC1206649

[55] Li Z-K, Luo L, Mei H, et al. Overdominant epistatic loci are the primary genetic basis of inbreeding depression and heterosis in rice. I. Biomass and grain yield. Genetics. 2001;158:1737–53. 10.1093/genetics/158.4.1737. 11514459 PMC1461764

[56] Janko K, Eisner J, Cigler P, et al. Unifying framework explaining how parental regulatory divergence can drive gene expression in hybrids and allopolyploids. Nat Commun. 2024;15:8714. 10.1038/s41467-024-52546-5. 39379366 PMC11461870

[57] Combes M-C, Hueber Y, Dereeper A, et al. Regulatory divergence between parental alleles determines gene expression patterns in hybrids. Genome Biol Evolut. 2015;7:1110–21. 10.1093/gbe/evv057. PMC4419803 25819221

[58] Matthews PD, Wurtzel ET. Metabolic engineering of carotenoid accumulation in Escherichia coli by modulation of the isoprenoid precursor pool with expression of deoxyxylulose phosphate synthase. Appl Microbiol Biotechnol. 2000;53:396–400. 10.1007/s002530051632. 10803894

[59] Carretero-Paulet L, Cairó A, Botella-Pavía P, et al. Enhanced flux through the methylerythritol 4-phosphate pathway in Arabidopsis plants overexpressing deoxyxylulose 5-phosphate reductoisomerase. Plant Mol Biol. 2006;62:683–95. 10.1007/s11103-006-9051-9. 16941216

[60] Springer NM, Stupar RM. Allelic variation and heterosis in maize: how do two halves make more than a whole?. Genome Res. 2007;17:264–75. 10.1101/gr.5347007. 17255553

[61] Guo M, Rupe MA, Yang X, et al. Genome-wide transcript analysis of maize hybrids: allelic additive gene expression and yield heterosis. Theor Appl Genet. 2006;113(5):831–45. 10.1007/s00122-006-0335-x. 16868764

[62] Goff SA, Zhang Q. Heterosis in elite hybrid rice: speculation on the genetic and biochemical mechanisms. Curr Opin Plant Biol. 2013;16:221–27. 10.1016/j.pbi.2013.03.009. 23587937

[63] Sahu SK, Thangaraj M, Kathiresan K. DNA extraction protocol for plants with high levels of secondary metabolites and polysaccharides without using liquid nitrogen and phenol. ISRN Molecular Biology. 2012;2012:1. 10.5402/2012/205049. PMC4890884 27335662

[64] Wang O, Chin R, Cheng X, et al. Efficient and unique cobarcoding of second-generation sequencing reads from long DNA molecules enabling cost-effective and accurate sequencing, haplotyping, and de novo assembly. Genome Res. 2019;29:798–808. 10.1101/gr.245126.118. 30940689 PMC6499310

[65] Lieberman-Aiden E, Van Berkum NL, Williams L, et al. Comprehensive mapping of long-range interactions reveals folding principles of the human genome. Science. 2009;326:289–93. 10.1126/science.1181369. 19815776 PMC2858594

[66] Huang J, Liang XM, Xuan YK, et al. BGISEQ-500 WGS library construction. *Protocols.io*. 2018. 10.17504/protocols.io.ps5dng6.Accessed on 2025 April 14.

[67] Ashton PM, Nair S, Dallman T, et al. MinION nanopore sequencing identifies the position and structure of a bacterial antibiotic resistance island. Nat Biotechnol. 2015;33:296–300. 10.1038/nbt.3103. 25485618

[68] Bolger AM, Lohse M, Usadel B. Trimmomatic: a flexible trimmer for Illumina sequence data. Bioinformatics. 2014;30:2114–20. 10.1093/bioinformatics/btu170. 24695404 PMC4103590

[69] Marcais G, Kingsford C. A fast, lock-free approach for efficient parallel counting of occurrences of k-mers. Bioinformatics. 2011;27:764–70. 10.1093/bioinformatics/btr011. 21217122 PMC3051319

[70] Vurture GW, Sedlazeck FJ, Nattestad M, et al. GenomeScope: fast reference-free genome profiling from short reads. Bioinformatics. 2017;33:2202–4. 10.1093/bioinformatics/btx153. 28369201 PMC5870704

[71] Li H, Durbin R. Fast and accurate short read alignment with Burrows-Wheeler transform. Bioinformatics. 2009;25:1754–60. 10.1093/bioinformatics/btp324. 19451168 PMC2705234

[72] Danecek P, Bonfield JK, Liddle J, et al. Twelve years of SAMtools and BCFtools. Gigascience. 2021;10: giab008. 10.1093/gigascience/giab008. 33590861 PMC7931819

[73] Repeat Library Construction-Advanced. http://weatherby.genetics.utah.edu/MAKER/wiki/index.php/Repeat_Library_Construction-Advanced. Accessed 12 December 2024.

[74] Chen N . Using RepeatMasker to identify repetitive elements in genomic sequences. Curr Protoc Bioinformatics. 2004;5. Chapter 4:Unit 4.10. 10.1002/0471250953.bi0410s05. 18428725

[75] Bao WD, Kojima KK, Kohany O. Repbase Update, a database of repetitive elements in eukaryotic genomes. Mobile DNA. 2015;6:1–6. 10.1186/s13100-015-0041-9. PMC4455052 26045719

[76] Xu Z, Wang H. LTR_FINDER: an efficient tool for the prediction of full-length LTR retrotransposons. Nucleic Acids Res. 2007;35:W265–68. 10.1093/nar/gkm286. 17485477 PMC1933203

[77] Flynn JM, Hubley R, Goubert C, et al. RepeatModeler2 for automated genomic discovery of transposable element families. Proc Natl Acad Sci U S A. 2020;117:9451–57. 10.1073/pnas.1921046117. 32300014 PMC7196820

[78] Benson G . Tandem repeats finder: a program to analyze DNA sequences. Nucleic Acids Res. 1999;27:573–80. 10.1093/nar/27.2.573. 9862982 PMC148217

[79] Bruna T, Hoff KJ, Lomsadze A, et al. BRAKER2: automatic eukaryotic genome annotation with GeneMark-EP plus and AUGUSTUS supported by a protein database. Nar Genomics Bioinformatics. 2021;3(1):lqaa108. 10.1093/nargab/lqaa108. 33575650 PMC7787252

[80] Kim D, Paggi JM, Park C, et al. Graph-based genome alignment and genotyping with HISAT2 and HISAT-genotype. Nat Biotechnol. 2019;37:907–15. 10.1038/s41587-019-0201-4. 31375807 PMC7605509

[81] Kriventseva EV, Kuznetsov D, Tegenfeldt F, et al. OrthoDB v10: sampling the diversity of animal, plant, fungal, protist, bacterial and viral genomes for evolutionary and functional annotations of orthologs. Nucleic Acids Res. 2019;47:D807–11. 10.1093/nar/gky1053. 30395283 PMC6323947

[82] Marcais G, Delcher AL, Phillippy AM, et al. MUMmer4: a fast and versatile genome alignment system. PLoS Comput Biol. 2018;14:e1005944. 10.1371/journal.pcbi.1005944. 29373581 PMC5802927

[83] Lowe TM, Eddy SR. tRNAscan-SE: a program for improved detection of transfer RNA genes in genomic sequence. Nucleic Acids Res. 1997;25(5):955–64. 10.1093/nar/25.5.955. 9023104 PMC146525

[84] Nawrocki EP, Kolbe DL, Eddy SR. Infernal 1.0: inference of RNA alignments. Bioinformatics. 2009;25:1335–37. 10.1093/bioinformatics/btp157. 19307242 PMC2732312

[85] Zheng Y, Jiao C, Sun H, et al. iTAK: a program for genome-wide prediction and classification of plant transcription factors, transcriptional regulators, and protein kinases. Mol Plant. 2016;9:1667–70. 10.1016/j.molp.2016.09.014. 27717919

[86] Emms DM, Kelly S. OrthoFinder: phylogenetic orthology inference for comparative genomics. Genome Biol. 2019;20:238. 10.1186/s13059-019-1832-y. 31727128 PMC6857279

[87] Katoh K, Standley DM. MAFFT multiple sequence alignment software version 7: improvements in performance and usability. Mol Biol Evol. 2013;30:772–80. 10.1093/molbev/mst010. 23329690 PMC3603318

[88] Castresana J . Selection of conserved blocks from multiple alignments for their use in phylogenetic analysis. Mol Biol Evol. 2000;17:540–52. 10.1093/oxfordjournals.molbev.a026334. 10742046

[89] Nguyen LT, Schmidt HA, von Haeseler A, et al. IQ-TREE: a fast and effective stochastic algorithm for estimating maximum-likelihood phylogenies. Mol Biol Evol. 2015;32:268–74. 10.1093/molbev/msu300. 25371430 PMC4271533

[90] Zhang C, Rabiee M, Sayyari E, et al. ASTRAL-III: polynomial time species tree reconstruction from partially resolved gene trees. BMC Bioinf. 2018;19:153. 10.1186/s12859-018-2129-y. PMC5998893 29745866

[91] Stamatakis A . RAxML version 8: a tool for phylogenetic analysis and post-analysis of large phylogenies. Bioinformatics. 2014;30:1312–13. 10.1093/bioinformatics/btu033. 24451623 PMC3998144

[92] Yang Z . PAML 4: phylogenetic analysis by maximum likelihood. Mol Biol Evol. 2007;24:1586–91. 10.1093/molbev/msm088. 17483113

[93] De Bie T, Cristianini N, Demuth JP, et al. CAFE: a computational tool for the study of gene family evolution. Bioinformatics. 2006;22:1269–71. 10.1093/bioinformatics/btl097. 16543274

[94] Huang da W, Sherman BT, Lempicki RA. Bioinformatics enrichment tools: paths toward the comprehensive functional analysis of large gene lists. Nucleic Acids Res. 2009;37:1–13. 10.1093/nar/gkn923. 19033363 PMC2615629

[95] Chen S, Yang P, Jiang F, et al. De novo analysis of transcriptome dynamics in the migratory locust during the development of phase traits. PLoS One. 2010;5:e15633. 10.1371/journal.pone.0015633. 21209894 PMC3012706

[96] Jin JJ, Yu WB, Yang JB, et al. GetOrganelle: a fast and versatile toolkit for accurate de novo assembly of organelle genomes. Genome Biol. 2020;21:1–31. 10.1186/s13059-020-02154-5. PMC7488116 32912315

[97] Shi L, Chen H, Jiang M, et al. CPGAVAS2, an integrated plastome sequence annotator and analyzer. Nucleic Acids Res. 2019;47:W65–W73. 10.1093/nar/gkz345. 31066451 PMC6602467

[98] Mayor C, Brudno M, Schwartz JR, et al. VISTA: visualizing global DNA sequence alignments of arbitrary length. Bioinformatics. 2000;16:1046–47. 10.1093/bioinformatics/16.11.1046. 11159318

[99] Roberts A, Pachter L. Streaming fragment assignment for real-time analysis of sequencing experiments. Nat Methods. 2013;10:71–73. 10.1038/nmeth.2251. 23160280 PMC3880119

[100] Love MI, Huber W, Anders S. Moderated estimation of fold change and dispersion for RNA-seq data with DESeq2. Genome Biol. 2014;15:550. 10.1186/s13059-014-0550-8. 25516281 PMC4302049

[101] Wang Y, Tang H, Debarry JD, et al. MCScanX: a toolkit for detection and evolutionary analysis of gene synteny and collinearity. Nucleic Acids Res. 2012;40:e49. 10.1093/nar/gkr1293. 22217600 PMC3326336

[102] Mu W, Darian JC, Sung W, et al. Supporting data for “The Haplotype-Resolved T2T Genome for *Bauhinia* × *blakeana* Sheds Light on the Genetic Basis of Flower Heterosis.” GigaScience Database. 2025. 10.5524/102678.40276955

[103] Mu W, Darian JC, Sung W, et al. Genome assembly of the orchid tree *Bauhinia purpurea*. GigaScience Database. 2025. 10.5524/102680.

[104] Mu W, Darian JC, Sung W, et al. Genome assembly of the orchid tree *Bauhinia variegata*. GigaScience Database. 2025. 10.5524/102681.

[105] Mu W, Darian JC, Sung W, et al. Genome assembly of the Hong Kong orchid tree *Bauhinia blakeana*. GigaScience Database. 2025.; 10.5524/102679.

