# Supplementary material for: The haplotype-resolved T2T genome for Bauhinia × blakeana sheds light on the genetic basis of flower heterosis
Source: Gigascience. 2025 Apr 25;14:giaf044. doi: 10.1093/gigascience/giaf044 (PMC12012898; doi:10.1093/gigascience/giaf044)
Supplement: giaf044_Supplemental_Files [file giaf044_supplemental_files.zip › Fig S4.pdf]

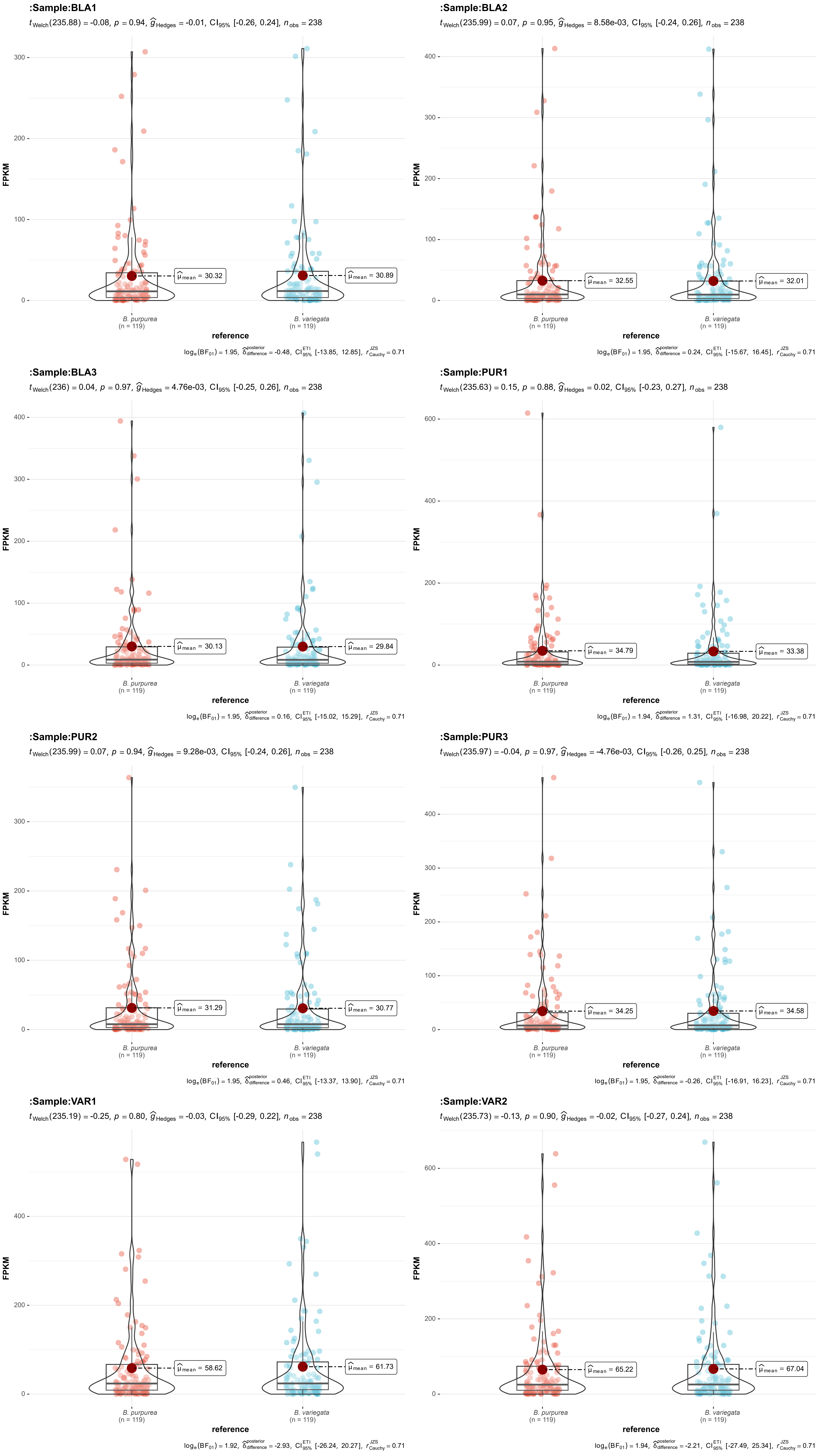
:Sample:BLA1
tWelch(235.88) = -0.08, p = 0.94, ĝHedges = -0.01, CI95% [-0.26, 0.24], nobs = 238
FPKM
μ̂mean = 30.32
μ̂mean = 30.89
B. purpurea
(n = 119)
B. variegata
(n = 119)
reference
loge(BF01) = 1.95, δ̂posterior difference = -0.48, CIETI 95% [-13.85, 12.85], rJZS Cauchy = 0.71
:Sample:BLA2
tWelch(235.99) = 0.07, p = 0.95, ĝHedges = 8.58e-03, CI95% [-0.24, 0.26], nobs = 238
FPKM
μ̂mean = 32.55
μ̂mean = 32.01
B. purpurea
(n = 119)
B. variegata
(n = 119)
reference
loge(BF01) = 1.95, δ̂posterior difference = 0.24, CIETI 95% [-15.67, 16.45], rJZS Cauchy = 0.71
:Sample:BLA3
tWelch(236) = 0.04, p = 0.97, ĝHedges = 4.76e-03, CI95% [-0.25, 0.26], nobs = 238
FPKM
μ̂mean = 30.13
μ̂mean = 29.84
B. purpurea
(n = 119)
B. variegata
(n = 119)
reference
loge(BF01) = 1.95, δ̂posterior difference = 0.16, CIETI 95% [-15.02, 15.29], rJZS Cauchy = 0.71
:Sample:PUR1
tWelch(235.63) = 0.15, p = 0.88, ĝHedges = 0.02, CI95% [-0.23, 0.27], nobs = 238
FPKM
μ̂mean = 34.79
μ̂mean = 33.38
B. purpurea
(n = 119)
B. variegata
(n = 119)
reference
loge(BF01) = 1.94, δ̂posterior difference = 1.31, CIETI 95% [-16.98, 20.22], rJZS Cauchy = 0.71
:Sample:PUR2
tWelch(235.99) = 0.07, p = 0.94, ĝHedges = 9.28e-03, CI95% [-0.24, 0.26], nobs = 238
FPKM
μ̂mean = 31.29
μ̂mean = 30.77
B. purpurea
(n = 119)
B. variegata
(n = 119)
reference
loge(BF01) = 1.95, δ̂posterior difference = 0.46, CIETI 95% [-13.37, 13.90], rJZS Cauchy = 0.71
:Sample:PUR3
tWelch(235.97) = -0.04, p = 0.97, ĝHedges = -4.76e-03, CI95% [-0.26, 0.25], nobs = 238
FPKM
μ̂mean = 34.25
μ̂mean = 34.58
B. purpurea
(n = 119)
B. variegata
(n = 119)
reference
loge(BF01) = 1.95, δ̂posterior difference = -0.26, CIETI 95% [-16.91, 16.23], rJZS Cauchy = 0.71
:Sample:VAR1
tWelch(235.19) = -0.25, p = 0.80, ĝHedges = -0.03, CI95% [-0.29, 0.22], nobs = 238
FPKM
μ̂mean = 58.62
μ̂mean = 61.73
B. purpurea
(n = 119)
B. variegata
(n = 119)
reference
loge(BF01) = 1.92, δ̂posterior difference = -2.93, CIETI 95% [-26.24, 20.27], rJZS Cauchy = 0.71
:Sample:VAR2
tWelch(235.73) = -0.13, p = 0.90, ĝHedges = -0.02, CI95% [-0.27, 0.24], nobs = 238
FPKM
μ̂mean = 65.22
μ̂mean = 67.04
B. purpurea
(n = 119)
B. variegata
(n = 119)
reference
loge(BF01) = 1.94, δ̂posterior difference = -2.21, CIETI 95% [-27.49, 25.34], rJZS Cauchy = 0.71
