# Supplementary figures and images for: The haplotype-resolved T2T genome for Bauhinia × blakeana sheds light on the genetic basis of flower heterosis
Source: Gigascience. 2025 Apr 25;14:giaf044. doi: 10.1093/gigascience/giaf044 (PMC12012898; doi:10.1093/gigascience/giaf044)

A

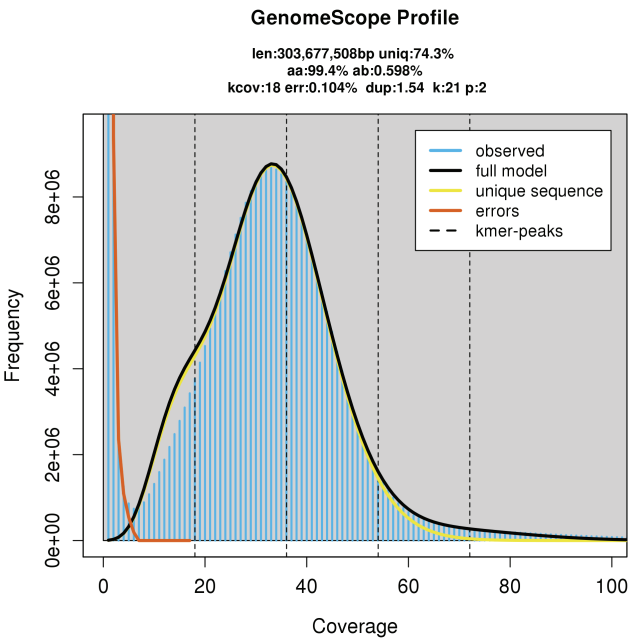

B

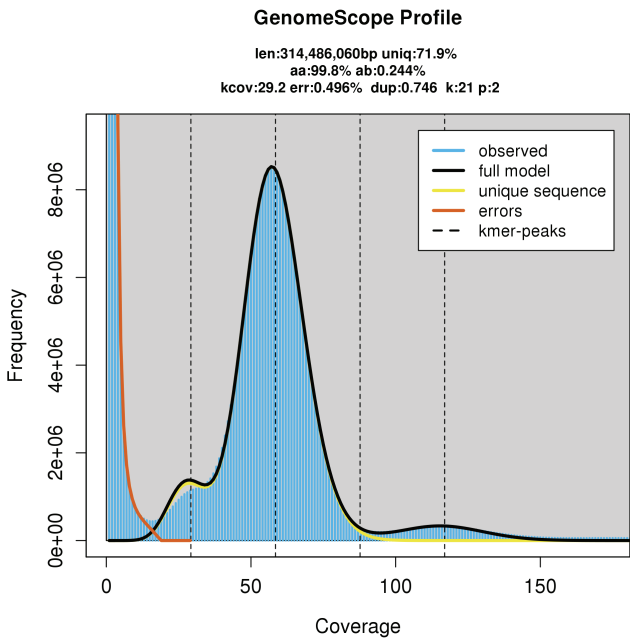

C

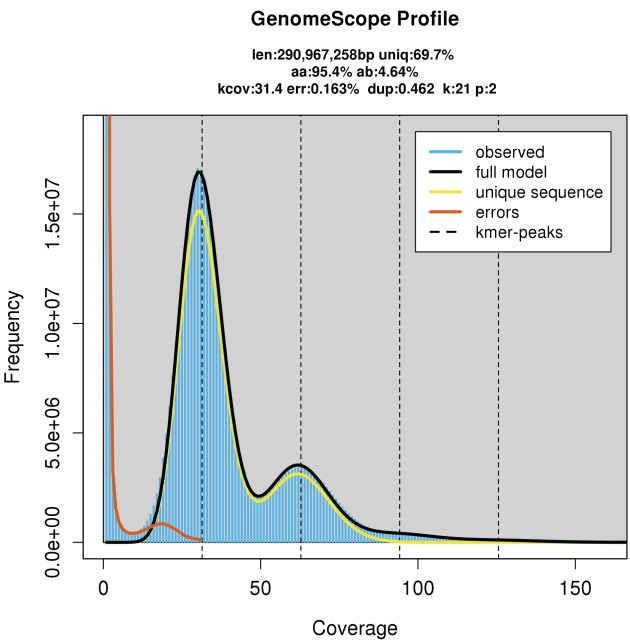

Supplement: giaf044_Supplemental_Files [file giaf044_supplemental_files.zip › Fig S1.pdf]

A

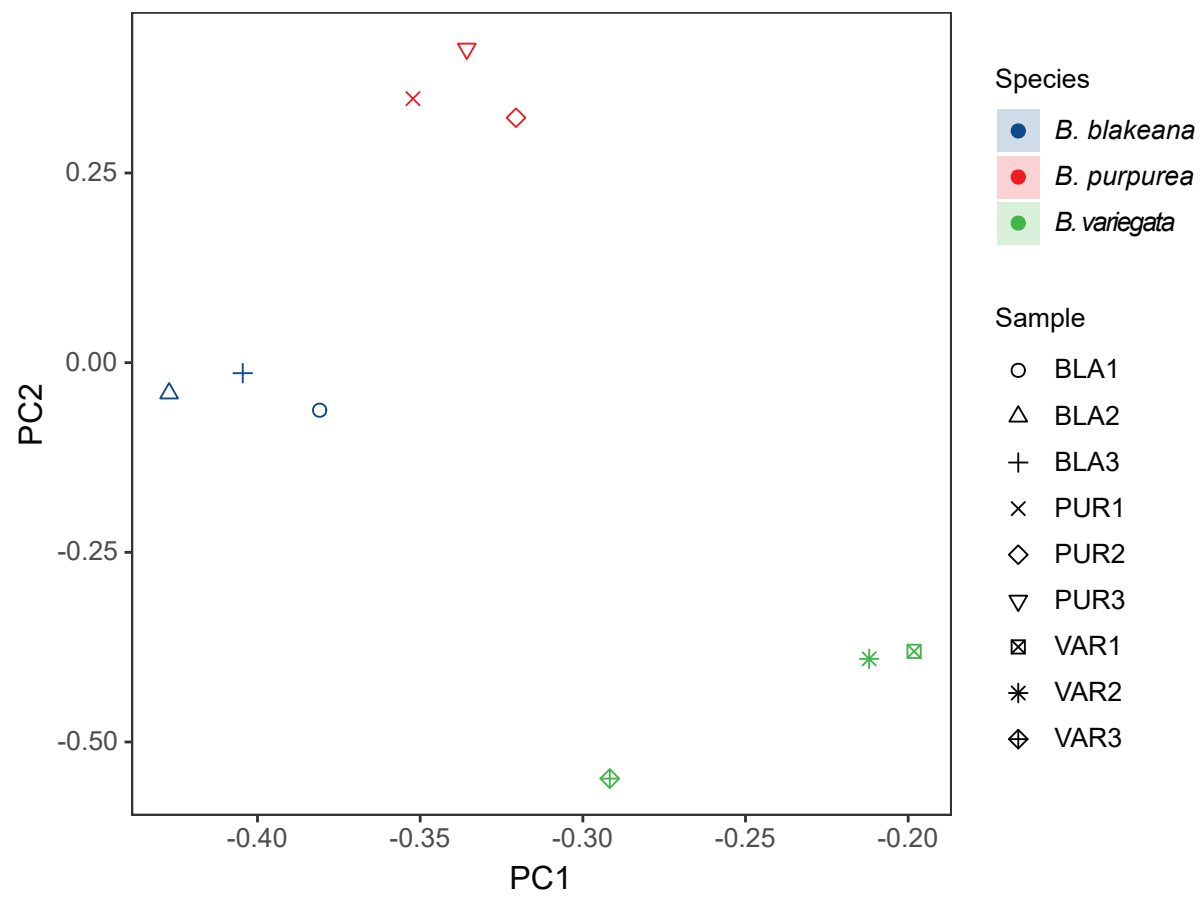

Supplement: giaf044_Supplemental_Files [file giaf044_supplemental_files.zip › Fig S3.pdf]

A

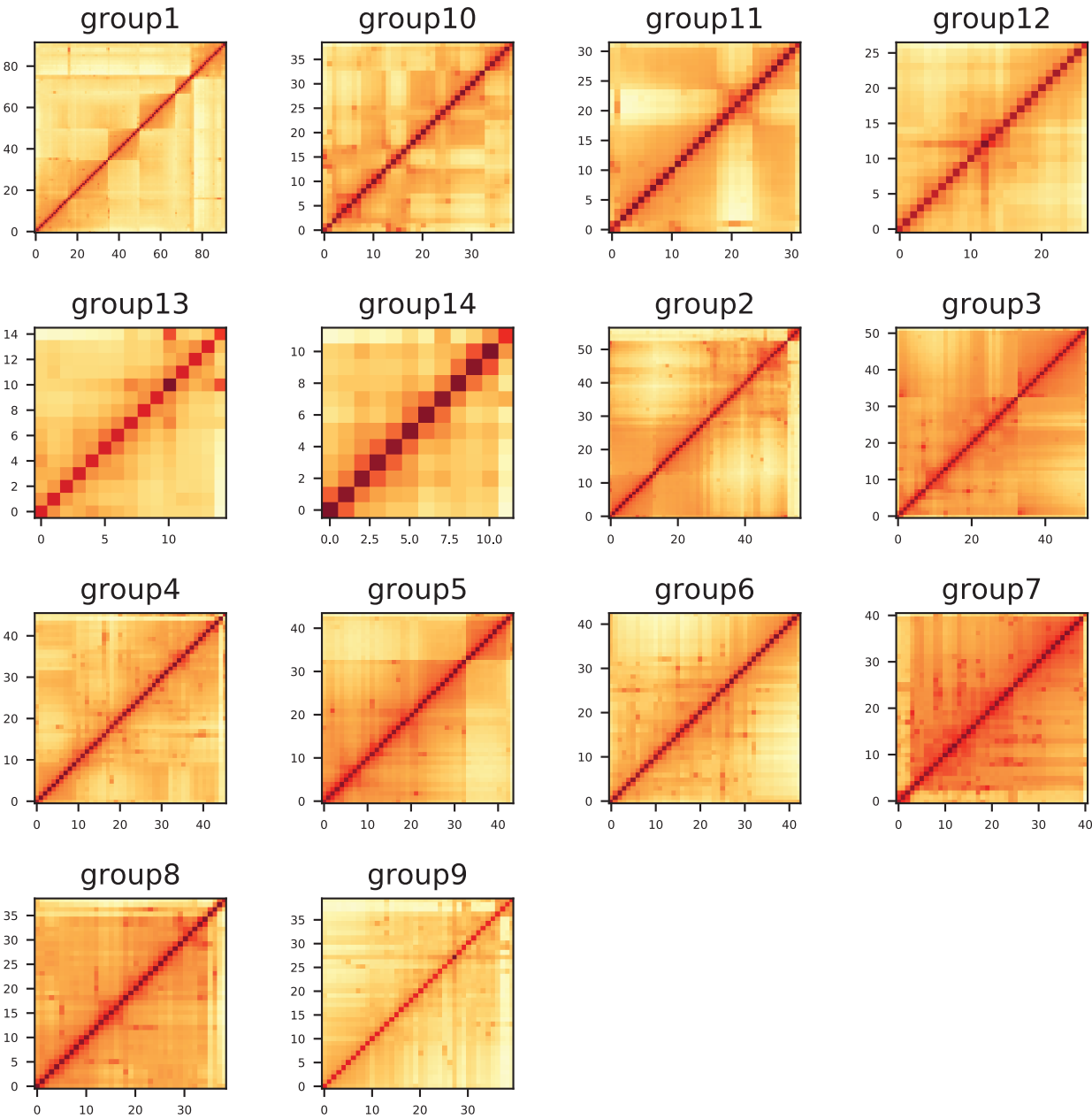

B

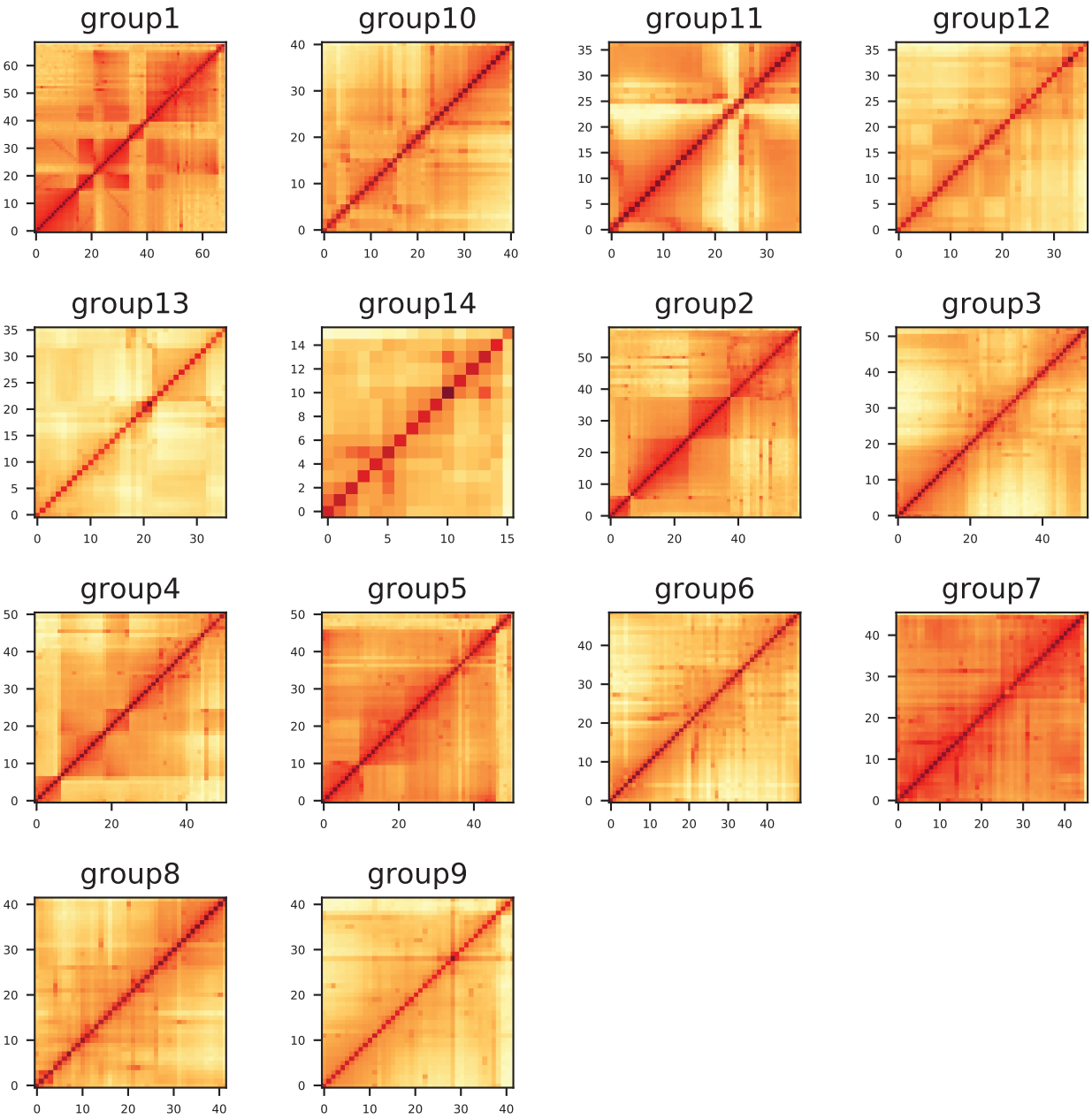

Supplement: giaf044_Supplemental_Files [file giaf044_supplemental_files.zip › Figure S2.pdf]

A

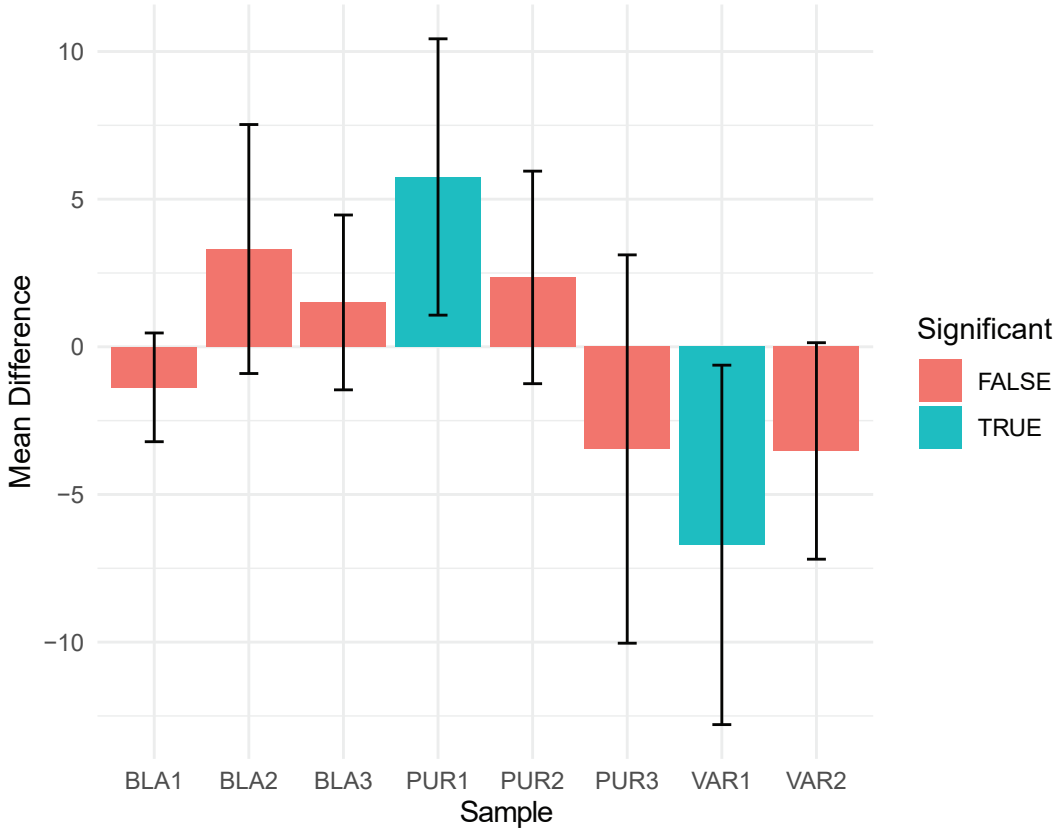

B

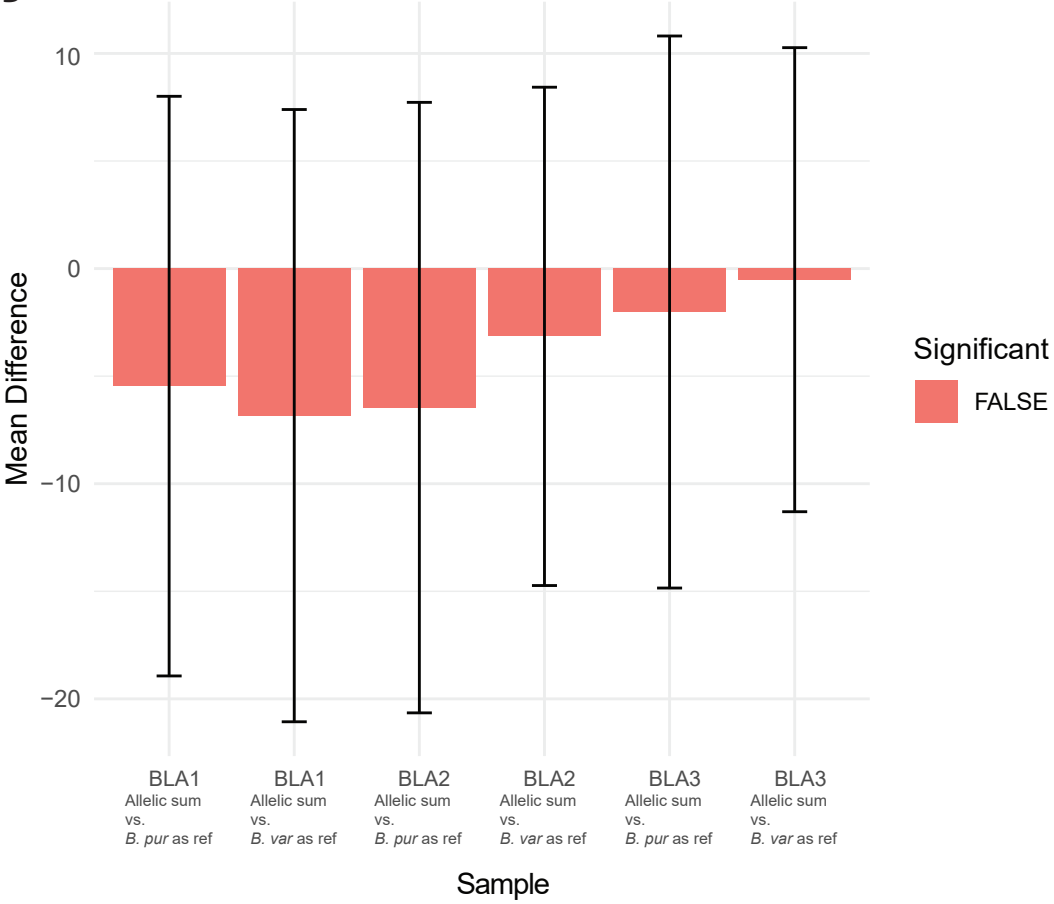

Supplement: giaf044_Supplemental_Files [file giaf044_supplemental_files.zip › Figure S5.pdf]
