# Supplementary material for: The haplotype-resolved T2T genome for Bauhinia × blakeana sheds light on the genetic basis of flower heterosis
Source: Gigascience. 2025 Apr 25;14:giaf044. doi: 10.1093/gigascience/giaf044 (PMC12012898; doi:10.1093/gigascience/giaf044)
Supplement: giaf044_GIGA-D-24-00537_Original_Submission [file giaf044_giga-d-24-00537_original_submission.pdf]

GigaScience

The Haplotype-resolved T2T Genome for Bauhinia x blakeana Sheds Light on the Genetic Basis of Flower Heterosis

--Manuscript Draft--

**Manuscript Number:**	GIGA-D-24-00537
**Full Title:**	The Haplotype-resolved T2T Genome for Bauhinia x blakeana Sheds Light on the Genetic Basis of Flower Heterosis
**Article Type:**	Research
**Funding Information:**	Collaborative Research Grant, Research Grants Council (C4049-23EF) — Dr. Stephen Kwok Wing Tsui
**Abstract:**	Background<br>The Hong Kong Orchid Tree Bauhinia x blakeana Dunn has long been proposed to be a sterile interspecific hybrid exhibiting flower heterosis when compared to its likely parental species, B. purpurea L. and B. variegata L. Here, we report comparative genomic and transcriptomic analyses of the three Bauhinia species.<br>Findings<br>We generated chromosome-level assemblies for the parental species and applied a trio-binning approach to construct a haplotype-resolved telomere-to-telomere (T2T) genome for B. blakeana. Comparative chloroplast genome analysis confirmed B. purpurea as the maternal parent. Transcriptome profiling of flower tissues highlighted a closer resemblance of B. blakeana to its maternal parent. Differential gene expression analyses revealed distinct expression patterns among the three species, particularly in biosynthetic and metabolic processes. To investigate the genetic basis of flower heterosis observed in B. blakeana, we focused on gene expression patterns within pigment biosynthesis-related pathways. High-patent dominance and over-dominance expression patterns were observed, particularly in genes associated with carotenoid biosynthesis. Additionally, allele-specific expression analysis revealed a balanced contribution of maternal and paternal alleles in shaping the gene expression patterns in B. blakeana.<br>Conclusions<br>Our study offers valuable insights into the genome architecture of hybrid B. blakeana, establishing a comprehensive genomic and transcriptomic resource for future functional genetics research within the Bauhinia genus. It also serves as a model for exploring the characteristics of hybrid species using T2T haplotype-resolved genomes, providing a novel approach to understanding genetic interactions and evolutionary mechanisms in complex genomes with high heterozygosity.
**Corresponding Author:**	Stephen Tsui<br><br>HONG KONG
**Corresponding Author Secondary Information:**	
**Corresponding Author's Institution:**	
**Corresponding Author's Secondary Institution:**	
**First Author:**	Weixue Mu
**First Author Secondary Information:**	
**Order of Authors:**	Weixue Mu
	Joshua Casey Darian
	Wing-Kin Sung
	Xing Guo
	Tuo Yang

Powered by Editorial Manager® and ProduXion Manager® from Aries Systems Corporation

	Mandy Wai Man Tang
	Ziqiang Chen
	Steve Kwan Hok Tong
	Irene Wing Shan Chik
	Robert L Davidson
	Scott C Edmunds
	Tong Wei
	Stephen Kwok Wing Tsui
**Order of Authors Secondary Information:**	
**Additional Information:**	
**Question**	**Response**
Are you submitting this manuscript to a special series or article collection?	No
**Experimental design and statistics**<br><br>Full details of the experimental design and statistical methods used should be given in the Methods section, as detailed in our Minimum Standards Reporting Checklist. Information essential to interpreting the data presented should be made available in the figure legends.<br><br>Have you included all the information requested in your manuscript?	Yes
**Resources**<br><br>A description of all resources used, including antibodies, cell lines, animals and software tools, with enough information to allow them to be uniquely identified, should be included in the Methods section. Authors are strongly encouraged to cite Research Resource Identifiers (RRIDs) for antibodies, model organisms and tools, where possible.<br><br>Have you included the information requested as detailed in our Minimum Standards Reporting Checklist?	Yes
**Availability of data and materials**	Yes

All datasets and code on which the conclusions of the paper rely must be either included in your submission or deposited in publicly available repositories (where available and ethically appropriate), referencing such data using a unique identifier in the references and in the "Availability of Data and Materials" section of your manuscript.<br><br>Have you have met the above requirement as detailed in our Minimum Standards Reporting Checklist?	
GigaScience has policies and guidelines in place for the use of generative AI-writing tools such as ChatGPT. If you have used such writing tools to assist with writing the manuscript this must be declared and cited in the text. Authors should not list AI-writing tools and other AI-assisted technologies as an author or co-author and should acknowledge that they are fully responsible for text generated or refined by AI-writing tools.<p><br><br>A summary of use (particularly in the introduction or among methods) needs to be included at the end of the paper, and the outputs should also be included as a supplementary file hosted in GigaDB or other open repositories. Please read our guidelines for more information. <p><br><br>By submitting to GigaScience, you are aware of the journal's AI-writing tools policy, and if you have declared use of such tools below, you have acknowledged this where appropriate in your manuscript and have made a summary of use and outputs available. <p><br><b>AI-assisted writing tools have been used in the preparation of this manuscript?	No

Powered by Editorial Manager® and ProduXion Manager® from Aries Systems Corporation

Manuscript
Click here to access/download;Manuscript;Bauhinia_manuscript.docx

The Haplotype-resolved T2T Genome for *Bauhinia x blakeana* Sheds Light on the Genetic Basis of Flower Heterosis

Weixue Mu[1,2,†], Joshua Casey Darian[3,†], Wing-Kin Sung[4,5,6,†], Xing Guo[7], Tuo Yang[8], Mandy Wai Man Tang[1], Ziqiang Chen[9], Steve Kwan Hok Tong[10,11], Irene Wing Shan Chik[10], Robert L Davidson[12], Scott C Edmunds[13], Tong Wei[7,*], Stephen Kwok Wing Tsui[1,2,*].

[1] School of Biomedical Sciences, The Chinese University of Hong Kong, Hong Kong

[2] Hong Kong Bioinformatics Centre, The Chinese University of Hong Kong, Hong Kong

[3] School of Computing, National University of Singapore, Singapore

[4] Department of Chemical Pathology, The Chinese University of Hong Kong, Hong Kong

[5] JC STEM Laboratory of Computational Genomics, Li Ka Shing Institute of Health Sciences, The Chinese University of Hong Kong, Hong Kong

[6] Hong Kong Genome Institute, Hong Kong

[7] BGI Research, Wuhan 430074, China

[8] Key Laboratory of Southern Subtropical Plant Diversity, Fairy Lake Botanical Garden, Shenzhen & Chinese Academy of Sciences, Shenzhen, China

[9] National Key Laboratory for Germplasm Innovation & Utilization of Horticultural Crops, College of Horticulture & Forestry Sciences, Huazhong Agricultural

University, Wuhan 430070, China

[10] BGI Genomics, Hong Kong

[11] International DNA Research Centre, Hong Kong

[12] School of Physics, Engineering & Computer Science, University of Hertfordshire, Hatfield, United Kingdom

[13] GigaScience Press, BGI Hong Kong Tech Co. Ltd., Hong Kong

[*] Corresponding authors

Tong Wei, weitong@genomics.cn; Stephen Kwok Wing Tsui, kwtsui@cuhk.edu.hk.

†These authors contributed equally.

Abstract

Background

The Hong Kong Orchid Tree *Bauhinia x blakeana* Dunn has long been proposed to be a sterile interspecific hybrid exhibiting flower heterosis when compared to its likely parental species, *B. purpurea* L. and *B. variegata* L. Here, we report comparative genomic and transcriptomic analyses of the three *Bauhinia* species.

Findings

We generated chromosome-level assemblies for the parental species and applied a trio-binning approach to construct a haplotype-resolved telomere-to-telomere (T2T) genome for *B. blakeana*. Comparative chloroplast genome analysis confirmed *B. purpurea* as the maternal parent. Transcriptome profiling of flower tissues highlighted a closer resemblance of *B. blakeana* to its maternal parent. Differential gene expression analyses revealed distinct expression patterns among the three species, particularly in biosynthetic and metabolic processes. To investigate the genetic basis of flower heterosis observed in *B. blakeana*, we focused on gene expression patterns within pigment biosynthesis-related pathways. High-patent dominance and over-dominance expression patterns were observed, particularly in genes associated with carotenoid biosynthesis. Additionally, allele-specific expression analysis revealed a balanced contribution of maternal and paternal alleles in shaping the gene expression patterns in *B. blakeana*.

Conclusions

Our study offers valuable insights into the genome architecture of hybrid *B. blakeana*,

establishing a comprehensive genomic and transcriptomic resource for future functional genetics research within the *Bauhinia* genus. It also serves as a model for exploring the characteristics of hybrid species using T2T haplotype-resolved genomes, providing a novel approach to understanding genetic interactions and evolutionary mechanisms in complex genomes with high heterozygosity.

Keywords

Bauhinia x blakeana, trio-binning, genome evolution, transcriptome profiling, flower heterosis

Background

Bauhinia x blakeana Dunn, commonly known as the Hong Kong Orchid Tree, is a popular ornamental tree species admired for its striking purplish orchid-like flowers and extended blooming period. Its initial discovery traced back to a chance discovery by French horticulturalist Jean-Marie Delavay on Hong Kong Island in the 1880s, where it was later determined to be completely sterile and grown solely by vegetative propagation [1]. In 1908, due to its distinctive characteristics, it was proposed as a new species [2]. With the species name honoring the former Governor of Hong Kong Sir Henry Blake, it has subsequently been made the emblem of the Hong Kong Special Administrative Region. However, the precise origin of *B. blakeana* remains uncertain and curious due to its sterility. Considering its limited natural occurrence and dependence on artificial cultivation, *B. blakeana* is often regarded as a horticultural

cultivar rather than a naturally existing species. Previous research has supported the hypothesis that *B. blakeana* is a diploid interspecific hybrid resulting from crosses between *Bauhinia purpurea L.* and *Bauhinia variegata L.* Morphological, karyotypic, and molecular investigations including the use of ISSR markers and sequencing of key genetic regions (rbcL, atpB-rbcL intergenic spacer, ITS1), have provided evidence supporting this rare interspecific hybridization event [1, 3-5]. However, these findings lack definitive confirmation, especially at the genomic level. Despite the significant horticultural, cultural and historical value of *B. blakeana*, our understanding of its biology remains limited primarily due to the absence of its genomic information.

Recent advancements in genome sequencing technologies, along with innovative bioinformatic approaches, have revolutionized our capacity to generate high-quality genomes for various plant species, including those with high levels of heterozygosity [6-8]. The availability of these high-quality genomes serves as a foundation for understanding the origin and evolutionary history of plants, as well as unraveling the genetic mechanisms governing essential traits. Additionally, novel methodologies such as high-throughput/resolution chromosome conformation capture (Hi-C) and assembly algorithms like trio-binning have emerged as powerful tools, enabling the construction of haplotype-resolved genomes [9-11]. The historical and cultural interest of Hong Kong *Bauhinia* lead to a community crowdfunded genome project to try to answer some of the questions on the species origin [12], but it only raised enough money to sequence the transcriptomes of the three species [13]. T2T-level assembly completeness and

haplotype-level resolution offers significant advantages in identifying genetic variations, particularly in the study of hybrid heterosis. It allows precise tracking and analysis of genetic variations across parental lines and their hybrid offspring, thereby facilitating a comprehensive understanding of the underlying genetic mechanisms.

Heterosis, also known as hybrid vigor, refers to the phenomenon in which hybrid offspring display enhanced or superior traits compared to their parents. When comparing the flower phenotype of *B. blakeana* to its putative parental species, *B. purpurea* and *B. variegata*, distinct characteristics such as more vibrant flower color, larger flower size, and an extended flowering period are observed, suggesting the presence of heterosis. Heterosis has been extensively studied and utilized in crop breeding [14-16]. However, the genetic basis of this phenomenon remains incompletely understood. Classical hypotheses, including dominance complementation, over-dominance, and epistasis, have been proposed to explain the genetic mechanisms underlying heterosis [15-17]. Transcriptome profiling is commonly employed to investigate heterosis at the transcriptional level, as gene expression plays a pivotal role in linking DNA sequence variation to resulting phenotypic diversity. Several modes of gene expression differences between parents and hybrids have been suggested as contributing factors to heterosis, including additivity/non-additivity, high-/low-parent dominance, and over-/under-dominance [18]. Gene expression is a complex process regulated by a combination of genetic and epigenetic variations, involving the interplay of various genomic elements, including cis-acting elements, trans-acting factors, their

intricate interactions, as well as other epigenomic factors [19, 20]. Furthermore, allele-specific expression (ASE) introduces another layer of complexity to the genetic basis of heterosis [21, 22].

In this study, we presented chromosome-level genome assemblies for the three *Bauhinia* species and employed a trio-binning strategy to reconstruct the high-quality haplotypes of the hybrid *B. blakeana* with gapless T2T completeness. The adoption of T2T genomes has significantly advanced genomics research by providing a detailed depiction of each chromosome from end to end, known as 'telomere-to-telomere'. It enhances our ability to characterize genomic structure and variations, particularly in regions rich in repetitive sequences, providing insights into mechanisms and genomic evolution while elucidating the genetic underpinnings of specific traits. Leveraging our haplotype-resolved T2T genome, through an integrated approach encompassing comparative genomics, transcriptomics, and ASE analyses, we have gained valuable insights into the evolutionary dynamics of *Bauhinia* species and shed light on the genetic basis underlying the intriguing biology of *B. blakeana*. Our haplotype-resolved T2T genome serves as a valuable reference for studying genomes with high heterozygosity, particularly in analyzing the traits of hybrid genomes. It also provides a clear roadmap for future studies, facilitating key discoveries of biosynthetic genes essential for synthetic biology applications.

Results

Sequencing and assembly of the three *Bauhinia* genomes

We incorporated a combination of sequencing technologies including single tube Long Fragment Read (stLFR), BGI-SEQ short-read (whole genome sequencing, or WGS), Oxford Nanopore Technologies long-read (ONT) and Hi-C sequencing methods to obtain high-quality genome assemblies for the three *Bauhinia* species (Supplementary Table S1). We first conducted *k*-mer analyses [23] for all three species to survey their overall genome characteristics. The genome size of *B. purpurea*, *B. variegata* and *B. blakeana* were estimated to be ~303.68 Mb, ~314.49 Mb and ~290.97 Mb, with a heterozygosity ratio of 0.60%, 0.24% and 4.64%, respectively (Supplementary Figure S1).

We performed assembly of the stLFR reads using the Supernova assembler [24] to generate draft genome assemblies for the parental species *B. purpurea* and *B. variegata*. This process yielded two assemblies with genome sizes of approximately 285.15 Mb and 311.01 Mb, respectively, closely matching their estimated genome sizes (Table 1). Subsequently, we utilized Hi-C data to anchor the two initial assemblies onto 14 pseudochromosomes, achieving high anchor rates of 99.98% for both parental species. The resulting assemblies exhibited scaffold N50 values of 21.60 Mb for *B. purpurea* and 24.40 Mb for *B. variegata*. (Table 1; Figure 1A; Supplementary Figure S2) The completeness of the assemblies was assessed using 1614 conserved embryophyte proteins from the BUSCO [26]. The analysis revealed a high level of completeness,

with 97.8% for *B. purpurea* and 98.4% for *B. variegata*, respectively. Furthermore, the high mapping rate (95.72% and 99.29%) and sequencing coverage (94.88% and 94.17%) observed provided further evidence of consistency between the assemblies and the WGS short reads, thereby confirming the high accuracy of our assemblies (Supplementary Table S2).

To address the complexities of the highly heterogeneous genome assembly of *B. blakeana*, we further generated ~17.66 Gb ONT long-reads for genome assembly. Employing a trio-binning approach, we categorized all sequencing reads into three groups: paternal reads, maternal reads, and ambiguous reads. Subsequently, we applied hypo-assembler [25] in haploid mode to assemble each haplotype, with paternal and ambiguous reads, and with maternal and ambiguous reads, respectively. The resulting two sets of high-quality, gap-free haplotypes, hereafter referred to as Hmat and Hpat, represent the maternal and paternal haplotypes of the allodiploid *B. blakeana* genome. Hmat exhibits a genome size of ~275.48 Mb, with a contig N50 value of 19.54 Mb, while Hpat has a size of ~290.70 Mb, with a contig N50 value of 20.99 Mb. (Figure 1B; Table 1). We used Merqury [26] to assess the phasing quality of the two *B. blakeana* haplotypes by comparing *k*-mers from parental read sets to the *k*-mers in each of the haplotype-resolved assemblies. We estimated QV scores of 40.46 for Hmat, 45.64 for Hpat, and 42.39 for the combined set of sequences (Supplementary Table S3A). We counted the number of expected haplotype-specific *k*-mers (hap-mers) present in the corresponding haplotype assemblies and found that the maternal and paternal

haplotypes recovered 92.21% and 95.82% of the expected hap-mers, respectively (Supplementary Table S3B). The maternal haplotype Hmat contains 1.74% of the paternal hap-mers, while the paternal haplotype Hpat contains 1.03% of the maternal hap-mers. These discrepancies are likely due to switch errors or base pair errors. The *k*-mers completeness for Hmat, Hpat and the combine diploid assembly was estimated to be 58.57%, 60.28% and 93.67%, respectively. The result indicates that approximately 40% of the *k*-mers were haplotype-specific, highlighting the high heterozygosity in *B. blakeana* (Supplementary Table S3C). The haplotype evaluation results align with a haplotype-resolved genome assembly, indicating a satisfactory resolution of both haplotypes within the *B. blakeana* genome assembly. We further evaluated the completeness of Hmat and Hpat using BUSCO, resulting in a high completeness score of 99.0% for Hmat and 99.2% for Hpat.

<u>Repeat and gene landscape of the three *Bauhinia* species</u>

Utilizing our four high-quality assemblies, we conducted annotations of repetitive elements and protein-coding genes to examine the repeat and gene landscape of the three *Bauhinia* species. Our analysis revealed varying percentages of repetitive elements in each assembly. Specifically, we found that Hpat contains 32.21% repetitive elements, followed by *B. purpurea* with 27.92%, *B. variegata* with 27.38%, and Hmat with 25.32% (Supplementary Table S4). Among these repetitive elements, LTR retrotransposons were the most prevalent in all four assemblies (Figure 1A, B).

We conducted further predictions and identified 37,804, 37,956, 38,735, and 40,111 protein-coding genes in Hmat, Hpat, *B. purpurea*, and *B. variegata*, respectively. Notably, a high percentage of these genes, 99.98%, 99.97%, 99.98%, and 99.96%, could be functionally annotated against at least one of the six databases searched, namely Nr, SwissProt [27], KEGG [28], KOG [29], TrEMBL [27], and InterPro [30] (Table 1). Moreover, we found that the gene number, gene length, CDS length, exon number, exon length, and intron length showed comparable characteristics across the four assemblies. The predicted gene sets for Hmat, Hpat, *B. purpurea*, and *B. variegata* were evaluated using BUSCO, yielding respective scores of 94.2%, 96.4%, 97.4%, and 97.8%. These results indicate a high level of functional completeness in the annotated proteomes, accurately representing the corresponding genomes.

We further predicted non-coding RNAs (ncRNAs) including miRNA, tRNA, rRNA and snRNA (Supplementary Table S5), as well as transcription factors (TFs), transcription regulators (TRs) and protein kinases (PTKs) in these assemblies (Supplementary Table S6). This comprehensive analysis provides valuable insights into the repetitive elements, protein-coding genes, and regulatory elements present in the genomes of these three *Bauhinia* species.

Structural variations between *B. blakeana* haplotype chromosomes

Structural variation (SV) encompasses a diverse range of genomic alterations, including inversions, translocations, and duplications, which can significantly impact the

organization and structure of the genome. In our study, we investigated SVs in the haplotype chromosomes of *B. blakeana*, which might be associated with its high genome heterozygosity and observed sterility. By utilizing repeat and gene annotations from previous analyses in conjunction with the quarTeT prediction software [31], we identified putative centromeres for each of the 14 pseudochromosomes in both Hmat and Hpat. The centromeres exhibited variable lengths, ranging from 101.60 Kb to 1.48 Mb in Hmat, and from 143.29 Kb to 2.79 Mb in Hpat (Figure 1C; Supplementary Table S7). Additionally, we conducted a search for the presence of the telomere repeat motif "TTTAGGG" along each of the haplotype assembly chromosomes. This allowed us to identify 27 potential telomeric regions in Hmat, with motif repeat numbers ranging from 12 to 848, as well as 27 potential telomeric regions in Hpat, with motif repeats ranging from 31 to 1,117 (Figure 1C; Supplementary Table S8). Notably, except for chromosome 8 in both the Hmat and Hpat assemblies, each chromosome displayed telomeres at both ends, indicating complete reconstruction to a gapless and telomere-to-telomere (T2T) level. Using the SyRI tool [32], we detected a total of 424 SVs between Hmat and Hpat, including 12 inversions (totaling 180.38Kb), 30 translocations (totaling 655.61Kb) and 382 duplications (totaling 3.81Mb) (Figure 1C; Supplementary Table S9). The relatively low number of observed SVs could be attributed to factors such as high synteny between the two parental species of *B. blakeana* or limitations in SV detection methods.

<u>Comparative genomic analysis of *Bauhinia* species</u>

To investigate the relationships and evolutionary history of *Bauhinia* species, we performed comparative genomic analyses involving the *Bauhinia* genomes (*B. purpurea* and *B. variegata*) and 13 other selected representative plant species. The selected species included 9 Fabaceae species from different subfamilies (*S. tora, L. albus, G. max, L. japonicus, M. truncatula, B. purpurea, B. variegata, C. canadensis, C. chinensis*), and 6 other eudicot species (*V. vinifera, C. mollissima, P. persica, P. trichocarpa, A. thaliana, C. canephora*) (Supplementary Table S10). To minimize potential impacts on the results, the sterile hybrid *B. blakeana* was excluded from the evolutionary analyses. Through gene clustering analysis, we identified 17,904 gene families in *B. purpurea* and 18,095 gene families in *B. variegata*. Across all 15 species, we identified 213 single-copy gene families shared among them. Subsequently, a maximum likelihood phylogenetic tree was constructed by combining all the genes within these single-copy gene families (Figure 2A; Supplementary Table S11). The topology of the generated phylogenetic tree was consistent with previous research findings [33]. Molecular dating analysis estimated the divergence of *Bauhinia* genus from the common ancestor with Cercis to have occurred approximately 57.1 million years ago (Mya), followed by the divergence of *B. purpurea* and *B. variegata* around 13.4 Mya.

We used the birth-and-death model to identify expanded and contracted gene families within the selected plant species by comparing them to gene families in their most recent common ancestor (MRCA). *B. purpurea* exhibited 1,138 gene family expansions

and 313 gene family contractions, while *B. variegata* had 1,456 expanded and 284 contracted gene families. In the examination of the entire *Bauhinia* genus, we identified 5,037 expanded gene families and 259 contracted gene families compared to their MRCA (Figure 2A). These 5,037 expanded gene families showed significant enrichment in the KEGG biosynthesis pathways related to bioactive compounds, including monoterpenoid, diterpenoid, flavonoid, terpenoid backbone and carotenoid (Figure 2B, Supplementary Table S12A). Notably, similar enrichment patterns were also observed in the expanded gene families of *B. purpurea* and *B. variegata*. Specifically, in *B. purpurea*, expanded gene families were enriched in KEGG terms such as "plant-pathogen interaction", "Isoflavonoid biosynthesis", "Flavone and flavonol biosynthesis" and “Monoterpenoid biosynthesis”. On the other hand, in *B. variegata*, expanded gene families were enriched in terms such as “Phenylpropanoid biosynthesis", Flavonoid biosynthesis" and "Sesquiterpenoid and triterpenoid biosynthesis" (Supplementary Table S12B-C).

Terpenes and terpenoids encompass a large and diverse group of natural compounds with multiple functions in plants. Terpene synthases (TPSs) are key enzymes responsible for the biosynthesis of terpenoids. These TPS proteins play crucial roles in plant growth, development, and in enhancing resistance to abiotic and biotic stress [34]. To deepen our understanding of terpenoid biosynthesis in *Bauhinia* species, we identified candidate TPSs in the *Bauhinia* species and other selected plants from the Fabaceae family that were used in our previous comparative genomic analyses. We

identified 39 TPS genes in *B. purpurea*, fewer than the 47 TPS genes found in *B. variegata* (Supplementary Table S13A). Within the Fabaceae family, *B. variegata* exhibits the highest TPS gene count, followed by *C. canadensis* (46), *M. truncatula* (41) and *B. purpurea* (39). Subsequently, a phylogenetic tree containing a total of 288 TPS genes across all Fabaceae species was constructed. These TPS genes were categorized into 6 clades, denoted as TPS-a, b, c, e, f, and g, according to the established subfamily classification of TPS genes (Figure 2C) [35]. The TPS-a, b and g collectively comprise the majority of these identified TPS genes. Within the *Bauhinia* species, TPS-b genes emerge as the most prevalent among the TPS genes, with 21 identified in *B. variegata* and 15 in *B. purpurea*, surpassing the TPS-b gene counts observed in all other species within the Fabaceae family. Following TPS-b, TPS-g genes exhibit the second-highest representation in the *Bauhinia* species, with 10 in *B. purpurea* and 9 in *B. variegata*, while TPS-a genes follow with 5 in *B. purpurea* and 7 in *B. variegata*. Notably, TPS-a, TPS-b, and TPS-g constitute clades specific to angiosperms, with TPS-a primarily containing sesquiterpene and diterpene synthases, while TPS-b and TPS-g clade typically encode monoterpene synthases.

To investigate the origin of the increased TPS gene count in the *Bauhinia* species in comparison to other species within the Fabaceae family, we analyzed the duplication events of TPS genes. The results revealed that transpositional duplications primarily drivers contributed to the expansion of TPS-b in *B. purpurea* (7, 46.67%), while proximal repeats (7, 36.84%) and tandem duplication (6, 31.58%) were the major

contributors to the expansion of TPS-b genes in *B. variegata* (Supplementary Table S13B). These expanded TPS-b genes are likely to contribute to the biosynthesis of monoterpenes, consequently enhancing the antimicrobial activity within these *Bauhinia* species.

Confirmation of maternal parent of *B. blakeana* through comparative chloroplast genome analyses

The parthenogenetic inheritance and low substitution rate of the chloroplast (cp) genome make it a valuable tool for phylogenetic analysis and determining hybrid parentage. Using our short-read sequencing data, we successfully assembled and annotated the cp genomes of three *Bauhinia* species. The complete sequences obtained were 156,100 bp for both *B. blakeana* and *B. purpurea*, and 155,415 bp for *B. variegata.* To ensure the accuracy of our assemblies, we performed comparative analyses using ClustalW alignment [36] and mVISTA software [37] to compare our assembled sequences with the previously published cp genomes of *B. blakeana* (MN413506), *B. purpurea* (NC061218), and *B. variegata* (MT176420) (Figure 3A). Our analysis revealed that our assembled *B. blakeana* and *B. variegata* cp genomes matched the published genomes, while a discrepancy was observed in the published *B. purpurea* cp genome. Specifically, a one base pair deletion was detected at the 116,948-base site in the published sequence, which was not present in our assembly (Figure 3B). Importantly, our assembled versions of the three *Bauhinia* cp genomes demonstrated identical sequences for both *B. blakeana* and *B. purpurea*, providing

strong evidence supporting *B. purpurea* as the maternal parent of *B. blakeana*.

To further explore the genetic relationships among the sequenced *Bauhinia* species, we utilized a maximum likelihood (ML) model to construct a phylogenetic tree. The tree included three additional *Bauhinia* species available in the NCBI database, with *C. canadensis* serving as the outgroup. The resulting phylogenetic structure was consistent with previous research, confirming a close genetic relationship among *B. blakeana*, *B. purpurea* and *B. variegata* (Figure 3C) [38].

Transcriptome Profiling of Flower Tissues of the three *Bauhinia* Species

In order to understand the gene expression dynamics among the three *Bauhinia* species, we conducted a comprehensive analysis of differential gene expression in flower tissues. Various DEG analyses were performed, including comparisons between the parental species, comparisons between *B. blakeana* and each of the parental species, as well as comparisons between *B. blakeana* the mid-parent value (MPV) (Figure 4A). To ensure the reliability of our analysis, we collected three biological replicates for each *Bauhinia* species and performed RNA sequencing, generating a substantial amount of sequencing data for each sample (Supplementary Table S1). Initially, the reads of each sample were aligned to the *B. purpurea* genome to generate a read count matrix, which was then used for principal component analysis (PCA). Upon analyzing the PCA results, we observed that one sample, VAR3, exhibited an abnormal location in the PCA plot (Supplementary Figure S3). To

maintain the integrity of the analysis and ensure that this outlier did not influence our results, we excluded the VAR3 sample from subsequent analyses.

To assess pre-existing differential gene expression, we first identified DEGs between *B. purpurea* and *B. variegata*. To avoid false-negative results where the expression level is zero due to the inability to map reads to the reference genome caused by significant genetic differences between the parental genomes, we only selected genes that expressed in both species (with raw counts >= 10) for further analyses. Regardless of whether we used *B. purpurea* or *B. variegata* as the reference, a similar number of DEGs was observed (Figure 4B). Using the *B. purpurea* genome as the reference, we identified a total of 6,988 DEGs, with 3,419 (48.93%) genes up-regulated in *B. purpurea* and 3,569 (51.07%) genes up-regulated in *B. variegata* ($\log_2$|FC (fold change) | > 2; $P < 0.01$). Similarly, when selecting *B. variegata* as the reference, we identified 7,052 DEGs, with 3,435 (48.71%) DEGs exhibiting higher expression levels in *B. purpurea* and 3,617 (51.29%) DEGs showing higher expression levels in *B. variegata* (Figure 4A, B).

To assess the functional implications of these DEGs, we performed KEGG enrichment analysis, and the results were highly consistent regardless of the reference species used (Supplementary Table S14). Specifically, enriched KEGG terms obtained using both reference genomes included "Photosynthesis," "Ribosome," and "Carbon fixation in photosynthetic organisms”, indicating differences in energy production and

metabolism between the two parental species. The term "Circadian rhythm" was also enriched, suggesting possible differences in the regulation of growth and flowering timing between them. Additionally, separate KEGG enrichment analyses were conducted on the up-regulated DEGs in *B. purpurea* and *B. variegata*, respectively. The overlapping results of enriched KEGG terms for up-regulated DEGs in *B. purpurea* included "Inositol phosphate metabolism", "ABC transporters", "Phosphatidylinositol signaling system" and "Circadian rhythm – plant". In contrast, the up-regulated DEGs in *B. variegata* revealed enrichment in "Ribosome", "Photosynthesis", and various metabolic pathways (Figure 4C).

B. blakeana and its parental species display altered transcriptome profiles

Considering the notable phenotypic distinctions between the parental species and *B. blakeana*, our subsequent goal was to evaluate the transcriptome divergence between them, aiming to reveal any possible association with the observed flower heterosis in *B. blakeana*. Using *B. purpurea* as the reference, we identified a total of 5,116 DEGs ($\log_2|FC| > 2$, $P < 0.01$) between *B. blakeana* and *B. purpurea*. Among these DEGs, 2,305 (45.05%) exhibited up-regulated in *B. blakeana*, while 2,811 (54.95%) showed up-regulated in *B. purpurea* (Figure 5A). These DEGs demonstrated significant enrichment in KEGG terms such as "Plant-pathogen interaction", "Plant hormone signal transduction", and various signaling and metabolic pathways. (Supplementary Table S15A). In the comparison between *B. blakeana* and *B. variegata*, we identified a larger set of DEGs, totaling 8,879 genes, using *B. variegata* as the reference. Among

these DEGs, 3,610 (40.20%) exhibited up-regulated in *B. blakeana*, while 5,371 (59.76%) genes showed up-regulated in *B. variegata* (Figure 5A). These DEGs were significantly enriched in KEGG terms including “Photosynthesis”, "Plant hormone signal transduction", as well as various biosynthesis and metabolic pathways (Supplementary Table S15D). Notably, the number of DEGs between *B. blakeana* and *B. variegata* was approximately twice as large as the number of DEGs between *B. blakeana* and *B. purpurea*, suggesting a stronger resemblance in the gene expression profile of *B. blakeana* to its maternal parent, *B. purpurea* (Figure 5A). Additionally, we conducted KEGG enrichment analysis on the DEGs with up-regulated expression levels in each species during the comparisons separately. Interestingly, when comparing *B. blakeana* with *B. purpurea*, there were several KEGG terms enriched in the DEGs with higher expression levels in *B. blakeana* that overlapped with the KEGG terms enriched in DEGs showing higher expression levels in *B. variegata* during the parental species comparison (Figure 5C; Supplementary Table S15B-C, E-F). Similar patterns were also found in the comparison between *B. blakeana* and *B. variegata*, indicating a high-parent dominance in these functions in *B. blakeana*.

To further investigate the gene expression patterns in *B. blakeana*, an additivity analysis was conducted to determine whether they followed an additive model, where the gene expression levels were not significantly different from the average level of parental gene expression, known as the MPV [18]. *B. purpurea* and *B. variegata* were used as references, comparing *B. blakeana* to the MPV, resulting in the identification of 7,111

and 7,287 DEGs, respectively ($log_2|FC| > 1$; $P < 0.01$) (Figure 5B). These MPV-hybrid DEGs were defined as genes with non-additive expression patterns, attributed to allelic interactions that alter regulatory networks and consequently result in gene activity patterns distinct from the average parental values [39]. Among these MPV-hybrid DEGs, 2,607 and 2,717 were up-regulated in *B. blakeana* when using *B. purpurea* and *B. variegata* as references, respectively. KEGG enrichment analysis of these MPV-hybrid DEGs revealed their involvement in energy conversion, utilization, and metabolic transformations. Specifically, pathways such as "Photosynthesis", "Carbon fixation in photosynthetic organisms", "Carbon metabolism", and various metabolic and biosynthetic pathways were significantly enriched (Supplementary Table S16). Importantly, a substantial proportion of genes (67.52%: 14,780 out of a total of 21,891 expressed genes when using *B. purpurea* as a reference, and 67.09%: 14,853 out of total 22,140 expressed genes when using *B. variegata* as a reference) in *B. blakeana* exhibited expression levels that followed an additive model, which can be explained by the combination of gene expression from its parental species. This suggests that while certain genes in *B. blakeana* exhibit non-additive expression patterns, indicating hybrid-specific regulation, a considerable number of genes maintain an additive expression profile, reflecting the balanced contribution of both parental genomes to the gene expression in the hybrid.

Differentially expressed alleles in *B. blakeana*

Expanding upon the discovery of non-additive expression patterns observed in the genes of *B. blakeana*, our study aimed to delve deeper into the underlying molecular mechanisms by identifying genes that exhibit ASE. To accomplish this, we employed two distinct approaches to comprehensively analyze the ASE patterns. Firstly, we utilized RNA-seq reads from the parental species and employed the HyLiTE pipeline [40] to generate a diagnostic SNP table, using the gene set of *B. purpurea* as the reference. Our analysis revealed that 33.86% and 32.65% of the *B. blakeana* RNA-seq reads could be unambiguously assigned to maternal and paternal alleles, respectively (Supplementary Table S17A). We observed no significant bias in the read assignment between the two parental alleles. By utilizing this information, we identified a total of 6,934 genes as ASEGs, among which 3,492 (50.36%) genes exhibited maternal allele dominance, while 3,442 (49.64%) genes displayed paternal allele dominance ($\log_2|FC| > 1$; $P < 0.01$) (Figure 6A). KEGG enrichment analysis was performed on these ASEGs, revealing significant enrichment in KEGG pathways associated with energy generation, transformation, and utilization. Enriched pathways included "Photosynthesis", "Ribosome", “Carbon metabolism” and "Oxidative phosphorylation" (Supplementary Table S17B). Besides the pathways related to energy metabolism, our analysis also identified significant enrichment of KEGG pathways associated with flower color formation, including "Anthocyanin biosynthesis" and "Porphyrin and chlorophyll metabolism". These pathways are known to play important roles in the synthesis and regulation of pigments responsible for flower coloration. Notably, the enrichment analysis of maternal allele dominance ASEGs revealed an enrichment of the

"Carotenoid biosynthesis" pathway (Supplementary Table S17C). Conversely, ASEGs showing paternal allele dominance were enriched in pathways such as "Anthocyanin biosynthesis", "Flavone and flavonol biosynthesis", and "Porphyrin and chlorophyll metabolism" (Supplementary Table S17D).

However, as a portion of the *B. blakeana* RNA-seq reads (33.49%) could not be assigned using the aforementioned method, we employed a genome-wide approach to identify ASEGs. This involved identifying syntenic gene blocks between the two *B. blakeana* haplotypes, Hmat and Hpat, and identifying a total of 10,421 gene pairs within these blocks that exhibited a one-to-one relationship within the same orthogroups, refer to as allele pairs. By mapping the *B. blakeana* RNA-seq reads to the metagenome constructed from the combined gene sets of Hmat and Hpat, we quantified allelic read counts and employed DESeq2 to identify ASEGs on a genome-wide scale, minimizing potential errors associated with a reference-dominated approach. This approach led to the identification of 2,614 ASEGs, with 1,254 showing maternal allele dominance and 1,360 showing paternal allele dominance ($\log_2|FC| > 1$; $P < 0.01$) (Figure 6B). The number of ASEGs with maternal allele dominance and paternal allele dominance was approximately equal, suggesting a balanced influence of both parental alleles and a well-maintained equilibrium in *B. blakeana*. Notably, we observed an interlaced genomic distribution of these ASEGs, with maternal and paternal dominance genes interspersed throughout the genome (Figure 6C). Furthermore, GO enrichment analysis of these ASEGs provided valuable insights into their functional implications. We

observed significant enrichment in several biological processes associated with maintaining genomic stability, responding to DNA damage, and ensuring proper cellular function under stress conditions. Enriched GO categories included "DNA repair", "response to DNA damage stimulus", "cellular response to stress", and "double-strand break repair" (Figure 6D; Supplementary Table S18).

Pigment biosynthesis in *B. blakeana*

B. blakeana exhibits flower heterosis, characterized by significant improvements in various traits compared to its parental species [1]. To investigate the underlying mechanisms associated with flower color formation in *Bauhinia* species, we constructed pigment metabolic pathways, specifically focusing on anthocyanins, carotenoids, and chlorophylls (Figure 7). Initially, we identified orthologous gene groups associated with these pigment metabolic pathways in *B. purpurea*, *B. variegata*, and the two haplotypes of *B. blakeana* (Supplementary Table S19-21). This information also allowed us to investigate the impact of reference genome choice on gene expression analysis. Using the ortholog information, we performed pairwise comparisons of expression values (FPKM: fragments per kilobase of transcript per million mapped reads) for orthologous genes within the same sample. Specifically, we compared the FPKM obtained using two different reference genomes: *B. purpurea* and *B. variegata*. Our findings were consistent with our previous results, as we observed no significant differences in gene expression among all groups when directly comparing the data between groups using Welch's t-test (Supplementary Table S22; Supplementary

Figure S4). However, when we performed the paired t-test, which considers the paired nature of the data within each group, we found no significant differences in gene expression in six out of the total eight samples, regardless of the reference genome chosen (Supplementary Table S23A; Supplementary Figure S5A). To examine whether the expression value of the maternal and paternal alleles add up to the expression value in *B. blakeana* when using *B. purpurea* or *B. variegata* as references, we obtained allelic expression counts of *B. blakeana* using the *B. blakeana* haplotype metagenome as a reference. Our analysis revealed no significant difference among three key expression values of the genes involved in pigment biosynthesis in *B. blakeana*: the sum of allelic expression level when using the haplotype metagenome as a reference; the expression level observed in *B. blakeana* when using *B. purpurea* as a reference; and the expression level observed in *B. blakeana* when using *B. variegata* as a reference (Supplementary Table S23B; Supplementary Figure S5B). This finding indicates that the combined expression value of the maternal and paternal alleles accurately represents the overall expression level in *B. blakeana*, regardless of whether *B. purpurea* or *B. variegata* is used as the reference genome. This consistency in expression levels strengthens the reliability of our analysis and demonstrates that our gene expression assessment effectively captures the contributions of both parental alleles.

We then investigated the copy number differences of metabolism genes within these pathways across four *Bauhinia* assemblies. In general, most genes displayed conserved copy numbers across all four assemblies. However, we observed an interesting

exception concerning the CHI (chalcone isomerase; EC:5.5.1.6) gene, which plays a crucial role in the anthocyanin biosynthesis pathway. Specifically, the *B. purpurea* and *B. blakeana* maternal haplotype exhibited three copies of the CHI gene, while the *B. variegata* and *B. blakeana* paternal haplotype contained four copies of CHI (Figure 7A; Supplementary Table S19). The presence of one less CHI gene in *B. purpurea* was further supported by the absence of RNA-Seq expression counts for its orthologous gene in *B. purpurea* samples when using *B. variegata* as a reference (Supplementary Table S22).

Subsequently, we conducted a comparative transcriptome analysis on these pigment biosynthesis pathways in the three *Bauhinia* species. To facilitate this analysis, we created a new expression matrix by calculating the average FPKM from the FPKM expression data obtained in previous DEG analyses, using *B. purpurea* and *B. variegata* as references (Supplementary Table S24). With this matrix, we examined the expression level dynamics of genes involved in pigment biosynthesis pathways in the parental species and the hybrid *B. blakeana* (Figure 7A-C). Interestingly, we observed that the paternal species, *B. variegata*, exhibited an overall higher expression level of these genes. Next, we summed up the average FPKM values of individual gene copies belonging to the same gene, resulting in a new expression matrix. This matrix allows us to consolidate the expression information and provide a representation measure of gene expression for further analysis (Supplementary Table S25). This approach allows us to capture the overall expression level of each functional gene within the context of

pigment biosynthesis pathways. We identified several genes (22.41%, 13 out of 58) that exhibited clear high-parent dominance, where the expression level differed between the two parents but resembled the higher expressing parent in *B. blakeana* (Figure 7D). Additionally, we observed five genes, including PAL, PDS, CYP97A3, ABA4 and HCAR, showing over-dominance expression patterns in *B. blakeana*. In these cases, *B. blakeana* exhibited higher expression levels compared to both parental species. Notably, these dominance complementation and over-dominance expression patterns were particularly evident in genes involved in carotenoid biosynthesis pathways (47.83%, 11 out of 23). These expression patterns of dominance complementation and over-dominance likely play a role in the elevated expression levels of carotenoid biosynthesis-related genes, thus contributing to the flower color heterosis in *B. blakeana*.

<u>The roles of gene copy expression preference and ASEGs in flower heterosis</u>

Expanding upon our previous examination of gene copy expression differences among the three *Bauhinia* species and overall ASE patterns in *B. blakeana*, our subsequent investigation aimed to delve deeper into the role of gene copy expression preference and ASEGs in flower color heterosis. Firstly, we examined the expression levels of individual gene copies to identify any distinct preferences in gene copy utilization within each specific gene. Our findings revealed variations in the expression levels of specific gene copies among the three *Bauhinia* species, indicating the presence of species-specific expression patterns. For instance, the DXR gene (1-Deoxy-D-xylulose-

5-phosphate reductoisomerase; EC:1.1.1.267), a key enzyme in the MEP pathway [41, 42], exhibited over-dominance expression in *B. blakeana*. Between total two copies of the DXR gene, DXR_2 was consistently favored and exhibiting higher expression levels in all three *Bauhinia* species (Supplementary Table S24). On the other hand, the PAL gene (Phenylalanine ammonia-lyase; EC:4.3.1.24), a crucial enzyme involved in plant metabolism responsible for the first step in the biosynthesis of various natural products containing the phenylpropane skeleton [43], also exhibited over-dominance expression in *B. blakeana*. However, this over-dominance pattern was not consistently observed across all four PAL gene copies. Among the examined gene copies, PAL_3 consistently demonstrated the highest expression levels in both *B. blakeana* and *B. variegata*, while *B. purpurea* specifically exhibited the highest expression level in PAL_4. The observed over-dominance in the PAL gene of *B. blakeana* was attributed to the elevated expression level of PAL_3 (Supplementary Table S24). Furthermore, variations in expression preferences were also observed in other genes within the pigment biosynthesis pathways, highlighting the presence of species-specific expression patterns that likely contribute to the character specialization observed within each *Bauhinia* species.

Motivated by the observed variations in gene copy utilization preferences and expression levels differences among the *Bauhinia* species, we further investigated the ASE patterns of each of these genes to determine whether the alleles in *B. blakeana* inherited the expression patterns corresponding to those of the parental species. To

evaluate ASE, we calculated the ASE ratio by dividing the maternal allele expression by the sum of maternal and paternal allele expressions. A significant ASE ratio was defined as values greater than 0.7 or less than 0.3 in at least two out of three *B. blakeana* samples. Genes exhibiting significant ASE ratios, indicating distinct allele-specific expression, were classified as ASEGs. Within the anthocyanin biosynthesis pathway, we identified 10 genes with maternal allele dominance and 7 genes with paternal allele dominance out of the total 27 genes analyzed (Figure 7A-C; Supplementary Table S26). In the carotenoid biosynthesis pathway, we found 9 genes with maternal allele dominance and 12 genes with paternal allele dominance out of the total 44 genes analyzed. Similarly, within the chlorophyll biosynthesis pathway, we detected 5 genes with maternal allele dominance and 13 genes with paternal allele dominance out of the total 60 genes analyzed. The proportions of ASEGs were 62.96% in the anthocyanin pathway, 47.73% in the carotenoid pathway, and 30.00% in the chlorophyll pathway. Although the majority (85.71%, 48 out of 56) of the identified ASEGs in *B. blakeana* exhibited parental allelic dominance biased towards the parent with a higher expression level, there are instances where the allelic dominance did not strictly correspond to the expression patterns of the parental species. This observation suggests that additional factors beyond the expression levels or *cis*-regulation of the parental species also play a role in regulating gene expression and establishing allelic dominance in *B. blakeana*. These factors, such as trans-regulatory elements, epigenetic modifications, or genetic interactions, may also contribute to shaping the observed expression patterns in *B. blakeana*.

Discussion

In this study, we successfully generated chromosome-level genome assemblies for two parental species, *B. purpurea* and *B. variegata*, as well as haplotype-resolved gapless genome assemblies for the hybrid *B. blakeana*. The utilization of the trio-binning assembly strategy, taking advantage of the high heterozygosity in the *B. blakeana* genome, enabled us to overcome the challenges posed by heterozygosity and obtain high-quality genome assemblies for further analyses. The haplotype-resolved genome assemblies served as a solid foundation for our extensive downstream investigations, offering prospects for delving into the complex characteristics of the heterogeneous *B. blakeana* genome and uncovering deeper insights into its biology. Furthermore, by obtaining the cp genomes of all three *Bauhinia* species, we were able to confirm *B. purpurea* as the maternal parent of *B. blakeana* through comparative cp genome analyses, as well as confirming a close phylogenetic relationship between *B. blakeana* and *B. variegata*.

Utilizing the high-quality genome assemblies, our subsequent comparative genomic analysis uncovered several gene families associated with terpenoid and flavonoid biosynthesis that have undergone expansions during the evolutionary diversification of the *Bauhinia* genus. Additionally, notable expansions were also observed within the TPS gene family of *Bauhinia* species when compared to other members in the Fabaceae family. Terpenes, commonly released by plants in response to insect herbivory, are primarily derived from the five-carbon precursor, isopentenyl diphosphate (IPP). These

compounds are synthesized through two distinct pathways within the plant cell: the mevalonate (MVA) pathway in the cytosol and the 2C-methyl erythritol 4-phosphate (MEP) pathway in plastids. The specific terpene synthase enzymes play a crucial role in determining the structure of the terpenes produced [44]. The expansion of TPS genes in *Bauhinia* species has the potential to confer heightened resistance to pathogens, establishing a more robust defense mechanism against a diverse range of microbial invaders. Terpenoids and flavonoids are major classes of secondary metabolites that exhibit a variety of pharmacological bioactivity, including anti-microbial, anti-inflammatory, anti-diabetic, and anti-cancer effects. The genus *Bauhinia* has a long history of usage in herbal medicine for treating conditions such as malaria, diarrhea, diabetes, and various other health conditions. Specifically, *B. purpurea* and *B. variegata* have been extensively used in traditional medicine and have been extensively investigated for their medicinal properties [45-48]. The expansion of TPS family genes, particularly TPS-b genes, likely contributes to the abundant terpenoid content, thereby underpinning the observed medicinal properties of these species. We also observed enrichment of the KEGG term 'Cutin, suberine, and wax biosynthesis' within the expanded gene families of the *Bauhinia* genus, potentially explaining the unique leaf characteristics of *B. blakeana*, characterized by cells and epicuticular wax arranged in a regular pattern, leading to its limited dust-catching capacity [49].

B. blakeana exhibits flower heterosis characterized by its large, showy, and vibrant magenta-colored flowers resembling orchids. Despite its sterile nature, *B. blakeana* has

gained popularity as an ornamental species worldwide, mainly due to its unique floral display and extended flowering period. Therefore, our study aimed to investigate the transcriptome profiles of flower tissues and the genetic mechanisms contributing to the observed phenotypic variation among the three *Bauhinia* species, with a specific focus on studying the flower color heterosis in *B. blakeana*. Specifically, *B. purpurea* displayed much paler coloration compared to *B. variegata*. Through further comparing the transcriptome profiles between *B. blakeana* and its parental species, we found that even though *B. blakeana* exhibits flower color that is more similar to its paternal parent, the general gene expression profile of *B. blakeana* aligns more closely with its maternal parent. We observed that the number of genes exhibiting up-regulated expression in *B. blakeana* is comparatively lower than the number of genes showing down-regulated expression when compared to the MPV. This observation suggests a potential trade-off, wherein *B. blakeana* may have sacrificed certain functional attributes in favor of achieving flower color traits and prolonged flowering period [50, 51].

There are two main classical hypotheses that aim to explain the mechanisms underlying heterosis: dominance and over-dominance hypothesis [52, 53]. The dominance hypothesis focuses on the significance of dominant alleles, while the over-dominance hypothesis emphasizes the advantages of heterozygosity. These two hypotheses are not mutually exclusive, as both mechanisms may contribute to heterosis. To investigate the genetic mechanisms underlying flower color heterosis in *B. blakeana*, we conducted analyses of gene expression patterns involved in pigment biosynthesis pathways. We

found 31.03% of these genes exhibited dominance complementation or over-dominance expression patterns. Specifically, within the subset of genes related to carotenoid biosynthesis pathways, approximately half of these genes (47.83%) displayed such expression patterns. Interestingly, upon checking, genes related to carotenoid biosynthesis pathways were also identified exhibiting significant differential expression levels ($log_2|FC| > 4$; $P < 0.01$) between the parental species. The pronounced variations in expression levels observed within the carotenoid biosynthesis pathways between the parental species may be associated with the higher proportion of genes exhibiting dominance complementation or over-dominance expression patterns in *B. blakeana*, likely to play a significant role driving the observed flower color traits.

ASE is another mechanism that has been suggested to contribute to heterosis [54-56]. We employed two distinct approaches to conduct genome-wide analyses of ASE in flower tissues of *B. blakeana*. Although there were variations in the total number of identified ASEGs between the two methods, we observed a balance in both the number and level of ASEGs biased towards each parental allele within each method. Despite the limitations of both approaches used, they yielded valuable insights into the ASE landscape within the *B. blakeana* genome, highlighting the equitable participation of maternal and paternal alleles in shaping the observed ASE patterns. Through our in-depth analysis of pigment biosynthesis-related genes in *B. blakeana*, we discovered that the ASE patterns demonstrate a preference for the parental allele linked to higher expression levels in the comparison between the parental species. The carotenoid

biosynthesis pathway displayed the highest proportion of ASEGs, consistent with the higher proportion of genes exhibiting dominance complementation or over-dominance expression patterns in this pathway. These findings emphasize the critical role of genetic regulation and interactions within the carotenoid pathway in driving the observed enhancements in flower color in *B. blakeana*.

Overall, our study provides comprehensive genomic and transcriptomic insights into the biology of *B. blakeana*. Through the utilization our *de novo* assembled haplotype-resolved and gapless T2T genome, we have advanced our understanding of the genomic structure and genetic mechanisms underlying the captivating flower color trait in this popular ornamental hybrid tree species, serving as a case study for investigating traits in hybrid species. Furthermore, the resources generated in this study lay the foundation for future genetic studies, breeding programs, and conservation initiatives in *Bauhinia* species.

Methods

Plant sampling, library preparation, and sequencing

Fresh leaves of three *Bauhinia* species, namely *Bauhinia x blakeana Dunn* (NCBI: txid180222), *Bauhinia purpurea L.* (NCBI: txid3806) and *Bauhinia variegata L.* (NCBI: txid167791), were collected from Shenzhen, Guangdong Province, China. To perform whole-genome sequencing on all three species, high-molecular-weight (HMW) genomic DNA were extracted using a modified CTAB (cetyltrimethylammonium

bromide) method [57]. The extracted DNA from each species was used to prepare Single-tube Long Fragment Read (stLFR) libraries [58], and WGS short-read libraries, following the respective protocols. Hi-C libraries were constructed for each species using the MboI enzyme and following the standard Hi-C library preparation protocol [59]. These libraries were subsequently sequenced on the BGIseq500 platform to generate pair-end reads with an insert size of ~250bp [60]. In addition, we prepared an extra ONT library for the hybrid species B. blakeana using the LSK108 kit (SQK-LSK108, Oxford), which was then sequenced on the Nanopore MinION sequencer [61].

To perform transcriptome sequencing, we collected three fully blossomed flower tissues from each individual of the three sequenced *Bauhinia* species. Total RNA was isolated using the TIANGEN Kit with DNase I and processed using the NEBNextUltra^{TM} RNA Library Prep Kit to create a pair-end library with a 250 bp insert size for each sample. The libraries were then barcoded and pooled together as an input for downstream sequencing on the BGI-DIPSEQ platform.

<u>Genome size estimation</u>

Previous studies have shown that the three *Bauhinia* species share the same chromosome number (2n=28) [4]. To estimate the genome size of each species, we performed *k*-mer analysis. First, the raw WGS short-reads were first filtered according to the sequencing quality with Trimmomatic (RRID:SCR_011848) (v0.40) with "ILLUMINACLIP:adapter.fa:2:30:10 HEADCROP:5 LEADING:3 TRAILING:3

SLIDINGWINDOW:5:15 MINLEN:95” parameter [62]. Next, *k*-mer frequencies were counted by Jellyfish (RRID:SCR_005491) (v2.2.6) with a *k*-value of 21 using the clean WGS reads [63]. Based on the 21-mer frequency distribution analysis with GenomeScope (RRID:SCR_017014) [64], we estimated the genome size of *B. purpurea*, *B. variegata* and *B. blakeana* to be ~303.68 Mb, ~314.49 Mb and ~290.97 Mb, respectively. Notably, the estimated genome size of *B. variegata* was close to the previously published genome size of 326.4Mb [33].

Genome assembly and quality control

To generate draft assemblies for the parental species *B. purpurea* and *B. variegata*, we performed *de novo* assembly using the Supernova assembler (RRID:SCR_016756) (v2.1.1) with the “--max reads 2140000000” parameter for each species using the stLFR reads [65]. Next, we used the clean WGS short-read data of each species to fill gaps in the draft assemblies using the GapCloser (RRID:SCR_015026) with default parameters. To further improve the assembly contiguity, we utilized Hi-C data from each parental species. We aligned the Hi-C data to the draft assemblies using BWA (RRID:SCR_010910) -MEM [66] and then integrated the assemblies from contig-level into pseudochromosome-level using ALLHiC (RRID:SCR_022750) [11]. Specifically, we used the bam files resulting from the alignment to assign contigs into a pre-defined number of groups (14 groups in our research), and unplaced contigs were assigned into partitioned clusters. Finally, we reordered and oriented each group to optimize the result and generate the fasta format sequences and agp location files. We evaluated the

genome scaffolding by plotting the chromatin contact matrix.

To assemble the hybrid offspring *B. blakeana*, we employed a trio-binning strategy to generate two fully phased haplotype assemblies. Firstly, we identified solid *k*-mers, which are *k*-mers that are unique in the three *Bauhinia* genomes. This yielded three sets of solid *k*-mers, for the hybrid, paternal, and maternal sequencing data. Subsequently, we defined paternal and maternal hap-mers. Paternal hap-mers are the intersection between the paternal and hybrid solid *k*-mers, while maternal hap-mers are the intersection between the maternal and hybrid solid *k*-mers. This definition is similar to the concept of hap-mers used in Merqury but adjusted to accommodate our solid *k*-mers. We proceeded to assemble the two haplotypes of *B. blakeana* separately. First, we categorized all reads into three groups: paternal reads, maternal reads, and ambiguous reads. Reads that exclusively contained paternal hap-mers as their solid *k*-mers were classified as paternal reads, and the same principle applied to maternal reads. Reads containing both types of hap-mers or neither were labeled as ambiguous. We then ran the hypo-assembler in haploid mode for each haplotype, once with paternal and ambiguous reads, and once with maternal and ambiguous reads. This approach resulted in two distinct yet more accurate assemblies. Following this, haplotype-specific Hi-C reads were aligned to their respective draft assemblies for scaffolding based on contact frequency. Subsequently, we manually clustered the remaining long reads from the previous steps and assembled them. This newly assembled set of contigs was used for gap-filling purposes. After completing the aforementioned steps, the majority of the

genome was resolved. However, not all the telomeres are fully assembled. To address this, we identified reads displaying a high abundance of telomere signals that were not utilized in the initial assembly. Subsequently, we manually clustered these reads based on their SNPs in comparison with the existing contig terminals, and then assigned the clusters to their respective terminal positions.

The genome completeness was evaluated by BUSCO (RRID:SCR_015008) using the embryophyta_odb10 database [67]. The genome continuity was evaluated by calculating contig N50 length. The accuracy of the genome was evaluated by mapping the WGS sequencing data to the genome with BWA-MEM and calculating mapping rate and coverages with SAMTOOLS (RRID:SCR_002105) [68]. To further assess the two haplotype genomes of *B. blakeana*, we used Merqury (RRID:SCR_022964) [26] to evaluate the haplotype-specific accuracy, completeness, and phase block continuity base on the trio information.

Identification of repetitive elements

To identify repetitive elements in our assembled genomes, we employed a combination of homology-based and *de novo* prediction methods following the Repeat Library Construction-Advanced pipeline [69]. Firstly, we employed RepeatMasker (RRID:SCR_012954) [70] and RepeatProteinMasker to identify transposable elements (TEs) based on similarity-based comparisons to search for known repeat sequences with Repbase (RRID:SCR_021169) [71]. In addition, we used LTR_Finder

(RRID:SCR_015247) [72] to search for LTR retrotransposons *de novo*. The resulting repetitive sequence libraries were then integrated using RepeatModeler (RRID:SCR_015027) [73] to create a complete and non-redundant custom library, which served as input for RepeatMasker to identify and classify TEs in the genome assemblies. Furthermore, we searched for tandem repeats across the genomes using Tandem Repeats Finder (RRID:SCR_022193) [74]. All identified repeats were used to soft mask the genome assemblies with RepeatMasker prior to gene structure prediction.

Protein-coding gene prediction and functional annotation

We utilized a combination of *ab initio*, homology-based, and RNA-seq-based approaches with the BRAKER2 (RRID:SCR_018964) pipeline [75] to predict the protein-coding gene set in our assembled genomes. To begin, we obtained and assembled the publicly available leaf transcriptome data for each species from the crowdfunded *Bauhinia* Genome project [13]. The leaf data were then aligned to the corresponding genomes using HISAT2 (RRID:SCR_015530) (v2.1.0) [76] with "--max-intronlen 500000 --sensitive --dta --dta-cufflinks --phred33 --no-discordant --no-mixed" parameters, and the resulting BAM files were sorted using SAMTOOLS. We used the BAM files, along with the OrthoDB (RRID:SCR_011980) v10.1 protein database [77] (the published *B. variegata* proteins were added), as input for BRAKER2 with "--softmasking --etpmode". We further filtered the predicted gene sets to remove any translated proteins less than 30 amino acids in length or with in-frame stop codons. Finally, we evaluated the completeness of the gene sets using BUSCO with the

embryophyta_odb10 database.

We used two methods to infer the functions of our predicted genes. First, we performed a BLASTP (RRID:SCR_001010) homolog search against public protein databases such as UniProtKB/Swiss-Prot (RRID:SCR_021164), TrEMBL, NCBI non-redundant (NR), and KEGG (RRID:SCR_012773). Second, we utilized InterProScan (RRID:SCR_005829) to search for conserved amino acid sequences, motifs, and domains by comparing the sequences against domain databases including Pfam (RRID:SCR_004726), PANTHER (RRID:SCR_004869), PRINTS (RRID:SCR_003412), PROSITE (RRID:SCR_003457), ProDom (RRID:SCR_006969), and SMART (RRID:SCR_005026).

Identification of structural variations, centromeres, and telomeres

The Nucmer alignment tool from the MUMmer (RRID:SCR_018171) [78] was used for conducting whole-genome alignments. Nucmer was executed with the -maxmatch option to retrieve all alignments between the *B. blakeana* allelic chromosomes, with parameters -c 500, -b 500, and -l 100. Subsequently, the delta-filter and show-coords subprograms were employed to filter the alignments and convert them into tab-delimited files. Lastly, SyRI (RRID:SCR_023008) [32] was used to detect inversions, translocations and duplications.

CentroMiner from the quarTeT prediction software (RRID:SCR_025258) [31] was

employed for centromere identification. To enhance its performance, the repeat and gene annotations obtained from previous analyses were added as input. The resulting predictions underwent a manual selection process to ensure accuracy and reliability before finalization. TeloExplorer from quarTeT was used for telomere identification by searching for the characteristic motif (TTTAGGG).

Identification of non-coding RNAs

In addition to protein-coding genes, we also identified ncRNAs within our assembled genomes. We used tRNAscan-SE (RRID:SCR_008637) [79] to identify tRNA genes, and BLASTN (RRID:SCR_001598) to search for rRNA genes by comparing the rRNA sequences of *Arabidopsis thaliana* and *Oryza sativa* against each of the three *Bauhinia* assemblies. We predicted miRNAs and snRNAs by searching the sequences against the Rfam (RRID:SCR_007891) database using Infernal (RRID:SCR_011809) [80].

Identification of transcription factors

We identified and classified transcription factors (TFs), transcription regulators (TRs), and protein kinases (PTKs) among our predicted gene models into different families using the online tool iTAK pipeline (http://bioinfo.bti.cornell.edu/cgi-bin/itak/index.cgi) with default parameters.

Phylogenetic analysis and divergence time estimation

Single copy genes from 15 selected plants were identified using OrthoFinder

(RRID:SCR_017118) [81] and subsequently used to construct the phylogenetic tree, following these steps: 1. For each single-copy gene orthogroup data set, we performed multiple amino acid sequence alignments using MAFFT (RRID:SCR_011811) (v.7.310) [82], followed by gap position removal using Gblocks (RRID:SCR_015945) (v.0.91b) (positions where 50% or more of the sequences have a gap were removed) [83]. 2. We used the maximum-likelihood (ML) software IQ-TREE (RRID:SCR_017254) (v 1.6.1) [84] to reconstruct the phylogenetic tree for each single-copy gene family. 3. The gene trees of each data set were then analyzed using ASTRAL (RRID:SCR_024520) (v.5.5.9) [85] to infer the species tree with quartet scores and posterior probabilities. 4. The sequences generated from step one was also concatenated as a single supermatrix and a concatenation tree was generated using RAxML (RRID:SCR_006086) [86].

We used the MCMCTree program in PAML package (RRID:SCR_014932) (v4.5) [87] to estimate the divergence time of each tree node, based on the estimated divergence times of the following nodes from TimeTree website (http://www.timetree.org): *C. canephora* – *V. vinifera* (111.4-123.9 MYA), *A. thaliana* - *V. vinifera* (111.24-117.56 MYA), *A. thaliana* - *M. truncatula* (102-112.5 MYA) and *A. thaliana* - *P. trichocarpa* (107-109 MYA). To perform this analysis, we used the sequential PHYLIP format nucleotide sequences and rooted phylogenetic tree derived from the result of the gene family analysis as inputs for MCMCTree.

We used CAFE (v2.1) [88] to infer the expansion and contraction of gene families based

on the phylogenetic analysis and divergence time. The input tree for CAFE was the species tree constructed by ASTRAL. For each gene family that was significantly expanded or contracted (P-value < 0.05), we inferred functional information based on the functional annotation results. KEGG and GO enrichment analyses of genes were conducted using an enrichment pipeline (https://sourceforge.net/projects/enrichmentpipeline/) (parameter setting: *p* Adjust Method: fdr; TestMethod: FisherChiSquare).

<u>Assembly of chloroplast genome and phylogenetic analysis of chloroplast genes</u>

The chloroplast genome (cp) of the three *Bauhinia* species were assembled using the clean WGS short-read data in GetOrganelle (RRID:SCR_022963) [89], and further annotated using CpGAVAS2 [90]. We obtained additional available *Bauhinia* cp genomes from the NCBI database, including *B. binate* (NC_037764.1), *B. brachycarpa* (NC_037762.1), *B. racemosa* (ON456405.1). *C. cancadensis* (KF856619.1) from the Cercis genus was also obtained to serve as outgroup. To construct the phylogenetic tree, a total of 77 protein-coding genes were aligned and trimmed following the same pipeline used for nuclear tree and the phylogenetic tree was built by RAxML with "-f a -#1000 -m PROTGAMMAJTT" parameters. In addition, we obtained previously published chloroplast genomes of *B. blakeana* (MN413506.1), *B. purpurea* (NC_061218.1), and *B. variegata* (MT176420) from the NCBI database for comparison with our assembled genomes using mVISTA (https://genome.lbl.gov/vista/mvista/instructions.shtml).

RNA-seq data analysis and ASE gene identification

RNA-seq sequencing data were trimmed using Trimmomatic to remove low-quality bases and adapter sequences. Clean reads of all three species were mapped to the selected reference genome using Bowtie 2 (RRID:SCR_016368) and the counts and FPKM value was calculated by eXpress program [91], which was incorporated in the Trinity (RRID:SCR_013048) package. DEGs were identified based on the counts using DESeq2 (RRID:SCR_015687) [92].

We used two different methods to identify alleles between *B. blakeana* maternal haplotype and paternal haplotype:

1. Syntenic gene blocks between *B. blakeana* maternal haplotype and paternal haplotype were identified using BLASTP and MCScanX (RRID:SCR_022067) [93] with annotations and protein sequences. Genes from the same orthogroup of the two haplotypes were identified using OrthoFinder. Gene pairs belonging to the same orthogroup and located in large syntenic blocks were identified as alleles. The assemblies and annotations of both haplotypes were then combined to construct a metagenome. Clean RNA-seq reads of *B. blakeana* were mapped to the metagenome using Bowtie2 by retaining the best alignment. FPKM and counts were calculated using the eXpress program. To screen for ASEGs, we employed DESeq2 using the allelic read count data.

2. Clean RNA-seq reads were processed by HyLiTE to produce tables of parental and allelic expression data in a single step. First, RNA-seq reads of all three species were mapped against the *B. purpurea* genome using Bowtie2. Next, all the BAM files were proceeded to SAMtools to generated the .pileup file. HyLiTE detected the SNPs that diagnostic of the two parental species and used it to determine the parental origin of the reads from the hybrid. The final output of HyLiTE contained the read count tables of total and allelic gene expression in the hybrid and parental accessions. We manually built the expression matrix by combining the output allelic gene expression tables of the three *B. blakeana* biological replicates for downstream DEG analysis. ASE genes were identified based on the counts using DESeq2.

Identification of flower pigmentation genes

To elucidate the mechanisms underlying flower pigmentation, we focused on the metabolism and accumulation of flavonols, anthocyanins, carotenoids and chlorophylls. Initially, we constructed the metabolic pathways associated with these compounds. For reference, we downloaded gene sequences encoding enzymes involved in these pathways from UniProt (RRID:SCR_002380). These reference sequences served as a basis for identifying corresponding genes in our assemblies. Our candidate gene selection process involved the following criteria: 1. Candidate gene sequences were identified through BLASTP searches using a cut-off *E*-value of 1e−05, comparing them to the query gene sequences we obtained. 2. Functional annotations of the candidate genes were manually inspected to ensure similarity to the query genes. 3. Following the

initial identification, the candidate genes underwent further verification by constructing phylogenetic trees. The maximum likelihood trees were constructed using IQTREE after aligning the sequences with MAFFT.

Data Availability

The raw genomic and flower tissue transcriptomic data, genome assemblies and annotations have been deposited to the China National GeneBank (CNGB) Sequence Archive (CNSA) under accession number CNP0001583 and CNP0006215. The leaf transcriptome data can be accessed on NCBI (https://www.ncbi.nlm.nih.gov/) under the BioProject: PRJEB21302 [13].

Abbreviations

T2T: telomere-to-telomere; Hi-C: high-throughput/resolution chromosome conformation capture; ASE: allele-specific expression; stLFR: Single-tube Long Fragment Read; WGS: Whole Genome Sequencing; ONT: Oxford Nanopore Technologies; SV: structural variation; CHI: chalcone isomerase; FPKM: fragments per kilobase of transcript per million mapped reads; MPV: mid-parent value; TPS: Terpene synthases.

Consent for publication

All the authors approved the manuscript and gave their consent for submission and publication.

Competing Interests

The authors declare that they have no competing interests.

Authors' Contributions

S.K.W.T. and T.W. conceived and supervised the research. Z.C., K.H.S.T, R.L.D and S.C.E collected the samples and performed the experiments. W.M., J.C.D, W.K.S., X.G., T.Y., M.W.M.T and W.S.C analyzed the data. W.M. and J.C.D wrote the manuscript. W.K.S, X.G, S.C.E, T.W and S.K.W.T revised the manuscript. All authors have read and approved the final manuscript.

Acknowledgements

This work was supported by the by the Collaborative Research Fund of the Research Grants Council (C4049-23EF), China National GeneBank (CNGB), Key Laboratory of Genomics, Ministry of Agriculture, and Guangdong Provincial Key Laboratory of core collection of crop genetic resources research and application.

References

1. Lau CP, Ramsden L, Saunders RM: **Hybrid origin of "Bauhinia blakeana" (Leguminosae: Caesalpinioideae), inferred using morphological, reproductive, and molecular data.** *Am J Bot* 2005, **92:**525-533.
2. Dunn S: **New Chinese plants.** *Journal of Botany* 1908, **46:**324-326.
3. Mak CY, Cheung KS, Yip PY, Kwan HS: **Molecular evidence for the hybrid origin of Bauhinia blakeana (Caesalpinioideae).** *J Integr Plant Biol* 2008, **50:**111-118.
4. Sharma AK, Raju DT: **Structure and behaviour of chromosomes in Bauhinia and allied genera.** *Cytologia* 1968, **33:**411-426.
5. Yuping L, Yingxiong Q, Chan YSG: **Dentification of Three Species in Bauhinia and Hybrid Origin of Bauhinia blakeana Using ISSR Markers.** *Acta Horticulturae Sinica* 2006, **33:**433.
6. Satam H, Joshi K, Mangrolia U, Waghoo S, Zaidi G, Rawool S, Thakare RP, Banday S, Mishra AK, Das G, Malonia SK: **Next-Generation Sequencing Technology: Current Trends and Advancements.** *Biology (Basel)* 2023, **12**.
7. Michael TP, VanBuren R: **Building near-complete plant genomes.** *Curr Opin Plant Biol* 2020, **54:**26-33.
8. Kress WJ, Soltis DE, Kersey PJ, Wegrzyn JL, Leebens-Mack JH, Gostel MR, Liu X, Soltis PS: **Green plant genomes: What we know in an era of rapidly expanding opportunities.** *Proc Natl Acad Sci U S A* 2022, **119**.
9. Garg S, Fungtammasan A, Carroll A, Chou M, Schmitt A, Zhou X, Mac S,

Peluso P, Hatas E, Ghurye J: **Accurate chromosome-scale haplotype-resolved assembly of human genomes.** *BioRxiv* 2019**:**810341.

10. Koren S, Rhie A, Walenz BP, Dilthey AT, Bickhart DM, Kingan SB, Hiendleder S, Williams JL, Smith TP, Phillippy AM: **De novo assembly of haplotype-resolved genomes with trio binning.** *Nature biotechnology* 2018, **36:**1174-1182.
11. Zhang X, Zhang S, Zhao Q, Ming R, Tang H: **Assembly of allele-aware, chromosomal-scale autopolyploid genomes based on Hi-C data.** *Nature Plants* 2019, **5:**833-845.
12. S. L: **Unique project to sequence the genome of the Hong Kong bauhinia tree.** SCMP; 2015.
13. KW TWKJBGcT: **Transcriptome assemblies of three Bauhinia species.**: GigaScience Database; 2018.
14. Huang X, Yang S, Gong J, Zhao Q, Feng Q, Zhan Q, Zhao Y, Li W, Cheng B, Xia J, et al: **Genomic architecture of heterosis for yield traits in rice.** *Nature* 2016, **537:**629-633.
15. Baranwal VK, Mikkilineni V, Zehr UB, Tyagi AK, Kapoor S: **Heterosis: emerging ideas about hybrid vigour.** *Journal of Experimental Botany* 2012, **63:**6309-6314.
16. Chen ZJ: **Genomic and epigenetic insights into the molecular bases of heterosis.** *Nature Reviews Genetics* 2013, **14:**471-482.
17. Hochholdinger F, Baldauf JA: **Heterosis in plants.** *Current Biology* 2018,

28:R1089-R1092.

18. Swanson-Wagner RA, Jia Y, DeCook R, Borsuk LA, Nettleton D, Schnable PS: **All possible modes of gene action are observed in a global comparison of gene expression in a maize F**

hybrid and its inbred parents. *Proceedings of the National Academy of Sciences of the United States of America* 2006, **103:**6805-6810.

19. Ma XA-O, Xing F, Jia Q, Zhang Q, Hu T, Wu B, Shao LA-O, Zhao YA-O, Zhang QA-O, Zhou DA-O: **Parental variation in CHG methylation is associated with allelic-specific expression in elite hybrid rice.**

20. Li DA-O, Lu XA-O, Zhu YA-OX, Pan JA-O, Zhou SA-O, Zhang XA-O, Zhu GA-O, Shang YA-O, Huang SA-O, Zhang CA-O: **The multi-omics basis of potato heterosis.**

21. Springer NM, Stupar RM: **Allele-specific expression patterns reveal biases and embryo-specific parent-of-origin effects in hybrid maize.** *Plant Cell* 2007, **19:**2391-2402.

22. Shao L, Xing F, Xu CH, Zhang QH, Che J, Wang XM, Song JM, Li XH, Xiao JH, Chen LL, et al: **Patterns of genome-wide allele-specific expression in hybrid rice and the implications on the genetic basis of heterosis.** *Proceedings of the National Academy of Sciences of the United States of America* 2019, **116:**5653-5658.

23. Chikhi R, Medvedev P: **Informed and automated k-mer size selection for genome assembly.** *Bioinformatics* 2014, **30:**31-37.

24. Weisenfeld NI, Kumar V, Shah P, Church DM, Jaffe DB: **Direct determination of diploid genome sequences.** *Genome Res* 2017, **27:**757-767.

25. **Creating diploid assemblies from Nanopore and Illumina reads with hypo-assembler.** *Nat Methods* 2024, **21:**560-561.

26. Rhie A, Walenz BP, Koren S, Phillippy AM: **Merqury: reference-free quality and phasing assessment for genome assemblies.**

27. Boeckmann B, Bairoch A, Apweiler R, Blatter MC, Estreicher A, Gasteiger E, Martin MJ, Michoud K, O'Donovan C, Phan I, et al: **The SWISS-PROT protein knowledgebase and its supplement TrEMBL in 2003.** *Nucleic Acids Res* 2003, **31:**365-370.

28. Kanehisa M, Araki M, Goto S, Hattori M, Hirakawa M, Itoh M, Katayama T, Kawashima S, Okuda S, Tokimatsu T, Yamanishi Y: **KEGG for linking genomes to life and the environment.** *Nucleic Acids Res* 2008, **36:**D480-484.

29. Tatusov RL, Fedorova ND, Jackson JD, Jacobs AR, Kiryutin B, Koonin EV, Krylov DM, Mazumder R, Mekhedov SL, Nikolskaya AN, et al: **The COG database: an updated version includes eukaryotes.** *BMC Bioinformatics* 2003, **4:**41.

30. Hunter S, Apweiler R, Attwood TK, Bairoch A, Bateman A, Binns D, Bork P, Das U, Daugherty L, Duquenne L, et al: **InterPro: the integrative protein signature database.** *Nucleic Acids Res* 2009, **37:**D211-215.

31. Lin Y, Ye C, Li X, Chen Q, Wu Y, Zhang F, Pan R, Zhang S, Chen S, Wang X, et al: **quarTeT: a telomere-to-telomere toolkit for gap-free genome assembly**

and centromeric repeat identification. *Hortic Res* 2023, **10:**uhad127.

32. Goel M, Sun H, Jiao WB, Schneeberger K: **SyRI: finding genomic rearrangements and local sequence differences from whole-genome assemblies.** *Genome Biol* 2019, **20:**277.

33. Zhong Y, Chen Y, Zheng D, Pang J, Liu Y, Luo S, Meng S, Qian L, Wei D, Dai S, Zhou R: **Chromosomal-level genome assembly of the orchid tree Bauhinia variegata (Leguminosae; Cercidoideae) supports the allotetraploid origin hypothesis of Bauhinia.** *DNA Res* 2022, **29**.

34. Chen F, Tholl D, Bohlmann J, Pichersky E: **The family of terpene synthases in plants: a mid-size family of genes for specialized metabolism that is highly diversified throughout the kingdom.** *Plant J* 2011, **66:**212-229.

35. Aubourg S, Lecharny A, Bohlmann J: **Genomic analysis of the terpenoid synthase (AtTPS) gene family of Arabidopsis thaliana.** *Mol Genet Genomics* 2002, **267:**730-745.

36. Thompson JD, Higgins Dg Fau - Gibson TJ, Gibson TJ: **CLUSTAL W: improving the sensitivity of progressive multiple sequence alignment through sequence weighting, position-specific gap penalties and weight matrix choice.**

37. Frazer KA, Pachter L, Poliakov A, Rubin EM, Dubchak I: **VISTA: computational tools for comparative genomics.** *Nucleic Acids Res* 2004, **32:**W273-279.

38. Xiao Y, Qu YY, Hao CH, Tang L, Zhang JL: **The complete chloroplast genome**

of Bauhinia racemosa Lam. (Fabaceae): a versatile tropical medicinal plant. *Mitochondrial DNA B Resour* 2022, **7:**1528-1530.

39. Stupar RM, Springer NM: **Cis-transcriptional variation in maize inbred lines B73 and Mo17 leads to additive expression patterns in the F1 hybrid.** *Genetics* 2006, **173:**2199-2210.
40. Duchemin W, Dupont P-Y, Campbell MA, Ganley ARD, Cox MP: **HyLiTE: accurate and flexible analysis of gene expression in hybrid and allopolyploid species.** *BMC Bioinformatics* 2015, **16:**8.
41. Carretero-Paulet L, Ahumada I, Cunillera N, Rodriguez-Concepcion M, Ferrer A, Boronat A, Campos N: **Expression and molecular analysis of the Arabidopsis DXR gene encoding 1-deoxy-D-xylulose 5-phosphate reductoisomerase, the first committed enzyme of the 2-C-methyl-D-erythritol 4-phosphate pathway.** *Plant Physiol* 2002, **129:**1581-1591.
42. Carretero-Paulet L, Cairó A Fau - Botella-Pavía P, Botella-Pavía P Fau - Besumbes O, Besumbes O Fau - Campos N, Campos N Fau - Boronat A, Boronat A Fau - Rodríguez-Concepción M, Rodríguez-Concepción M: **Enhanced flux through the methylerythritol 4-phosphate pathway in Arabidopsis plants overexpressing deoxyxylulose 5-phosphate reductoisomerase.**
43. Cochrane FC, Davin LB, Lewis NG: **The Arabidopsis phenylalanine ammonia lyase gene family: kinetic characterization of the four PAL isoforms.** *Phytochemistry* 2004, **65:**1557-1564.

44. Lichtenthaler HK: **The 1-Deoxy-D-Xylulose-5-Phosphate Pathway of Isoprenoid Biosynthesis in Plants.** *Annu Rev Plant Physiol Plant Mol Biol* 1999, **50:**47-65.

45. Gudavalli D, Pandey K, Ede VG, Sable D, Ghagare AS, Kate AS: **Phytochemistry and pharmacological activities of five species of Bauhinia genus: A review.** *Fitoterapia* 2024, **174:**105830.

46. da Fonseca STD, Teixeira TR, Ferreira JMS, Lima L, Luyten W, Castro AHF: **Flavonoid-Rich Fractions of Bauhinia holophylla Leaves Inhibit Candida albicans Biofilm Formation and Hyphae Growth.** *Plants (Basel)* 2022, **11**.

47. Chinnappan S, Kandasamy S, Arumugam S, Seralathan KK, Thangaswamy S, Muthusamy G: **Biomimetic synthesis of silver nanoparticles using flower extract of Bauhinia purpurea and its antibacterial activity against clinical pathogens.** *Environ Sci Pollut Res Int* 2018, **25:**963-969.

48. Mishra A, Sharma AK, Kumar S, Saxena AK, Pandey AK: **Bauhinia variegata leaf extracts exhibit considerable antibacterial, antioxidant, and anticancer activities.** *Biomed Res Int* 2013, **2013:**915436.

49. Liu L, Guan D, Peart MR: **The morphological structure of leaves and the dust-retaining capability of afforested plants in urban Guangzhou, South China.** *Environ Sci Pollut Res Int* 2012, **19:**3440-3449.

50. Seymour DK, Chae E, Grimm DG, Martín Pizarro C, Habring-Müller A, Vasseur F, Rakitsch B, Borgwardt KM, Koenig DA-O, Weigel DA-O: **Genetic architecture of nonadditive inheritance in Arabidopsis thaliana hybrids.**

51. Birchler JA, Yao H Fau - Chudalayandi S, Chudalayandi S Fau - Vaiman D, Vaiman D Fau - Veitia RA, Veitia RA: **Heterosis.**

52. Xiao J, Li J Fau - Yuan L, Yuan L Fau - Tanksley SD, Tanksley SD: **Dominance is the major genetic basis of heterosis in rice as revealed by QTL analysis using molecular markers.**

53. Li ZK, Luo Lj Fau - Mei HW, Mei Hw Fau - Wang DL, Wang Dl Fau - Shu QY, Shu Qy Fau - Tabien R, Tabien R Fau - Zhong DB, Zhong Db Fau - Ying CS, Ying Cs Fau - Stansel JW, Stansel Jw Fau - Khush GS, Khush Gs Fau - Paterson AH, Paterson AH: **Overdominant epistatic loci are the primary genetic basis of inbreeding depression and heterosis in rice. I. Biomass and grain yield.**

54. Springer NM, Stupar RM: **Allelic variation and heterosis in maize: how do two halves make more than a whole?**

55. Guo M, Rupe Ma Fau - Yang X, Yang X Fau - Crasta O, Crasta O Fau - Zinselmeier C, Zinselmeier C Fau - Smith OS, Smith Os Fau - Bowen B, Bowen B: **Genome-wide transcript analysis of maize hybrids: allelic additive gene expression and yield heterosis.**

56. Goff SA, Zhang Q: **Heterosis in elite hybrid rice: speculation on the genetic and biochemical mechanisms.**

57. Sahu SK, Thangaraj M, Kathiresan K: **DNA Extraction Protocol for Plants with High Levels of Secondary Metabolites and Polysaccharides without Using Liquid Nitrogen and Phenol.** *ISRN Mol Biol* 2012, **2012:**205049.

58. Wang O, Chin R, Cheng X, Wu MKY, Mao Q, Tang J, Sun Y, Anderson E, Lam

HK, Chen D, et al: **Efficient and unique cobarcoding of second-generation sequencing reads from long DNA molecules enabling cost-effective and accurate sequencing, haplotyping, and de novo assembly.** *Genome Res* 2019, **29:**798-808.

59. Lieberman-Aiden E, Van Berkum NL, Williams L, Imakaev M, Ragoczy T, Telling A, Amit I, Lajoie BR, Sabo PJ, Dorschner MO: **Comprehensive mapping of long-range interactions reveals folding principles of the human genome.** *science* 2009, **326:**289-293.

60. **BGISEQ-500 WGS library construction** [https://www.protocols.io/view/bgiseq-500-wgs-library-construction-ps5dng6]

61. Ashton PM, Nair S, Dallman T, Rubino S, Rabsch W, Mwaigwisya S, Wain J, O'Grady JA-O: **MinION nanopore sequencing identifies the position and structure of a bacterial antibiotic resistance island.**

62. Bolger AM, Lohse M, Usadel B: **Trimmomatic: a flexible trimmer for Illumina sequence data.** *Bioinformatics* 2014, **30:**2114-2120.

63. Marcais G, Kingsford C: **A fast, lock-free approach for efficient parallel counting of occurrences of k-mers.** *Bioinformatics* 2011, **27:**764-770.

64. Vurture GW, Sedlazeck FJ, Nattestad M, Underwood CJ, Fang H, Gurtowski J, Schatz MC: **GenomeScope: fast reference-free genome profiling from short reads.** *Bioinformatics* 2017, **33:**2202-2204.

65. Weisenfeld NI, Kumar V, Shah P, Church DM, Jaffe DB: **Direct determination of diploid genome sequences.**

66. Li H, Durbin R: **Fast and accurate short read alignment with Burrows-Wheeler transform.** *Bioinformatics* 2009, **25:**1754-1760.

67. Simao FA, Waterhouse RM, Ioannidis P, Kriventseva EV, Zdobnov EM: **BUSCO: assessing genome assembly and annotation completeness with single-copy orthologs.** *Bioinformatics* 2015, **31:**3210-3212.

68. Danecek P, Bonfield JK, Liddle J, Marshall J, Ohan V, Pollard MO, Whitwham A, Keane T, McCarthy SA, Davies RM, Li H: **Twelve years of SAMtools and BCFtools.** *Gigascience* 2021, **10**.

69. **Repeat Library Construction-Advanced** [http://weatherby.genetics.utah.edu/MAKER/wiki/index.php/Repeat_Library_Construction-Advanced]

70. Chen N: **Using RepeatMasker to identify repetitive elements in genomic sequences.** *Curr Protoc Bioinformatics* 2004, **Chapter 4:**Unit 4 10.

71. Bao WD, Kojima KK, Kohany O: **Repbase Update, a database of repetitive elements in eukaryotic genomes.** *Mobile DNA* 2015, **6**.

72. Xu Z, Wang H: **LTR_FINDER: an efficient tool for the prediction of full-length LTR retrotransposons.** *Nucleic Acids Research* 2007, **35:**W265-W268.

73. Flynn JM, Hubley R, Goubert C, Rosen J, Clark AG, Feschotte C, Smit AF: **RepeatModeler2 for automated genomic discovery of transposable element families.** *Proc Natl Acad Sci U S A* 2020, **117:**9451-9457.

74. Benson G: **Tandem repeats finder: a program to analyze DNA sequences.** *Nucleic Acids Research* 1999, **27:**573-580.

75. Bruna T, Hoff KJ, Lomsadze A, Stanke M, Borodovsky M: **BRAKER2: automatic eukaryotic genome annotation with GeneMark-EP plus and AUGUSTUS supported by a protein database.** *Nar Genomics and Bioinformatics* 2021, **3**.

76. Kim D, Paggi JM, Park C, Bennett C, Salzberg SL: **Graph-based genome alignment and genotyping with HISAT2 and HISAT-genotype.** *Nat Biotechnol* 2019, **37:**907-915.

77. Kriventseva EV, Kuznetsov D, Tegenfeldt F, Manni M, Dias R, Simao FA, Zdobnov EM: **OrthoDB v10: sampling the diversity of animal, plant, fungal, protist, bacterial and viral genomes for evolutionary and functional annotations of orthologs.** *Nucleic Acids Res* 2019, **47:**D807-D811.

78. Marcais G, Delcher AL, Phillippy AM, Coston R, Salzberg SL, Zimin A: **MUMmer4: A fast and versatile genome alignment system.** *PLoS Comput Biol* 2018, **14:**e1005944.

79. Lowe TM, Eddy SR: **tRNAscan-SE: a program for improved detection of transfer RNA genes in genomic sequence.** *Nucleic Acids Res* 1997, **25:**955-964.

80. Nawrocki EP, Kolbe DL, Eddy SR: **Infernal 1.0: inference of RNA alignments.** *Bioinformatics* 2009, **25:**1335-1337.

81. Emms DM, Kelly S: **OrthoFinder: phylogenetic orthology inference for comparative genomics.** *Genome Biol* 2019, **20:**238.

82. Katoh K, Standley DM: **MAFFT Multiple Sequence Alignment Software**

Version 7: Improvements in Performance and Usability. *Molecular Biology and Evolution* 2013, **30:**772-780.

83. Castresana J: **Selection of conserved blocks from multiple alignments for their use in phylogenetic analysis.** *Molecular Biology and Evolution* 2000, **17:**540-552.

84. Nguyen LT, Schmidt HA, von Haeseler A, Minh BQ: **IQ-TREE: a fast and effective stochastic algorithm for estimating maximum-likelihood phylogenies.** *Mol Biol Evol* 2015, **32:**268-274.

85. Zhang C, Rabiee M, Sayyari E, Mirarab S: **ASTRAL-III: polynomial time species tree reconstruction from partially resolved gene trees.** *BMC Bioinformatics* 2018, **19:**153.

86. Stamatakis A: **RAxML version 8: a tool for phylogenetic analysis and post-analysis of large phylogenies.** *Bioinformatics* 2014, **30:**1312-1313.

87. Yang Z: **PAML 4: phylogenetic analysis by maximum likelihood.** *Mol Biol Evol* 2007, **24:**1586-1591.

88. De Bie T, Cristianini N, Demuth JP, Hahn MW: **CAFE: a computational tool for the study of gene family evolution.** *Bioinformatics* 2006, **22:**1269-1271.

89. Jin JJ, Yu WB, Yang JB, Song Y, dePamphilis CW, Yi TS, Li DZ: **GetOrganelle: a fast and versatile toolkit for accurate de novo assembly of organelle genomes.** *Genome Biology* 2020, **21**.

90. Shi L, Chen H, Jiang M, Wang L, Wu X, Huang L, Liu C: **CPGAVAS2, an integrated plastome sequence annotator and analyzer.** *Nucleic Acids Res*

2019, **47:**W65-W73.

91. Roberts A, Pachter L: **Streaming fragment assignment for real-time analysis of sequencing experiments.** *Nat Methods* 2013, **10:**71-73.

92. Love MI, Huber W, Anders S: **Moderated estimation of fold change and dispersion for RNA-seq data with DESeq2.** *Genome Biol* 2014, **15:**550.

93. Wang Y, Tang H, Debarry JD, Tan X, Li J, Wang X, Lee TH, Jin H, Marler B, Guo H, et al: **MCScanX: a toolkit for detection and evolutionary analysis of gene synteny and collinearity.** *Nucleic Acids Res* 2012, **40:**e49.

Table 1. Statistics for genome assembly and annotation of three *Bauhinia* species.

Species	*Bauhinia blakeana* Hmat	*Bauhinia blakeana* Hpat	*Bauhinia purpurea*	*Bauhinia variegata*
**Assembly feature**				
Estimated genome size	290,967,258	290,967,258	303,677,508	314,486,060
Assembled genome size	275,484,977	290,698,387	285,147,376	311,011,643
GC content	34.05%	34.22%	33.88%	34.04%
N50 of contigs (bp)	19,540,838	20,987,561	161,057	109,234
N50 of scaffold (bp)	19,540,838	20,987,561	1,475,774	2,613,106
Complete BUSCOs	C:99.0%[S:81.5%,D:17.5%],F:0.6%,M:0.4%	C:99.2%[S:78.6%,D:20.6%],F:0.7%,M:0.1%	C:97.8%[S:77.6%,D:20.2%],F:1.4%,M:0.8%	C:98.4%[S:77.0%,D:21.4%],F:1.2%,M:0.4%
**HIC**				
Anchor size	/	/	285,099,865	310,940,945
Anchor rate	/	/	99.98%	99.98%
Number of pseudochromosomes	14	14	14	14
N50 of scaffold (bp)	19,540,838	19,540,838	21,596,737	24,404,849
**Characteristics of protein-coding genes**				
Total number of protein-coding genes	37,804	37,956	38,735	40,111
Mean gene size (bp)	2615.06	2619.36	2602.43	2595.09
Mean CDS length (bp)	1120.11	1179.70	1192.31	1187.11
Mean exon number per gene	5.36	5.16	5.13	5.08
Mean exon length (bp)	208.89	228.60	232.52	233.74
Mean intron length (bp)	342.71	346.02	341.63	345.20
Complete BUSCOs	C:94.2%[S:78.4%,D:15.8%],F:4.0%,M:1.8%	C:96.4%[S:79.2%,D:17.2%],F:2.4%,M:1.2%	C:97.4%[S:78.4%,D:19.0%],F:1.4%,M:1.2%	C:97.8%[S:77.0%,D:20.8%],F:1.4%,M:0.8%
**Functional annotation by searching public databases**				
% of proteins with hits in NCBI nr database	97.25%	98.16%	97.80%	95.80%
% of proteins with hits in Swiss-Prot database	75.86%	78.54%	80.60%	79.54%
% of proteins with hits in KEGG database	70.12%	72.58%	74.49%	50.44%
% of proteins with hits in KOG database	70.77%	73.24%	75.01%	74.67%
% of proteins with hits in TrEMBL database	92.98%	94.23%	96.62%	96.30%
% of proteins with hits in Interpro database	93.68%	94.95%	96.83%	96.89%

% of proteins with functional annotation (combined)	99.98%	99.97%	99.98%	99.96%

Figure legends

Figure 1. Genome assemblies of *the Bauhinia* species.

(A) Circos plot showing the genome assemblies for *B. purpurea* (Bpur1-Bpur14) and *B. variegata* (Bvar1-Bvar14). The outer tracks display pseudochromosomes, a) gene number, b) GC content, c) repeat density, d) LTR density, e) LTR/Copia density, f) LTR/Gypsy density. Synteny gene blocks between the two assemblies are visualized by links inside the circle. **(B)** Circos plot illustrating the maternal haplotype (Hmat1-Hmat14) and the paternal haplotype (Hpat1-Hpat14) of *B. blakeana*. **(C)** Structural variations between the two *B. blakeana* haplotype assemblies. The maternal haplotype Hmat were used as the reference. Gene density, centromere, and telomere locations were also plotted.

Figure 2. Comparative genomic analysis and expansion of terpene synthase genes.

(A) Phylogenetic relationship and divergence times between *Bauhinia* species and other selected plant species. The blue numbers near the divergence nodes represent divergence times. Expanded and contracted ortholog groups at the corresponding node are indicated by green and red numbers, respectively. **(B)** KEGG enrichment analysis of expanded gene families on the *Bauhinia* genus clade, displaying the top20 KEGG terms. **(C)** Identification of candidate terpene synthases (TPSs) in the *Bauhinia* species

with subfamily classification revealing six major clades (TPS-a, b, c, e, f, and g).

Figure 3. Maternal parent confirmation of *B. blakeana* through comparative chloroplast genome analyses.

(A) Comparative analysis of six *Bauhinia* cp genomes using mVISTA, with the assembled *B. blakeana* genome from this study as the reference. The y-axis represents the percent identity, ranging from 50% to 100%. Grey arrows indicate the direction of gene transcription. **(B)** Identification of a one-base pair deletion at the 1169,48 base site in the published *B. purpurea* cp genome (NC061218). **(C)** Phylogenetic tree of the *Bauhinia* genus construced based on available cp genomes.

Figure 4. Comparative transcriptomics analyses.

(A) Summary of DEGs in each possible comparison among the three *Bauhinia* species. Arrows represent the comparisons, with the numbers and proportions of up-regulated DEGs indicated at the arrows ends. The tag in the middle of the arrow and the arrow color indicate the reference genome used. **(B)** Box plot illustrating the number and distribution of DEGs (DE1+, log2|FC| > 1) between *B. purpurea* and *B. variegata* using different reference genomes. DEGs with log2|FC| between 1 to 2 (1-2), DEGs with log2|FC| between 2 to 4 (2-4), etc. **(C)** KEGG enrichment analysis of DEGs exhibiting up-regulated expression level in *B. purpurea* (pink) compared to *B. variegata* (blue).

Figure 5. Comparative transcriptomics analyses of DEGs between *B. blakeana* and

parental species.

(A) Box plot illustrating the number and distribution of DEGs (DE1+, log2|FC| > 1) between *B. blakeana* and *B. purpurea* (with *B. purpurea* as the reference) and between *B. blakeana* and *B. variegata* (with *B. variegata* as the reference). **(B)** KEGG enrichment analysis of DEGs with up-regulated expression levels in *B. blakeana* (purple), *B. purpurea* (pink) and *B. variegata* (blue) in the comparisons of *B. blakeana* to the parental species. The arrows highlight overlapping KEGG terms enriched in DEGs with higher expression levels in *B. purpurea* (red) or *B. variegata* (blue) during the parental species comparison. **(C)** Box plot illustrating the number and distribution of DEGs (DE1+, log2|FC| > 1) between *B. blakeana* and the MPV using different references.

Figure 6. Analysis of differentially expressed alleles in *B. blakeana*.

(A) Volcano plot displaying ASEGs in *B. blakeana* identified using the HyLiTe pipeline. **(B)** Volcano plot illustrating ASEGs in *B. blakeana* identified through genome-wide identification. **(C)** Genomic distribution of ASEGs revealing an interlaced pattern with scattered distribution. ASEGs with maternal allele dominance were plotted on the Hmat chromosomes, and ASEGs with paternal allele dominance were plotted on the Hpat chromosomes, respectively. **(D)** Top 12 GO enrichment results of ASEGs in *B. blakeana*, with paternal allele dominance ASEGs (blue dots) and maternal allele dominance ASEGs (red dots) within each GO category plotted.

Figure 7. Pigment biosynthesis-related metabolic pathways in *B. blakeana*.

The carotenoid enzymatic genes are divided into three groups: "MEP Pathway", "Carotene Biosynthesis", and "Xanthophylls Biosynthesis". The chlorophyll enzymatic genes are divided into three groups: "Chlorophyll Biosynthesis", "Chlorophyll Cycle", and "Chlorophyll Degradation". Genes identified as ASEGs are marked by the gene ID, with maternal allele dominance shown in red and paternal allele dominance shown in blue. **(A)** Heatmap depicting gene expression values (FPKM) within the anthocyanin metabolic pathway across three *Bauhinia* species. **(B)** Heatmap depicting gene expression values (FPKM) within the carotenoid metabolic pathway across three *Bauhinia* species. **(C)** Heatmap depicting gene expression values (FPKM) within the chlorophyll metabolic pathway across three *Bauhinia* species. **(D)** Heatmap depicting gene expression values (sum-up FPKM of each gene copy of the same gene) within the pigment biosynthesis-related metabolic pathway across three *Bauhinia* species. The expression pattern of the corresponding gene in *B. blakeana* is marked by the colored dot after the gene ID, with yellow dot indicating over-dominance and pink dot indicating high parent dominance.

Supplementary Figure S1: *K*-mer analysis of three *Bauhinia* species with GenomeScope 2.0 (*k*=21). The x-axis refers to the *k*-mer coverage, and the y-axis refers to the frequency of the *k*-mer for a given coverage. **(A)** Genome survey result of *B. purpurea*. **(B)** Genome survey result of *B. variegata*. **(C)** Genome survey result of *B. blakeana*.

Supplementary Figure S2. Hi-C plot of the pseudochromosomes level assemblies of *B. purpurea* and *B. variegata*. **(A)** Chromatin contact matrix of *B. purpurea* generated by ALLHiC. **(B)** Chromatin contact matrix of *B. variegata* generated by ALLHiC.

Supplementary Figure S3: Principal component analysis (PCA) plot of RNA-seq data.

PCA plot of RNA-seq data with three *Bauhinia* species and 3 biological replicates each. The x-axis represents the first principal component (PC1), The y-axis represents the second principal component (PC2). The position of each sample in the plot indicates its relative similarity or dissimilarity to other samples based on the alignment of RNA-seq data to the selected reference.

Supplementary Figure S4: Violin box plots comparing gene expression values (FPKM) for orthologous genes within the same sample, using *B. purpurea* and *B. variegata* as reference genomes. The figure consists of eight violin box plots, each representing a different sample. The violin plot shows the distribution of gene expression values, with the width indicating the density of data points at different expression levels. Welch's t-test was employed to compare the gene expression data between groups.

Supplementary Figure S5: Bar plot of the mean difference in gene expression

values (FPKM) from paired t-test results. (A) Bar plot illustrating the mean difference in gene expression values for each of the *Bauhinia* samples, with consideration given to distinct reference genomes used (*B. purpurea* or *B. variegata*). **(B)** Bar plot illustrating the mean difference in expression values within *B. blakeana* samples when utilizing varying reference genomes. We compared the allelic expression using the *B. blakeana* haplotype metagenome with the overall expression values when using either *B. purpurea* or *B. variegata* as reference.

Figure 1

Click here to access/download;Figure;Fig 1.pdf

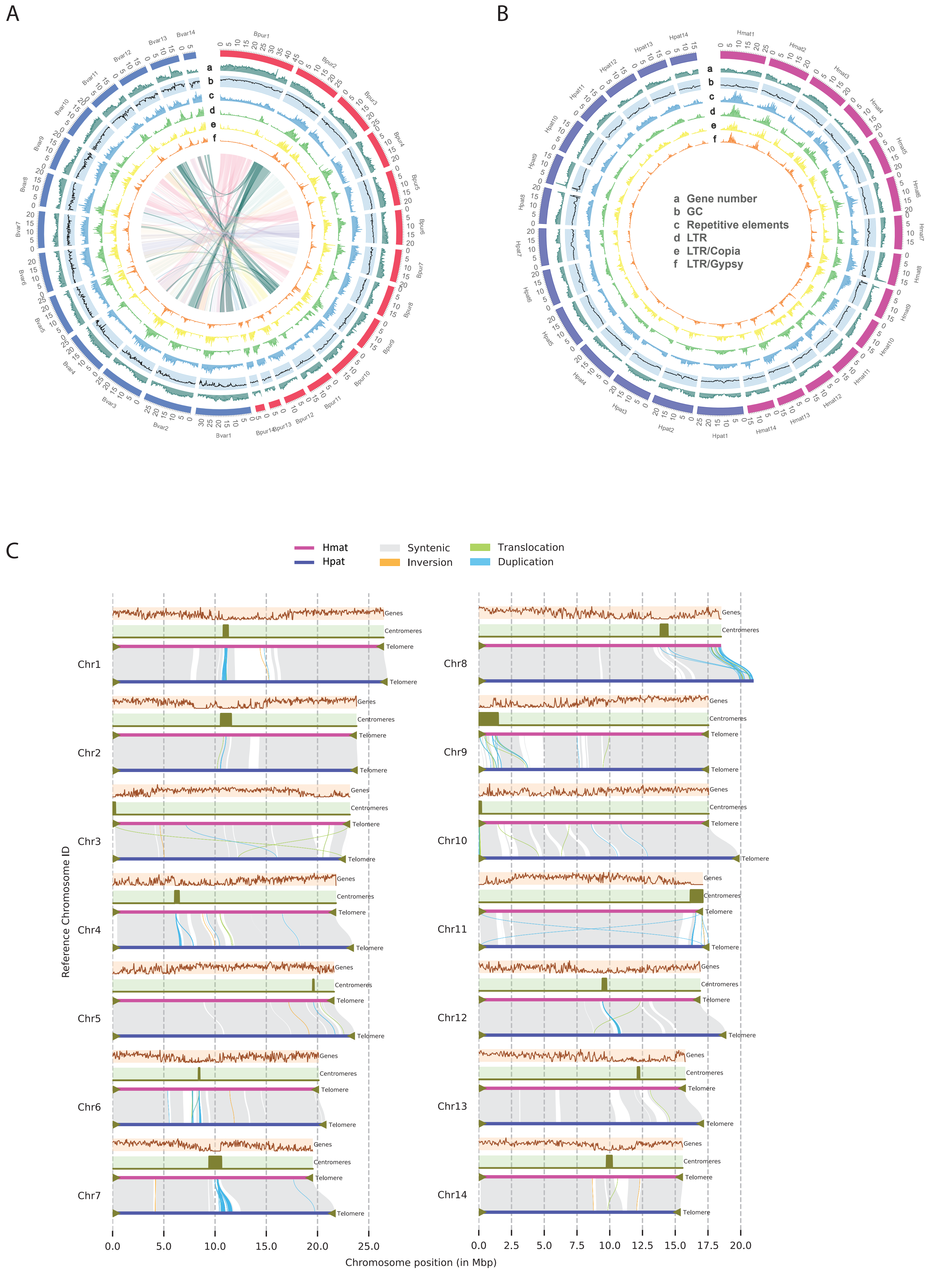

Figure 2

Click here to access/download;Figure;Fig 2.pdf

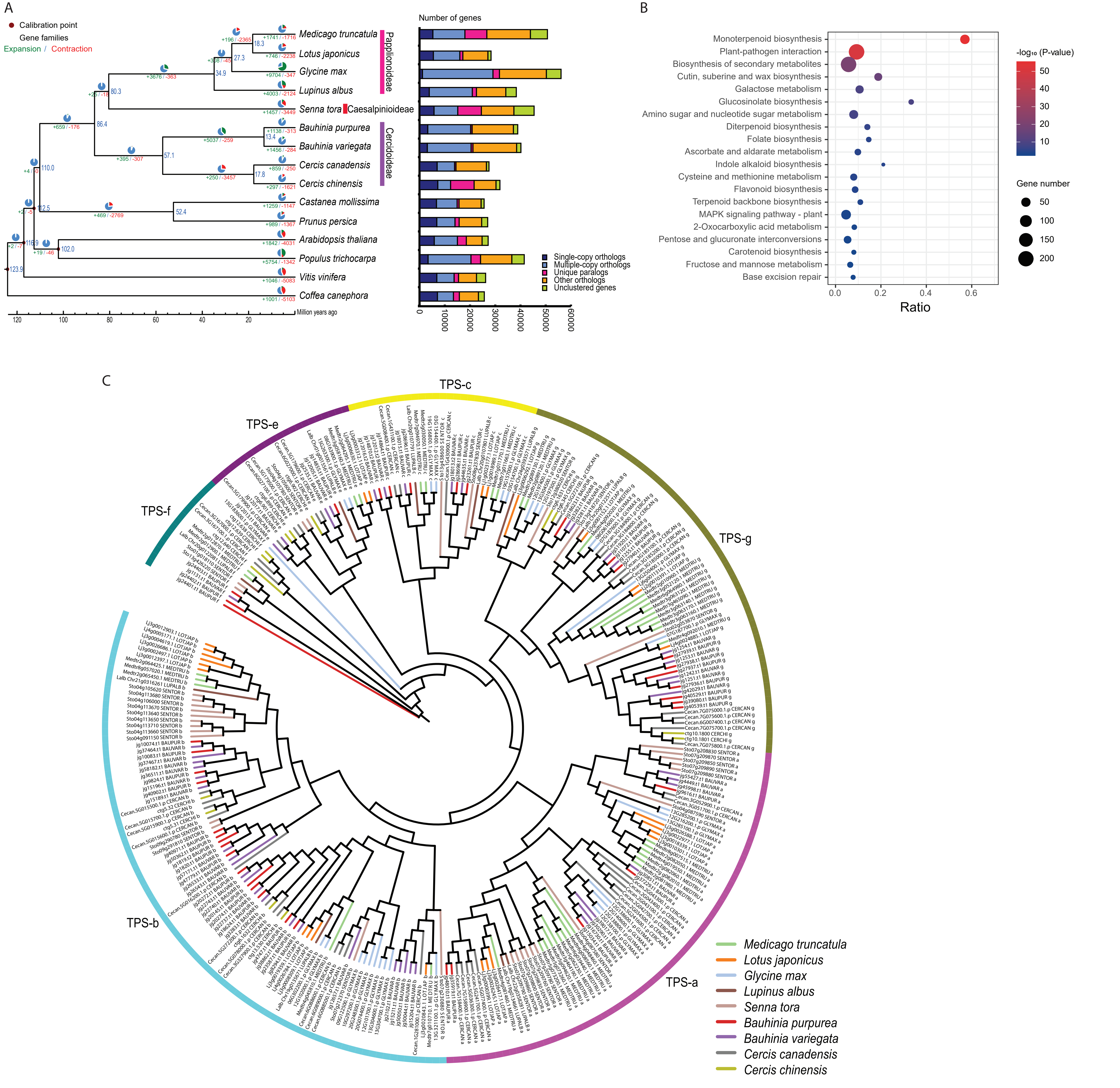

Figure 3

Click here to access/download;Figure;Fig 3.pdf

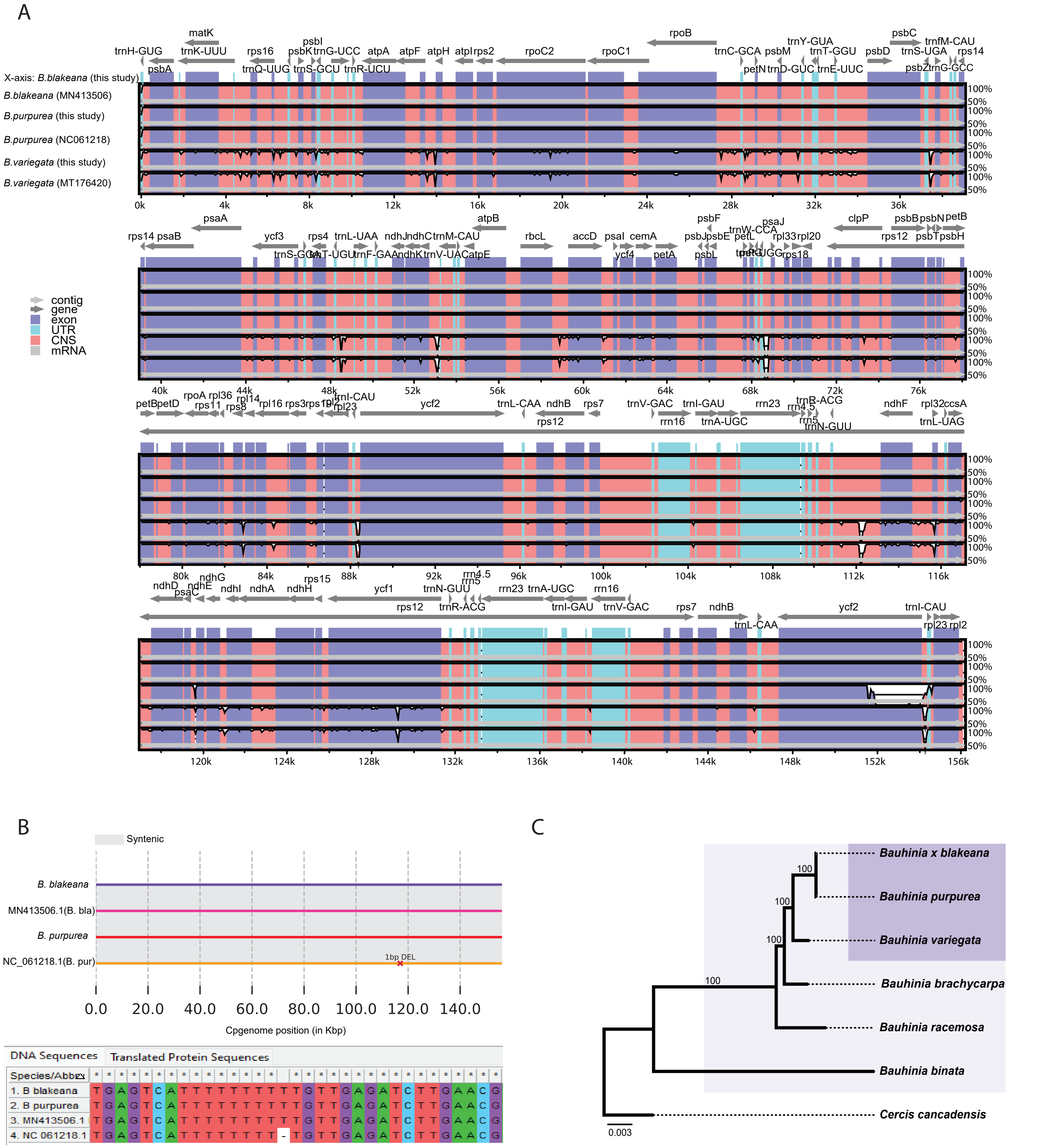

Figure 4

Click here to access/download;Figure;Fig 4.pdf

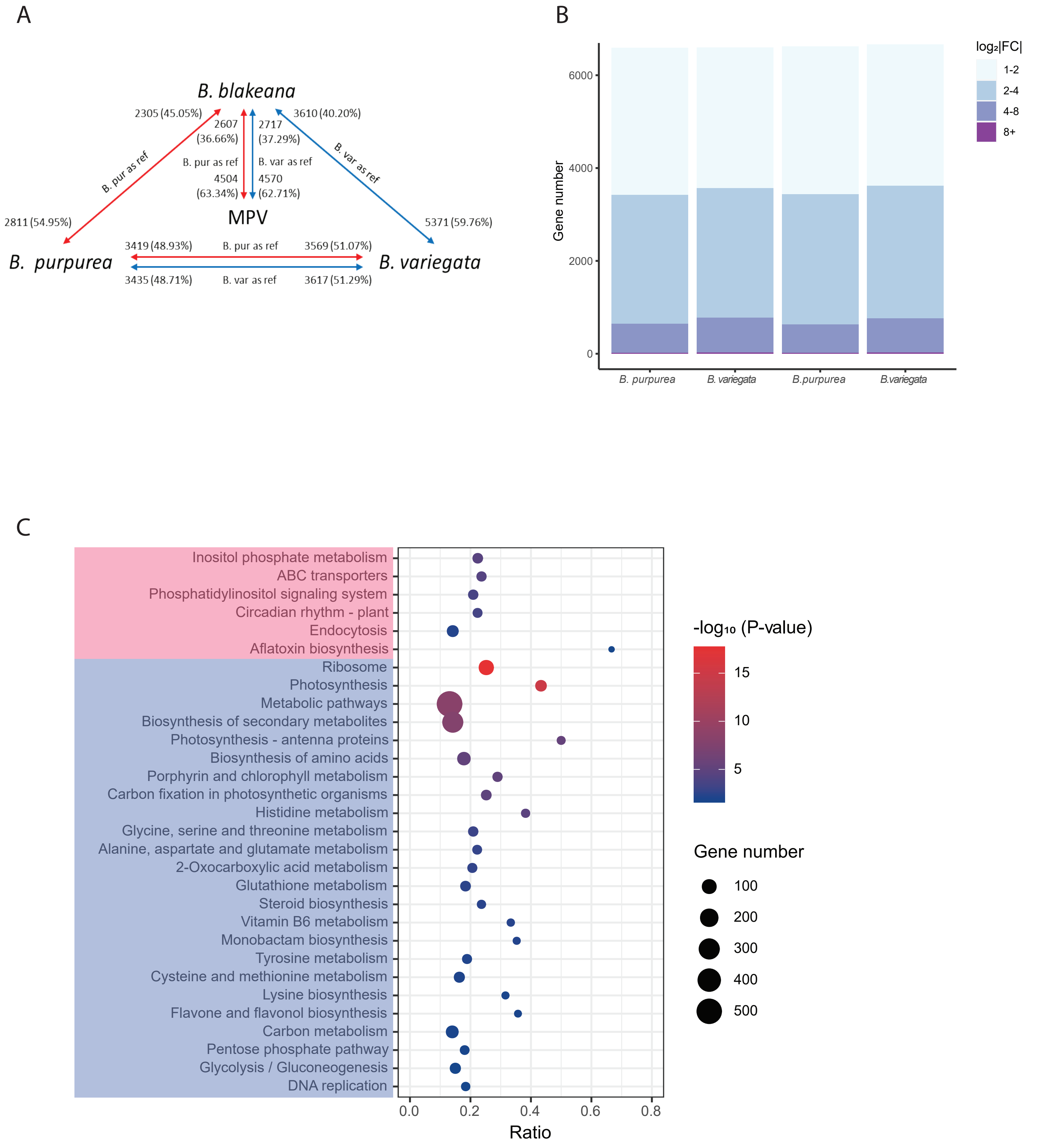

Figure 5

Click here to access/download;Figure;Fig 5.pdf

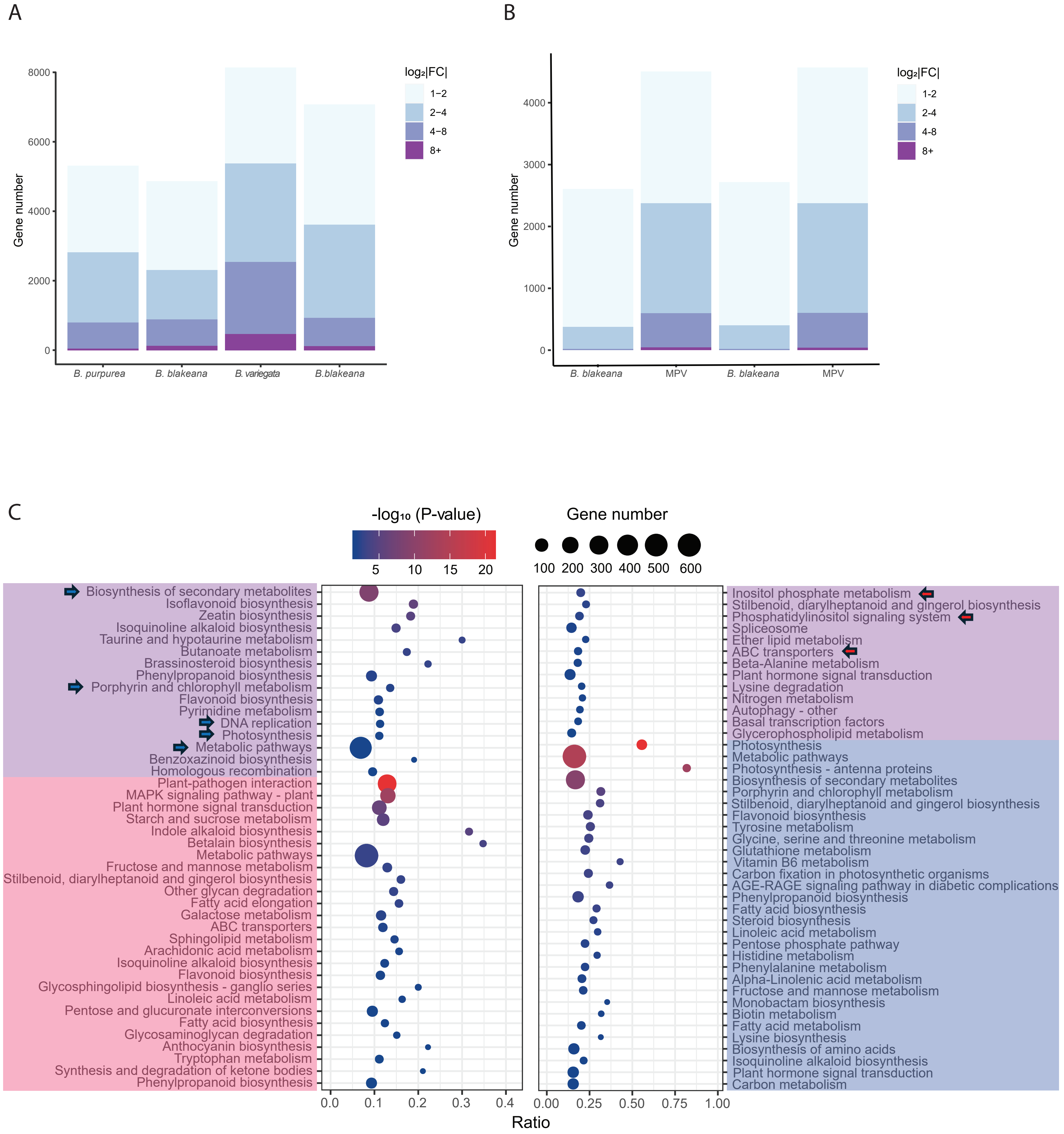

Figure 6

Click here to access/download;Figure;Fig 6.pdf

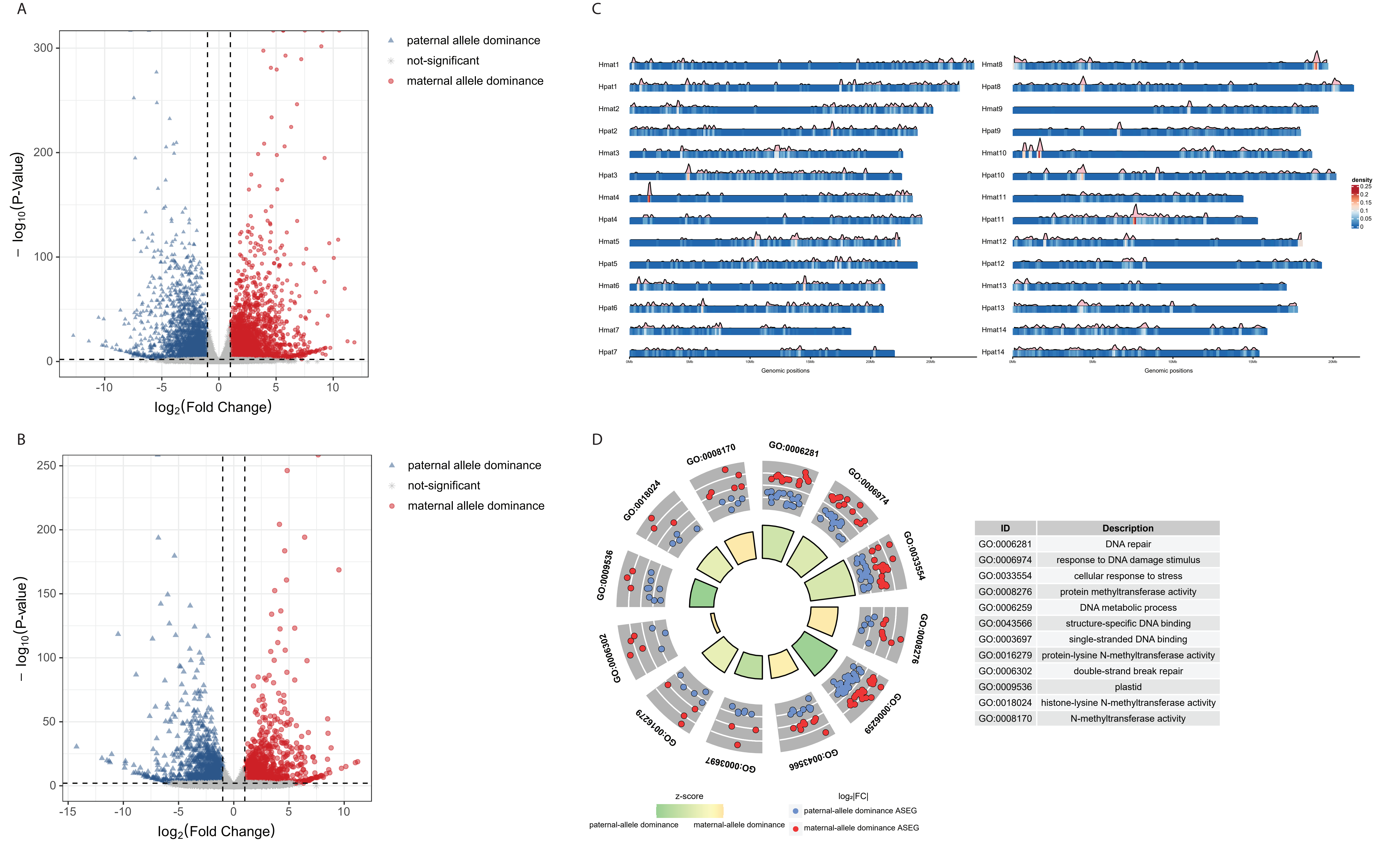

ID	Description
GO:0006281	DNA repair
GO:0006974	response to DNA damage stimulus
GO:0033554	cellular response to stress
GO:0008276	protein methyltransferase activity
GO:0006259	DNA metabolic process
GO:0043566	structure-specific DNA binding
GO:0003697	single-stranded DNA binding
GO:0016279	protein-lysine N-methyltransferase activity
GO:0006302	double-strand break repair
GO:0009536	plastid
GO:0018024	histone-lysine N-methyltransferase activity
GO:0008170	N-methyltransferase activity

Figure 7

Click here to access/download;Figure;Fig 7.pdf

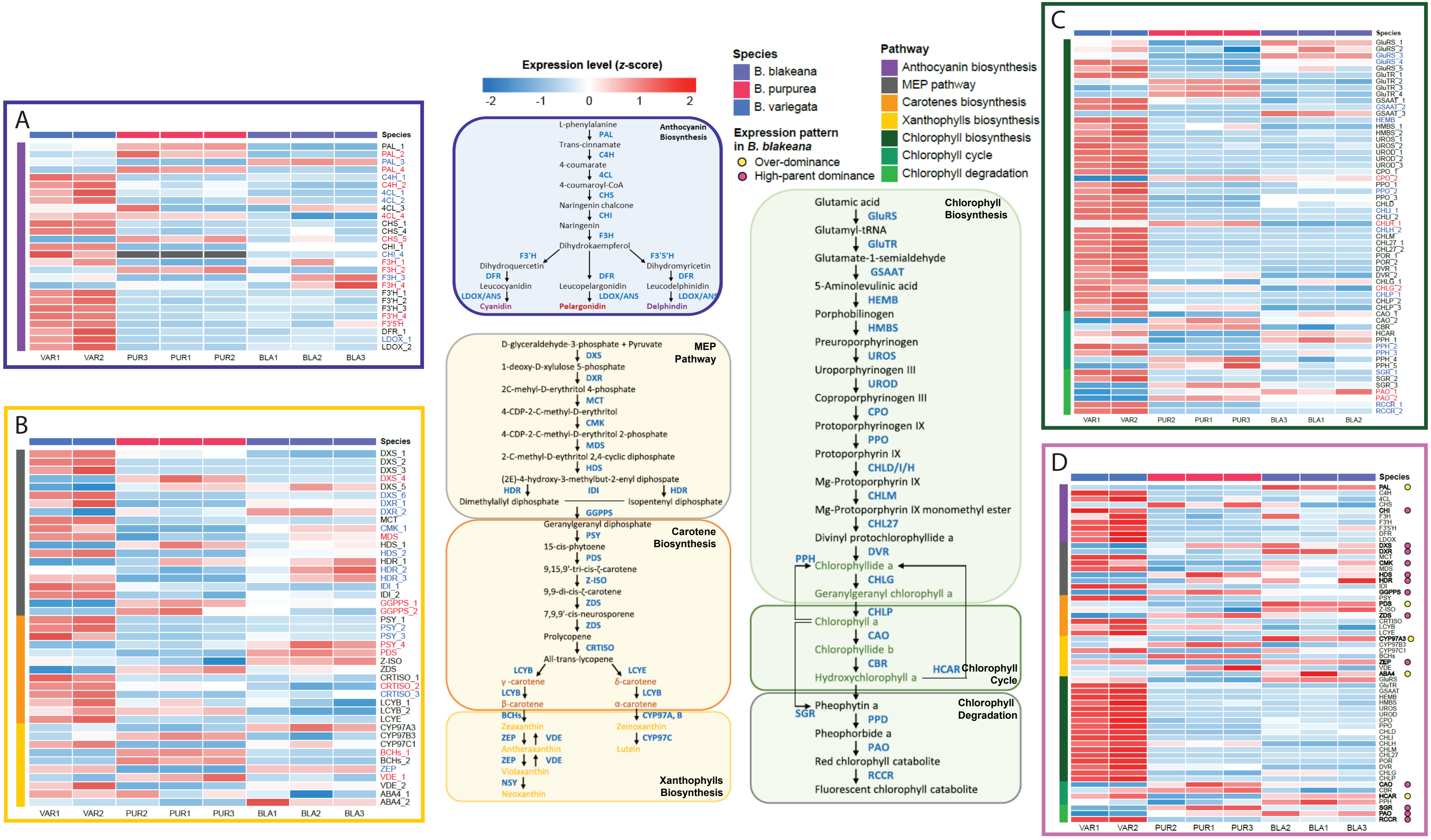

Supplementary Material TableS1-S23

Click here to access/download
Supplementary Material
Table S1-S26_Supplementary Material.xlsx

Supplementary Figure S1

Click here to access/download
Supplementary Material
Fig S1.pdf

Supplementary Figure S2

Click here to access/download
Supplementary Material
Fig S2.pdf

Supplementary Figure S3

Click here to access/download
Supplementary Material
Fig S3.pdf

Supplementary Figure S4

Click here to access/download
Supplementary Material
Fig S4.pdf

Supplementary Figure S5

Click here to access/download
Supplementary Material
Fig S5.pdf
