# Supplementary material for: The haplotype-resolved T2T genome for Bauhinia × blakeana sheds light on the genetic basis of flower heterosis
Source: Gigascience. 2025 Apr 25;14:giaf044. doi: 10.1093/gigascience/giaf044 (PMC12012898; doi:10.1093/gigascience/giaf044)
Supplement: giaf044_GIGA-D-24-00537_Revision_1 [file giaf044_giga-d-24-00537_revision_1.pdf]

GigaScience

The Haplotype-resolved T2T Genome for Bauhinia x blakeana Sheds Light on the Genetic Basis of Flower Heterosis

--Manuscript Draft--

**Manuscript Number:**	GIGA-D-24-00537R1
**Full Title:**	The Haplotype-resolved T2T Genome for Bauhinia x blakeana Sheds Light on the Genetic Basis of Flower Heterosis
**Article Type:**	Research
**Funding Information:**	Collaborative Research Grant, Research Grants Council (C4049-23EF) — Dr. Stephen Kwok Wing Tsui<br>General Research Grant, Research Grants Council (14119420) — Dr. Stephen Kwok Wing Tsui<br>General Research Grant, Research Grants Council (14121222) — Dr. Stephen Kwok Wing Tsui<br>Theme-based Research Scheme, Research Grants Council (T11-709/21-N) — Dr. Stephen Kwok Wing Tsui<br>Theme-based Research Scheme, Research Grants Council (T12-716/22-R) — Dr. Stephen Kwok Wing Tsui<br>Theme-based Research Scheme, Research Grants Council (T11-702/24-N) — Dr. Stephen Kwok Wing Tsui
**Abstract:**	Background<br>The Hong Kong Orchid Tree Bauhinia x blakeana Dunn has long been proposed to be a sterile interspecific hybrid exhibiting flower heterosis when compared to its likely parental species, B. purpurea L. and B. variegata L. Here, we report comparative genomic and transcriptomic analyses of the three Bauhinia species.<br>Findings<br>We generated chromosome-level assemblies for the parental species and applied a trio-binning approach to construct a haplotype-resolved telomere-to-telomere (T2T) genome for B. blakeana. Comparative chloroplast genome analysis confirmed B. purpurea as the maternal parent. Transcriptome profiling of flower tissues highlighted a closer resemblance of B. blakeana to its maternal parent. Differential gene expression analyses revealed distinct expression patterns among the three species, particularly in biosynthetic and metabolic processes. To investigate the genetic basis of flower heterosis observed in B. blakeana, we focused on gene expression patterns within pigment biosynthesis-related pathways. High-patent dominance and over-dominance expression patterns were observed, particularly in genes associated with carotenoid biosynthesis. Additionally, allele-specific expression analysis revealed a balanced contribution of maternal and paternal alleles in shaping the gene expression patterns in B. blakeana.<br>Conclusions<br>Our study offers valuable insights into the genome architecture of hybrid B. blakeana, establishing a comprehensive genomic and transcriptomic resource for future functional genetics research within the Bauhinia genus. It also serves as a model for exploring the characteristics of hybrid species using T2T haplotype-resolved genomes, providing a novel approach to understanding genetic interactions and evolutionary mechanisms in complex genomes with high heterozygosity.
**Corresponding Author:**	Stephen Tsui, PhD<br>The Chinese University of Hong Kong<br>Shatin, HONG KONG
**Corresponding Author Secondary Information:**	
**Corresponding Author's Institution:**	The Chinese University of Hong Kong

Powered by Editorial Manager® and ProduXion Manager® from Aries Systems Corporation

**Corresponding Author's Secondary Institution:**	
**First Author:**	Weixue Mu
**First Author Secondary Information:**	
**Order of Authors:**	Weixue Mu
	Joshua Casey Darian
	Wing-Kin Sung
	Xing Guo
	Tuo Yang
	Mandy Wai Man Tang
	Ziqiang Chen
	Steve Kwan Hok Tong
	Irene Wing Shan Chik
	Robert L Davidson
	Scott C Edmunds
	Tong Wei
	Stephen Kwok Wing Tsui
**Order of Authors Secondary Information:**	
**Response to Reviewers:**	Reviewer reports:<br><br>Reviewer #1: This paper presents the genome assemblies of three Bauhinia species: B. purpurea, B. variegata, and B. blakeana. The third of these is an infertile hybrid. The paper presents evidence that B. blakeana and B. purpurea are the maternal and paternal parents, respectively. The largest portion of the analysis presented in the paper describes differential expression observed between the two homoeologous chromosomes from the parents.<br><br>The paper is, I think, exemplary in the rigor and completeness of its analyses. The assemblies all sound to be of very high quality. The differential expression analyses are difficult to conduct well; but I think that the completeness of the contributing genomes aids in producing compelling results.<br><br>I have the following recommendations:<br>1. There is a basic aspect of the cytogenetics that I think should be explicitly described early-on. Is the hybridization that produced B. blakeana due to contribution of one set of chromatids from B. variegata and one set from B. purpurea, such that the 2n (sporophytic) genome has 28 chromatids -- the same as in both parents? I assume this is the case, but it differs from other cases of wide hybridization that are followed by a genome doubling, such as Arachis hypogaea. In A. hypogaea, a merger of A. duranensis and A. ipaensis was followed by a genome doubling. Presumably, the genome doubling rescued the probably-sterile initial wide hybrid.<br>R: We thank the reviewer for these insightful comments. As suggested, we have addressed the cytogenetic basis of B. blakeana's hybrid origin in the manuscript (Page 5). Specifically, we have clarified that B. blakeana likely arose from the integration of one set of chromatids from each parent (B. purpurea and B. variegata), resulting in a diploid (2n) genome with 28 chromatids, consistent with both parental species. This distinguishes B. blakeana from other cases of wide hybridization followed by genome doubling, such as Arachis hypogaea, where genome duplication restored fertility in the hybrid. In B. blakeana, the absence of genome doubling likely contributes to its sterility due to irregular chromosome segregation and abnormal spindle formation during microsporogenesis.<br><br>2. It seems that it would be worthwhile to mention that Bauhinia (the genus) appears to have been the product of an allotetraploid merger, with double the number of

chromosomes in the earlier-diverging genus Cercis. The relevant citation would be Zhong et al. (2022), which is already cited, in the context of the phylogeny of the Cercidoideae. This earlier result is relevant in that it would be another instance of a "wide hybrid" in the genus (with an additional genome doubling).
R: As recommended, we have mentioned the allotetraploid origin of the genus Bauhinia and its relationship to the earlier-diverging genus Cercis in the revised manuscript (Page 5). We believe this addition enriches the discussion on the evolutionary dynamics of Bauhinia and provides a broader context for understanding the genomic history of the genus.

3. (a grammatical error): pages 19-20: (four instances): "exhibited up-regulated" should be either "exhibited up-regulation" or "were up-regulated" ("up-regulated" is a past-participle of the verb "regulate," whereas "regulation" is a noun. The construction "exhibited upregulation" uses a verb-noun form.)
R: We appreciate the reviewer's attention to detail. In the revised manuscript, we have corrected all instances of "exhibited up-regulated" to either "exhibited up-regulation" or "were up-regulated" as suggested (Pages 20-21).

Reviewer #2: Mu et al. reports a haplotype-resolved telomere-to-telomere (T2T) genome for B. blakeana plus chromosome-level assemblies for its putatively parental species. Furthermore several transcriptome-based gene expression analysis strengthens the quality of the paper. I have some comments listed below that are need to be addressed.

Major issue:
As confirmation of the hybrid origin of B. blakeana is one of most interesting topics, I would suggest to add a further analysis regarding the issue. Although the genome sequences of chloroplasts were used in this study, the comparison of chloroplast genome sequences is not enough. Inference of hybridization still needs to be conducted using the whole genome data. Due to the difficulties in obtaining population genomic data, I recommend to use phytop, a software based on astral tree, which can be used to detect the hybridization in a preliminary manner (see: Hong-Yun Shang et al., Phytop: A tool for visualizing and recognizing signals of incomplete lineage sorting and hybridization using species trees output from ASTRAL, Horticulture Research, 2024;, uhae330, https://doi.org/10.1093/hr/uhae330).
R: We thank the reviewer for the insightful suggestion to further validate the hybrid origin of B. blakeana using whole-genome data. Following the recommended approach, we conducted phylogenomic analyses with two distinct datasets to investigate incomplete lineage sorting (ILS) and introgression (IH) signals:
Dataset 1: 214 Single-Copy Orthologs from 13 Species
This dataset included the 13 species used in our comparative genomics analysis. However, no significant ILS/IH signals were detected between the parental species (B. purpurea and B. variegata) or within the Bauhinia and Cercis genus nodes. We attribute this result to the limited number of orthologs and the potential evolutionary distance between the selected species, which may obscure hybridization signals.

Dataset 2: 2,360 Single-Copy Orthologs from 6 Taxa
To increase resolution, we analyzed a larger dataset comprising B. purpurea, B. variegata, the two haplotypes of B. blakeana (Hmat and Hpat), C. canadensis, C. chinensis, and A. thaliana as the outgroup.
Despite the clear phylogenetic clustering of haplotypes with their respective parental species, no significant ILS/IH signals were detected at deeper nodes (e.g., Bauhinia or Cercis genus nodes). This suggests that the hybridization event leading to B. blakeana was likely a single, recent occurrence rather than a result of recurrent gene flow.

We have integrated the results from Dataset 2 into the revised manuscript (Pages 18–19, Figure 3D), including a detailed description of the phylogenomic analysis and its implications for the hybrid origin of B. blakeana.

Minor issues:
1) "high mapping rate (95.72% and 99.29%) and sequencing coverage (94.88% and 94.17%)", Dose the sequencing coverage here refers to the percentage of the genome sequence that was covered by WGS reads? How much x depth of these coverage

Powered by Editorial Manager® and ProduXion Manager® from Aries Systems Corporation

data?
R: Yes, the sequencing coverage refers to the proportion of bases sequenced (covered by WGS reads) relative to the total genome size. The mean depth of coverage was 44.93x for B. purpurea and 61.83x for B. variegata, as detailed in Supplementary Table S2. These two assemblies were constructed using stLFR data, which is distinct from the WGS dataset used for assembly quality evaluation. During the calculation of the coverage ratio, we applied a quality filter (-q 10) in the BamDeal software to exclude low-quality WGS reads. Below is a summary of the relevant data:
Reads Bauhinia purpureaBauhinia variegata
WGSNumber of reads173,500,900 200,296,314
Mapping rate95.72%99.29%
Mean depth44.9361.83
Coverage Rate94.88%94.17%
We have also clarified these points in the revised manuscript (Page 10)

2) Page 16, Figure 3a shows a large gap starting at approximately position 152k. Is this gap indicative of a sequence missing in NC061218 compared to the sequence in this study? If this is due to a structural variation, then this should be explained in the text.
R: We appreciate the reviewer’s attention to detail for identifying this inconsistency. To address this, we re-examined the original data and re-ran the mVISTA comparison with refined parameters. The revised Figure 3A confirms that the sequences of our assembled B. purpurea chloroplast genome and the published NC061218 are fully collinear, with no evidence of structural variations or missing sequences at this position.

3) Page20，"showing higher expression levels in B. variegata during the parental species comparison"，What does this statement mean? Is it saying that the DEGs of B. blakeana vs B. purpurea are similar to those of B. variegata vs B. purpurea? "Similar patterns were also found in the comparison between B. blakeana and B. variegata," where are the data, results or images for this?
R: To clarify, the statement indicates that the KEGG pathways enriched in up-regulated DEGs in B. blakeana (vs B. purpurea) overlapped with the pathways enriched in up-regulated DEGs in B. variegata (vs B. purpurea), as highlighted by arrows in Figure 5B. This functional overlap suggests that B. blakeana exhibits expression patterns resembling the higher-expressing parent (B. variegata) in these pathways. Similarly, when comparing B. blakeana vs B. variegata, we observed overlapping KEGG terms between up-regulated DEGs in B. blakeana and those enriched in B. purpurea (vs B. variegata), further supporting the functional high-parent dominance pattern. These results are presented in: Figure 5B: Arrows and color-coding (red for B. purpurea-dominant terms, blue for B. variegata-dominant terms) explicitly show the overlapping pathways; Supplementary Table S14, S15: Full lists of enriched KEGG terms for all comparisons.
We recognize that the original text could have been more explicit. In the revised manuscript, we have rephrased this section to emphasize functional convergence rather than direct gene-level overlap (Page 22).

4) page 21,"2,607 and 2,717 were up-regulated in B. blakeana when using B. purpurea and B. variegata as references," down-regulated in B. blakean could be also important and why only the up-regulation was analyzed here?
R: To clarify, both up- and down-regulated DEGs in the B. blakeana vs mid-parent value (MPV) comparisons were analyzed for their functional implications. KEGG enrichment analyses were performed separately for: all DEGs (up- and down-regulated combined); up-regulated DEGs and down-regulated DEGs. The complete results are provided in Supplementary Table S16.
In the original text, we focused on up-regulated DEGs to highlight the functional categories potentially contributing to heterosis. However, we fully agree that down-regulated DEGs are equally important. For example, down-regulated genes in B. blakeana were enriched in pathways such as “Photosynthesis” and “Biosynthesis of secondary metabolites” (Supplementary Table S16B, E), suggesting potential energy reallocation towards heterosis-related traits.
In the revised manuscript, we have added a discussion of down-regulated DEGs to provide a more balanced perspective (Page 23).

5) page22, What is ASE? The acronym needs to be explained when appearing for the

Powered by Editorial Manager® and ProduXion Manager® from Aries Systems Corporation

first time. "6,934 genes as ASEGs", according to the diagnostic SNP table, did these ASE Genes refer to genes which reads can be uniquely align to maternal or paternal alleles?
Interestingly, did the ASEGs overlap significantly with the differentially expressed genes mentioned in page20?: "Notably, the number of DEGs between B. blakeana and B. variegata was approximately twice as large as the number of DEGs between B. blakeana and B. purpurea, suggesting a stronger resemblance in the gene expression profile of B. blakeana to its maternal parent, B. purpurea (Figure 5A)." Also, is there a significant overlap between ASEGs and the MPV-hybrid DEGs in the previous paragraph?

R: Below, we address each point in detail:
1.Definition of ASE: Allele-specific expression (ASE) refers to the phenomenon where the two alleles of a gene in a diploid organism exhibit differential expression. This term is now defined upon its first mention in the Background section (Page 8).

2.ASEG Identification: The 6,934 ASEGs were identified using the HyLiTE pipeline, which was a diagnostic SNP-based method. In brief, RNA-seq reads from B. blakeana were aligned to the B. purpurea reference genome (cds sequences), and diagnostic SNPs fixed between B. purpurea and B. variegata were used to assign reads to maternal or paternal alleles. Reads uniquely assigned to either allele was used to manually construct an expression matrix for DESeq2 analysis. Full methodological details were also revised and provided in the Methods section (Page 48-49).

3.Overlap Between ASEGs and DEGs/ MPV-hybrid DEGs: We acknowledge the reviewer’s interest in exploring potential overlaps between ASEGs and DEGs. However, the reference genome dependency complicates direct comparisons. The DEGs identified in B. blakeana vs parental species comparisons (Page 21) and ASEGs (Page 25-26) were analyzed using different reference genomes (B. purpurea/B. variegata for DEGs vs B. blakeana metagenome for ASEGs). This discrepancy prevents direct overlap analysis at the gene level.

6) page23，"Furthermore, GO enrichment analysis of these ASEGs provided valuable insights into their functional implications" Why was only GO analysis performed here and not KEGG analysis? For the two methods of identifying ASEGs, dose the first method utilizes snp specific to Hmat and Hpat? How does the second method that uses DESeq2 to identify ASEGs? Please give a more detailed description on these methods.
R: Below, we address each point in detail:
1.GO vs KEGG Enrichment Analysis:
To address this point, we have now incorporated KEGG pathway enrichment analysis into the ASEG functional characterization (Page 26). These findings complement the GO analysis and highlight the involvement of ASEGs in critical processes such as DNA repair, stress adaptation, and metabolic regulation.

2.Methodological Details for ASEG Identification
Method 1: Diagnostic SNP-Based ASEG Identification:
RNA-seq reads from B. blakeana and its parents (B. purpurea and B. variegata) were aligned to the B. purpurea reference genome. HyLiTE identified diagnostic SNPs (positions fixed for alternative alleles between the parents) to distinguish maternal and paternal alleles in the hybrid. Reads uniquely mapped to either maternal or paternal alleles were extracted and quantified. DESeq2 was applied to allelic counts to identify ASEGs.
Method 2: Haplotype-Resolved ASEG Identification:
A metagenome was constructed by combining the Hmat and Hpat haplotypes of B. blakeana. RNA-seq reads from B. blakeana were aligned to the metagenome, and allele-specific counts were quantified using featureCounts. DESeq2 was applied to the allele-specific count matrix to identify ASEGs.

Method 1 relies on diagnostic SNPs between the parental genomes, which may miss allele-specific events in regions with low SNP density or high sequence divergence. Method 2 leverages haplotype-resolved assemblies, providing a more comprehensive view of allele-specific expression across the entire genome.

More detailed descriptions of these methods are added in the Methods section (Page 48-49) as recommended.

7) Page 29, ASEGs were mentioned on page 22 as well. Are the identification methods the same for both cases (page22 and 29)?
R: We thank the reviewer for this critical question. The ASEG identification methods in both sections utilized the same data set (RNA-seq reads aligned to the haplotype-resolved metagenome to generate an allelic count matrix), the analytical thresholds were purposefully tailored to address distinct biological questions:
1.Genome-wide ASEG identification (Page 25-26):
A stringent statistical framework was applied using DESeq2 ($P < 0.01$, $|log2FC| > 1$) to identify ASEGs with robust genome-wide significance. This approach prioritized minimizing false positives across the entire transcriptome.
2.Pathway-specific ASEG profiling (Page 31):
We relaxed the statistical threshold to capture ASEGs with reproducible allelic biases (maternal/paternal ASE ratio >0.7 or <0.3 in ≥2 replicates), even if they did not meet genome-wide significance. This adjustment aimed to expand the candidate pool of ASEGs within functionally critical pathways, enabling a more comprehensive analysis of allele-specific contributions to flower color. Prioritize biological relevance in key metabolic pathways where subtle allelic imbalances may disproportionately impact phenotypic outcomes.

We have revised the Methods and Results sections to explicitly distinguish these two methodologies (Page 31, Page 49-50).

8) Page 32, "we found that even though B. blakeana exhibits flower color that is more similar to its paternal parent, the general gene expression profile of B. blakeana aligns more closely with its maternal parent." "We observed that the number of genes exhibiting up-regulated expression in B. blakeana is comparatively lower than the number of genes showing down-regulated expression when compared to the MPV." These statements need to be supported by citing results.
R: We have revised the text to explicitly cite supporting results (Page 34).

9) Page 33, "The pronounced variations in expression levels observed within the carotenoid biosynthesis pathways between the parental species may be associated with the higher proportion of genes exhibiting dominance complementation or over-dominance expression patterns in B. blakeana," I do not understand why the variations in expression levels between the parents are related to the dominance expression patterns in B. blakeana? Should it not be the expression differences between B. blakeana and its parents that are related to the dominance expression patterns?
R: To clarify the link between parental expression divergence and hybrid dominance patterns, we have revised the text and provided additional mechanistic insights based on recent literature (Page 36).

In Figure 7, the metabolic processes of the pathway are shown. An analysis of maternal and paternal allele dominance as well as ASE may be added to discuss what potential consequences might arise in the metabolism related to flower color? Which metabolites or processes in pigment biosynthesis might be enhanced or weakened due to allele dominance, and what impact could this have on phenotypes such as flower color? How are dominance expression patterns and ASE related to heterosis? Which genes in these pathways belong to dominance complementation, and which belong to over-dominance?
What specific roles might ASE play? Overall, it is too simple and preliminary to state that ASE and dominance expression patterns are related to heterosis.
R: We thank the reviewer for emphasizing the need to deepen our analysis of ASE and dominance patterns in the context of metabolic pathways. We have addressed these points as follows:
1.In Figure 7A-C, we have highlighted genes exhibiting maternal allele dominance (bold italic gene copy ID in red) and paternal allele dominance (bold italic gene copy ID in blue). In Figure 7D, we marked genes with high-parent dominance (pink dot behind the gene ID) and over-dominance (yellow dot behind the gene ID) to clearly distinguish their expression patterns. We have also listed the gene in the result part (Page 29). These annotations provide a comprehensive view of how allele-specific regulation and

Powered by Editorial Manager® and ProduXion Manager® from Aries Systems Corporation

	dominance patterns are distributed across the carotenoid, anthocyanin, and chlorophyll biosynthesis pathways.<br>2.We have expanded the discussion in the revised text (Pages 36–37) to address how dominance expression patterns and ASE influence metabolic flux and likely contribute to flower color heterosis.<br><br>In addition, the text mentioned "The carotenoid biosynthesis pathway displayed the highest proportion of ASEGs, consistent with the higher proportion of genes exhibiting dominance complementation or over-dominance expression patterns in this pathway." However, in the heatmap, many gene expressions in BLA seem weaker than those in VAR, which seems to contradict the patterns of dominance expression.<br>R: We have revised the manuscript and removed the mentioned text. However, based on Figure 7D, we observed that within the carotenoid biosynthesis pathway (including "MEP Pathway", "Carotene Biosynthesis", and "Xanthophylls Biosynthesis"), there are 11 genes displaying dominance complementation or over-dominance expression patterns, as indicated by the presence of pink and yellow dots. The heatmap representation of these genes aligns with the expected dominance complementation or over-dominance expression patterns.
Additional Information:	
Question	Response
Are you submitting this manuscript to a special series or article collection?	No
**Experimental design and statistics**<br><br><br>Full details of the experimental design and statistical methods used should be given in the Methods section, as detailed in our Minimum Standards Reporting Checklist. Information essential to interpreting the data presented should be made available in the figure legends.<br><br><br>Have you included all the information requested in your manuscript?	Yes
**Resources**<br><br><br>A description of all resources used, including antibodies, cell lines, animals and software tools, with enough information to allow them to be uniquely identified, should be included in the Methods section. Authors are strongly encouraged to cite Research Resource Identifiers (RRIDs) for antibodies, model organisms and tools, where possible.<br><br><br>Have you included the information requested as detailed in our Minimum	Yes

Powered by Editorial Manager® and ProduXion Manager® from Aries Systems Corporation

Standards Reporting Checklist?	
**Availability of data and materials**<br><br>All datasets and code on which the conclusions of the paper rely must be either included in your submission or deposited in publicly available repositories (where available and ethically appropriate), referencing such data using a unique identifier in the references and in the "Availability of Data and Materials" section of your manuscript.<br><br>Have you have met the above requirement as detailed in our Minimum Standards Reporting Checklist?	Yes
GigaScience has policies and guidelines in place for the use of generative AI-writing tools such as ChatGPT. If you have used such writing tools to assist with writing the manuscript this must be declared and cited in the text. Authors should not list AI-writing tools and other AI-assisted technologies as an author or co-author and should acknowledge that they are fully responsible for text generated or refined by AI-writing tools.<p><br><br>A summary of use (particularly in the introduction or among methods) needs to be included at the end of the paper, and the outputs should also be included as a supplementary file hosted in GigaDB or other open repositories. Please read our guidelines for more information. <p><br><br>By submitting to GigaScience, you are aware of the journal's AI-writing tools policy, and if you have declared use of such tools below, you have acknowledged this where appropriate in your manuscript and have made a summary of use and outputs available. <p><br><b>AI-assisted writing tools have been used in the preparation of this manuscript?	No

Powered by Editorial Manager® and ProduXion Manager® from Aries Systems Corporation

Manuscript

Click here to access/download;Manuscript;Bauhinia_manuscript-

The Haplotype-resolved T2T Genome for *Bauhinia x blakeana* Sheds Light on the Genetic Basis of Flower Heterosis

Weixue Mu[1,2,†], Joshua Casey Darian[3,†], Wing-Kin Sung[4,5,6,†], Xing Guo[7], Tuo Yang[8], Mandy Wai Man Tang[1], Ziqiang Chen[9], Steve Kwan Hok Tong[10,11], Irene Wing Shan Chik[10], Robert L Davidson[12], Scott C Edmunds[13], Tong Wei[7,*], Stephen Kwok Wing Tsui[1,2,*].

[1] School of Biomedical Sciences, The Chinese University of Hong Kong, Shatin, N. T., Hong Kong

[2] Hong Kong Bioinformatics Centre, The Chinese University of Hong Kong, Shatin, N.T., Hong Kong

[3] School of Computing, National University of Singapore, 13 Computing Drive, Block COM1, Singapore 117417

[4] Department of Chemical Pathology, The Chinese University of Hong Kong, Shatin, N.T., Hong Kong

[5] JC STEM Laboratory of Computational Genomics, Li Ka Shing Institute of Health Sciences, The Chinese University of Hong Kong, Shatin, N.T., Hong Kong

[6] Hong Kong Genome Institute, Hong Kong Science Park, Shatin, N.T., Hong Kong

[7] BGI Research, East Lake High-Tech Development Zone, Wuhan 430074, China

[8] Key Laboratory of Southern Subtropical Plant Diversity, Fairy Lake Botanical Garden, Shenzhen & Chinese Academy of Sciences, Shenzhen 518004, China

[9] National Key Laboratory for Germplasm Innovation & Utilization of Horticultural Crops, College of Horticulture & Forestry Sciences, Huazhong Agricultural University, Wuhan 430070, China

[10] BGI Genomics, 16 Dai Fu Street, Tai Po Industrial Estate, Tai Po, N.T., Hong Kong

[11] International DNA Research Centre, 576-586 Castle Peak Road, Cheung Sha Wan, Kowloon, Hong Kong

[12] School of Physics, Engineering & Computer Science, University of Hertfordshire, Hatfield, AL10 9AB, United Kingdom

[13] GigaScience Press, BGI Hong Kong Tech Co. Ltd., 50 Bonham Strand Sheung Wan, Hong Kong

[*] Corresponding authors

Tong Wei, weitong@genomics.cn; Stephen Kwok Wing Tsui, kwtsui@cuhk.edu.hk.

†These authors contributed equally.

Weixue Mu [0000-0002-2675-261X]; Joshua Casey Darian; Wing-Kin Sung [0000-0001-7806-7086]; Xing Guo [0000-0003-2046-9773]; Tuo Yang [0000-0002-2147-2475]; Mandy Wai Man Tang; Ziqiang Chen; Steve Kwan Hok Tong; Irene Wing Shan Chik; Robert L Davidson [0000-0002-0311-9774]; Scott C Edmunds [0000-0001-6444-1436]; Tong Wei [0000-0002-2692-7192]; Stephen Kwok Wing Tsui [0000-0003-0686-4259]

Abstract

Background

The Hong Kong Orchid Tree *Bauhinia x blakeana* Dunn has long been proposed to be a sterile interspecific hybrid exhibiting flower heterosis when compared to its likely parental species, *B. purpurea* L. and *B. variegata* L. Here, we report comparative genomic and transcriptomic analyses of the three *Bauhinia* species.

Findings

We generated chromosome-level assemblies for the parental species and applied a trio-binning approach to construct a haplotype-resolved telomere-to-telomere (T2T) genome for *B. blakeana*. Comparative chloroplast genome analysis confirmed *B. purpurea* as the maternal parent. Transcriptome profiling of flower tissues highlighted a closer resemblance of *B. blakeana* to its maternal parent. Differential gene expression analyses revealed distinct expression patterns among the three species, particularly in biosynthetic and metabolic processes. To investigate the genetic basis of flower heterosis observed in *B. blakeana*, we focused on gene expression patterns within pigment biosynthesis-related pathways. High-parent dominance and over-dominance expression patterns were observed, particularly in genes associated with carotenoid

biosynthesis. Additionally, allele-specific expression analysis revealed a balanced contribution of maternal and paternal alleles in shaping the gene expression patterns in *B. blakeana*.

Conclusions

Our study offers valuable insights into the genome architecture of hybrid *B. blakeana*, establishing a comprehensive genomic and transcriptomic resource for future functional genetics research within the *Bauhinia* genus. It also serves as a model for exploring the characteristics of hybrid species using T2T haplotype-resolved genomes, providing a novel approach to understanding genetic interactions and evolutionary mechanisms in complex genomes with high heterozygosity.

Keywords

Bauhinia x blakeana, trio-binning, genome evolution, transcriptome profiling, flower heterosis

Background

Bauhinia x blakeana Dunn, commonly known as the Hong Kong Orchid Tree, is a popular ornamental tree species admired for its striking purplish orchid-like flowers and extended blooming period. Its initial discovery traced back to a chance discovery by French horticulturalist Jean-Marie Delavay on Hong Kong Island in the 1880s, where it was later determined to be completely sterile and grown solely by vegetative propagation [1]. In 1908, due to its distinctive characteristics, it was proposed as a new

species [2]. With the species name honoring the former Governor of Hong Kong Sir Henry Blake, it has subsequently been made the emblem of the Hong Kong Special Administrative Region. However, the taxonomic status and precise origin of *B. blakeana* remains uncertain and curious because of its sterility. Due to this complete sterility, artificial propagation methods are conventionally required for *B. blakeana*, often involving cutting or grafting onto rootstocks of other *Bauhinia* species. Considering its limited natural occurrence and dependence on artificial cultivation, *B. blakeana* is usually regarded as a horticultural cultivar rather than a naturally existing species.

The sterility of *B. blakeana* has prompted the hypothesis that it may be an interspecific hybrid between *Bauhinia purpurea L.* and *Bauhinia variegata L.*, a proposition first proposed by de Wit based on the shared morphological similarities among the three species [3]. The potential for hybridization between *B. purpurea* and *B. variegata* is further supported by their coexistence across much of their distribution ranges, partially overlapping flowering periods, xenogamous nature, and shared common pollinators [1]. Moreover, the consistent diploid chromosome number of 2n = 28 across all three species indicates that the genesis of *B. blakeana* likely involved the integration of one set of chromosomes from each parent, mirroring that of both parents and ruling out the idea of *B. blakeana* as a sterile polyploid [4]. It is worth noting that the genus *Bauhinia*, a member of Cercidoideae, one of the six subfamilies of Leguminosae, stands as the largest genus within the subfamily and appears to have arisen from an allotetraploid

merger, exhibiting double the chromosome count observed in the earlier-diverging genus *Cercis* [5]. In some instances, wide hybridization is succeeded by the following genome doubling, a phenomenon that can potentially restore fertility to the initial potentially sterile wide hybrid. However, the 2n genome of *B. blakeana* is likely the reason causing irregular chromosome segregation and abnormal spindle formation during microsporogenesis, ultimately resulting in its complete sterility [6]. Previous research, encompassing morphological, karyotypic, and molecular analyses including the utilization of ISSR markers and sequencing of key genetic regions (rbcL, atpB-rbcL intergenic spacer, ITS1), have provided evidence supporting this rare interspecific hybridization event [1, 4, 6, 7]. While these discoveries provide valuable insights, there remains a lack of definitive confirmation, especially at the genomic level. Despite the significant horticultural, cultural, and historical value of *B. blakeana*, our understanding of its biology remains limited primarily due to the absence of its genomic information.

Recent advancements in genome sequencing technologies, along with innovative bioinformatic approaches, have revolutionized our capacity to generate high-quality genomes for various plant species, including those with high levels of heterozygosity [8-10]. The availability of these high-quality genomes serves as a foundation for understanding the origin and evolutionary history of plants, as well as unraveling the genetic mechanisms governing essential traits. Additionally, novel methodologies such as high-throughput/resolution chromosome conformation capture (Hi-C) and assembly algorithms like trio-binning have emerged as powerful tools, enabling the construction

of haplotype-resolved genomes [11-13]. The historical and cultural interest of Hong Kong *Bauhinia* led to a community-crowdfunded genome project to try to answer some of the questions on the species' origin [14], but it only raised enough money to sequence the transcriptomes of the three key species [15]. Telomere-to-telomere (T2T)-level assembly completeness and haplotype-level resolution offer significant advantages in identifying genetic variations, particularly in the study of hybrid heterosis. It allows precise tracking and analysis of genetic variations across parental lines and their hybrid offspring, thereby facilitating a comprehensive understanding of the underlying genetic mechanisms.

Heterosis, also known as hybrid vigor, refers to the phenomenon in which hybrid offspring display enhanced or superior traits compared to their parents. When comparing the flower phenotype of *B. blakeana* to its putative parental species, *B. purpurea* and *B. variegata*, distinct characteristics such as more vibrant flower color, larger flower size, and an extended flowering period are observed, suggesting the presence of heterosis. Heterosis has been extensively studied and utilized in crop breeding [16-18]. However, the genetic basis of this phenomenon remains incompletely understood. Classical hypotheses, including dominance complementation, over-dominance, and epistasis, have been proposed to explain the genetic mechanisms underlying heterosis [17-19]. Transcriptome profiling is commonly employed to investigate heterosis at the transcriptional level, as gene expression plays a pivotal role in linking DNA sequence variation to resulting phenotypic diversity. Several modes of

gene expression differences between parents and hybrids have been suggested as contributing factors to heterosis, including additivity/non-additivity, high-/low-parent dominance, and over-/under-dominance [20]. Gene expression is a complex process regulated by a combination of genetic and epigenetic variations, involving the interplay of various genomic elements, including cis-acting elements, trans-acting factors, their intricate interactions, as well as other epigenomic factors [21, 22]. Moreover, allele-specific expression (ASE) introduces an additional layer of complexity to the genetic underpinnings of heterosis by elucidating the differential mRNA abundance (gene expression imbalance) between alleles in diploid (or higher-ploidy) organisms [23, 24].

In this study, we presented chromosome-level genome assemblies for the three *Bauhinia* species and employed a trio-binning strategy to reconstruct the high-quality haplotypes of the hybrid *B. blakeana* with gapless T2T completeness. The adoption of T2T genomes has significantly advanced genomics research by providing a detailed depiction of each chromosome from end to end, known as 'telomere-to-telomere'. It enhances our ability to characterize genomic structure and variations, particularly in regions rich in repetitive sequences, providing insights into mechanisms and genomic evolution while elucidating the genetic underpinnings of specific traits. Leveraging our haplotype-resolved T2T genome, through an integrated approach encompassing comparative genomics, transcriptomics, and ASE analyses, we have gained valuable insights into the evolutionary dynamics of *Bauhinia* species and shed light on the genetic basis underlying the intriguing biology of *B. blakeana*. Our haplotype-resolved

T2T genome serves as a valuable reference for studying genomes with high heterozygosity, particularly in analyzing the traits of hybrid genomes. It also provides a clear roadmap for future studies, facilitating key discoveries of biosynthetic genes essential for synthetic biology applications.

Results

Sequencing and assembly of the three *Bauhinia* genomes

We employed a multi-platform sequencing strategy, combining single-tube Long Fragment Read (stLFR), BGI-SEQ short-read (whole genome sequencing, WGS), Oxford Nanopore Technologies (ONT) long-read, and Hi-C sequencing methods to obtain high-quality genome assemblies for the three *Bauhinia* species (Supplementary Table S1). We first conducted *k*-mer analyses [25] for all three species to survey their overall genome characteristics. The genome size of *B. purpurea*, *B. variegata,* and *B. blakeana* was estimated to be ~303.68 Mb, ~314.49 Mb, and ~290.97 Mb, with a heterozygosity ratio of 0.60%, 0.24%, and 4.64%, respectively (Supplementary Figure S1).

We performed assembly of the stLFR reads using the Supernova assembler [26] to generate draft genome assemblies for the parental species *B. purpurea* and *B. variegata*. This process yielded two assemblies with genome sizes of approximately 285.15 Mb and 311.01 Mb, respectively, closely matching their estimated genome sizes (Table 1). Subsequently, we utilized Hi-C data to anchor the two initial assemblies onto 14

pseudochromosomes, achieving high anchor rates of 99.98% for both parental species. The resulting assemblies exhibited scaffold N50 values of 21.60 Mb for *B. purpurea* and 24.40 Mb for *B. variegata*. (Table 1; Figure 1A; Supplementary Figure S2) The completeness of the assemblies was assessed using 1614 conserved embryophyte proteins from the BUSCO [27]. The analysis revealed a high level of completeness, with 97.8% for *B. purpurea* and 98.4% for *B. variegata*, respectively. To evaluate the quality of the genome assemblies, we calculated the mapping rate and sequencing coverage using WGS data. The mapping rate was 95.72% for *B. purpurea* and 99.29% for *B. variegata*, with coverage rates of 94.88% and 94.17%, respectively (Supplementary Table S2). These high values provide strong evidence of consistency between the assemblies and the WGS short reads, confirming the high accuracy of our assemblies.

To overcome the challenges posed by the high heterozygosity of the *B. blakeana* genome, we further generated ~17.66 Gb ONT long-reads for assembly. Employing a trio-binning approach, we categorized all sequencing reads into three groups: paternal reads, maternal reads, and ambiguous reads. Subsequently, we applied hypo-assembler [28] in haploid mode to assemble each haplotype, with paternal and ambiguous reads, and with maternal and ambiguous reads, respectively. The resulting two sets of high-quality, gap-free haplotypes, hereafter referred to as Hmat and Hpat, represent the maternal and paternal haplotypes of the allodiploid *B. blakeana* genome. Hmat exhibits a genome size of ~275.48 Mb, with a contig N50 value of 19.54 Mb, while Hpat has a

size of ~290.70 Mb, with a contig N50 value of 20.99 Mb. (Figure 1B; Table 1). We used Merqury [29] to evaluate the phasing quality of the two *B. blakeana* haplotypes by comparing *k*-mers from parental read sets to the *k*-mers in each of the haplotype-resolved assemblies. We estimated QV scores of 40.46 for Hmat, 45.64 for Hpat, and 42.39 for the combined set of sequences (Supplementary Table S3A). We counted the number of expected haplotype-specific *k*-mers (hap-mers) present in the corresponding haplotype assemblies and found that the maternal and paternal haplotypes recovered 92.21% and 95.82% of the expected hap-mers, respectively (Supplementary Table S3B). The maternal haplotype Hmat contains 1.74% paternal hap-mers, while the paternal haplotype Hpat contains 1.03% maternal hap-mers. These discrepancies likely arose from switch errors or base-pair errors. The *k*-mers completeness for Hmat, Hpat, and the combined diploid assembly were estimated to be 58.57%, 60.28%, and 93.67%, respectively. The result indicates that approximately 40% of the *k*-mers were haplotype-specific, highlighting the high heterozygosity in *B. blakeana* (Supplementary Table S3C). The haplotype evaluation results align with a haplotype-resolved genome assembly, indicating a satisfactory resolution of both haplotypes within the *B. blakeana* genome assembly. We further evaluated the completeness of Hmat and Hpat using BUSCO, resulting in a high completeness score of 99.0% for Hmat and 99.2% for Hpat.

Genome annotation reveals repeat and gene landscapes

Utilizing our four high-quality assemblies, we conducted annotations of repetitive elements and protein-coding genes to examine the repeat and gene landscape of the

three *Bauhinia* species. Our analysis revealed varying percentages of repetitive elements in each assembly. Specifically, we found that Hpat contains 32.21% repetitive elements, followed by *B. purpurea* with 27.92%, *B. variegata* with 27.38%, and Hmat with 25.32% (Supplementary Table S4). Among these repetitive elements, LTR retrotransposons were the most prevalent in all four assemblies (Figure 1A, B).

We identified 37,804, 37,956, 38,735, and 40,111 protein-coding genes in Hmat, Hpat, *B. purpurea*, and *B. variegata*, respectively. Notably, a high percentage of these genes, 99.98%, 99.97%, 99.98%, and 99.96%, could be functionally annotated against at least one of the six databases searched, namely Nr, SwissProt [30], KEGG [31], KOG [32], TrEMBL [30], and InterPro [33] (Table 1). Moreover, we found that the gene number, gene length, CDS length, exon number, exon length, and intron length showed comparable characteristics across the four assemblies. The predicted gene sets for Hmat, Hpat, *B. purpurea*, and *B. variegata* were evaluated using BUSCO, yielding respective scores of 94.2%, 96.4%, 97.4%, and 97.8%. These results indicate a high level of functional completeness in the annotated proteomes, accurately representing the corresponding genomes.

We further predicted non-coding RNAs (ncRNAs) including miRNA, tRNA, rRNA, and snRNA (Supplementary Table S5), as well as transcription factors (TFs), transcription regulators (TRs), and protein kinases (PTKs) in these assemblies (Supplementary Table S6). This comprehensive analysis provides valuable insights into

the repetitive elements, protein-coding genes, and regulatory elements present in the genomes of these three *Bauhinia* species.

<u>Structural variations in *B. blakeana* haplotype chromosomes</u>

Structural variation (SV) encompasses a diverse range of genomic alterations, including inversions, translocations, and duplications, which can significantly impact the organization and structure of the genome. In our study, we investigated SVs in the haplotype chromosomes of *B. blakeana*, which might be associated with its high genome heterozygosity and observed sterility. By utilizing repeat and gene annotations from previous analyses in conjunction with the quarTeT prediction software [34], we identified putative centromeres for each of the 14 pseudochromosomes in both Hmat and Hpat. The centromeres exhibited variable lengths, ranging from 101.60 Kb to 1.48 Mb in Hmat, and from 143.29 Kb to 2.79 Mb in Hpat (Figure 1C; Supplementary Table S7). Additionally, we searched for the presence of the telomere repeat motif "TTTAGGG" along each of the haplotype assembly chromosomes. This allowed us to identify 27 potential telomeric regions in Hmat, with motif repeat numbers ranging from 12 to 848, as well as 27 potential telomeric regions in Hpat, with motif repeats ranging from 31 to 1,117 (Figure 1C; Supplementary Table S8). Notably, except for chromosome 8 in both the Hmat and Hpat assemblies, each chromosome displayed telomeres at both ends, indicating complete reconstruction to a gapless and telomere-to-telomere (T2T) level. Using the SyRI tool [35], we detected a total of 424 SVs between Hmat and Hpat, including 12 inversions (totaling 180.38Kb), 30 translocations

(totaling 655.61Kb), and 382 duplications (totaling 3.81Mb) (Figure 1C; Supplementary Table S9). The relatively low number of observed SVs could be attributed to factors such as high synteny between the two parental species of *B. blakeana* or limitations in SV detection methods.

Comparative genomics reveals evolutionary dynamics in *Bauhinia*

To investigate the relationships and evolutionary history of *Bauhinia* species, we performed comparative genomic analyses involving the *Bauhinia* genomes (*B. purpurea* and *B. variegata*) and 13 other selected representative plant species. The selected species included 9 Fabaceae species from different subfamilies (*S. tora, L. albus, G. max, L. japonicus, M. truncatula, B. purpurea, B. variegata, C. canadensis, C. chinensis*), and 6 other eudicot species (*V. vinifera, C. mollissima, P. persica, P. trichocarpa, A. thaliana, C. canephora*) (Supplementary Table S10). To minimize potential impacts on the results, the sterile hybrid *B. blakeana* was excluded from the evolutionary analyses. Through gene clustering analysis, we identified 17,904 gene families in *B. purpurea* and 18,095 gene families in *B. variegata*. Across all 15 species, we identified 213 single-copy gene families shared among them. Subsequently, a maximum likelihood phylogenetic tree was constructed by combining all the genes within these single-copy gene families (Figure 2A; Supplementary Table S11). The topology of the generated phylogenetic tree was consistent with previous research findings [5]. Molecular dating analysis estimated the divergence of the *Bauhinia* genus from the common ancestor with Cercis to have occurred approximately 57.1 million

years ago (Mya), followed by the divergence of *B. purpurea* and *B. variegata* around 13.4 Mya.

We used the birth-and-death model to identify expanded and contracted gene families within the selected plant species by comparing them to gene families in their most recent common ancestor (MRCA). *B. purpurea* exhibited 1,138 gene family expansions and 313 gene family contractions, while *B. variegata* had 1,456 expanded and 284 contracted gene families. Examining the entire *Bauhinia* genus, we identified 5,037 expanded gene families and 259 contracted gene families compared to their MRCA (Figure 2A). These 5,037 expanded gene families showed significant enrichment in the KEGG biosynthesis pathways related to bioactive compounds, including monoterpenoid, diterpenoid, flavonoid, terpenoid backbone, and carotenoid (Figure 2B, Supplementary Table S12A). Notably, similar enrichment patterns were also observed in the expanded gene families of *B. purpurea* and *B. variegata*. Specifically, in *B. purpurea*, expanded gene families were enriched in KEGG terms such as "plant-pathogen interaction", "Isoflavonoid biosynthesis", "Flavone and flavonol biosynthesis", and “Monoterpenoid biosynthesis”. On the other hand, in *B. variegata*, expanded gene families were enriched in terms such as “Phenylpropanoid biosynthesis", Flavonoid biosynthesis" and "Sesquiterpenoid and triterpenoid biosynthesis" (Supplementary Table S12B-C).

Evolution and expansion of terpene synthase genes in *Bauhinia*

Terpenes and terpenoids encompass a large and diverse group of natural compounds with multiple functions in plants. Terpene synthases (TPSs) are key enzymes responsible for the biosynthesis of terpenoids. These TPS proteins play crucial roles in plant growth, development, and in enhancing resistance to abiotic and biotic stress [36]. To deepen our understanding of terpenoid biosynthesis in *Bauhinia* species, we identified candidate TPSs in the *Bauhinia* species and other selected plants from the Fabaceae family that were used in our previous comparative genomic analyses. We identified 39 TPS genes in *B. purpurea*, fewer than the 47 TPS genes found in *B. variegata* (Supplementary Table S13A). Within the Fabaceae family, *B. variegata* exhibits the highest TPS gene count, followed by *C. canadensis* (46), *M. truncatula* (41), and *B. purpurea* (39). Subsequently, a phylogenetic tree containing a total of 288 TPS genes across all Fabaceae species was constructed. These TPS genes were categorized into 6 clades, denoted as TPS-a, b, c, e, f, and g, according to the established subfamily classification of TPS genes (Figure 2C) [37]. The TPS-a, b, and g collectively comprise the majority of these identified TPS genes. Within the *Bauhinia* species, TPS-b genes emerge as the most prevalent among the TPS genes, with 21 identified in *B. variegata* and 15 in *B. purpurea*, surpassing the TPS-b gene counts observed in all other species within the Fabaceae family. Following TPS-b, TPS-g genes exhibit the second-highest representation in the *Bauhinia* species, with 10 in *B. purpurea* and 9 in *B. variegata*, while TPS-a genes follow with 5 in *B. purpurea* and 7 in *B. variegata*. Notably, TPS-a, TPS-b, and TPS-g constitute clades specific to angiosperms, with TPS-

a primarily containing sesquiterpene and diterpene synthases, while TPS-b and TPS-g clade typically encode monoterpene synthases.

To investigate the origin of the increased TPS gene count in the *Bauhinia* species in comparison to other species within the Fabaceae family, we analyzed the duplication events of TPS genes. The results showed that transpositional duplication was the primary driver contributing to the expansion of TPS-b in *B. purpurea* (7, 46.67%), while proximal repeats (7, 36.84%) and tandem duplication (6, 31.58%) were the major contributors to the expansion of TPS-b genes in *B. variegata* (Supplementary Table S13B). These expanded TPS-b genes are likely to contribute to the biosynthesis of monoterpenes, consequently enhancing the antimicrobial activity within these *Bauhinia* species.

<u>Genomic and phylogenetic insights into the maternal parentage and hybrid origin of *B. blakeana*</u>

The parthenogenetic inheritance and low substitution rate of the chloroplast (cp) genome make it a valuable tool for phylogenetic analysis and determining hybrid parentage. Using our short-read sequencing data, we successfully assembled and annotated the cp genomes of three *Bauhinia* species. The complete sequences obtained were 156,100 bp for both *B. blakeana* and *B. purpurea*, and 155,415 bp for *B. variegata.* To ensure the accuracy of our assemblies, we performed comparative analyses using ClustalW alignment [38] and mVISTA software [39] to compare our

assembled sequences with the previously published cp genomes of *B. blakeana* (MN413506), *B. purpurea* (NC061218), and *B. variegata* (MT176420) (Figure 3A). Our assembled *B. blakeana* and *B. variegata* cp genomes matched published references, whereas the published *B. purpurea* genome contained a 1-bp deletion (Figure 3B). Importantly, our assembled versions of the three *Bauhinia* cp genomes demonstrated identical sequences for both *B. blakeana* and *B. purpurea*, providing strong evidence supporting *B. purpurea* as the maternal parent of *B. blakeana*.

We used a maximum likelihood (ML) model to construct a phylogenetic tree to further explore the genetic relationships among the sequenced Bauhinia species. The tree included three additional *Bauhinia* species available in the NCBI database, with *C. canadensis* serving as the outgroup. The resulting phylogenetic structure was consistent with previous research, confirming a close genetic relationship among *B. blakeana*, *B. purpurea,* and *B. variegata* (Figure 3C) [40].

To investigate the hybrid origin of *B. blakeana* more comprehensively, we expanded our study by incorporating a broader genomic perspective. Initially, a phylogenetic tree was constructed, integrating the two *B. blakeana* haplotypes alongside *B. purpurea*, *B. variegata*, *C. canadensis*, *C. chinensis*, and *A. thaliana* as an outgroup, which was inferred from 2,360 single-copy orthologous gene trees using the ASTRAL method. Subsequently, we utilized Phytop to assess the heterogeneity within the species tree by quantifying incomplete lineage sorting (ILS) and

introgression/ hybridization (IH) [41]. ILS and IH are crucial concepts in evolutionary biology that play a significant role in understanding genetic relationships and divergence patterns among species. Our results showed that *B. purpurea* and the maternal haplotype of *B. blakeana* (Hmat) formed a strongly supported monophyletic group, with ILS-e = 2.3%, ILS-i = 3.4%, IH-e = 1.0%, and IH-i = 1.1% (Figure 3D). Additionally, *B. variegata* and the paternal haplotype of *B. blakeana* (Hpat) formed another monophyletic clade, with ILS-e = 3.5%, ILS-i = 5.2%, and no detectable introgression signals (IH-e = 0.0%, IH-i = 0.0%). Notably, the ILS/IH indices for both clades were relatively low, suggesting a limited phylogenetic conflict between the parental species and their respective *B. blakeana* haplotypes. The absence of significant IH signals at the *Bauhinia* genus node further supports the hypothesis that *B. blakeana* more likely originated from a rare single, recent hybridization event rather than recurrent gene flow. The low ILS/IH indices, combined with the clear phylogenetic clustering of *B. blakeana* haplotypes with their parental species, provide genomic evidence of its hybrid origin and highlight the utility of phased haplotypes in resolving complex evolutionary histories.

<u>Transcriptomic profiling reveals flower tissue gene expression patterns</u>

To understand the gene expression dynamics among the three *Bauhinia* species, we conducted a comprehensive analysis of differential gene expressions in flower tissues. Various DEG analyses were performed, including comparisons between the parental species, comparisons between *B. blakeana* and each of the parental species, as well as

comparisons between *B. blakeana* and the mid-parent value (MPV) (Figure 4A). To ensure the reliability of our analysis, we collected three biological replicates for each *Bauhinia* species and performed RNA sequencing, generating a substantial amount of sequencing data for each sample (Supplementary Table S1). Initially, the reads of each sample were aligned to the *B. purpurea* genome to generate a read count matrix, which was then used for principal component analysis (PCA). Upon analyzing the PCA results, we observed that one sample, VAR3, exhibited an abnormal location in the PCA plot (Supplementary Figure S3). To maintain the integrity of the analysis and ensure that this outlier did not influence our results, we excluded the VAR3 sample from subsequent analyses.

To assess pre-existing differential gene expressions, we first identified DEGs between *B. purpurea* and *B. variegata*. To avoid false-negative results where the expression level is zero due to the inability to map reads to the reference genome caused by significant genetic differences between the parental genomes, we only selected genes that expressed in both species (with raw counts >= 10) for further analyses. Regardless of whether we used *B. purpurea* or *B. variegata* as the reference, a similar number of DEGs was observed (Figure 4B). Using the *B. purpurea* genome as the reference, we identified a total of 6,988 DEGs, with 3,419 (48.93%) genes up-regulated in *B. purpurea* and 3,569 (51.07%) genes up-regulated in *B. variegata* ($\log_2$|FC (fold change) | > 2; $P < 0.01$). Similarly, when selecting *B. variegata* as the reference, we identified 7,052 DEGs, with 3,435 (48.71%) DEGs exhibiting higher

expression levels in *B. purpurea* and 3,617 (51.29%) DEGs showing higher expression levels in *B. variegata* (Figure 4A, B).

To assess the functional implications of these DEGs, we performed a KEGG enrichment analysis, and the results were highly consistent regardless of the reference species used (Supplementary Table S14). Specifically, enriched KEGG terms obtained using both reference genomes included "Photosynthesis," "Ribosome," and "Carbon fixation in photosynthetic organisms", indicating differences in energy production and metabolism between the two parental species. The term "Circadian rhythm" was also enriched, suggesting possible differences in the regulation of growth and flowering timing between them. Additionally, separate KEGG enrichment analyses were conducted on the up-regulated DEGs in *B. purpurea* and *B. variegata*, respectively. The overlapping results of enriched KEGG terms for up-regulated DEGs in *B. purpurea* included “Inositol phosphate metabolism”, “ABC transporters”, “Phosphatidylinositol signaling system” and “Circadian rhythm – plant”. In contrast, the up-regulated DEGs in *B. variegata* revealed enrichment in “Ribosome”, “Photosynthesis”, and various metabolic pathways (Figure 4C).

Transcriptome divergence between *B. blakeana* and its parental species

Considering the notable phenotypic distinctions between the parental species and *B. blakeana*, our subsequent goal was to evaluate the transcriptome divergence between them, aiming to reveal any possible association with the observed flower heterosis in

B. blakeana. Using *B. purpurea* as the reference, we identified a total of 5,116 DEGs ($log_2|FC| > 2$, $P < 0.01$) between *B. blakeana* and *B. purpurea*. Among these DEGs, 2,305 (45.05%) were up-regulated in *B. blakeana*, while 2,811 (54.95%) were up-regulated in *B. purpurea* (Figure 5A). These DEGs demonstrated significant enrichment in KEGG terms such as "Plant-pathogen interaction", "Plant hormone signal transduction", and various signaling and metabolic pathways. (Supplementary Table S15A). In the comparison between *B. blakeana* and *B. variegata*, we identified a larger set of DEGs, totaling 8,981 genes, using *B. variegata* as the reference. Among these DEGs, 3,610 (40.20%) exhibited up-regulation in *B. blakeana*, while 5,371 (59.76%) genes exhibited up-regulation in *B. variegata* (Figure 5A). These DEGs were significantly enriched in KEGG terms including “Photosynthesis”, and "Plant hormone signal transduction", as well as various biosynthesis and metabolic pathways (Supplementary Table S15D). Notably, the number of DEGs between *B. blakeana* and *B. variegata* was approximately twice as large as the number of DEGs between *B. blakeana* and *B. purpurea*, suggesting a stronger resemblance in the gene expression profile of *B. blakeana* to its maternal parent, *B. purpurea* (Figure 5A). Additionally, we conducted KEGG enrichment analysis on the DEGs with up-regulated expression levels in each species during the comparisons separately. Interestingly, several KEGG pathways enriched in up-regulated DEGs in *B. blakeana* (vs *B. purpurea*) overlapped with those enriched in up-regulated DEGs in *B. variegata* (vs *B. purpurea*). A similar pattern was observed when comparing *B. blakeana* vs *B. variegata*, where up-regulated DEGs in *B. blakeana* overlapped with pathways enriched in *B. purpurea* (vs *B.*

variegata) (Figure 5B; Supplementary Table S14 B, E; Supplementary Table S15 C, F). This functional convergence suggests that *B. blakeana* retains expression patterns resembling the higher-expressing parent in these pathways.

To further investigate the gene expression patterns in *B. blakeana*, an additivity analysis was conducted to determine whether they followed an additive model, where the gene expression levels were not significantly different from the average level of parental gene expression, known as the MPV [20]. *B. purpurea* and *B. variegata* were used as references, comparing *B. blakeana* to the MPV, resulting in the identification of 7,111 and 7,287 DEGs, respectively ($\log_2|FC| > 1$; $P < 0.01$) (Figure 5C). These MPV-hybrid DEGs were defined as genes with non-additive expression patterns, attributed to allelic interactions that alter regulatory networks and consequently result in gene activity patterns distinct from the average parental values [42]. KEGG enrichment analysis of these MPV-hybrid DEGs revealed their involvement in energy conversion, utilization, and metabolic transformations. Specifically, pathways such as "Photosynthesis", "Carbon fixation in photosynthetic organisms", "Carbon metabolism", and various metabolic and biosynthetic pathways were significantly enriched (Supplementary Table S16A, D). Among these MPV-hybrid DEGs, 2,607 and 2,717 exhibited up-regulation, while 4,504 and 4,570 showed down-regulation in *B. blakeana* when using *B. purpurea* and *B. variegata* as references, respectively. The up-regulated DEGs in *B. blakeana* were enriched in pathways critical for cellular maintenance, biosynthesis, and stress responses, including “Autophagy”, “Ribosome biogenesis in eukaryotes” and

"Arginine and proline metabolism". In contrast, down-regulated DEGs were predominantly associated with primary metabolism and energy production, including "Photosynthesis", "Biosynthesis of secondary metabolites" and "Carbon fixation in photosynthetic organisms", (Supplementary Table S16B-C, E-F). Importantly, a substantial proportion of genes (67.52%: 14,780 out of a total of 21,891 expressed genes when using *B. purpurea* as a reference, and 67.09%: 14,853 out of a total of 22,140 expressed genes when using *B. variegata* as a reference) in *B. blakeana* exhibited expression levels that followed an additive model, which can be explained by the combination of gene expression from its parental species. This suggests that while certain genes in *B. blakeana* exhibit non-additive expression patterns, indicating hybrid-specific regulation, a considerable number of genes maintain an additive expression profile, reflecting the balanced contribution of both parental genomes to the gene expression in the hybrid.

<u>Allele-specific expression patterns in *B. blakeana*</u>

Expanding upon the discovery of non-additive expression patterns observed in the genes of *B. blakeana*, our study aimed to delve deeper into the underlying molecular mechanisms by identifying genes that exhibit ASE, in which case the gene expression level differed between the two alleles. To accomplish this, we employed the HyLiTE (Hybrid Lineage Transcriptome Explorer) pipeline [41], which utilizes diagnostic SNPs to assign RNA-seq reads to maternal or paternal alleles. Of the *B. blakeana* RNA-seq reads, 33.86% and 32.65% were unambiguously assigned to maternal and paternal

alleles, respectively, with no significant bias in allelic assignment (Supplementary Table S17A). Using DESeq2 on allelic read counts, we identified 6,934 ASEGs ($P < 0.01$, $|log2FC| > 1$), with nearly equal proportions of maternal (3,492, 50.36%) and paternal (3,442, 49.64%) allele dominance (Figure 6A). KEGG enrichment analysis was performed on these ASEGs, revealing significant enrichment in KEGG pathways associated with energy generation, transformation, and utilization. Enriched pathways included "Photosynthesis", "Ribosome", “Carbon metabolism” and "Oxidative phosphorylation" (Supplementary Table S17B). Besides the pathways related to energy metabolism, our analysis also identified significant enrichment of KEGG pathways associated with flower color formation, including "Anthocyanin biosynthesis" and "Porphyrin and chlorophyll metabolism". These pathways are known to play important roles in the synthesis and regulation of pigments responsible for flower coloration. Notably, the enrichment analysis of maternal allele dominance ASEGs revealed an enrichment of the "Carotenoid biosynthesis" pathway (Supplementary Table S17C). Conversely, ASEGs showing paternal allele dominance were enriched in pathways such as "Anthocyanin biosynthesis", "Flavone and flavonol biosynthesis", and "Porphyrin and chlorophyll metabolism" (Supplementary Table S17D).

However, as a portion of the *B. blakeana* RNA-seq reads (33.49%) could not be assigned using the aforementioned method, we employed a genome-wide approach to identify ASEGs. This involved identifying syntenic gene blocks between the two *B. blakeana* haplotypes, Hmat and Hpat, and identifying a total of 10,421 gene pairs within

these blocks that exhibited a one-to-one relationship within the same orthogroups, referred to as allele pairs. By mapping the *B. blakeana* RNA-seq reads to the metagenome constructed from the combined gene sets of Hmat and Hpat, we quantified allelic read counts and employed DESeq2 to identify ASEGs on a genome-wide scale, minimizing potential errors associated with a reference-dominated approach. This approach led to the identification of 2,614 ASEGs, with 1,254 showing maternal allele dominance and 1,360 showing paternal allele dominance ($\log_2|FC| > 1$; $P < 0.01$) (Figure 6B). The number of ASEGs with maternal allele dominance and paternal allele dominance was approximately equal, suggesting a balanced influence of both parental alleles and a well-maintained equilibrium in *B. blakeana*. Notably, we observed an interlaced genomic distribution of these ASEGs, with maternal and paternal dominance genes interspersed throughout the genome (Figure 6C). Furthermore, GO and KEGG enrichment analyses of these ASEGs provided valuable insights into their functional implications. We observed significant enrichment in several biological processes associated with maintaining genomic stability, responding to DNA damage, and ensuring proper cellular function under stress conditions. Enriched GO categories included "DNA repair", "response to DNA damage stimulus", "cellular response to stress", and "double-strand break repair" (Figure 6D; Supplementary Table S18 A-B). KEGG pathway analysis further revealed enrichment in pathways such as 'Non-homologous end-joining', 'Circadian rhythm - plant', and 'Glycosylphosphatidylinositol (GPI)-anchor biosynthesis' (Supplementary Table S18 C-D).

Pigment biosynthesis in *B. blakeana*

B. blakeana exhibits flower heterosis, characterized by significant improvements in various traits compared to its parental species [1]. To investigate the underlying mechanisms associated with flower color formation in *Bauhinia* species, we constructed pigment metabolic pathways, specifically focusing on anthocyanins, carotenoids, and chlorophylls (Figure 7). Initially, we identified orthologous gene groups associated with these pigment metabolic pathways in *B. purpurea*, *B. variegata*, and the two haplotypes of *B. blakeana* (Supplementary Table S19-21). This information also allowed us to investigate the impact of reference genome choice on gene expression analysis. Using ortholog information, we performed pairwise comparisons of expression values (FPKM: fragments per kilobase of transcript per million mapped reads) for orthologous genes within the same sample. Specifically, we compared the FPKM values obtained using two different reference genomes: *B. purpurea* and *B. variegata*. Our findings were consistent with our previous results, as we observed no significant differences in gene expression among all groups when directly comparing the data between groups using Welch's t-test (Supplementary Table S22; Supplementary Figure S4). However, when we performed the paired t-test, which considers the paired nature of the data within each group, we found no significant differences in gene expression in six out of the total eight samples, regardless of the reference genome chosen (Supplementary Table S23A; Supplementary Figure S5A). To examine whether the expression value of the maternal and paternal alleles add up to the expression value in *B. blakeana* when using *B. purpurea* or *B. variegata* as references, we obtained allelic

expression counts of *B. blakeana* using the *B. blakeana* haplotype metagenome as a reference (Supplementary Table S22). Our analysis revealed no significant difference among three key expression values of the genes involved in pigment biosynthesis in *B. blakeana*: the sum of allelic expression level when using the haplotype metagenome as a reference; the expression level observed in *B. blakeana* when using *B. purpurea* as a reference; and the expression level observed in *B. blakeana* when using *B. variegata* as a reference (Supplementary Table S23B; Supplementary Figure S5B). This finding indicates that the combined expression value of the maternal and paternal alleles accurately represents the overall expression level in *B. blakeana*, regardless of whether *B. purpurea* or *B. variegata* is used as the reference genome. This consistency in expression levels strengthens the reliability of our analysis and demonstrates that our gene expression assessment effectively captures the contributions of both parental alleles.

We then investigated the copy number differences of metabolism genes within these pathways across four *Bauhinia* assemblies. In general, most genes displayed conserved copy numbers across all four assemblies. However, we observed an interesting exception concerning the CHI (chalcone isomerase; EC:5.5.1.6) gene, which plays a crucial role in the anthocyanin biosynthesis pathway. Specifically, the *B. purpurea* and *B. blakeana* maternal haplotype exhibited three copies of the CHI gene, while the *B. variegata* and *B. blakeana* paternal haplotype contained four copies of CHI (Figure 7A; Supplementary Table S19). The presence of one less CHI gene in *B. purpurea* was

further supported by the absence of RNA-Seq expression counts for its orthologous gene in *B. purpurea* samples when using *B. variegata* as a reference (Supplementary Table S22).

Subsequently, we conducted a comparative transcriptome analysis on these pigment biosynthesis pathways in the three *Bauhinia* species. To facilitate this analysis, we created a new expression matrix by calculating the average FPKM from the FPKM expression data obtained in previous DEG analyses, using *B. purpurea* and *B. variegata* as references (Supplementary Table S24). With this matrix, we examined the expression level dynamics of genes involved in pigment biosynthesis pathways in the parental species and the hybrid *B. blakeana* (Figure 7A-C). Interestingly, we observed that the paternal species, *B. variegata*, exhibited an overall higher expression level of these genes. Next, we summed up the average FPKM values of individual gene copies belonging to the same gene, resulting in a new expression matrix. This matrix allows us to consolidate expression information and provide a representation measure of gene expression for further analysis (Supplementary Table S25). This approach allowed us to capture the overall expression level of each functional gene within the context of pigment biosynthesis pathways. We identified several genes (22.41%, 13 out of 58) that exhibited clear high-parent dominance, where the expression level differed between the two parents but resembled the higher expressing parent in *B. blakeana*, namely CHI, DXS, DXR, CMK, HDS, HDR, GGPPS, ZDS, ZEP, CAO, SGR, PAO and RCCR (Figure 7D; Supplementary Table S25). Additionally, we observed five genes, including

PAL, PDS, CYP97A3, ABA4, and HCAR, showing over-dominance expression patterns in *B. blakeana*. In these cases, *B. blakeana* exhibited higher expression levels compared to both parental species. Notably, these dominance complementation and over-dominance expression patterns were particularly evident in genes involved in carotenoid biosynthesis pathways (47.83%, 11 out of 23). These expression patterns of dominance complementation and over-dominance likely play a role in the elevated expression levels of carotenoid biosynthesis-related genes, thus contributing to the flower color heterosis in *B. blakeana*.

The roles of gene copy expression preference and ASEGs in flower color heterosis

Expanding upon our previous examination of gene copy expression differences among the three *Bauhinia* species and overall ASE patterns in *B. blakeana*, our subsequent investigation aimed to delve deeper into the role of gene copy expression preference and ASEGs in flower color heterosis. Firstly, we examined the expression levels of individual gene copies to identify any distinct preferences in gene copy utilization within each specific gene. Our findings revealed variations in the expression levels of specific gene copies among the three *Bauhinia* species, indicating the presence of species-specific expression patterns. For instance, the DXR gene (1-Deoxy-D-xylulose-5-phosphate reductoisomerase; EC:1.1.1.267), a key enzyme in the MEP pathway [43, 44], exhibited over-dominance expression in *B. blakeana*. Between a total of two copies of the DXR gene, DXR_2 was consistently favored and exhibited higher expression levels in all three *Bauhinia* species (Supplementary Table S24). On the other hand, the

PAL gene (Phenylalanine ammonia-lyase; EC:4.3.1.24), a crucial enzyme involved in plant metabolism responsible for the first step in the biosynthesis of various natural products containing the phenylpropane skeleton [45], also exhibited over-dominance expression in *B. blakeana*. However, this over-dominance pattern was not consistently observed across all four PAL gene copies. Among the examined gene copies, PAL_3 consistently demonstrated the highest expression levels in both *B. blakeana* and *B. variegata*, while *B. purpurea* specifically exhibited the highest expression level in PAL_4. The observed over-dominance in the PAL gene of *B. blakeana* was attributed to the elevated expression level of PAL_3 (Supplementary Table S24). Furthermore, variations in expression preferences were also observed in other genes within the pigment biosynthesis pathways, highlighting the presence of species-specific expression patterns that are likely to contribute to the character specialization observed within each *Bauhinia* species.

Motivated by the observed variations in gene copy utilization preferences and expression level differences among the *Bauhinia* species, we further investigated the ASE patterns of each of these genes to determine whether the alleles in *B. blakeana* inherited the expression patterns corresponding to those of the parental species. To investigate ASE within pigment biosynthesis pathways, we applied a replicate-specific thresholding approach (ASE ratios >0.7 or <0.3 in ≥ 2 replicates) to identify ASEGs with consistent allelic bias. Specifically, we calculated the ASE ratio by dividing maternal allele expression by the sum of maternal and paternal allele expressions.

Genes were classified as ASEGs if they exhibited significant ASE ratios (>0.7 maternal allele dominance; or <0.3 paternal allele dominance) in at least two out of three *B. blakeana* replicates. This method, while less stringent than the genome-wide DESeq2 analysis, allowed us to capture a broader profile of ASEGs with reproducible allelic imbalances. It allowed targeted exploration of allele-specific regulation in pathways critical to flower color heterosis in *B. blakeana*. Within the anthocyanin biosynthesis pathway, we identified 10 genes with maternal allele dominance and 7 genes with paternal allele dominance out of the total 27 genes analyzed (Figure 7A-C; Supplementary Table S26). In the carotenoid biosynthesis pathway, we found 9 genes with maternal allele dominance and 12 genes with paternal allele dominance out of the total 43 genes analyzed. Similarly, within the chlorophyll biosynthesis pathway, we detected 5 genes with maternal allele dominance and 13 genes with paternal allele dominance out of the total 58 genes analyzed. The proportions of ASEGs were 62.96% in the anthocyanin pathway, 48.84% in the carotenoid pathway, and 31.03% in the chlorophyll pathway. Although the majority (85.71%, 48 out of 56) of the identified ASEGs in *B. blakeana* exhibited parental allelic dominance biased towards the parent with a higher expression level, there are instances where the allelic dominance did not strictly correspond to the expression patterns of the parental species. This observation suggests that additional factors beyond the expression levels or *cis*-regulation of the parental species also play a role in regulating gene expression and establishing allelic dominance in *B. blakeana*. These factors, such as trans-regulatory elements, epigenetic

modifications, or genetic interactions, may also contribute to shaping the observed expression patterns in *B. blakeana*.

Discussion

In this study, we successfully generated chromosome-level genome assemblies for two parental species, *B. purpurea,* and *B. variegata*, as well as haplotype-resolved gapless genome assemblies for the hybrid *B. blakeana*. The utilization of the trio-binning assembly strategy, taking advantage of the high heterozygosity in the *B. blakeana* genome, enabled us to overcome the challenges posed by heterozygosity and obtain high-quality genome assemblies for further analyses. The haplotype-resolved genome assemblies served as a solid foundation for our extensive downstream investigations, offering prospects for delving into the complex characteristics of the heterogeneous *B. blakeana* genome and uncovering deeper insights into its biology. Furthermore, by obtaining the cp genomes of all three *Bauhinia* species, we were able to confirm *B. purpurea* as the maternal parent of *B. blakeana* through comparative cp genome analyses, as well as confirming a close phylogenetic relationship between *B. blakeana* and *B. variegata*. Nuclear phylogenomics further corroborated the hybrid origin as the strong monophyly of *B. blakeana* haplotypes with their respective parents (Hmat with *B. purpurea*; Hpat with *B. variegata*).

Utilizing the high-quality genome assemblies, our subsequent comparative genomic analysis uncovered several gene families associated with terpenoid and flavonoid

biosynthesis that have undergone expansions during the evolutionary diversification of the *Bauhinia* genus. Additionally, notable expansions were also observed within the TPS gene family of *Bauhinia* species when compared to other members in the Fabaceae family. Terpenes, commonly released by plants in response to insect herbivory, are primarily derived from the five-carbon precursor, isopentenyl diphosphate (IPP). These compounds are synthesized through two distinct pathways within the plant cell: the mevalonate (MVA) pathway in the cytosol and the 2C-methyl erythritol 4-phosphate (MEP) pathway in plastids. The specific terpene synthase enzymes play a crucial role in determining the structure of the terpenes produced [46]. The expansion of TPS genes in *Bauhinia* species has the potential to confer heightened resistance to pathogens, establishing a more robust defense mechanism against a diverse range of microbial invaders. Terpenoids and flavonoids are major classes of secondary metabolites that exhibit a variety of pharmacological bioactivity, including anti-microbial, anti-inflammatory, anti-diabetic, and anti-cancer effects. The genus *Bauhinia* has a long history of usage in herbal medicine for treating conditions such as malaria, diarrhea, diabetes, and various other health conditions. Specifically, *B. purpurea* and *B. variegata* have been extensively used in traditional medicine and have been extensively investigated for their medicinal properties [47-50]. The expansion of TPS family genes, particularly TPS-b genes, likely contributes to the abundant terpenoid content, thereby underpinning the observed medicinal properties of these species. We also observed enrichment of the KEGG term 'Cutin, suberine, and wax biosynthesis' within the expanded gene families of the *Bauhinia* genus, potentially explaining the unique leaf

characteristics of *B. blakeana*, characterized by cells and epicuticular wax arranged in a regular pattern, leading to its limited dust-catching capacity [51].

B. blakeana exhibits flower heterosis characterized by its large, showy, and vibrant magenta-colored flowers resembling orchids. Despite its sterile nature, *B. blakeana* has gained popularity as an ornamental species worldwide, mainly due to its unique floral display and extended flowering period. Therefore, our study aimed to investigate the transcriptome profiles of flower tissues and the genetic mechanisms contributing to the observed phenotypic variation among the three *Bauhinia* species, with a specific focus on studying the flower color heterosis in *B. blakeana*. Specifically, *B. purpurea* displayed much paler coloration compared to *B. variegata*. Through further comparing the transcriptome profiles between *B. blakeana* and its parental species, we found that even though *B. blakeana* exhibits flower color that is more similar to its paternal parent [1], the general gene expression profile of *B. blakeana* aligns more closely with its maternal parent as evidenced by the lower number of DEGs between *B. blakeana* and *B. purpurea* (5,116 genes) compared to *B. blakeana* and *B. variegata* (8,981 genes) (Figure 5A). We observed that the number of genes exhibiting up-regulated expression in *B. blakeana* (2,607 and 2,717 genes using *B. purpurea* and *B. variegata* as references, respectively) is comparatively lower than the number of genes showing down-regulated expression (4,504 and 4,570 genes) when compared to the MPV (Figure 5C). This observation suggests a potential trade-off, wherein *B. blakeana* may have sacrificed certain functional attributes in favor of achieving heterosis-related traits such as flower

color and a prolonged flowering period [52, 53].

There are two main classical hypotheses that aim to explain the mechanisms underlying heterosis: dominance and over-dominance hypothesis [54, 55]. The dominance hypothesis focuses on the significance of dominant alleles, while the over-dominance hypothesis emphasizes the advantages of heterozygosity. These two hypotheses are not mutually exclusive, as both mechanisms may contribute to heterosis. To investigate the genetic mechanisms underlying flower color heterosis in *B. blakeana*, we conducted analyses of gene expression patterns involved in pigment biosynthesis pathways. We found that 31.03% (18 out of 58) of these genes exhibited dominance complementation or over-dominance expression patterns (Figure 7D). Notably, within the subset of genes related to carotenoid biosynthesis pathways, approximately half (47.83%, 11 out of 23) displayed such non-additive expression modes. Genes associated with carotenoid biosynthesis showed significant expression differences between the parental species, with enrichment of the carotenoid biosynthesis pathway (KEGG pathway ko00906) among DEGs with large expression disparities (log2|FC| > 4; $P < 0.01$) between *B. purpurea* and *B. variegata*. The pronounced variations in expression levels observed within the carotenoid biosynthesis pathways between the parental species may reflect underlying divergence in cis/trans-regulatory elements or epigenetic differences. Recent studies have shown that when parental species exhibit significant expression divergence for a particular gene, hybrids often exhibit an expression-level dominance-UP pattern, where promoter activity and transcriptional output in the hybrid are biased

toward the parent with higher expression levels [56, 57]. This regulatory asymmetry likely arises from differential binding affinities of TFs and epigenetic modifications inherited from the parents, ultimately driving higher-than-average gene expression in the hybrid. Such mechanisms may explain the observed enrichment of non-additive expression patterns in carotenoid biosynthesis genes in *B. blakeana*. For example, DXS (1-deoxy-D-xylulose 5-phosphate synthase; EC:2.2.1.7) and DXR (1-deoxy-D-xylulose 5-phosphate reductoisomerase; EC:1.1.1.267), which catalyze the first two committed steps of the MEP pathway, exhibited high-parent dominance in *B. blakeana*. Subsequent enzymes (CMK, HDS, HDR; EC:2.7.1.148, EC:1.17.7.1, EC:1.17.1.2) also showed dominance complementation, suggesting coordinated upregulation of the MEP pathway, potentially enhancing the flux of isoprenoid precursors into carotenoid biosynthesis, thus contributing to the flower color heterosis in *B. blakeana* [58, 59].

ASE is another mechanism that has been suggested to contribute to heterosis [60-62]. We employed two distinct approaches to conduct genome-wide analyses of ASE in flower tissues of *B. blakeana*. Although there were variations in the total number of identified ASEGs between the two methods, we observed a balance in both the number and level of ASEGs biased towards each parental allele within each method. Despite the limitations of both approaches used, they yielded valuable insights into the ASE landscape within the *B. blakeana* genome, highlighting the equitable participation of maternal and paternal alleles in shaping the observed ASE patterns. Through our in-depth analysis of pigment biosynthesis-related genes in *B. blakeana*, we discovered that

the ASE patterns demonstrate a preference for the parental allele linked to higher expression levels in the comparison between the parental species (Figure 7A-C). However, it is important to note that the ASE patterns do not consistently correlate with dominance complementation or over-dominance expression patterns. In our analysis, genes exhibiting dominance complementation or over-dominance expression patterns may display diverse ASE behaviors across their different copies. For example, within the same gene, there can be variations among gene copies: all copies of a gene may exhibit ASE bias towards the same parental allele (eg. DXR and GGPPS), and some copies of a gene may show ASE bias towards different parental alleles (eg. PAL and DXS), and in some cases, all copies of a gene may not display ASE at all (eg. ZDS and ABA4). The variability observed indicates that dominance patterns and ASE are influenced by complex layers of regulation, highlighting the intricate interplay between allelic-specific expression and genetic regulatory mechanisms. Further investigation into the specific regulatory factors influencing ASE and dominance patterns, such as TFs, *cis*-regulatory elements, and epigenetic modifications will provide a deeper understanding of how these mechanisms interact to shape gene expression and phenotypic outcomes. Resolving these complexities will elucidate the molecular mechanisms underlying heterosis and uncover how hybrid organisms acquire superior traits via synergistic interactions between parental genetic and epigenetic regulatory networks.

Overall, our study provides comprehensive genomic and transcriptomic insights into

the biology of *B. blakeana*. Through the utilization of our *de novo* assembled haplotype-resolved and gapless T2T genome, we have advanced our understanding of the genomic structure and genetic mechanisms underlying the captivating flower color trait in this popular ornamental hybrid tree species, serving as a case study for investigating traits in hybrid species. Furthermore, the resources generated in this study lay the foundation for future genetic studies, breeding programs, and conservation initiatives in *Bauhinia* species.

Methods

Plant sampling, library preparation, and sequencing

Fresh leaves of three *Bauhinia* species, namely *Bauhinia x blakeana Dunn* (NCBI: txid180222), *Bauhinia purpurea L.* (NCBI: txid3806), and *Bauhinia variegata L.* (NCBI: txid167791), were collected from Shenzhen, Guangdong Province, China. To perform whole-genome sequencing on all three species, high-molecular-weight (HMW) genomic DNA was extracted using a modified CTAB (cetyltrimethylammonium bromide) method [63]. The extracted DNA from each species was used to prepare Single-tube Long Fragment Read (stLFR) libraries [64], and WGS short-read libraries, following the respective protocols. Hi-C libraries were constructed for each species using the MboI enzyme and following the standard Hi-C library preparation protocol [65]. These libraries were subsequently sequenced on the BGISEQ500 platform to generate pair-end reads with an insert size of ~250bp [66]. In addition, we prepared an extra ONT library for the hybrid species *B. blakeana* using the LSK108 kit (SQK-

LSK108, Oxford), which was then sequenced on the Nanopore MinION sequencer [67].

To perform transcriptome sequencing, we collected three fully blossomed flower tissues from each individual of the three sequenced *Bauhinia* species. Total RNA was isolated using the TIANGEN Kit with DNase I and processed using the NEBNextUltra^{TM} RNA Library Prep Kit to create a pair-end library with a 250 bp insert size for each sample. The libraries were then barcoded and pooled together as input for downstream sequencing on the BGI-DIPSEQ platform.

Genome size estimation

Previous studies have shown that the three *Bauhinia* species share the same chromosome number (2n=28) [4]. To estimate the genome size of each species, we performed *k*-mer analysis. First, the raw WGS short-reads were filtered according to the sequencing quality with Trimmomatic (RRID: SCR_011848) (v0.40) with "ILLUMINACLIP:adapter.fa:2:30:10 HEADCROP:5 LEADING:3 TRAILING:3 SLIDINGWINDOW:5:15 MINLEN:95" parameter [68]. Next, *k*-mer frequencies were counted by Jellyfish (RRID: SCR_005491) (v2.2.6) with a *k*-value of 21 using the clean WGS reads [69]. Based on the 21-mer frequency distribution analysis with GenomeScope (RRID: SCR_017014) [70], we estimated the genome size of *B. purpurea*, *B. variegata,* and *B. blakeana* to be ~303.68 Mb, ~314.49 Mb, and ~290.97 Mb, respectively. Notably, the estimated genome size of *B. variegata* was close to the previously published genome size of 326.4Mb [5].

Genome assembly and quality control

To generate draft assemblies for the parental species *B. purpurea* and *B. variegata*, we performed *de novo* assembly using the Supernova assembler (RRID: SCR_016756) (v2.1.1) with the "--max reads 2140000000" parameter for each species using the stLFR reads [71]. Next, we used the clean WGS short-read data of each species to fill gaps in the draft assemblies using the GapCloser (RRID: SCR_015026) with default parameters. To further improve the assembly contiguity, we utilized Hi-C data from each parental species. We aligned the Hi-C data to the draft assemblies using BWA (RRID: SCR_010910) -MEM [72] and then integrated the assemblies from contig-level into pseudochromosome-level using ALLHiC (RRID: SCR_022750) [13]. Specifically, we used the bam files resulting from the alignment to assign contigs into a pre-defined number of groups (14 groups in our research), and unplaced contigs were assigned into partitioned clusters. Finally, we reordered and oriented each group to optimize the result and generate the fasta format sequences and agp location files. We evaluated the genome scaffolding by plotting the chromatin contact matrix.

To assemble the hybrid offspring *B. blakeana*, we employed a trio-binning strategy to generate two fully phased haplotype assemblies. Firstly, we identified solid *k*-mers, which are *k*-mers that are unique in the three *Bauhinia* genomes. This yielded three sets of solid *k*-mers, for the hybrid, paternal, and maternal sequencing data. Subsequently, we defined paternal and maternal hap-mers. Paternal hap-mers are the

intersection between the paternal and hybrid solid *k*-mers, while maternal hap-mers are the intersection between the maternal and hybrid solid *k*-mers. This definition is similar to the concept of hap-mers used in Merqury but adjusted to accommodate our solid *k*-mers. We proceeded to assemble the two haplotypes of *B. blakeana* separately. First, we categorized all reads into three groups: paternal reads, maternal reads, and ambiguous reads. Reads that exclusively contained paternal hap-mers as their solid *k*-mers were classified as paternal reads, and the same principle applied to maternal reads. Reads containing both types of hap-mers or neither were labeled as ambiguous. We then ran the hypo-assembler in haploid mode for each haplotype, once with paternal and ambiguous reads, and once with maternal and ambiguous reads. This approach resulted in two distinct yet more accurate assemblies. Following this, haplotype-specific Hi-C reads were aligned to their respective draft assemblies for scaffolding based on contact frequency. Subsequently, we manually clustered the remaining long reads from the previous steps and assembled them. This newly assembled set of contigs was used for gap-filling purposes. After completing the aforementioned steps, the majority of the genome was resolved. However, not all the telomeres are fully assembled. To address this, we identified reads displaying a high abundance of telomere signals that were not utilized in the initial assembly. Subsequently, we manually clustered these reads based on their SNPs in comparison with the existing contig terminals and then assigned the clusters to their respective terminal positions.

The genome completeness was evaluated by BUSCO (RRID: SCR_015008) using the

embryophyta_odb10 database [27]. The genome continuity was evaluated by calculating contig N50 length. The accuracy of the genome was evaluated by mapping the WGS sequencing data to the genome with BWA-MEM and calculating mapping rate and coverages with SAMTOOLS (RRID: SCR_002105) [73]. To further assess the two haplotype genomes of *B. blakeana*, we used Merqury (RRID: SCR_022964) [29] to evaluate the haplotype-specific accuracy, completeness, and phase block continuity based on the trio information.

Identification of repetitive elements

To identify repetitive elements in our assembled genomes, we employed a combination of homology-based and *de novo* prediction methods following the Repeat Library Construction-Advanced pipeline [74]. Firstly, we employed RepeatMasker (RRID: SCR_012954) [75] and RepeatProteinMasker to identify transposable elements (TEs) based on similarity-based comparisons to search for known repeat sequences with Repbase (RRID: SCR_021169) [76]. In addition, we used LTR_Finder (RRID:SCR_015247) [77] to search for LTR retrotransposons *de novo*. The resulting repetitive sequence libraries were then integrated using RepeatModeler (RRID: SCR_015027) [78] to create a complete and non-redundant custom library, which served as input for RepeatMasker to identify and classify TEs in the genome assemblies. Furthermore, we searched for tandem repeats across the genomes using Tandem Repeats Finder (RRID: SCR_022193) [79]. All identified repeats were used to soft mask the genome assemblies with RepeatMasker before gene structure prediction.

Protein-coding gene prediction and functional annotation

We utilized a combination of *ab initio*, homology-based, and RNA-seq-based approaches with the BRAKER2 (RRID: SCR_018964) pipeline [80] to predict the protein-coding gene set in our assembled genomes. To begin, we obtained and assembled the publicly available leaf transcriptome data for each species from the crowdfunded *Bauhinia* Genome project [15]. The leaf data were then aligned to the corresponding genomes using HISAT2 (RRID: SCR_015530) (v2.1.0) [81] with "--max-intronlen 500000 --sensitive --dta --dta-cufflinks --phred33 --no-discordant --no-mixed" parameters, and the resulting BAM files were sorted using SAMTOOLS. We used the BAM files, along with the OrthoDB (RRID: SCR_011980) v10.1 protein database [82] (the published *B. variegata* proteins were added), as input for BRAKER2 with "--softmasking --etpmode". We further filtered the predicted gene sets to remove any translated proteins less than 30 amino acids in length or with in-frame stop codons. Finally, we evaluated the completeness of the gene sets using BUSCO with the embryophyta_odb10 database.

We used two methods to infer the functions of our predicted genes. First, we performed a BLASTP (RRID: SCR_001010) homolog search against public protein databases such as UniProtKB/Swiss-Prot (RRID: SCR_021164), TrEMBL, NCBI non-redundant (NR), and KEGG (RRID: SCR_012773). Second, we utilized InterProScan (RRID: SCR_005829) to search for conserved amino acid sequences, motifs, and domains by

comparing the sequences against domain databases including Pfam (RRID: SCR_004726), PANTHER (RRID: SCR_004869), PRINTS (RRID: SCR_003412), PROSITE (RRID: SCR_003457), ProDom (RRID: SCR_006969), and SMART (RRID: SCR_005026).

Identification of structural variations, centromeres, and telomeres

The Nucmer alignment tool from the MUMmer (RRID: SCR_018171) [83] was used for conducting whole-genome alignments. Nucmer was executed with the -maxmatch option to retrieve all alignments between the *B. blakeana* allelic chromosomes, with parameters -c 500, -b 500, and -l 100. Subsequently, the delta-filter and show-coords subprograms were employed to filter the alignments and convert them into tab-delimited files. Lastly, SyRI (RRID: SCR_023008) [35] was used to detect inversions, translocations, and duplications.

CentroMiner from the quarTeT prediction software (RRID: SCR_025258) [34] was employed for centromere identification. To enhance its performance, the repeat and gene annotations obtained from previous analyses were added as input. The resulting predictions underwent a manual selection process to ensure accuracy and reliability before finalization. TeloExplorer from quarTeT was used for telomere identification by searching for the characteristic motif (TTTAGGG).

Identification of non-coding RNAs

In addition to protein-coding genes, we also identified ncRNAs within our assembled genomes. We used tRNAscan-SE (RRID: SCR_008637) [84] to identify tRNA genes, and BLASTN (RRID: SCR_001598) to search for rRNA genes by comparing the rRNA sequences of *Arabidopsis thaliana* and *Oryza sativa* against each of the three *Bauhinia* assemblies. We predicted miRNAs and snRNAs by searching the sequences against the Rfam (RRID: SCR_007891) database using Infernal (RRID: SCR_011809) [85].

Identification of transcription factors

We identified and classified transcription factors (TFs), transcription regulators (TRs), and protein kinases (PTKs) among our predicted gene models into different families using the online tool iTAK pipeline with default parameters [86].

Phylogenetic analysis and divergence time estimation

Single copy genes from 15 selected plants were identified using OrthoFinder (RRID: SCR_017118) [87] and subsequently used to construct the phylogenetic tree, following these steps: 1. For each single-copy gene orthogroup data set, we performed multiple amino acid sequence alignments using MAFFT (RRID: SCR_011811) (v.7.310) [88], followed by gap position removal using Gblocks (RRID: SCR_015945) (v.0.91b) (positions where 50% or more of the sequences have a gap were removed) [89]. 2. We used the maximum-likelihood (ML) software IQ-TREE (RRID: SCR_017254) (v.1.6.1) [90] to reconstruct the phylogenetic tree for each single-copy gene family. 3. The gene trees of each data set were then analyzed using ASTRAL (RRID: SCR_024520) (v.5.5.9)

[91] to infer the species tree with quartet scores and posterior probabilities. 4. The sequences generated from step one were also concatenated as a single supermatrix and a concatenation tree was generated using RAxML (RRID: SCR_006086) [92].

We used the MCMCTree program in the PAML package (RRID:SCR_014932) (v4.5) [93] to estimate the divergence time of each tree node, based on the estimated divergence times of the following nodes from the TimeTree (RRID:SCR_021162): *C. canephora – V. vinifera* (111.4-123.9 MYA), *A. thaliana* - *V. vinifera* (111.24-117.56 MYA), *A. thaliana* - *M. truncatula* (102-112.5 MYA) and *A. thaliana* - *P. trichocarpa* (107-109 MYA). To perform this analysis, we used the sequential PHYLIP format nucleotide sequences and rooted phylogenetic tree derived from the result of the gene family analysis as inputs for MCMCTree.

We used CAFE (v2.1) [94] to infer the expansion and contraction of gene families based on the phylogenetic analysis and divergence time. The input tree for CAFE was the species tree constructed by ASTRAL. For each gene family that was significantly expanded or contracted (P-value < 0.05), we inferred functional information based on the functional annotation results. KEGG and GO enrichment analyses of genes were conducted using an enrichment pipeline (parameter setting: *p* Adjust Method: fdr; TestMethod: FisherChiSquare) [95, 96].

<u>Assembly of chloroplast genome and phylogenetic analysis of hybrid origin</u>

The chloroplast genomes (cp) of the three *Bauhinia* species were assembled using the clean WGS short-read data in GetOrganelle (RRID: SCR_022963) [97], and further annotated using CpGAVAS2 [98]. We obtained additional available *Bauhinia* cp genomes from the NCBI database, including *B. binate* (NC_037764.1), *B. brachycarpa* (NC_037762.1), *B. racemosa* (ON456405.1). *C. cancadensis* (KF856619.1) from the Cercis genus was also obtained to serve as an outgroup. To construct the phylogenetic tree, a total of 77 protein-coding genes were aligned and trimmed following the same pipeline used for the nuclear tree, and the phylogenetic tree was built by RAxML with "-f a -#1000 -m PROTGAMMAJTT" parameters. In addition, we obtained previously published chloroplast genomes of *B. blakeana* (MN413506.1), *B. purpurea* (NC_061218.1), and *B. variegata* (MT176420) from the NCBI database for comparison with our assembled genomes using mVISTA [39, 99].

Hybrid origin analysis based on whole genome data followed the phylogenetic workflow described previously, with modifications to taxon sampling and data processing. Single-copy orthologs were identified from *B. purpurea*, *B. variegata*, *B. blakeana* haplotypes (Hmat/Hpat), *C. canadensis*, *C. chinensis*, and *A. thaliana* using OrthoFinder (RRID: SCR_017118) [87] with default parameters. From the resulting 2,360 single-copy genes, gene trees were constructed using IQ-TREE (RRID: SCR_017254) [90]. Subsequently, species tree inference was performed via ASTRAL (RRID: SCR_024520) [91] with the "-t 2" parameter. Phytop [41] was employed to quantify ILS and IH signals based on the ASTRAL species tree.

RNA-seq data analysis and ASE gene identification

RNA-seq sequencing data were trimmed using Trimmomatic to remove low-quality bases and adapter sequences. Clean reads of all three species were mapped to the selected reference genome using Bowtie 2 (RRID: SCR_016368) and the counts and FPKM value were calculated by the eXpress program [100], which was incorporated in the Trinity (RRID: SCR_013048) package. DEGs were identified based on the counts using DESeq2 (RRID: SCR_015687) [101].

We employed two distinct methods for genome-wide identification of ASEGs in *B. blakeana*:

1. Diagnostic SNP-Based Method: Clean RNA-seq reads were processed by HyLiTE to produce tables of parental and allelic expression data in a single step. First, RNA-seq reads from *B. blakeana* and its parents were aligned to the *B. purpurea* reference genome using Bowtie2 with default parameters. Next, alignments were proceeded to SAMtools to generate the .pileup file. HyLiTE was used to detect diagnostic SNPs (positions with fixed differences between *B. purpurea* and *B. variegata*) and assign *B. blakeana* reads to parental alleles. The HyLiTE output provided maternal and paternal allele counts per gene for each *B. blakeana* sample. These counts were manually transformed into a DESeq2-compatible matrix, where each gene's expression was represented as a two-column matrix (maternal counts vs paternal counts). DESeq2 was then applied to test for significant allelic imbalance.

2. Haplotype-Resolved Method: Syntenic gene blocks between *B. blakeana* maternal haplotype and paternal haplotype were identified using BLASTP and MCScanX (RRID: SCR_022067) [102] with annotations and protein sequences. Genes from the same orthogroup of the two haplotypes were identified using OrthoFinder. Gene pairs belonging to the same orthogroup and located in large syntenic blocks were identified as alleles. The assemblies and annotations of both haplotypes were then combined to construct a metagenome. Clean RNA-seq reads of *B. blakeana* were mapped to the metagenome using Bowtie2 by retaining the best alignment. FPKM and counts were calculated using the eXpress program. To screen for ASEGs, we employed DESeq2 using the allelic read count data.

For ASEG identification in pigment biosynthesis pathways, we applied a replicate-specific thresholding approach. This approach relaxed statistical stringency compared to the DESeq2-based methods described above to capture ASEGs with reproducible allelic biases, even if they did not meet genome-wide significance thresholds. For each gene in B. *blakeana*, we calculated the ASE ratio as:

$$\text{ASE Ratio} = \frac{\text{Maternal Allele Expression (FPKM)}}{\text{Maternal + Paternal Allele Expression (FPKM)}}$$

The allele expression values were derived from RNA-seq read counts mapped to the haplotype-resolved metagenome, same as the haplotype-resolved method described above. ASEG was defined as a gene with an ASE ratio >0.7 (maternal dominance) or <0.3 (paternal dominance) in ≥2 of 3 replicates.

Identification of flower pigmentation genes

To elucidate the mechanisms underlying flower pigmentation, we focused on the metabolism and accumulation of flavonols, anthocyanins, carotenoids, and chlorophylls. Initially, we constructed the metabolic pathways associated with these compounds. For reference, we downloaded gene sequences encoding enzymes involved in these pathways from UniProt (RRID: SCR_002380). These reference sequences served as a basis for identifying corresponding genes in our assemblies. Our candidate gene selection process involved the following criteria: 1. Candidate gene sequences were identified through BLASTP searches using a cut-off E-value of 1e−05, comparing them to the query gene sequences we obtained. 2. Functional annotations of the candidate genes were manually inspected to ensure similarity to the query genes. 3. Following the initial identification, the candidate genes underwent further verification by constructing phylogenetic trees. The maximum likelihood trees were constructed using IQTREE after aligning the sequences with MAFFT.

Data Availability

The raw genomic sequencing data for all three analyzed species have been deposited in the NCBI Sequence Read Archive (SRA) under BioProject accession number PRJNA1219298. The genome assemblies, annotations, and flower tissue transcriptomic data are available at the China National GeneBank (CNGB) Sequence Archive (CNSA) under accession numbers CNP0001583 and CNP0006215. Additionally, previously published leaf transcriptome data can be accessed via NCBI BioProject PRJEB21302

and GigaDB [15]. All additional supporting data are available in the *GigaScience* repository, GigaDB [103], with individual datasets for parental species *B. purpurea* [104], *B. variegata* [105] and the hybrid species *B. blakeana* [106].

Editors' note

GigaScience Press was one of the founders of Bauhinia Genome in 2015, a community genomics project engaging the community in the sequencing of Hong Kong's floral emblem to promote genomics literacy and education. As an open science project also teaching reproducible science practices, we committed to make all the data, protocols and supporting materials open and transparent and we hope the publication of this fully complete genome and its supporting data fulfils that pledge. We would like to thank the Bauhinia Genome community for crowdfunding the original *Bauhinia* transcriptomic data used here, and their ongoing support and interest. For more see the website bauhiniagenome.hk

Abbreviations

ASE: allele-specific expression; ASEG: allele-specific expressed genes; CHI: chalcone isomerase; FPKM: fragments per kilobase of transcript per million mapped reads; Hi-C: high-throughput/resolution chromosome conformation capture; MPV: mid-parent value; ONT: Oxford Nanopore Technologies; stLFR: Single-tube Long Fragment Read; SV: structural variation; T2T: telomere-to-telomere; TPS: Terpene synthases; WGS: Whole Genome Sequencing.

Consent for publication

All the authors approved the manuscript and gave their consent for submission and publication.

Competing Interests

The authors declare that they have no competing interests.

Authors' Contributions

S.K.W.T. and T.W. conceived and supervised the research. Z.C., K.H.S.T, R.L.D and S.C.E collected the samples and performed the experiments. W.M., J.C.D, W.K.S., X.G., T.Y., M.W.M.T and W.S.C analyzed the data. W.M. and J.C.D. wrote the manuscript. W.K.S, X.G, S.C.E, T.W and S.K.W.T revised the manuscript. All authors have read and approved the final manuscript.

Acknowledgments

This project was supported by the General Research Funds (Ref. No.: 14119420 and 14121222), Collaborative Research Fund (Ref. No.: C4049-23EF), and Theme-based Research Scheme (Ref. No.: T11-709/21-N, T12-716/22-R, and T11-702/24-N) from the Hong Kong Research Grants Council. Additional support was provided by the China National GeneBank (CNGB), the Key Laboratory of Genomics, Ministry of Agriculture, and the Guangdong Provincial Key Laboratory of Core Collection of Crop

Genetic Resources Research and Application.

References

1. Lau CP, Ramsden L, Saunders RM: **Hybrid origin of "*Bauhinia blakeana*" (Leguminosae: Caesalpinioideae), inferred using morphological, reproductive, and molecular data.** *Am J Bot* 2005, **92:**525-533.
2. Dunn S: **New Chinese plants.** *Journal of Botany* 1908, **46:**324-326.
3. de Wit HC: **A revision of Malaysian Bauhinieae.** *Reinwardtia* 1956, **3:**381-541.
4. Sharma AK, Raju DT: **Structure and behaviour of chromosomes in *Bauhinia* and allied genera.** *Cytologia* 1968, **33:**411-426.
5. Zhong Y, Chen Y, Zheng D, Pang J, Liu Y, Luo S, Meng S, Qian L, Wei D, Dai S, Zhou R: **Chromosomal-level genome assembly of the orchid tree *Bauhinia variegata* (Leguminosae; Cercidoideae) supports the allotetraploid origin hypothesis of Bauhinia.** *DNA Res* 2022, **29**.
6. Mak CY, Cheung KS, Yip PY, Kwan HS: **Molecular evidence for the hybrid origin of *Bauhinia blakeana* (Caesalpinioideae).** *J Integr Plant Biol* 2008, **50:**111-118.
7. Yuping L, Yingxiong Q, Chan YSG: **Dentification of Three Species in Bauhinia and Hybrid Origin of *Bauhinia blakeana* Using ISSR Markers.** *Acta Horticulturae Sinica* 2006, **33:**433.
8. Satam H, Joshi K, Mangrolia U, Waghoo S, Zaidi G, Rawool S, Thakare RP, Banday S, Mishra AK, Das G, Malonia SK: **Next-Generation Sequencing Technology: Current Trends and Advancements.** *Biology (Basel)* 2023, **12**.
9. Michael TP, VanBuren R: **Building near-complete plant genomes.** *Curr Opin Plant Biol* 2020, **54:**26-33.
10. Kress WJ, Soltis DE, Kersey PJ, Wegrzyn JL, Leebens-Mack JH, Gostel MR, Liu X, Soltis PS: **Green plant genomes: What we know in an era of rapidly expanding opportunities.** *Proc Natl Acad Sci U S A* 2022, **119**.
11. Garg S, Fungtammasan A, Carroll A, Chou M, Schmitt A, Zhou X, Mac S, Peluso P, Hatas E, Ghurye J: **Accurate chromosome-scale haplotype-resolved assembly of human genomes.** *Nat Biotechnol. 2021.doi: 10.1038/s41587-020-0711-0.*
12. Koren S, Rhie A, Walenz BP, Dilthey AT, Bickhart DM, Kingan SB, Hiendleder S, Williams JL, Smith TP, Phillippy AM: **De novo assembly of haplotype-resolved genomes with trio binning.** *Nature biotechnology* 2018, **36:**1174-1182.
13. Zhang X, Zhang S, Zhao Q, Ming R, Tang H: **Assembly of allele-aware, chromosomal-scale autopolyploid genomes based on Hi-C data.** *Nature plants* 2019, **5:**833-845.

14. Lazarus S: **Unique project to sequence the genome of the Hong Kong bauhinia tree.** SCMP; 2015. https://www.scmp.com/magazines/post-magazine/article/1875917/unique-project-sequence-genome-hong-kong-bauhinia-tree
15. Tang WM; Kwok JSL; Bauhinia Genome community; Tsui KW (2018): Transcriptome assemblies of three *Bauhinia* species. GigaScience Database. https://doi.org/10.5524/100345
16. Huang X, Yang S, Gong J, Zhao Q, Feng Q, Zhan Q, Zhao Y, Li W, Cheng B, Xia J, et al: **Genomic architecture of heterosis for yield traits in rice.** *Nature* 2016, **537:**629-633.
17. Baranwal VK, Mikkilineni V, Zehr UB, Tyagi AK, Kapoor S: **Heterosis: emerging ideas about hybrid vigour.** *Journal of Experimental Botany* 2012, **63:**6309-6314.
18. Chen ZJ: **Genomic and epigenetic insights into the molecular bases of heterosis.** *Nature Reviews Genetics* 2013, **14:**471-482.
19. Hochholdinger F, Baldauf JA: **Heterosis in plants.** *Current Biology* 2018, **28:**R1089-R1092.
20. Swanson-Wagner RA, Jia Y, DeCook R, Borsuk LA, Nettleton D, Schnable PS: **All possible modes of gene action are observed in a global comparison of gene expression in a maize F1 hybrid and its inbred parents.** *Proceedings of the National Academy of Sciences* 2006, **103:**6805-6810.
21. Ma X, Xing F, Jia Q, Zhang Q, Hu T, Wu B, Shao L, Zhao Y, Zhang Q, Zhou D-X: **Parental variation in CHG methylation is associated with allelic-specific expression in elite hybrid rice.** *Plant Physiology* 2021, **186:**1025-1041.
22. Li D, Lu X, Zhu Y, Pan J, Zhou S, Zhang X, Zhu G, Shang Y, Huang S, Zhang C: **The multi-omics basis of potato heterosis.** *Journal of Integrative Plant Biology* 2022, **64:**671-687.
23. Springer NM, Stupar RM: **Allele-specific expression patterns reveal biases and embryo-specific parent-of-origin effects in hybrid maize.** *Plant Cell* 2007, **19:**2391-2402.
24. Shao L, Xing F, Xu CH, Zhang QH, Che J, Wang XM, Song JM, Li XH, Xiao JH, Chen LL, et al: **Patterns of genome-wide allele-specific expression in hybrid rice and the implications on the genetic basis of heterosis.** *PNAS* 2019, **116:**5653-5658.
25. Chikhi R, Medvedev P: **Informed and automated k-mer size selection for genome assembly.** *Bioinformatics* 2014, **30:**31-37.
26. Weisenfeld NI, Kumar V, Shah P, Church DM, Jaffe DB: **Direct determination of diploid genome sequences.** *Genome Res* 2017, **27:**757-767.
27. Simao FA, Waterhouse RM, Ioannidis P, Kriventseva EV, Zdobnov EM: **BUSCO: assessing genome assembly and annotation completeness with single-copy orthologs.** *Bioinformatics* 2015, **31:**3210-3212.
28. **Creating diploid assemblies from Nanopore and Illumina reads with hypo-assembler.** *Nat Methods* 2024, **21:**560-561.
29. Rhie A, Walenz BP, Koren S, Phillippy AM: **Merqury: reference-free quality, completeness, and phasing assessment for genome assemblies.** *Genome Biol* 2020, **21:** (1):245. doi: 10.1186/s13059-020-02134-9.
30. Boeckmann B, Bairoch A, Apweiler R, Blatter MC, Estreicher A, Gasteiger E, Martin MJ, Michoud K, O'Donovan C, Phan I, et al: **The SWISS-PROT protein knowledgebase and its supplement TrEMBL in 2003.** *Nucleic Acids Res* 2003, **31:**365-370.

31. Kanehisa M, Araki M, Goto S, Hattori M, Hirakawa M, Itoh M, Katayama T, Kawashima S, Okuda S, Tokimatsu T, Yamanishi Y: **KEGG for linking genomes to life and the environment.** *Nucleic Acids Res* 2008, **36:**D480-484.
32. Tatusov RL, Fedorova ND, Jackson JD, Jacobs AR, Kiryutin B, Koonin EV, Krylov DM, Mazumder R, Mekhedov SL, Nikolskaya AN, et al: **The COG database: an updated version includes eukaryotes.** *BMC Bioinformatics* 2003, **4:**41.
33. Hunter S, Apweiler R, Attwood TK, Bairoch A, Bateman A, Binns D, Bork P, Das U, Daugherty L, Duquenne L, et al: **InterPro: the integrative protein signature database.** *Nucleic Acids Res* 2009, **37:**D211-215.
34. Lin Y, Ye C, Li X, Chen Q, Wu Y, Zhang F, Pan R, Zhang S, Chen S, Wang X, et al: **quarTeT: a telomere-to-telomere toolkit for gap-free genome assembly and centromeric repeat identification.** *Hortic Res* 2023, **10:**uhad127.
35. Goel M, Sun H, Jiao WB, Schneeberger K: **SyRI: finding genomic rearrangements and local sequence differences from whole-genome assemblies.** *Genome Biol* 2019, **20:**277.
36. Chen F, Tholl D, Bohlmann J, Pichersky E: **The family of terpene synthases in plants: a mid-size family of genes for specialized metabolism that is highly diversified throughout the kingdom.** *Plant J* 2011, **66:**212-229.
37. Aubourg S, Lecharny A, Bohlmann J: **Genomic analysis of the terpenoid synthase (AtTPS) gene family of Arabidopsis thaliana.** *Mol Genet Genomics* 2002, **267:**730-745.
38. Thompson JD, Higgins DG, Gibson TJ: **CLUSTAL W: improving the sensitivity of progressive multiple sequence alignment through sequence weighting, position-specific gap penalties and weight matrix choice.** *Nucleic acids research* 1994, **22:**4673-4680.
39. Frazer KA, Pachter L, Poliakov A, Rubin EM, Dubchak I: **VISTA: computational tools for comparative genomics.** *Nucleic Acids Res* 2004, **32:**W273-279.
40. Xiao Y, Qu YY, Hao CH, Tang L, Zhang JL: **The complete chloroplast genome of *Bauhinia racemosa* Lam. (Fabaceae): a versatile tropical medicinal plant.** *Mitochondrial DNA B Resour* 2022, **7:**1528-1530.
41. Shang H-Y, Jia K-H, Li N-W, Zhou M-J, Yang H, Tian X-L, Ma Y-P, Zhang R-G: **Phytop: A tool for visualizing and recognizing signals of incomplete lineage sorting and hybridization using species trees output from ASTRAL.** *Horticulture Research* 2024**:**uhae330.
42. Stupar RM, Springer NM: **Cis-transcriptional variation in maize inbred lines B73 and Mo17 leads to additive expression patterns in the F1 hybrid.** *Genetics* 2006, **173:**2199-2210.
43. Carretero-Paulet L, Ahumada I, Cunillera N, Rodriguez-Concepcion M, Ferrer A, Boronat A, Campos N: **Expression and molecular analysis of the Arabidopsis DXR gene encoding 1-deoxy-D-xylulose 5-phosphate reductoisomerase, the first committed enzyme of the 2-C-methyl-D-erythritol 4-phosphate pathway.** *Plant Physiol* 2002, **129:**1581-1591.
44. Carretero-Paulet L, Cairo A, Botella-Pavía P, Besumbes O, Campos N, Boronat A, Rodríguez-Concepción M: **Enhanced flux through the methylerythritol 4-phosphate pathway in Arabidopsis plants overexpressing deoxyxylulose 5-phosphate**

reductoisomerase. *Plant molecular biology* 2006, **62:**683-695.
45. Cochrane FC, Davin LB, Lewis NG: **The Arabidopsis phenylalanine ammonia lyase gene family: kinetic characterization of the four PAL isoforms.** *Phytochemistry* 2004, **65:**1557-1564.
46. Lichtenthaler HK: **The 1-Deoxy-D-Xylulose-5-Phosphate Pathway of Isoprenoid Biosynthesis in Plants.** *Annu Rev Plant Physiol Plant Mol Biol* 1999, **50:**47-65.
47. Gudavalli D, Pandey K, Ede VG, Sable D, Ghagare AS, Kate AS: **Phytochemistry and pharmacological activities of five species of Bauhinia genus: A review.** *Fitoterapia* 2024, **174:**105830. doi: 10.1016/j.fitote.2024.105830.
48. da Fonseca STD, Teixeira TR, Ferreira JMS, Lima L, Luyten W, Castro AHF: **Flavonoid-Rich Fractions of *Bauhinia holophylla* Leaves Inhibit *Candida albicans* Biofilm Formation and Hyphae Growth.** *Plants (Basel)* 2022, **11**.
49. Chinnappan S, Kandasamy S, Arumugam S, Seralathan KK, Thangaswamy S, Muthusamy G: **Biomimetic synthesis of silver nanoparticles using flower extract of *Bauhinia purpurea* and its antibacterial activity against clinical pathogens.** *Environ Sci Pollut Res Int* 2018, **25:**963-969.
50. Mishra A, Sharma AK, Kumar S, Saxena AK, Pandey AK: ***Bauhinia variegata* leaf extracts exhibit considerable antibacterial, antioxidant, and anticancer activities.** *Biomed Res Int* 2013, **2013:**915436.
51. Liu L, Guan D, Peart MR: **The morphological structure of leaves and the dust-retaining capability of afforested plants in urban Guangzhou, South China.** *Environ Sci Pollut Res Int* 2012, **19:**3440-3449.
52. Seymour DK, Chae E, Grimm DG, Martin Pizarro C, Habring-Müller A, Vasseur F, Rakitsch B, Borgwardt KM, Koenig D, Weigel D: **Genetic architecture of nonadditive inheritance in Arabidopsis thaliana hybrids.** *PNAS* 2016, **113:**E7317-E7326.
53. Birchler JA, Yao H, Chudalayandi S, Vaiman D, Veitia RA: **Heterosis.** *The Plant Cell* 2010, **22:**2105-2112.
54. Xiao J, Li J, Yuan L, Tanksley SD: **Dominance is the major genetic basis of heterosis in rice as revealed by QTL analysis using molecular markers.** *Genetics* 1995, **140:**745-754.
55. Li Z-K, Luo L, Mei H, Wang D, Shu Q, Tabien R, Zhong D, Ying C, Stansel J, Khush G: **Overdominant epistatic loci are the primary genetic basis of inbreeding depression and heterosis in rice. I. Biomass and grain yield.** *Genetics* 2001, **158:**1737-1753.
56. Janko K, Eisner J, Cigler P, Tichopad T: **Unifying framework explaining how parental regulatory divergence can drive gene expression in hybrids and allopolyploids.** *Nat Commun* 2024, **15:**8714.
57. Combes M-C, Hueber Y, Dereeper A, Rialle S, Herrera J-C, Lashermes P: **Regulatory Divergence between Parental Alleles Determines Gene Expression Patterns in Hybrids.** *Genome Biology and Evolution* 2015, **7:**1110-1121.
58. Matthews PD, Wurtzel ET: **Metabolic engineering of carotenoid accumulation in Escherichia coli by modulation of the isoprenoid precursor pool with expression of deoxyxylulose phosphate synthase.** *Applied Microbiology and Biotechnology* 2000, **53:**396-400.
59. Carretero-Paulet L, Cairó A, Botella-Pavía P, Besumbes O, Campos N, Boronat A, Rodríguez-Concepción M: **Enhanced flux through the methylerythritol 4-phosphate**

pathway in Arabidopsis plants overexpressing deoxyxylulose 5-phosphate reductoisomerase. *Plant Molecular Biology* 2006, **62:**683-695.

60. Springer NM, Stupar RM: **Allelic variation and heterosis in maize: how do two halves make more than a whole?** *Genome research* 2007, **17:**264-275.
61. Guo M, Rupe MA, Yang X, Crasta O, Zinselmeier C, Smith OS, Bowen B: **Genome-wide transcript analysis of maize hybrids: allelic additive gene expression and yield heterosis.** *Theoretical and Applied Genetics* 2006, **113**(5)**:**831-845. doi: 10.1007/s00122-006-0335-x.
62. Goff SA, Zhang Q: **Heterosis in elite hybrid rice: speculation on the genetic and biochemical mechanisms.** *Current opinion in plant biology* 2013, **16:**221-227.
63. Sahu SK, Thangaraj M, Kathiresan K: **DNA Extraction Protocol for Plants with High Levels of Secondary Metabolites and Polysaccharides without Using Liquid Nitrogen and Phenol.** *ISRN Mol Biol* 2012, **2012:**205049.
64. Wang O, Chin R, Cheng X, Wu MKY, Mao Q, Tang J, Sun Y, Anderson E, Lam HK, Chen D, et al: **Efficient and unique cobarcoding of second-generation sequencing reads from long DNA molecules enabling cost-effective and accurate sequencing, haplotyping, and de novo assembly.** *Genome Res* 2019, **29:**798-808.
65. Lieberman-Aiden E, Van Berkum NL, Williams L, Imakaev M, Ragoczy T, Telling A, Amit I, Lajoie BR, Sabo PJ, Dorschner MO: **Comprehensive mapping of long-range interactions reveals folding principles of the human genome.** *Science* 2009, **326:**289-293.
66. Liang XM et al. **BGISEQ-500 WGS library construction.** *Protocols.io. 2018. https://dx.doi.org/10.17504/protocols.io.ps5dng6.*
67. Ashton PM, Nair S, Dallman T, Rubino S, Rabsch W, Mwaigwisya S, Wain J, O'grady J: **MinION nanopore sequencing identifies the position and structure of a bacterial antibiotic resistance island.** *Nature biotechnology* 2015, **33:**296-300.
68. Bolger AM, Lohse M, Usadel B: **Trimmomatic: a flexible trimmer for Illumina sequence data.** *Bioinformatics* 2014, **30:**2114-2120.
69. Marcais G, Kingsford C: **A fast, lock-free approach for efficient parallel counting of occurrences of k-mers.** *Bioinformatics* 2011, **27:**764-770.
70. Vurture GW, Sedlazeck FJ, Nattestad M, Underwood CJ, Fang H, Gurtowski J, Schatz MC: **GenomeScope: fast reference-free genome profiling from short reads.** *Bioinformatics* 2017, **33:**2202-2204.
71. Weisenfeld NI, Kumar V, Shah P, Church DM, Jaffe DB: **Direct determination of diploid genome sequences.** *Genome research* 2017, **27:**757-767.
72. Li H, Durbin R: **Fast and accurate short read alignment with Burrows-Wheeler transform.** *Bioinformatics* 2009, **25:**1754-1760.
73. Danecek P, Bonfield JK, Liddle J, Marshall J, Ohan V, Pollard MO, Whitwham A, Keane T, McCarthy SA, Davies RM, Li H: **Twelve years of SAMtools and BCFtools.** *Gigascience* 2021, **10**. doi: 10.1093/gigascience/giab008.
74. **Repeat Library Construction-Advanced** [http://weatherby.genetics.utah.edu/MAKER/wiki/index.php/Repeat_Library_Construction-Advanced]. Accessed December 12, 2024.
75. Chen N: **Using RepeatMasker to identify repetitive elements in genomic sequences.**

Curr Protoc Bioinformatics 2004, Chapter 4:Unit 4.10. doi: 10.1002/0471250953.bi0410s05.
76. Bao WD, Kojima KK, Kohany O: **Repbase Update, a database of repetitive elements in eukaryotic genomes.** *Mobile DNA* 2015, **6**. doi: 10.1186/s13100-015-0041-9.
77. Xu Z, Wang H: **LTR_FINDER: an efficient tool for the prediction of full-length LTR retrotransposons.** *Nucleic Acids Research* 2007, **35:**W265-W268.
78. Flynn JM, Hubley R, Goubert C, Rosen J, Clark AG, Feschotte C, Smit AF: **RepeatModeler2 for automated genomic discovery of transposable element families.** *Proc Natl Acad Sci U S A* 2020, **117:**9451-9457.
79. Benson G: **Tandem repeats finder: a program to analyze DNA sequences.** *Nucleic Acids Research* 1999, **27:**573-580.
80. Bruna T, Hoff KJ, Lomsadze A, Stanke M, Borodovsky M: **BRAKER2: automatic eukaryotic genome annotation with GeneMark-EP plus and AUGUSTUS supported by a protein database.** *Nar Genomics and Bioinformatics* 2021, **3**. doi: 10.1093/nargab/lqaa108.
81. Kim D, Paggi JM, Park C, Bennett C, Salzberg SL: **Graph-based genome alignment and genotyping with HISAT2 and HISAT-genotype.** *Nat Biotechnol* 2019, **37:**907-915.
82. Kriventseva EV, Kuznetsov D, Tegenfeldt F, Manni M, Dias R, Simao FA, Zdobnov EM: **OrthoDB v10: sampling the diversity of animal, plant, fungal, protist, bacterial and viral genomes for evolutionary and functional annotations of orthologs.** *Nucleic Acids Res* 2019, **47:**D807-D811.
83. Marcais G, Delcher AL, Phillippy AM, Coston R, Salzberg SL, Zimin A: **MUMmer4: A fast and versatile genome alignment system.** *PLoS Comput Biol* 2018, **14:**e1005944.
84. Lowe TM, Eddy SR: **tRNAscan-SE: a program for improved detection of transfer RNA genes in genomic sequence.** *Nucleic Acids Res* 1997, **25**(5):955-64. doi: 10.1093/nar/25.5.955.
85. Nawrocki EP, Kolbe DL, Eddy SR: **Infernal 1.0: inference of RNA alignments.** *Bioinformatics* 2009, **25:**1335-1337. doi: 10.1093/bioinformatics/btp157.
86. Zheng Y, Jiao C, Sun H, Rosli HG, Pombo MA, Zhang P, Banf M, Dai X, Martin GB, Giovannoni JJ, et al: **iTAK: A Program for Genome-wide Prediction and Classification of Plant Transcription Factors, Transcriptional Regulators, and Protein Kinases.** *Mol Plant* 2016, **9:**1667-1670.
87. Emms DM, Kelly S: **OrthoFinder: phylogenetic orthology inference for comparative genomics.** *Genome Biol* 2019, **20:**238. doi: 10.1186/s13059-019-1832-y.
88. Katoh K, Standley DM: **MAFFT Multiple Sequence Alignment Software Version 7: Improvements in Performance and Usability.** *Molecular Biology and Evolution* 2013, **30:**772-780.
89. Castresana J: **Selection of conserved blocks from multiple alignments for their use in phylogenetic analysis.** *Molecular Biology and Evolution* 2000, **17:**540-552.
90. Nguyen LT, Schmidt HA, von Haeseler A, Minh BQ: **IQ-TREE: a fast and effective stochastic algorithm for estimating maximum-likelihood phylogenies.** *Mol Biol Evol* 2015, **32:**268-274.
91. Zhang C, Rabiee M, Sayyari E, Mirarab S: **ASTRAL-III: polynomial time species tree reconstruction from partially resolved gene trees.** *BMC Bioinformatics* 2018, **19:**153.
92. Stamatakis A: **RAxML version 8: a tool for phylogenetic analysis and post-analysis of large phylogenies.** *Bioinformatics* 2014, **30:**1312-1313.

93. Yang Z: **PAML 4: phylogenetic analysis by maximum likelihood.** *Mol Biol Evol* 2007, **24:**1586-1591.
94. De Bie T, Cristianini N, Demuth JP, Hahn MW: **CAFE: a computational tool for the study of gene family evolution.** *Bioinformatics* 2006, **22:**1269-1271.
95. Huang da W, Sherman BT, Lempicki RA: **Bioinformatics enrichment tools: paths toward the comprehensive functional analysis of large gene lists.** *Nucleic Acids Res* 2009, **37:**1-13.
96. Chen S, Yang P, Jiang F, Wei Y, Ma Z, Kang L: **De novo analysis of transcriptome dynamics in the migratory locust during the development of phase traits.** *PLoS One* 2010, **5:**e15633. doi: 10.1371/journal.pone.0015633.
97. Jin JJ, Yu WB, Yang JB, Song Y, dePamphilis CW, Yi TS, Li DZ: **GetOrganelle: a fast and versatile toolkit for accurate de novo assembly of organelle genomes.** *Genome Biology* 2020, **21**. doi: 10.1186/s13059-020-02154-5.
98. Shi L, Chen H, Jiang M, Wang L, Wu X, Huang L, Liu C: **CPGAVAS2, an integrated plastome sequence annotator and analyzer.** *Nucleic Acids Res* 2019, **47:**W65-W73. doi: 10.1093/nar/gkz345.
99. Mayor C, Brudno M, Schwartz JR, Poliakov A, Rubin EM, Frazer KA, Pachter LS, Dubchak I: **VISTA : visualizing global DNA sequence alignments of arbitrary length.** *Bioinformatics* 2000, **16:**1046-1047. doi: 10.1093/bioinformatics/16.11.1046.
100. Roberts A, Pachter L: **Streaming fragment assignment for real-time analysis of sequencing experiments.** *Nat Methods* 2013, **10:**71-73. doi: 10.1038/nmeth.2251.
101. Love MI, Huber W, Anders S: **Moderated estimation of fold change and dispersion for RNA-seq data with DESeq2.** *Genome Biol* 2014, **15:**550. doi: 10.1186/s13059-014-0550-8.
102. Wang Y, Tang H, Debarry JD, Tan X, Li J, Wang X, Lee TH, Jin H, Marler B, Guo H, et al: **MCScanX: a toolkit for detection and evolutionary analysis of gene synteny and collinearity.** *Nucleic Acids Res* 2012, **40:**e49. doi: 10.1093/nar/gkr1293.
103. Mu W, Darian JC, Sung W, et al. Supporting data for "The Haplotype-resolved T2T Genome for *Bauhinia* x *blakeana* Sheds Light on the Genetic Basis of Flower Heterosis" GigaScience Database. 2025. https://doi.org/10.5524/102678
104. Mu W, Darian JC, Sung W, et al. Genome assembly of the Orchid *Tree Bauhinia purpurea* GigaScience Database. 2025. https://doi.org/10.5524/102680
105. Mu W, Darian JC, Sung W, et al. Genome assembly of the Orchid Tree *Bauhinia variegata* GigaScience Database. 2025. https://doi.org/10.5524/102681
106. Mu W, Darian JC, Sung W, et al. Genome assembly of the Hong Kong Orchid Tree *Bauhinia blakeana* GigaScience Database. 2025. https://doi.org/10.5524/102679

Table 1. Statistics for genome assembly and annotation of three *Bauhinia* species.

Species	*Bauhinia blakeana* Hmat	*Bauhinia blakeana* Hpat	*Bauhinia purpurea*	*Bauhinia variegata*
**Assembly feature**				
Estimated genome size	290,967,258	290,967,258	303,677,508	314,486,060
Assembled genome size	275,484,977	290,698,387	285,147,376	311,011,643
GC content	34.05%	34.22%	33.88%	34.04%
N50 of contigs (bp)	19,540,838	20,987,561	161,057	109,234
N50 of scaffold (bp)	19,540,838	20,987,561	1,475,774	2,613,106
Complete BUSCOs	C:99.0%[S:81.5%,D:17.5%],F:0.6%,M:0.4%	C:99.2%[S:78.6%,D:20.6%],F:0.7%,M:0.1%	C:97.8%[S:77.6%,D:20.2%],F:1.4%,M:0.8%	C:98.4%[S:77.0%,D:21.4%],F:1.2%,M:0.4%
**HIC**				
Anchor size	/	/	285,099,865	310,940,945
Anchor rate	/	/	99.98%	99.98%
Number of pseudochromosomes	14	14	14	14
N50 of scaffold (bp)	19,540,838	19,540,838	21,596,737	24,404,849
**Characteristics of protein-coding genes**				
Total number of protein-coding genes	37,804	37,956	38,735	40,111
Mean gene size (bp)	2615.06	2619.36	2602.43	2595.09
Mean CDS length (bp)	1120.11	1179.70	1192.31	1187.11
Mean exon number per gene	5.36	5.16	5.13	5.08
Mean exon length (bp)	208.89	228.60	232.52	233.74
Mean intron length (bp)	342.71	346.02	341.63	345.20
Complete BUSCOs	C:94.2%[S:78.4%,D:15.8%],F:4.0%,M:1.8%	C:96.4%[S:79.2%,D:17.2%],F:2.4%,M:1.2%	C:97.4%[S:78.4%,D:19.0%],F:1.4%,M:1.2%	C:97.8%[S:77.0%,D:20.8%],F:1.4%,M:0.8%
**Functional annotation by searching public databases**				
% of proteins with hits in NCBI nr database	97.25%	98.16%	97.80%	95.80%
% of proteins with hits in Swiss-Prot database	75.86%	78.54%	80.60%	79.54%
% of proteins with hits in KEGG database	70.12%	72.58%	74.49%	50.44%
% of proteins with hits in KOG database	70.77%	73.24%	75.01%	74.67%
% of proteins with hits in TrEMBL database	92.98%	94.23%	96.62%	96.30%

% of proteins with hits in Interpro database	93.68%	94.95%	96.83%	96.89%
% of proteins with functional annotation (combined)	99.98%	99.97%	99.98%	99.96%

Figure legends

Figure 1. Genome assemblies of *the Bauhinia* species.

(A) Circos plot of *B. purpurea* (Bpur1–Bpur14) and *B. variegata* (Bvar1–Bvar14) genome assemblies. Outer tracks depict pseudochromosomes with annotation tracks (from outer to inner): (a) gene density, (b) GC content, (c) repeat element density, (d) LTR retrotransposon density, (e) LTR/Copia subclass density, (f) LTR/Gypsy subclass density. Synteny blocks between species are visualized by internal links. **(B)** Haplotype-resolved Circos plot of *B. blakeana.* Maternal (Hmat1–Hmat14) and paternal (Hpat1–Hpat14) haplotypes are annotated with gene density and repetitive element distribution. **(C)** Structural variations (SVs) between *B. blakeana* haplotypes. SVs (inversions, translocations, and duplications) were identified using SyRI, with the maternal haplotype (Hmat) as the reference. Additional annotations include gene density, centromere regions, and telomere positions.

Figure 2. Comparative genomic analysis and terpene synthase (TPS) gene family expansion in *Bauhinia*. (A) Phylogenetic tree with divergence times of *Bauhinia* and related angiosperms. Divergence times (blue labels, million years ago) and gene family expansions (green) or contractions (red) at key evolutionary nodes are plotted. **(B)**

KEGG enrichment of expanded gene families in *Bauhinia*. The top 20 enriched pathways (adjusted $p < 0.05$) are plotted. Terpenoid backbone biosynthesis (ko00900) and related metabolic pathways are annotated. **(C)** Phylogenetic classification of terpene synthases (TPSs) in *Bauhinia*. Phylogenetic tree of TPS proteins showing six major subfamilies (TPS-a/b/c/e/f/g).

Figure 3. Genomic evidence for the maternal parentage and hybrid origin of *B. blakeana*. **(A)** Comparative analysis of six *Bauhinia* chloroplast (cp) genomes using mVISTA, with the assembled *B. blakeana* cp genome from this study as the reference. The y-axis represents percent identity (50%–100%). Grey arrows indicate transcriptional orientation. **(B)** Identification of a one-base pair deletion at position 116,948 in the published *B. purpurea* cp genome (NC061218). **(C)** Phylogenetic tree of the *Bauhinia* genus based on available cp genomes, with *C. canadensis* as the outgroup. **(D)** Phylogenetic tree of *B. blakeana* haplotypes (Hmat, Hpat) and parental species (*B. purpurea, B. variegata*), inferred from 2,360 single-copy nuclear genes using the ASTRAL method. ILS/IH indices are calculated and shown.

Figure 4. Comparative transcriptomics analyses.

(A) Global DEGs landscape across *Bauhinia* species comparisons. Pairwise comparisons include parental species (*B. purpurea* vs. *B. variegat*a), hybrids (*B. blakeana* vs. each parent), and mid-parent value (MPV) deviation (*B. blakeana* vs. MPV). Arrows represent the comparisons, with the numbers and proportions of up-

regulated DEGs indicated at the arrow ends. Reference genome impacts are color-coded (*B. purpurea*: red; *B. variegata*: blue). **(B)** Reference genome bias in DEG detection between parental species. Box plots compare log2|FC| distributions (binned: 1–2, 2–4, 4–8, >8) using alternate references. Both references yield comparable total DEGs. **(C)** Functional divergence of species-specific up-regulated genes. KEGG enrichment of *B. purpurea*-upregulated genes (pink), and *B. variegata*-upregulated genes (blue).

Figure 5. Transcriptomic divergence between *B. blakeana* and parental species.

(A) Distribution of DEGs between *B. blakeana* and parental species. Box plot illustrating the number and distribution of DEGs (DE1+, log2|FC| > 1) between *B. blakeana* and *B. purpurea* (with *B. purpurea* as the reference) and between *B. blakeana* and *B. variegata* (with *B. variegata* as the reference), binned by fold-change intervals (1–2, 2–4, 4–8, >8). **(B)** KEGG pathway enrichment of up-regulated DEGs in hybrid and parental comparisons. Enriched terms for *B. blakeana*-upregulated genes (purple), *B. purpurea*-upregulated genes (pink), and *B. variegata*-upregulated genes (blue). Arrows mark shared pathways from parental comparisons (red: *B. purpurea*; blue: *B. variegata*). **(C)** Distribution of DEGs between *B. blakeana* and MPV. Box plot illustrating the number and distribution of DEGs (DE1+, log2|FC| > 1) between *B. blakeana* and the MPV using *B. purpurea* (red) or *B. variegata* (blue) as references.

Figure 6. Allele-specific expression (ASE) landscape in *B. blakeana*.

(A) Volcano plot of ASEGs identified by diagnostic SNP-based method using the

HyLiTe pipeline. Dots represent ASEGs with red/blue indicating maternal/paternal allele dominance. **(B)** Volcano plot of ASEGs identified by the haplotype-resolved method. Dots represent ASEGs with red/blue indicating maternal/paternal allele dominance. **(C)** Genomic distribution of ASEGs. Maternal-dominant ASEGs mapped to maternal haplotype (Hmat1–Hmat14), paternal-dominant ASEGs to paternal haplotype (Hpat1–Hpat14). **(D)** Functional enrichment of ASEGs with parental allele bias. Top 12 GO enrichment results of ASEGs in *B. blakeana*, with paternal allele dominance ASEGs (blue dots) and maternal allele dominance ASEGs (red dots) within each GO category plotted.

Figure 7. Pigment biosynthesis dynamics and allelic regulation in *B. blakeana*. The carotenoid enzymatic genes are divided into three groups: "MEP Pathway", "Carotene Biosynthesis", and "Xanthophylls Biosynthesis". The chlorophyll enzymatic genes are divided into three groups: "Chlorophyll Biosynthesis", "Chlorophyll Cycle", and "Chlorophyll Degradation". **(A)** Anthocyanin pathway expression profiles across *Bauhinia* species. Heatmap of gene expression (FPKM) for anthocyanin biosynthesis genes. ASEGs (allele-specific expressed genes) are labeled with gene IDs (maternal allele dominance: red; paternal: blue). **(B)** Carotenoid pathway expression profiles across *Bauhinia* species. Expression profiles of *MEP Pathway*, *Carotene Biosynthesis*, and *Xanthophylls Biosynthesis* modules. ASEG annotations as in (A). **(C)** Chlorophyll pathway expression profiles across *Bauhinia* species. Expression patterns in *Biosynthesis*, *Cycle*, and *Degradation* modules. ASEG labels follow (A). **(D)**

Parental dominance and hybrid expression patterns in pigment pathways. Summed FPKM (per gene copy) heatmap. Colored dots after gene IDs indicate *B. blakeana* expression patterns: yellow (over-dominance), pink (high-parent dominance).

Supplementary Figure S1: Genome survey analysis of *Bauhinia* species. (A-C) *K*-mer spectra (k=21) for *B. purpurea*, *B. variegata*, and *B. blakeana* generated by GenomeScope 2.0. Peaks correspond to heterozygous (left) and homozygous (right) *k*-mer distributions. The x-axis refers to the *k*-mer coverage, and the y-axis refers to the frequency of the *k*-mer for a given coverage.

Supplementary Figure S2. Hi-C scaffolding of *Bauhinia* pseudochromosomes.

(A) *B. purpurea* chromatin contact matrix. **(B)** *B. variegata* matrix. Colors indicate contact frequency.

Supplementary Figure S3: Principal component analysis (PCA) plot of RNA-seq data.

PCA of transcriptomes (PC1 vs. PC2) for *B. purpurea*, *B. variegata*, and *B. blakeana* (n=3 replicates). Outlier sample VAR3 (grey) was excluded from downstream analysis.

Supplementary Figure S4: Reference genome bias in ortholog expression quantification.

The figure consists of eight violin box plots, each representing a different sample. The

violin plot shows the distribution of gene expression values, with the width indicating the density of data points at different expression levels. Welch's t-test was employed to compare the gene expression data between groups.

Supplementary Figure S5: Expression concordance between allelic and reference-based quantification.

(A) Bar plot illustrating the mean difference (paired *t*-test) in gene expression values for each of the *Bauhinia* samples, with consideration given to distinct reference genomes used (*B. purpurea* or *B. variegata*). **(B)** Bar plot illustrating the mean difference in expression values within *B. blakeana* samples when utilizing varying reference genomes. We compared the allelic expression using the *B. blakeana* haplotype metagenome with the overall expression values when using either *B. purpurea* or *B. variegata* as a reference.

Figure 1

Click here to access/download;Figure;Fig 1.pdf

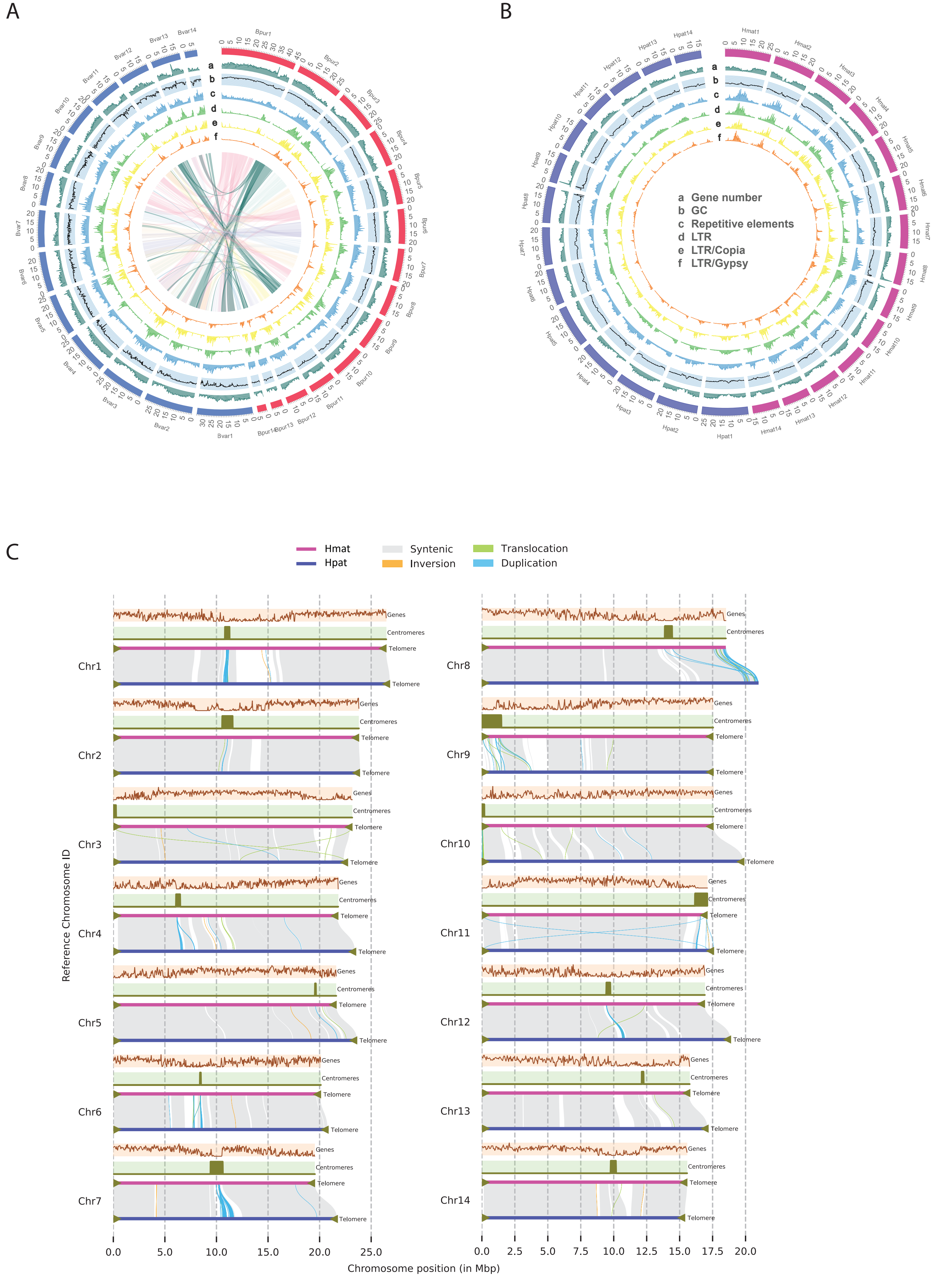

Figure 2 Click here to access/download;Figure;Fig 2.pdf

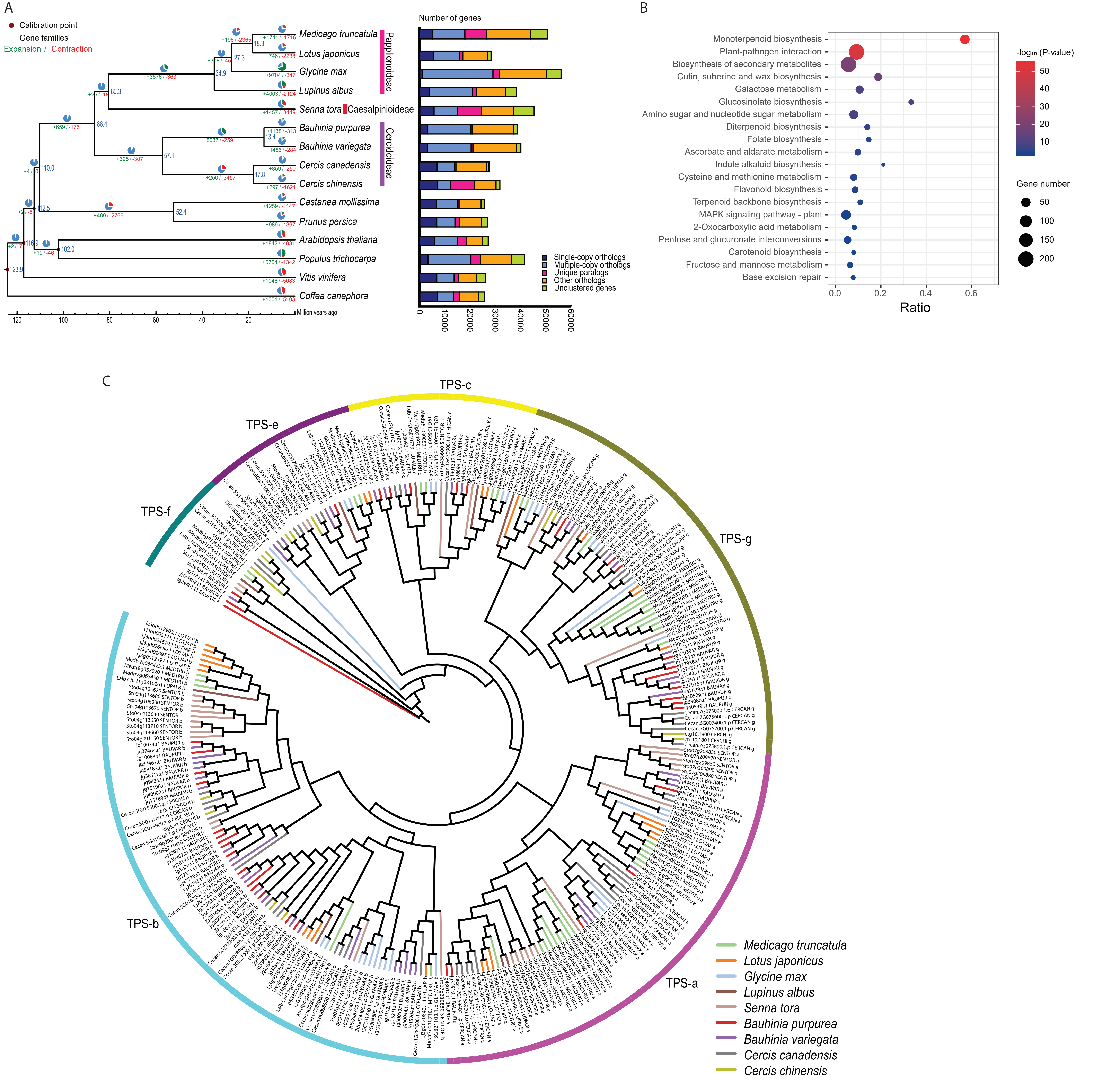

Figure 3

Click here to access/download;Figure;Figure 3.pdf

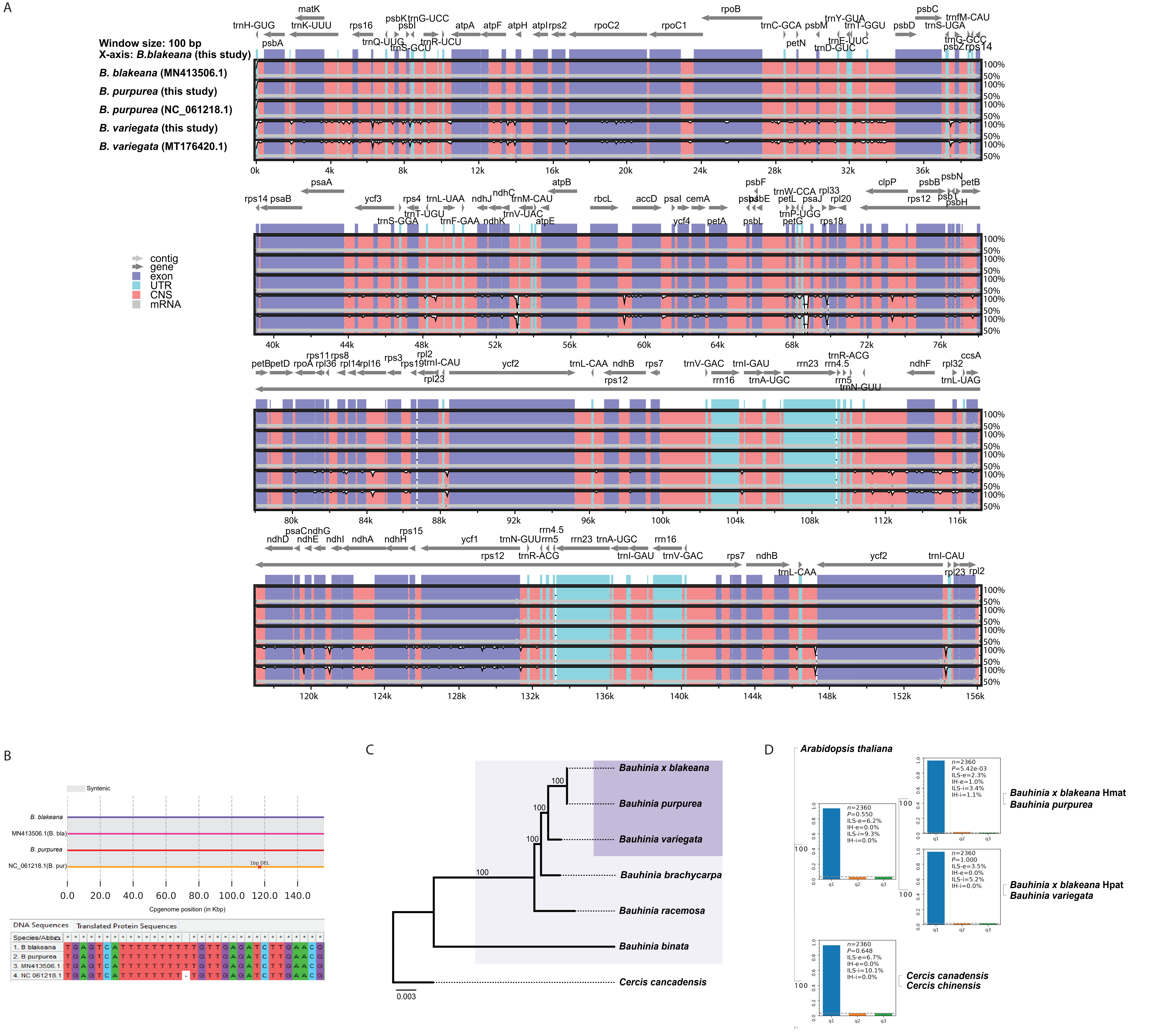

Figure 4

Click here to access/download;Figure;Figure 4.pdf

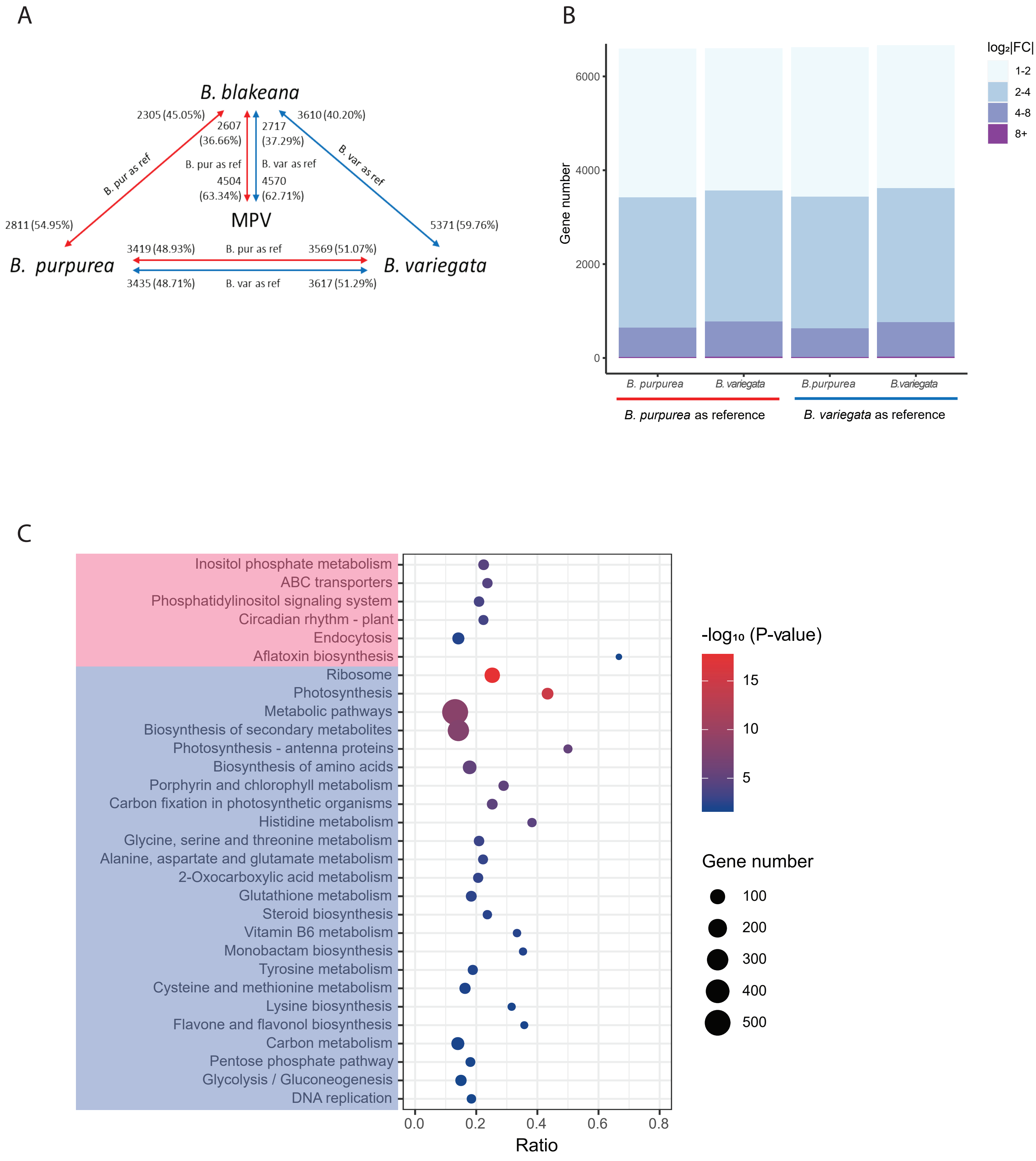

Figure 5

Click here to access/download;Figure;Figure 5.pdf

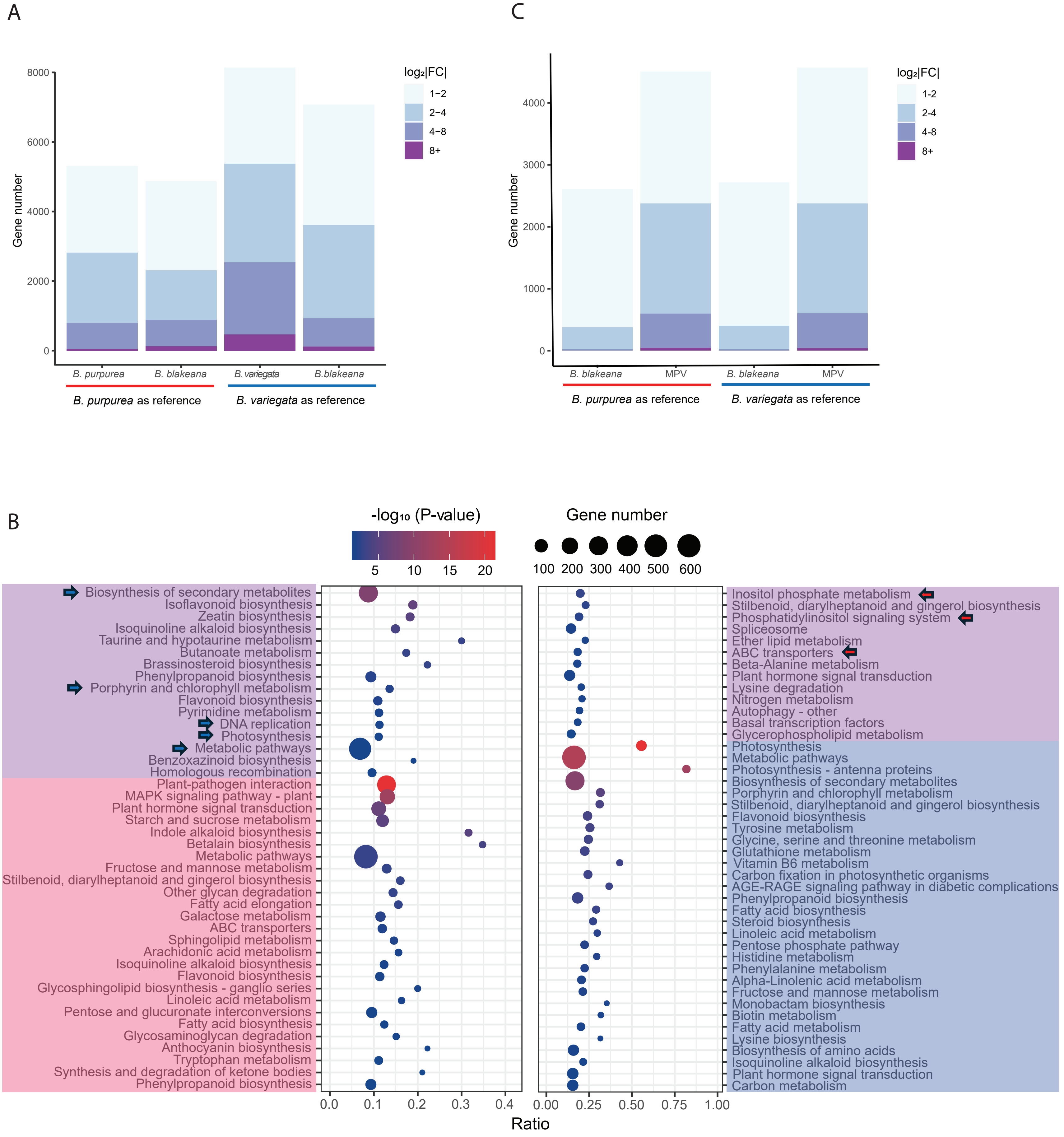

Figure 6

Click here to access/download;Figure;Fig 6.pdf

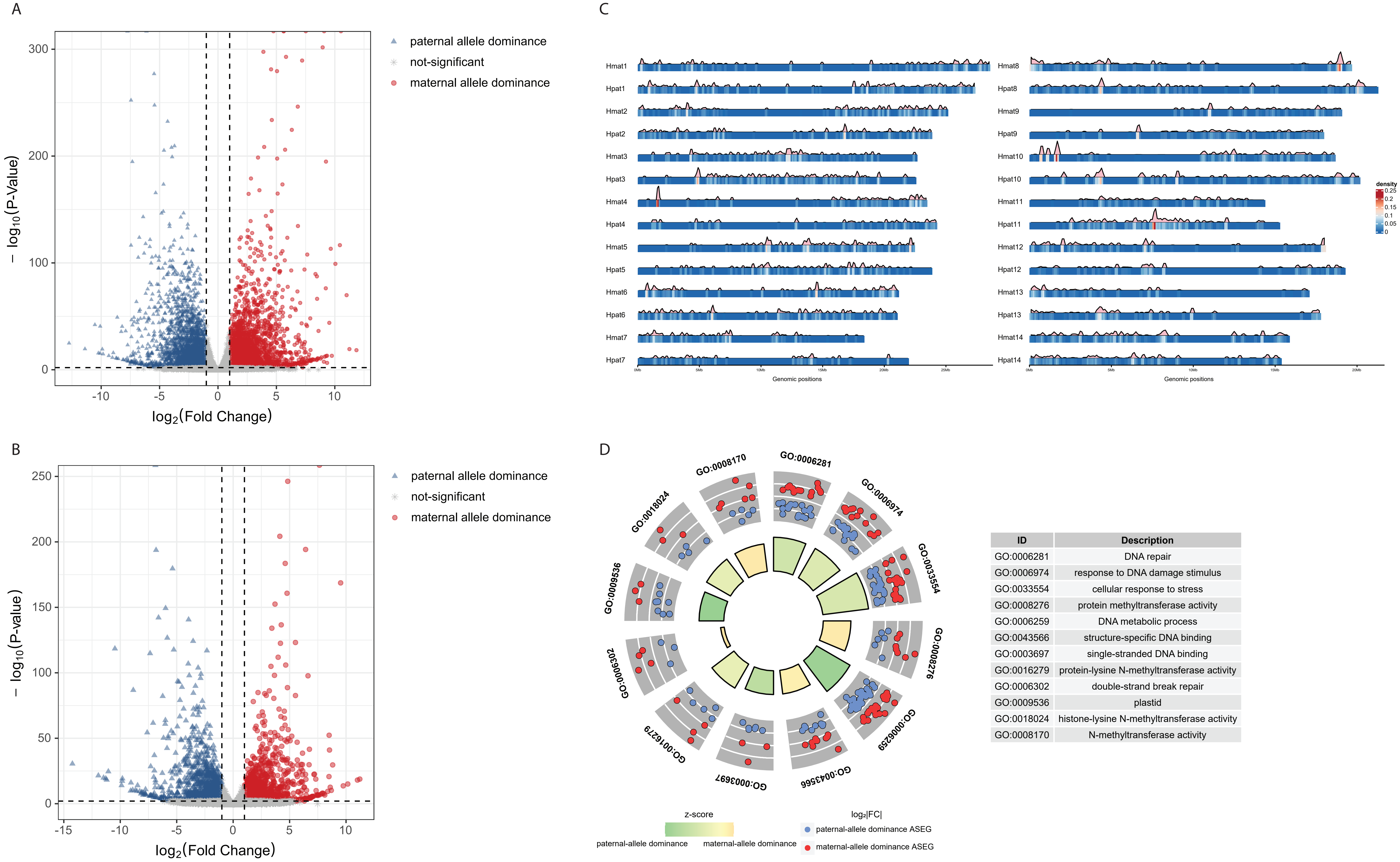

ID	Description
GO:0006281	DNA repair
GO:0006974	response to DNA damage stimulus
GO:0033554	cellular response to stress
GO:0008276	protein methyltransferase activity
GO:0006259	DNA metabolic process
GO:0043566	structure-specific DNA binding
GO:0003697	single-stranded DNA binding
GO:0016279	protein-lysine N-methyltransferase activity
GO:0006302	double-strand break repair
GO:0009536	plastid
GO:0018024	histone-lysine N-methyltransferase activity
GO:0008170	N-methyltransferase activity

Figure 7

Click here to access/download;Figure;Figure 7.pdf

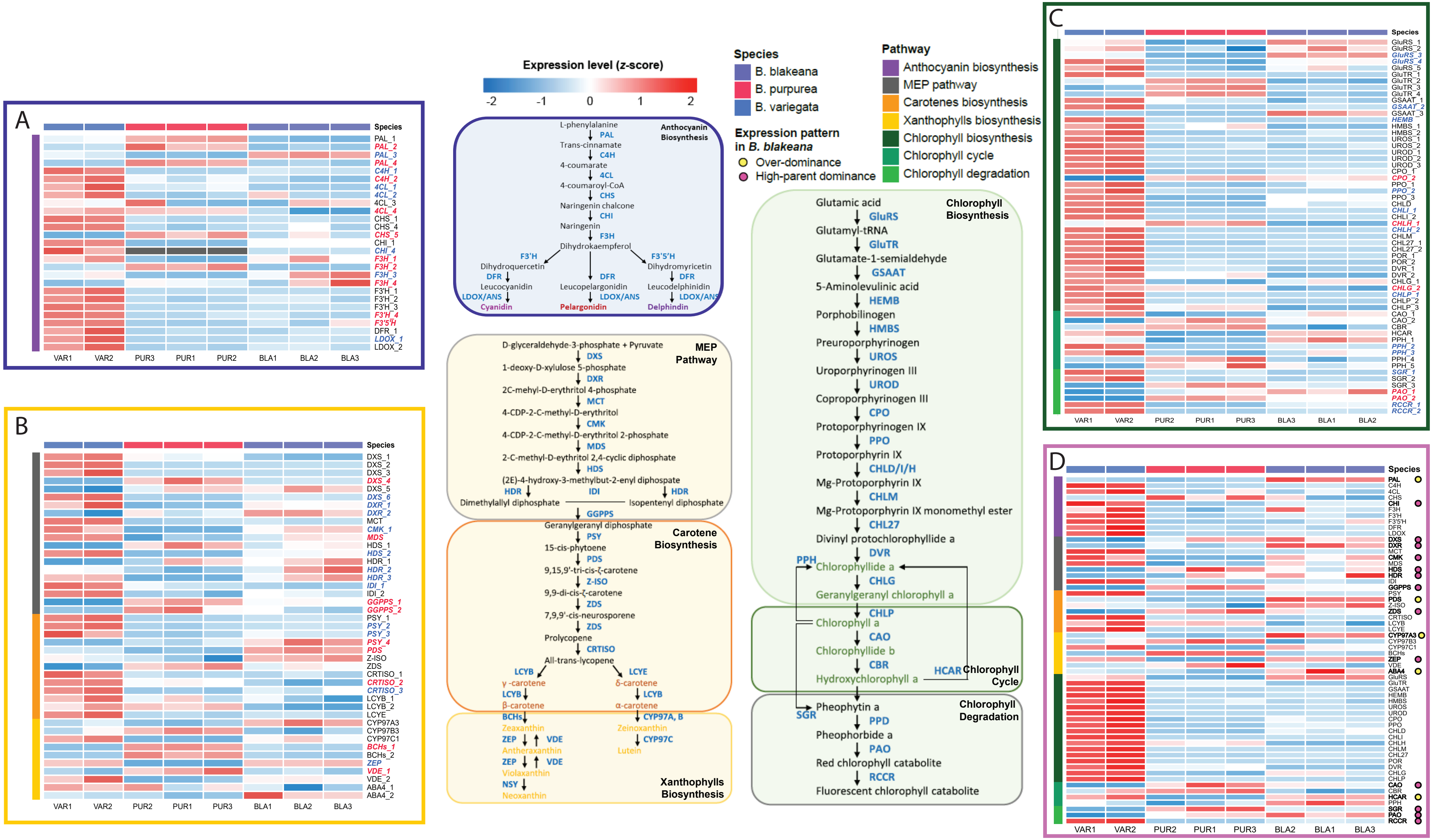

Supplementary Figure S1

Click here to access/download;Figure;Fig S1.pdf

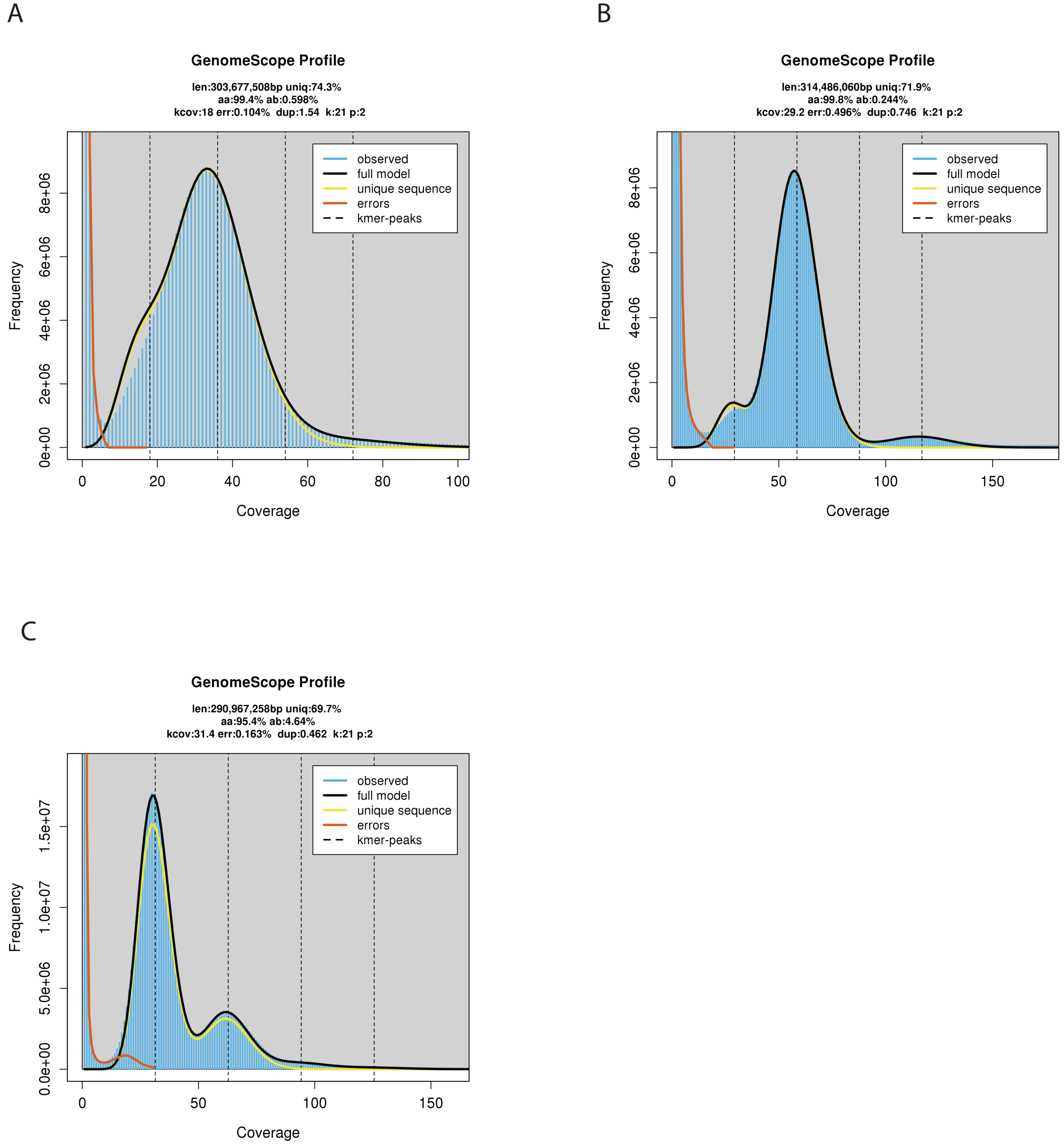

Supplementary Figure S2

Click here to access/download;Figure;Figure S2.pdf

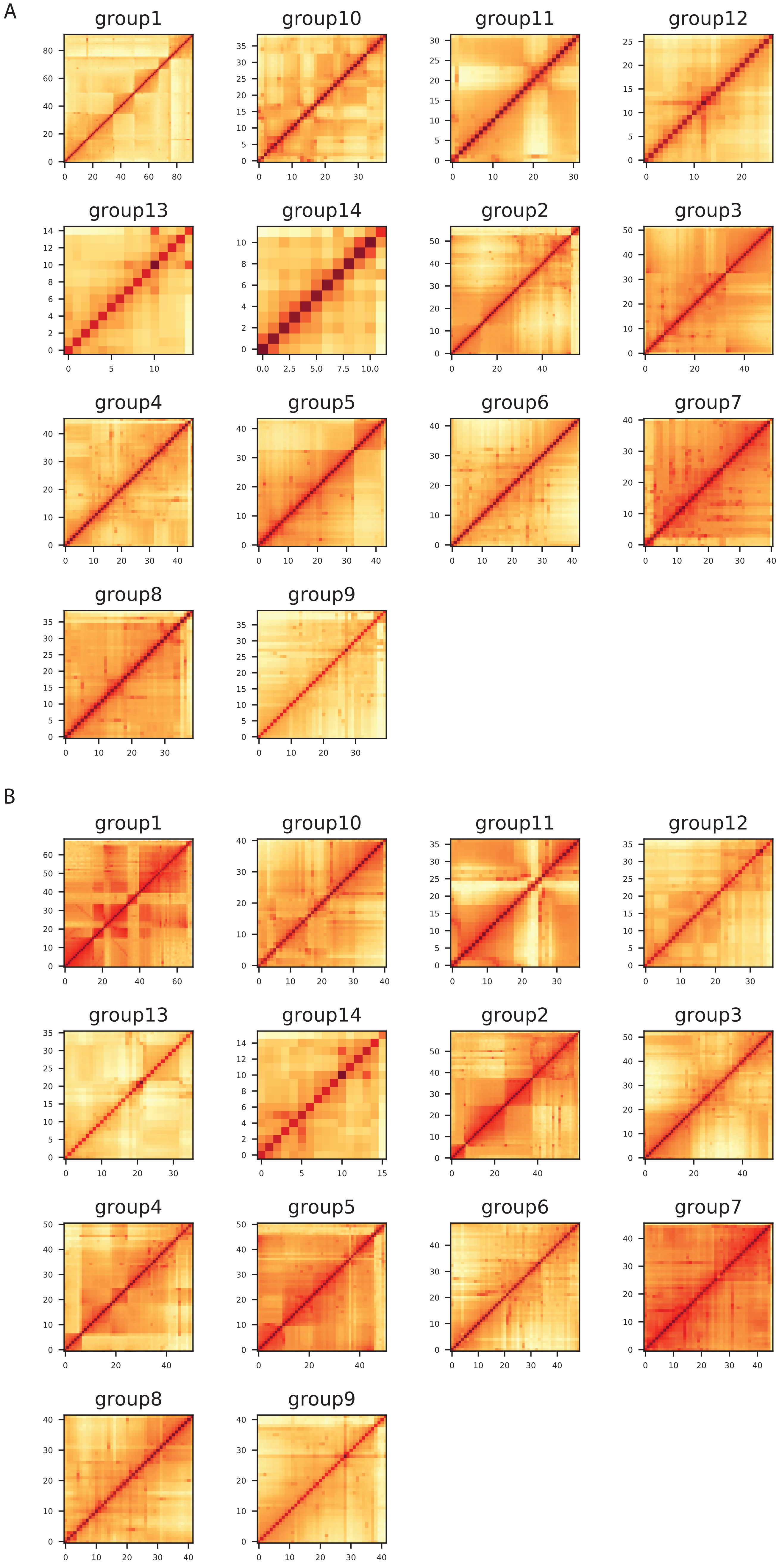

Supplementary Figure S3

Click here to access/download;Figure;Fig S3.pdf

A

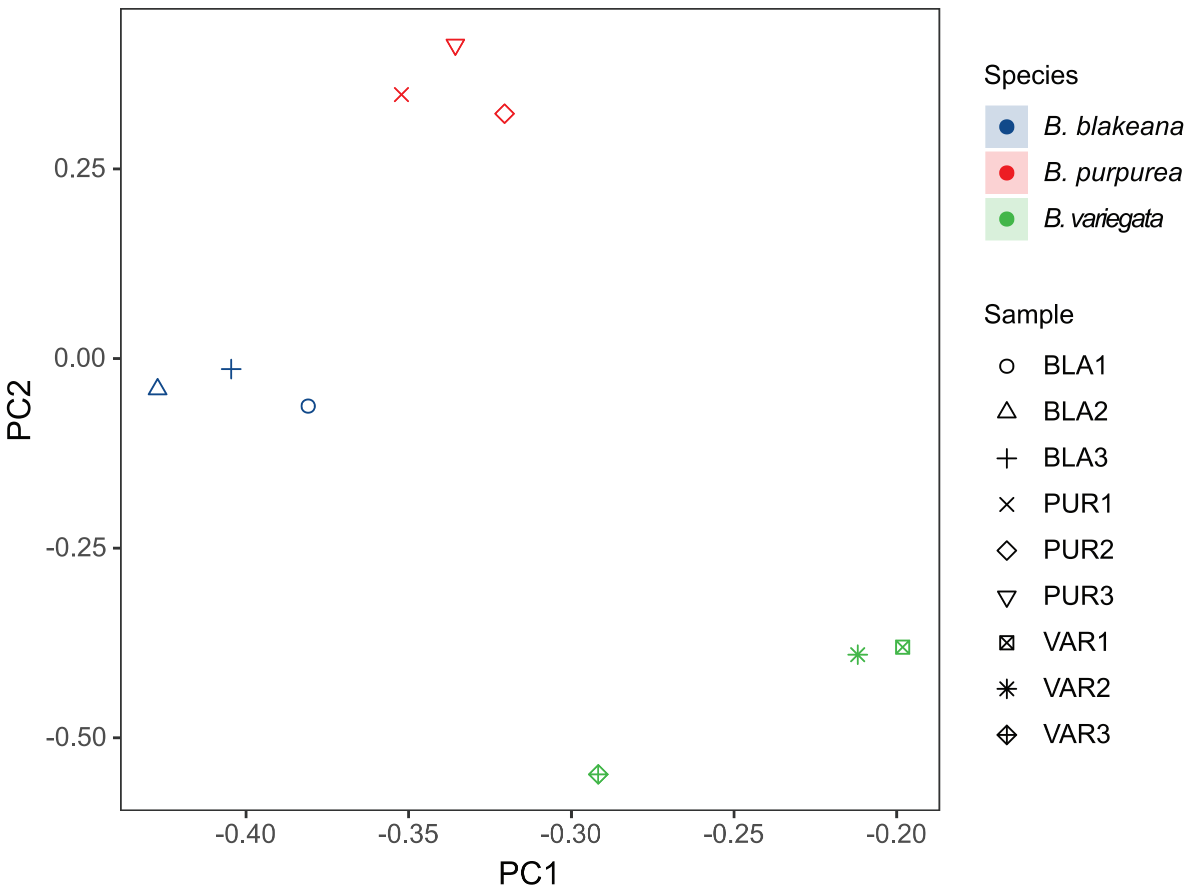

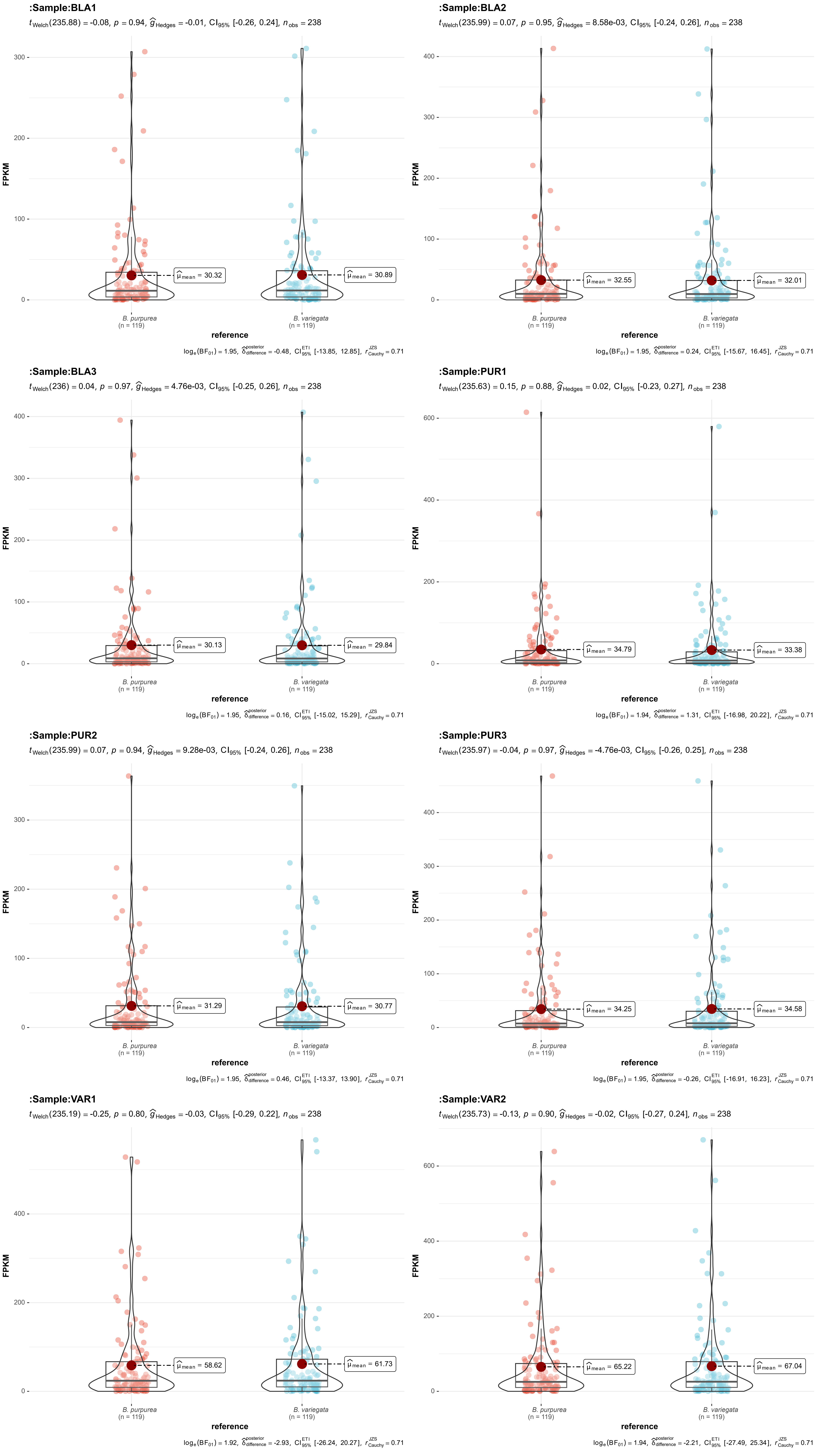
:Sample:BLA1
$t_{Welch}(235.88) = -0.08$, $p = 0.94$, $\hat{g}_{Hedges} = -0.01$, $CI_{95\%}$ [-0.26, 0.24], $n_{obs} = 238$
$\hat{\mu}_{mean} = 30.32$
$\hat{\mu}_{mean} = 30.89$
$\log_e(BF_{01}) = 1.95$, $\hat{\delta}^{posterior}_{difference} = -0.48$, $CI^{ETI}_{95\%}$ [-13.85, 12.85], $r^{JZS}_{Cauchy} = 0.71$
:Sample:BLA2
$t_{Welch}(235.99) = 0.07$, $p = 0.95$, $\hat{g}_{Hedges} = 8.58e-03$, $CI_{95\%}$ [-0.24, 0.26], $n_{obs} = 238$
$\hat{\mu}_{mean} = 32.55$
$\hat{\mu}_{mean} = 32.01$
$\log_e(BF_{01}) = 1.95$, $\hat{\delta}^{posterior}_{difference} = 0.24$, $CI^{ETI}_{95\%}$ [-15.67, 16.45], $r^{JZS}_{Cauchy} = 0.71$
:Sample:BLA3
$t_{Welch}(236) = 0.04$, $p = 0.97$, $\hat{g}_{Hedges} = 4.76e-03$, $CI_{95\%}$ [-0.25, 0.26], $n_{obs} = 238$
$\hat{\mu}_{mean} = 30.13$
$\hat{\mu}_{mean} = 29.84$
$\log_e(BF_{01}) = 1.95$, $\hat{\delta}^{posterior}_{difference} = 0.16$, $CI^{ETI}_{95\%}$ [-15.02, 15.29], $r^{JZS}_{Cauchy} = 0.71$
:Sample:PUR1
$t_{Welch}(235.63) = 0.15$, $p = 0.88$, $\hat{g}_{Hedges} = 0.02$, $CI_{95\%}$ [-0.23, 0.27], $n_{obs} = 238$
$\hat{\mu}_{mean} = 34.79$
$\hat{\mu}_{mean} = 33.38$
$\log_e(BF_{01}) = 1.94$, $\hat{\delta}^{posterior}_{difference} = 1.31$, $CI^{ETI}_{95\%}$ [-16.98, 20.22], $r^{JZS}_{Cauchy} = 0.71$
:Sample:PUR2
$t_{Welch}(235.99) = 0.07$, $p = 0.94$, $\hat{g}_{Hedges} = 9.28e-03$, $CI_{95\%}$ [-0.24, 0.26], $n_{obs} = 238$
$\hat{\mu}_{mean} = 31.29$
$\hat{\mu}_{mean} = 30.77$
$\log_e(BF_{01}) = 1.95$, $\hat{\delta}^{posterior}_{difference} = 0.46$, $CI^{ETI}_{95\%}$ [-13.37, 13.90], $r^{JZS}_{Cauchy} = 0.71$
:Sample:PUR3
$t_{Welch}(235.97) = -0.04$, $p = 0.97$, $\hat{g}_{Hedges} = -4.76e-03$, $CI_{95\%}$ [-0.26, 0.25], $n_{obs} = 238$
$\hat{\mu}_{mean} = 34.25$
$\hat{\mu}_{mean} = 34.58$
$\log_e(BF_{01}) = 1.95$, $\hat{\delta}^{posterior}_{difference} = -0.26$, $CI^{ETI}_{95\%}$ [-16.91, 16.23], $r^{JZS}_{Cauchy} = 0.71$
:Sample:VAR1
$t_{Welch}(235.19) = -0.25$, $p = 0.80$, $\hat{g}_{Hedges} = -0.03$, $CI_{95\%}$ [-0.29, 0.22], $n_{obs} = 238$
$\hat{\mu}_{mean} = 58.62$
$\hat{\mu}_{mean} = 61.73$
$\log_e(BF_{01}) = 1.92$, $\hat{\delta}^{posterior}_{difference} = -2.93$, $CI^{ETI}_{95\%}$ [-26.24, 20.27], $r^{JZS}_{Cauchy} = 0.71$
:Sample:VAR2
$t_{Welch}(235.73) = -0.13$, $p = 0.90$, $\hat{g}_{Hedges} = -0.02$, $CI_{95\%}$ [-0.27, 0.24], $n_{obs} = 238$
$\hat{\mu}_{mean} = 65.22$
$\hat{\mu}_{mean} = 67.04$
$\log_e(BF_{01}) = 1.94$, $\hat{\delta}^{posterior}_{difference} = -2.21$, $CI^{ETI}_{95\%}$ [-27.49, 25.34], $r^{JZS}_{Cauchy} = 0.71$
FPKM
reference
B. purpurea (n = 119)
B. variegata (n = 119)

Supplementary Figure S5

Click here to access/download;Figure;Figure S5.pdf

A

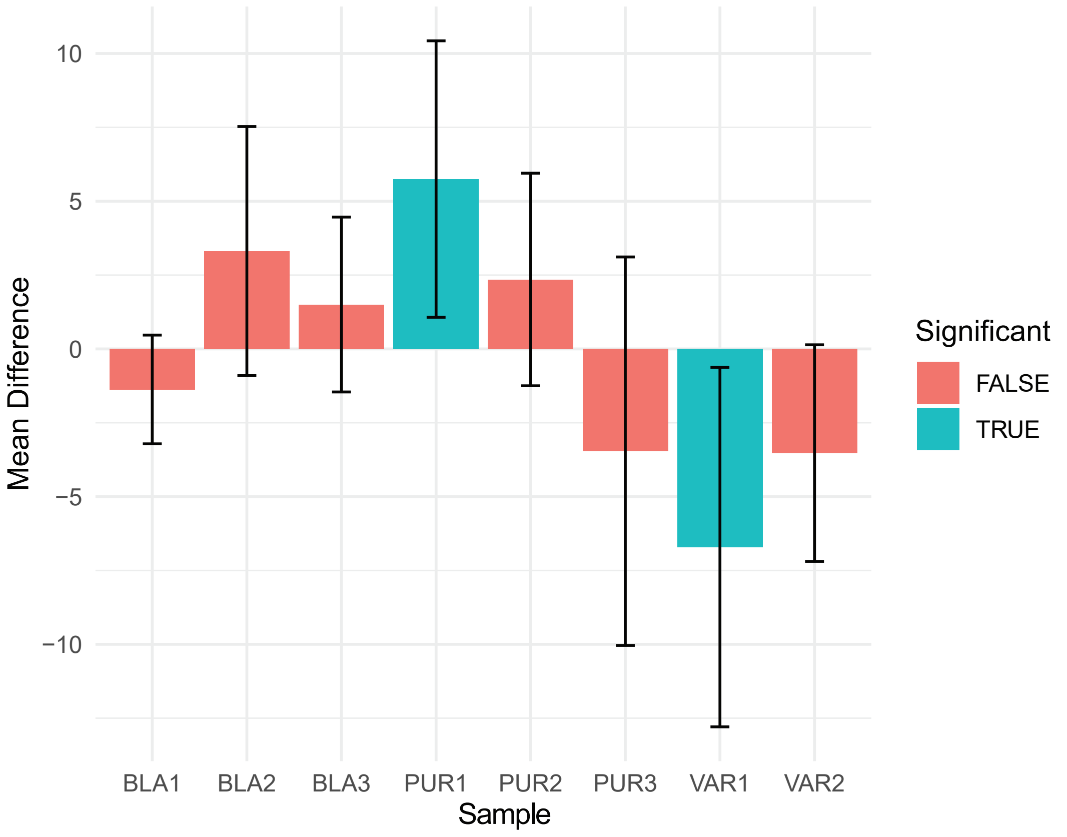

B

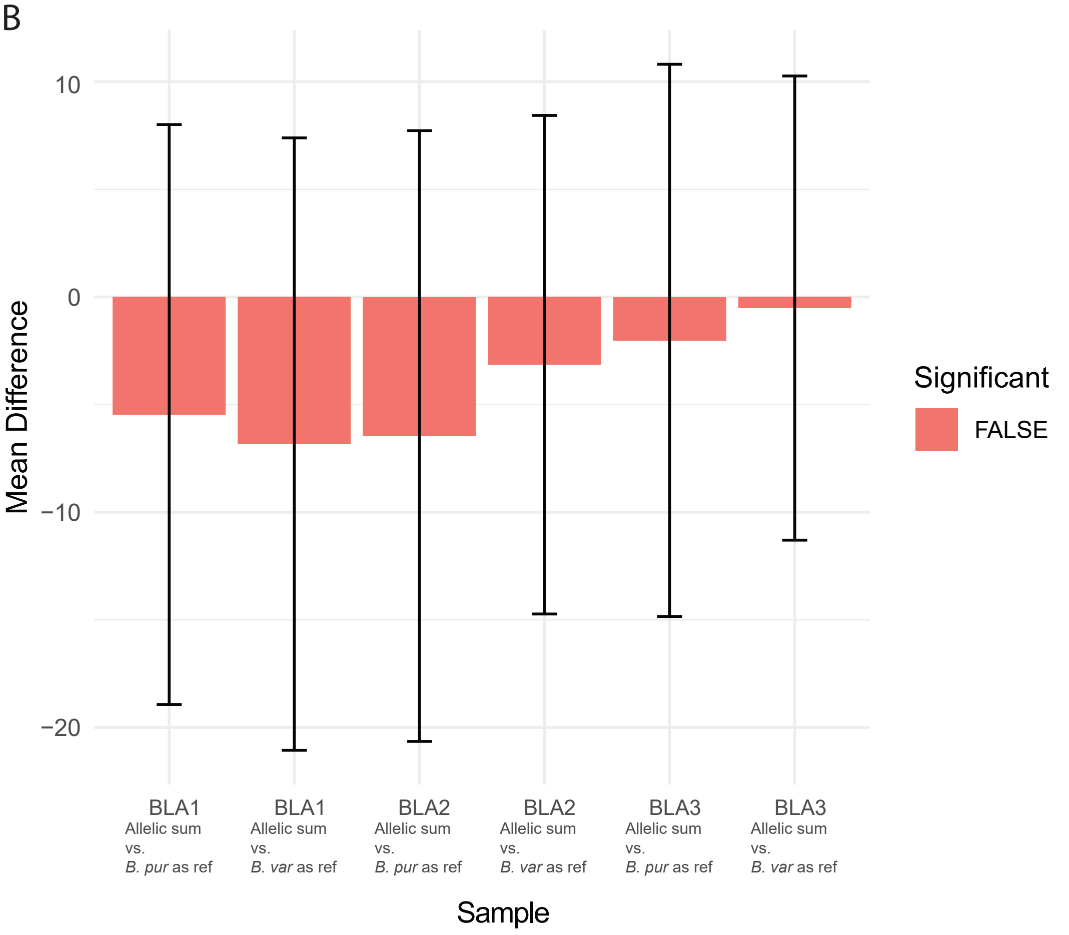

Supplementary Table S1-26

Click here to access/download

Supplementary Material

Table S1-S26_Supplementary Material.xlsx
