# Supplementary material for: The haplotype-resolved T2T genome for Bauhinia × blakeana sheds light on the genetic basis of flower heterosis
Source: Gigascience. 2025 Apr 25;14:giaf044. doi: 10.1093/gigascience/giaf044 (PMC12012898; doi:10.1093/gigascience/giaf044)
Supplement: giaf044_Response_to_Reviewer_Comments_Original_Submission [file giaf044_response_to_reviewer_comments_original_submission.pdf]

Author's Response To Reviewer Comments

Reviewer reports:

Reviewer #1: This paper presents the genome assemblies of three Bauhinia species: B. purpurea, B. variegata, and B. blakeana. The third of these is an infertile hybrid. The paper presents evidence that B. blakeana and B. purpurea are the maternal and paternal parents, respectively. The largest portion of the analysis presented in the paper describes differential expression observed between the two homoeologous chromosomes from the parents.

The paper is, I think, exemplary in the rigor and completeness of its analyses. The assemblies all sound to be of very high quality. The differential expression analyses are difficult to conduct well; but I think that the completeness of the contributing genomes aids in producing compelling results.

I have the following recommendations:
1. There is a basic aspect of the cytogenetics that I think should be explicitly described early-on. Is the hybridization that produced B. blakeana due to contribution of one set of chromatids from B. variegata and one set from B. purpurea, such that the 2n (sporophytic) genome has 28 chromatids -- the same as in both parents? I assume this is the case, but it differs from other cases of wide hybridization that are followed by a genome doubling, such as Arachis hypogaea. In A. hypogaea, a merger of A. duranensis and A. ipaensis was followed by a genome doubling. Presumably, the genome doubling rescued the probably-sterile initial wide hybrid.
R: We thank the reviewer for these insightful comments. As suggested, we have addressed the cytogenetic basis of B. blakeana's hybrid origin in the manuscript (Page 5). Specifically, we have clarified that B. blakeana likely arose from the integration of one set of chromatids from each parent (B. purpurea and B. variegata), resulting in a diploid (2n) genome with 28 chromatids, consistent with both parental species. This distinguishes B. blakeana from other cases of wide hybridization followed by genome doubling, such as Arachis hypogaea, where genome duplication restored fertility in the hybrid. In B. blakeana, the absence of genome doubling likely contributes to its sterility due to irregular chromosome segregation and abnormal spindle formation during microsporogenesis.

2. It seems that it would be worthwhile to mention that Bauhinia (the genus) appears to have been the product of an allotetraploid merger, with double the number of chromosomes in the earlier-diverging genus Cercis. The relevant citation would be Zhong et al. (2022), which is already cited, in the context of the phylogeny of the Cercidoideae. This earlier result is relevant in that it would be another instance of a "wide hybrid" in the genus (with an additional genome doubling).
R: As recommended, we have mentioned the allotetraploid origin of the genus Bauhinia and its relationship to the earlier-diverging genus Cercis in the revised manuscript (Page 5). We believe this addition enriches the discussion on the evolutionary dynamics of Bauhinia and provides a broader context for understanding the genomic history of the genus.

3. (a grammatical error): pages 19-20: (four instances): "exhibited up-regulated" should be either "exhibited up-regulation" or "were up-regulated" ("up-regulated" is a past-participle of the verb "regulate," whereas "regulation" is a noun. The construction "exhibited upregulation" uses a verb-noun form.)
R: We appreciate the reviewer’s attention to detail. In the revised manuscript, we have corrected all instances of "exhibited up-regulated" to either "exhibited up-regulation" or "were up-regulated" as suggested (Pages 20-21).

Reviewer #2: Mu et al. reports a haplotype-resolved telomere-to-telomere (T2T) genome for B. blakeana plus chromosome-level assemblies for its putatively parental species. Furthermore several transcriptome-based gene expression analysis strengthens the quality of the paper. I have some comments listed below that are need to be addressed.

Major issue:
As confirmation of the hybrid origin of B. blakeana is one of most interesting topics, I would suggest to add

a further analysis regarding the issue. Although the genome sequences of chloroplasts were used in this study, the comparison of chloroplast genome sequences is not enough. Inference of hybridization still needs to be conducted using the whole genome data. Due to the difficulties in obtaining population genomic data, I recommend to use phytop, a software based on astral tree, which can be used to detect the hybridization in a preliminary manner (see: Hong-Yun Shang et al., Phytop: A tool for visualizing and recognizing signals of incomplete lineage sorting and hybridization using species trees output from ASTRAL, Horticulture Research, 2024;, uhae330, https://doi.org/10.1093/hr/uhae330).
R: We thank the reviewer for the insightful suggestion to further validate the hybrid origin of B. blakeana using whole-genome data. Following the recommended approach, we conducted phylogenomic analyses with two distinct datasets to investigate incomplete lineage sorting (ILS) and introgression (IH) signals:
Dataset 1: 214 Single-Copy Orthologs from 13 Species
This dataset included the 13 species used in our comparative genomics analysis. However, no significant ILS/IH signals were detected between the parental species (B. purpurea and B. variegata) or within the Bauhinia and Cercis genus nodes. We attribute this result to the limited number of orthologs and the potential evolutionary distance between the selected species, which may obscure hybridization signals.

Dataset 2: 2,360 Single-Copy Orthologs from 6 Taxa
To increase resolution, we analyzed a larger dataset comprising B. purpurea, B. variegata, the two haplotypes of B. blakeana (Hmat and Hpat), C. canadensis, C. chinensis, and A. thaliana as the outgroup. Despite the clear phylogenetic clustering of haplotypes with their respective parental species, no significant ILS/IH signals were detected at deeper nodes (e.g., Bauhinia or Cercis genus nodes). This suggests that the hybridization event leading to B. blakeana was likely a single, recent occurrence rather than a result of recurrent gene flow.

We have integrated the results from Dataset 2 into the revised manuscript (Pages 18–19, Figure 3D), including a detailed description of the phylogenomic analysis and its implications for the hybrid origin of B. blakeana.

Minor issues:
1) "high mapping rate (95.72% and 99.29%) and sequencing coverage (94.88% and 94.17%)", Dose the sequencing coverage here refers to the percentage of the genome sequence that was covered by WGS reads? How much x depth of these coverage data?
R: Yes, the sequencing coverage refers to the proportion of bases sequenced (covered by WGS reads) relative to the total genome size. The mean depth of coverage was 44.93x for B. purpurea and 61.83x for B. variegata, as detailed in Supplementary Table S2. These two assemblies were constructed using stLFR data, which is distinct from the WGS dataset used for assembly quality evaluation. During the calculation of the coverage ratio, we applied a quality filter (-q 10) in the BamDeal software to exclude low-quality WGS reads. Below is a summary of the relevant data:
Reads Bauhinia purpurea Bauhinia variegata
WGS Number of reads 173,500,900 200,296,314
Mapping rate 95.72% 99.29%
Mean depth 44.93 61.83
Coverage Rate 94.88% 94.17%
We have also clarified these points in the revised manuscript (Page 10)

2) Page 16, Figure 3a shows a large gap starting at approximately position 152k. Is this gap indicative of a sequence missing in NC061218 compared to the sequence in this study? If this is due to a structural variation, then this should be explained in the text.
R: We appreciate the reviewer’s attention to detail for identifying this inconsistency. To address this, we re-examined the original data and re-ran the mVISTA comparison with refined parameters. The revised Figure 3A confirms that the sequences of our assembled B. purpurea chloroplast genome and the published NC061218 are fully collinear, with no evidence of structural variations or missing sequences at this position.

3) Page20, "showing higher expression levels in B. variegata during the parental species comparison", What does this statement mean? Is it saying that the DEGs of B. blakeana vs B. purpurea are similar to

those of B. variegata vs B. purpurea? "Similar patterns were also found in the comparison between B. blakeana and B. variegata," where are the data, results or images for this?
R: To clarify, the statement indicates that the KEGG pathways enriched in up-regulated DEGs in B. blakeana (vs B. purpurea) overlapped with the pathways enriched in up-regulated DEGs in B. variegata (vs B. purpurea), as highlighted by arrows in Figure 5B. This functional overlap suggests that B. blakeana exhibits expression patterns resembling the higher-expressing parent (B. variegata) in these pathways. Similarly, when comparing B. blakeana vs B. variegata, we observed overlapping KEGG terms between up-regulated DEGs in B. blakeana and those enriched in B. purpurea (vs B. variegata), further supporting the functional high-parent dominance pattern. These results are presented in: Figure 5B: Arrows and color-coding (red for B. purpurea-dominant terms, blue for B. variegata-dominant terms) explicitly show the overlapping pathways; Supplementary Table S14, S15: Full lists of enriched KEGG terms for all comparisons.
We recognize that the original text could have been more explicit. In the revised manuscript, we have rephrased this section to emphasize functional convergence rather than direct gene-level overlap (Page 22).

4) page 21,"2,607 and 2,717 were up-regulated in B. blakeana when using B. purpurea and B. variegata as references," down-regulated in B. blakean could be also important and why only the up-regulation was analyzed here?
R: To clarify, both up- and down-regulated DEGs in the B. blakeana vs mid-parent value (MPV) comparisons were analyzed for their functional implications. KEGG enrichment analyses were performed separately for: all DEGs (up- and down-regulated combined); up-regulated DEGs and down-regulated DEGs. The complete results are provided in Supplementary Table S16.
In the original text, we focused on up-regulated DEGs to highlight the functional categories potentially contributing to heterosis. However, we fully agree that down-regulated DEGs are equally important. For example, down-regulated genes in B. blakeana were enriched in pathways such as “Photosynthesis” and “Biosynthesis of secondary metabolites” (Supplementary Table S16B, E), suggesting potential energy reallocation towards heterosis-related traits.
In the revised manuscript, we have added a discussion of down-regulated DEGs to provide a more balanced perspective (Page 23).

5) page22, What is ASE? The acronym needs to be explained when appearing for the first time. "6,934 genes as ASEGs", according to the diagnostic SNP table, did these ASE Genes refer to genes which reads can be uniquely align to maternal or paternal alleles?
Interestingly, did the ASEGs overlap significantly with the differentially expressed genes mentioned in page20?: "Notably, the number of DEGs between B. blakeana and B. variegata was approximately twice as large as the number of DEGs between B. blakeana and B. purpurea, suggesting a stronger resemblance in the gene expression profile of B. blakeana to its maternal parent, B. purpurea (Figure 5A)." Also, is there a significant overlap between ASEGs and the MPV-hybrid DEGs in the previous paragraph?

R: Below, we address each point in detail:
1. Definition of ASE: Allele-specific expression (ASE) refers to the phenomenon where the two alleles of a gene in a diploid organism exhibit differential expression. This term is now defined upon its first mention in the Background section (Page 8).

2. ASEG Identification: The 6,934 ASEGs were identified using the HyLiTE pipeline, which was a diagnostic SNP-based method. In brief, RNA-seq reads from B. blakeana were aligned to the B. purpurea reference genome (cds sequences), and diagnostic SNPs fixed between B. purpurea and B. variegata were used to assign reads to maternal or paternal alleles. Reads uniquely assigned to either allele was used to manually construct an expression matrix for DESeq2 analysis. Full methodological details were also revised and provided in the Methods section (Page 48-49).

3. Overlap Between ASEGs and DEGs/ MPV-hybrid DEGs: We acknowledge the reviewer’s interest in exploring potential overlaps between ASEGs and DEGs. However, the reference genome dependency complicates direct comparisons. The DEGs identified in B. blakeana vs parental species comparisons (Page 21) and ASEGs (Page 25-26) were analyzed using different reference genomes (B. purpurea/B. variegata for DEGs vs B. blakeana metagenome for ASEGs). This discrepancy prevents direct overlap analysis at the

gene level.

6) page23, "Furthermore, GO enrichment analysis of these ASEGs provided valuable insights into their functional implications" Why was only GO analysis performed here and not KEGG analysis? For the two methods of identifying ASEGs, dose the first method utilizes snp specific to Hmat and Hpat? How does the second method that uses DESeq2 to identify ASEGs? Please give a more detailed description on these methods.

R: Below, we address each point in detail:

1. GO vs KEGG Enrichment Analysis:

To address this point, we have now incorporated KEGG pathway enrichment analysis into the ASEG functional characterization (Page 26). These findings complement the GO analysis and highlight the involvement of ASEGs in critical processes such as DNA repair, stress adaptation, and metabolic regulation.

2. Methodological Details for ASEG Identification

Method 1: Diagnostic SNP-Based ASEG Identification:

RNA-seq reads from B. blakeana and its parents (B. purpurea and B. variegata) were aligned to the B. purpurea reference genome. HyLiTE identified diagnostic SNPs (positions fixed for alternative alleles between the parents) to distinguish maternal and paternal alleles in the hybrid. Reads uniquely mapped to either maternal or paternal alleles were extracted and quantified. DESeq2 was applied to allelic counts to identify ASEGs.

Method 2: Haplotype-Resolved ASEG Identification:

A metagenome was constructed by combining the Hmat and Hpat haplotypes of B. blakeana. RNA-seq reads from B. blakeana were aligned to the metagenome, and allele-specific counts were quantified using featureCounts. DESeq2 was applied to the allele-specific count matrix to identify ASEGs.

Method 1 relies on diagnostic SNPs between the parental genomes, which may miss allele-specific events in regions with low SNP density or high sequence divergence.

Method 2 leverages haplotype-resolved assemblies, providing a more comprehensive view of allele-specific expression across the entire genome.

More detailed descriptions of these methods are added in the Methods section (Page 48-49) as recommended.

7) Page 29, ASEGs were mentioned on page 22 as well. Are the identification methods the same for both cases (page22 and 29)?

R: We thank the reviewer for this critical question. The ASEG identification methods in both sections utilized the same data set (RNA-seq reads aligned to the haplotype-resolved metagenome to generate an allelic count matrix), the analytical thresholds were purposefully tailored to address distinct biological questions:

1. Genome-wide ASEG identification (Page 25-26):

A stringent statistical framework was applied using DESeq2 ($P < 0.01$, $|log2FC| > 1$) to identify ASEGs with robust genome-wide significance. This approach prioritized minimizing false positives across the entire transcriptome.

2. Pathway-specific ASEG profiling (Page 31):

We relaxed the statistical threshold to capture ASEGs with reproducible allelic biases (maternal/paternal ASE ratio >0.7 or <0.3 in ≥ 2 replicates), even if they did not meet genome-wide significance. This adjustment aimed to expand the candidate pool of ASEGs within functionally critical pathways, enabling a more comprehensive analysis of allele-specific contributions to flower color. Prioritize biological relevance in key metabolic pathways where subtle allelic imbalances may disproportionately impact phenotypic outcomes.

We have revised the Methods and Results sections to explicitly distinguish these two methodologies (Page 31, Page 49-50).

8) Page 32, "we found that even though B. blakeana exhibits flower color that is more similar to its paternal parent, the general gene expression profile of B. blakeana aligns more closely with its maternal

parent." "We observed that the number of genes exhibiting up-regulated expression in B. blakeana is comparatively lower than the number of genes showing down-regulated expression when compared to the MPV." These statements need to be supported by citing results.
R: We have revised the text to explicitly cite supporting results (Page 34).

9) Page 33, "The pronounced variations in expression levels observed within the carotenoid biosynthesis pathways between the parental species may be associated with the higher proportion of genes exhibiting dominance complementation or over-dominance expression patterns in B. blakeana," I do not understand why the variations in expression levels between the parents are related to the dominance expression patterns in B. blakeana? Should it not be the expression differences between B. blakeana and its parents that are related to the dominance expression patterns?
R: To clarify the link between parental expression divergence and hybrid dominance patterns, we have revised the text and provided additional mechanistic insights based on recent literature (Page 36).

In Figure 7, the metabolic processes of the pathway are shown. An analysis of maternal and paternal allele dominance as well as ASE may be added to discuss what potential consequences might arise in the metabolism related to flower color? Which metabolites or processes in pigment biosynthesis might be enhanced or weakened due to allele dominance, and what impact could this have on phenotypes such as flower color? How are dominance expression patterns and ASE related to heterosis? Which genes in these pathways belong to dominance complementation, and which belong to over-dominance?
What specific roles might ASE play? Overall, it is too simple and preliminary to state that ASE and dominance expression patterns are related to heterosis.
R: We thank the reviewer for emphasizing the need to deepen our analysis of ASE and dominance patterns in the context of metabolic pathways. We have addressed these points as follows:
1. In Figure 7A-C, we have highlighted genes exhibiting maternal allele dominance (bold italic gene copy ID in red) and paternal allele dominance (bold italic gene copy ID in blue). In Figure 7D, we marked genes with high-parent dominance (pink dot behind the gene ID) and over-dominance (yellow dot behind the gene ID) to clearly distinguish their expression patterns. We have also listed the gene in the result part (Page 29). These annotations provide a comprehensive view of how allele-specific regulation and dominance patterns are distributed across the carotenoid, anthocyanin, and chlorophyll biosynthesis pathways.
2. We have expanded the discussion in the revised text (Pages 36–37) to address how dominance expression patterns and ASE influence metabolic flux and likely contribute to flower color heterosis.

In addition, the text mentioned "The carotenoid biosynthesis pathway displayed the highest proportion of ASEGs, consistent with the higher proportion of genes exhibiting dominance complementation or over-dominance expression patterns in this pathway." However, in the heatmap, many gene expressions in BLA seem weaker than those in VAR, which seems to contradict the patterns of dominance expression.
R: We have revised the manuscript and removed the mentioned text. However, based on Figure 7D, we observed that within the carotenoid biosynthesis pathway (including "MEP Pathway", "Carotene Biosynthesis", and "Xanthophylls Biosynthesis"), there are 11 genes displaying dominance complementation or over-dominance expression patterns, as indicated by the presence of pink and yellow dots. The heatmap representation of these genes aligns with the expected dominance complementation or over-dominance expression patterns.
