# Supplementary material for: The haplotype-resolved T2T genome for Bauhinia × blakeana sheds light on the genetic basis of flower heterosis
Source: Gigascience. 2025 Apr 25;14:giaf044. doi: 10.1093/gigascience/giaf044 (PMC12012898; doi:10.1093/gigascience/giaf044)
Supplement: giaf044_Reviewer_1_Report_Original_Submission — Steven Cannon -- 12/23/2024 [file giaf044_reviewer_1_report_original_submission.pdf]

**Reviewer Report**

**Title: The Haplotype-resolved T2T Genome for Bauhinia x blakeana Sheds Light on the Genetic Basis of Flower Heterosis**

**Version: Original Submission** **Date:** 12/23/2024

**Reviewer name: Steven Cannon**

**Reviewer Comments to Author:**

This paper presents the genome assemblies of three Bauhinia species: B. purpurea, B. variegata, and B. blakeana. The third of these is an infertile hybrid. The paper presents evidence that B. blakeana and B. purpurea are the maternal and paternal parents, respectively. The largest portion of the analysis presented in the paper describes differential expression observed between the two homoeologous chromosomes from the parents.
The paper is, I think, exemplary in the rigor and completeness of its analyses. The assemblies all sound to be of very high quality. The differential expression analyses are difficult to conduct well; but I think that the completeness of the contributing genomes aids in producing compelling results.
I have the following recommendations:
1. There is a basic aspect of the cytogenetics that I think should be explicitly described early-on. Is the hybridization that produced B. blakeana due to contribution of one set of chromatids from B. variegata and one set from B. purpurea, such that the 2n (sporophytic) genome has 28 chromatids -- the same as in both parents? I assume this is the case, but it differs from other cases of wide hybridization that are followed by a genome doubling, such as Arachis hypogaea. In A. hypogaea, a merger of A. duranensis and A. ipaensis was followed by a genome doubling. Presumably, the genome doubling rescued the probably-sterile initial wide hybrid.
2. It seems that it would be worthwhile to mention that Bauhinia (the genus) appears to have been the product of an allotetraploid merger, with double the number of chromosomes in the earlier-diverging genus Cercis. The relevant citation would be Zhong et al. (2022), which is already cited, in the context of the phylogeny of the Cercidoideae. This earlier result is relevant in that it would be another instance of a "wide hybrid" in the genus (with an additional genome doubling).
3. (a grammatical error): pages 19-20: (four instances): "exhibited up-regulated" should be either "exhibited up-regulation" or "were up-regulated" ("up-regulated" is a past-participle of the verb "regulate," whereas "regulation" is a noun. The construction "exhibited upregulation" uses a verb-noun form.)

**Methods**

Are the methods appropriate to the aims of the study, are they well described, and are necessary controls included? Choose an item.

**Conclusions**

Are the conclusions adequately supported by the data shown? Choose an item.

**Reporting Standards**

Does the manuscript adhere to the journal's guidelines on [minimum standards of reporting?]() Choose an item.

Choose an item.

**Statistics**

Are you able to assess all statistics in the manuscript, including the appropriateness of statistical tests used? Choose an item.

**Quality of Written English**

Please indicate the quality of language in the manuscript: Choose an item.

**Declaration of Competing Interests**

Please complete a declaration of competing interests, considering the following questions:

- Have you in the past five years received reimbursements, fees, funding, or salary from an organisation that may in any way gain or lose financially from the publication of this manuscript, either now or in the future?
- Do you hold any stocks or shares in an organisation that may in any way gain or lose financially from the publication of this manuscript, either now or in the future?
- Do you hold or are you currently applying for any patents relating to the content of the manuscript?
- Have you received reimbursements, fees, funding, or salary from an organization that holds or has applied for patents relating to the content of the manuscript?
- Do you have any other financial competing interests?
- Do you have any non-financial competing interests in relation to this paper?

If you can answer no to all of the above, write 'I declare that I have no competing interests' below. If your reply is yes to any, please give details below.

I declare that I have no competing interests

I agree to the open peer review policy of the journal. I understand that my name will be included on my report to the authors and, if the manuscript is accepted for publication, my named report including any attachments I upload will be posted on the website along with the authors' responses. I agree for my report to be made available under an Open Access Creative Commons CC-BY license (http://creativecommons.org/licenses/by/4.0/). I understand that any comments which I do not wish to be included in my named report can be included as confidential comments to the editors, which will not be published.

Choose an item.

To further support our reviewers, we have joined with Publons, where you can gain additional credit to further highlight your hard work (see: https://publons.com/journal/530/gigascience). On publication of this paper, your review will be automatically added to Publons, you can then choose whether or not to claim your Publons credit. I understand this statement.

Yes Choose an item.
