# Supplementary material for: The haplotype-resolved T2T genome for Bauhinia × blakeana sheds light on the genetic basis of flower heterosis
Source: Gigascience. 2025 Apr 25;14:giaf044. doi: 10.1093/gigascience/giaf044 (PMC12012898; doi:10.1093/gigascience/giaf044)
Supplement: giaf044_Reviewer_2_Report_Original_Submission — Yongpeng Ma -- 12/29/2024 [file giaf044_reviewer_2_report_original_submission.pdf]

Reviewer Report

Title: The Haplotype-resolved T2T Genome for Bauhinia x blakeana Sheds Light on the Genetic Basis of Flower Heterosis

Version: Original Submission **Date:** 12/29/2024

Reviewer name: Yongpeng Ma

Reviewer Comments to Author:

Mu et al. reports a haplotype-resolved telomere-to-telomere (T2T) genome for B. blakeana plus chromosome-level assemblies for its putatively parental species. Furthermore several transcriptome-based gene expression analysis strengthens the quality of the paper. I have some comments listed below that are need to be addressed.
Major issue:
As confirmation of the hybrid origin of B. blakeana is one of most interesting topics, I would suggest to add a further analysis regarding the issue. Although the genome sequences of chloroplasts were used in this study, the comparison of chloroplast genome sequences is not enough. Inference of hybridization still needs to be conducted using the whole genome data. Due to the difficulties in obtaining population genomic data, I recommend to use phytop, a software based on astral tree, which can be used to detect the hybridization in a preliminary manner (see: Hong-Yun Shang et al., Phytop: A tool for visualizing and recognizing signals of incomplete lineage sorting and hybridization using species trees output from ASTRAL, Horticulture Research, 2024;, uhae330, https://doi.org/10.1093/hr/uhae330).
Minor issues:
1) "high mapping rate (95.72% and 99.29%) and sequencing coverage (94.88% and 94.17%)", Dose the sequencing coverage here refers to the percentage of the genome sequence that was covered by WGS reads? How much x depth of these coverage data?
2) Page 16, Figure 3a shows a large gap starting at approximately position 152k. Is this gap indicative of a sequence missing in NC061218 compared to the sequence in this study? If this is due to a structural variation, then this should be explained in the text.
3) Page20ï¼Œ"showing higher expression levels in B. variegata during the parental species comparison"ï¼ŒWhat does this statement mean? Is it saying that the DEGs of B. blakeana vs B. purpurea are similar to those of B. variegata vs B. purpurea? "Similar patterns were also found in the comparison between B. blakeana and B. variegata," where are the data, results or images for this?
4) page 21,"2,607 and 2,717 were up-regulated in B. blakeana when using B. purpurea and B. variegata as references," down-regulated in B. blakean could be also important and why only the up-regulation was analyzed here?
5) page22, What is ASE? The acronym needs to be explained when appearing for the first time. "6,934 genes as ASEGs", according to the diagnostic SNP table, did these ASE Genes refer to genes which reads can be uniquely align to maternal or paternal alleles? Interestingly, did the ASEGs overlap significantly with the differentially expressed genes mentioned in page20?: "Notably, the number of DEGs between B. blakeana and B. variegata was approximately twice as large as the number of DEGs between B.

blakeana and B. purpurea, suggesting a stronger resemblance in the gene expression profile of B. blakeana to its maternal parent, B. purpurea (Figure 5A)." Also, is there a significant overlap between ASEGs and the MPV-hybrid DEGs in the previous paragraph?
6) page23ï¼Œ"Furthermore, GO enrichment analysis of these ASEGs provided valuable insights into their functional implications" Why was only GO analysis performed here and not KEGG analysis? For the two methods of identifying ASEGs, dose the first method utilizes snp specific to Hmat and Hpat? How does the second method that uses DESeq2 to identify ASEGs? Please give a more detailed description on these methods.
7) Page 29, ASEGs were mentioned on page 22 as well. Are the identification methods the same for both cases (page22 and 29)?
8) Page 32, "we found that even though B. blakeana exhibits flower color that is more similar to its paternal parent, the general gene expression profile of B. blakeana aligns more closely with its maternal parent." "We observed that the number of genes exhibiting up-regulated expression in B. blakeana is comparatively lower than the number of genes showing down-regulated expression when compared to the MPV." These statements need to be supported by citing results.
9) Page 33, "The pronounced variations in expression levels observed within the carotenoid biosynthesis pathways between the parental species may be associated with the higher proportion of genes exhibiting dominance complementation or over-dominance expression patterns in B. blakeana," I do not understand why the variations in expression levels between the parents are related to the dominance expression patterns in B. blakeana? Should it not be the expression differences between B. blakeana and its parents that are related to the dominance expression patterns?
In Figure 7, the metabolic processes of the pathway are shown. An analysis of maternal and paternal allele dominance as well as ASE may be added to discuss what potential consequences might arise in the metabolism related to flower color? Which metabolites or processes in pigment biosynthesis might be enhanced or weakened due to allele dominance, and what impact could this have on phenotypes such as flower color? How are dominance expression patterns and ASE related to heterosis? Which genes in these pathways belong to dominance complementation, and which belong to over-dominance? What specific roles might ASE play? Overall, it is too simple and preliminary to state that ASE and dominance expression patterns are related to heterosis.
In addition, the text mentioned "The carotenoid biosynthesis pathway displayed the highest proportion of ASEGs, consistent with the higher proportion of genes exhibiting dominance complementation or over-dominance expression patterns in this pathway." However, in the heatmap, many gene expressions in BLA seem weaker than those in VAR, which seems to contradict the patterns of dominance expression.

Methods

Are the methods appropriate to the aims of the study, are they well described, and are necessary controls included? Choose an item.

Conclusions

Are the conclusions adequately supported by the data shown? Choose an item.

Reporting Standards

Does the manuscript adhere to the journal's guidelines on minimum standards of reporting? Choose an item.

Choose an item.

Statistics

Are you able to assess all statistics in the manuscript, including the appropriateness of statistical tests used? Choose an item.

Quality of Written English

Please indicate the quality of language in the manuscript: Choose an item.

Declaration of Competing Interests

Please complete a declaration of competing interests, considering the following questions:

- Have you in the past five years received reimbursements, fees, funding, or salary from an organisation that may in any way gain or lose financially from the publication of this manuscript, either now or in the future?
- Do you hold any stocks or shares in an organisation that may in any way gain or lose financially from the publication of this manuscript, either now or in the future?
- Do you hold or are you currently applying for any patents relating to the content of the manuscript?
- Have you received reimbursements, fees, funding, or salary from an organization that holds or has applied for patents relating to the content of the manuscript?
- Do you have any other financial competing interests?
- Do you have any non-financial competing interests in relation to this paper?

If you can answer no to all of the above, write 'I declare that I have no competing interests' below. If your reply is yes to any, please give details below.

I declare that I have no competing interests

I agree to the open peer review policy of the journal. I understand that my name will be included on my report to the authors and, if the manuscript is accepted for publication, my named report including any attachments I upload will be posted on the website along with the authors' responses. I agree for my report to be made available under an Open Access Creative Commons CC-BY license (http://creativecommons.org/licenses/by/4.0/). I understand that any comments which I do not wish to

be included in my named report can be included as confidential comments to the editors, which will not be published.

Choose an item.

To further support our reviewers, we have joined with Publons, where you can gain additional credit to further highlight your hard work (see: https://publons.com/journal/530/gigascience). On publication of this paper, your review will be automatically added to Publons, you can then choose whether or not to claim your Publons credit. I understand this statement.

Yes Choose an item.
